# Protein thiol alterations drive pathologic liquid–liquid phase separation in the aging brain

**DOI:** 10.1038/s41594-026-01857-w

**Published:** 2026-07-30

**Authors:** Thibaut Vignane, Martín Hugo, Christian Hoffmann, Polina Reichert, Antonia Katsouda, Davide D’Andrea, Han Wang, Johannes V. Tromm, Marko Miler, Ferran Comas, Dunja Petrovic, Suyuan Chen, Jan Lj. Miljkovic, Danail Stoychev, Erik Lacko, Vladimir M. Jovanovic, Suwarna Chakraborty, Sunil J. Tripathi, Edwin Vázquez-Rosa, Jordan L. Morris, Suvagata Roy Chowdhury, Julien Prudent, Albert Sickmann, Natalija Polovic, Andrew A. Pieper, Ilan Davis, Kostas Tokatlidis, Bindu D. Paul, Michael P. Murphy, Christian Münch, Andreas Papapetropoulos, Dragomir Milovanovic, Milos R. Filipovic

**Affiliations:** 1 Leibniz Institute for Analytical Sciences, ISAS, e.V., Dortmund, Germany; 2 Institute of Molecular Systems Medicine, Faculty of Medicine, Goethe University, Frankfurt, Germany; 3 Institute of Biochemistry, Charité-Universitätsmedizin Berlin, Corporate Member of Freie Universität Berlin, Humboldt-Universität Berlin, and Berlin Institute of Health, Berlin, Germany; 4 School of Molecular Biosciences, MVLS College, University of Glasgow, Glasgow, UK; 5 Clinical, Experimental Surgery and Translational Research Center, Biomedical Research Foundation of the Academy of Athens, Athens, Greece; 6 Laboratory of Pharmacology, Department of Pharmacy, National and Kapodistrian University of Athens, Athens, Greece; 7 MRC Mitochondrial Biology Unit, University of Cambridge, Cambridge, UK; 8 Bioinformatics Solution Center, Institute for Informatics, Freie Universität Berlin, Berlin, Germany; 9 Department of Pharmacology and Molecular Sciences, Johns Hopkins University School of Medicine, Baltimore, MD USA; 10 Department of Psychiatry, Case Western Reserve University, Cleveland, OH USA; 11 Brain Health Medicines Center, Harrington Discovery Institute, University Hospitals Cleveland Medical Center, Cleveland, OH USA; 12 Geriatric Psychiatry, GRECC, Louis Stokes Cleveland VA Medical Center, Cleveland, OH USA; 13 Institute for Transformative Molecular Medicine, Case Western Reserve University School of Medicine, Cleveland, OH USA; 14 Department of Biochemistry, Faculty of Chemistry, University of Belgrade, Belgrade, Serbia; 15 Department of Pathology, Case Western Reserve University School of Medicine, Cleveland, OH USA; 16 Department of Neurosciences, Case Western Reserve University School of Medicine, Cleveland, OH USA; 17 The Solomon H. Snyder Department of Neuroscience, Johns Hopkins University School of Medicine, Baltimore, MD USA; 18 Department of Psychiatry and Behavioral Sciences, Johns Hopkins University School of Medicine, Baltimore, MD USA; 19 Lieber Institute for Brain Development, Baltimore, MD USA; 20 Department of Medicine, University of Cambridge, Cambridge, UK; 21 Present Address: Serra Húnter Fellow, Departament de Bioquimica i Biologia Molecular, Universitat Autònoma de Barcelona, Bellaterra, Barcelona, Spain

**Keywords:** Proteomics, Post-translational modifications

## Abstract

Cellular homeostasis relies on regulation of processes, including protein post-translational modifications (PTMs) and biomolecular condensation. Aging disrupts the equilibrium of these processes, increasing susceptibility to disease and mortality. Here we used chemoproteomic techniques to generate an atlas of cysteine PTMs in the mouse brain and showed that age-related increases in thiol oxidation promoted the formation of biomolecular condensates. By contrast, protein persulfidation, regulated by hydrogen sulfide production, inhibited biomolecular condensation, preserving protein function. Age-induced alterations in cysteine PTMs influenced the phase separation properties of synapsin 1 and G3BP2, leading to impaired neurotransmitter release and defective stress granule formation and resolution, features associated with aging and neurodegenerative diseases. Mice deficient in cystathionine γ-lyase, the enzyme responsible for hydrogen sulfide production, exhibited reduced lifespans and spontaneously developed protein aggregates with age. Our results highlight the therapeutic potential of protein persulfidation in reversal of dysregulated biomolecular condensation and suggest that sulfide donors could be used to mitigate age-related diseases.

## Abstract (web summary)

Vignane et al. show that age-related protein oxidation disrupts normal brain protein organization, promoting harmful condensates and aggregation linked to neurodegeneration, whereas hydrogen sulfide-driven persulfidation counteracts these effects.

## Main

**Fig. 1:**
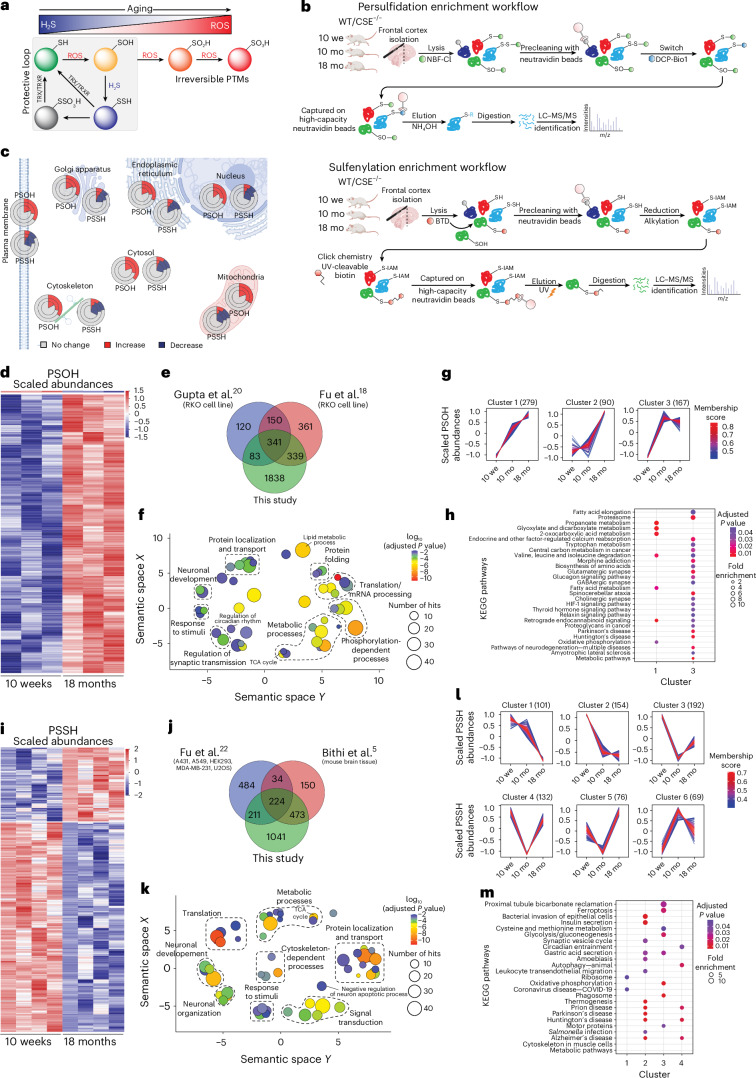
Chemoproteomic analysis of protein sulfenylation and persulfidation reveals substantial alteration of cysteine modifications in aging. **a**, Protective persulfidation loop prevents cysteine hyperoxidation. **b**, Chemoproteomic workflows of PSOH and PSSH detection. The PSSH workflow was based on that of Zivanovic et al.^4^, whereas the PSOH workflow was adapted from work by Fu et al.^18^. **c**, PSOH and PSSH changes in subcellular compartments. From inside to outside, circles indicate changes in 10-month-old and 18-month-old WT mice and 10-week-old *Cse*^−/−^ mice compared to 10-week-old WT animals, respectively. Color denotes the type of change, with red indicating a significant increase and blue a significant decrease, whereas gray represents the proportion of proteins in which the cysteine modification did not change. Proteins with multiple localization are also presented. **d**, Heatmap showing significant changes in PSOH in 18-month-old mice compared to 10-week-old mice (*n* = 3 animals; Welch’s *t*-test with FDR-adjusted *P* ≤ 0.05, fold change > 30%). **e**, Venn diagram comparing sulfenylated proteins identified in our study against two other datasets (from Fu et al.^18^ and Gupta et al.^20^). **f**, GO term enrichment (biological process) of proteins showing significant increases in PSOH (*n* = 3 animals; Welch’s *t*-test with FDR-adjusted *P* ≤ 0.05, fold change > 30%) at least once across the age. Enrichment was performed using DAVID, and outcomes were visualized with REVIGO. Significant GO terms were defined as those that passed the Benjamini-adjusted *P* value threshold of 0.01. Circle dimensions correspond to the protein count within specific GO terms, and color gradients indicate significance. **g**, Hierarchical cluster analysis of proteins with significant changes in PSOH levels (*n* = 3 animals; Welch’s *t*-test with FDR-adjusted *P* ≤ 0.05, fold change > 30%) at least once for all 3 ages based on trend profiles. **h**, KEGG pathway enrichment analysis for each cluster shown in **g**. Benjamini-adjusted *P* values were used to filter for significant enrichment. **i**, Heatmap showing significant changes in PSSH in 18-month-old mice compared to 10-week-old mice (*n* = 4 animals, Welch’s *t*-test with FDR-adjusted *P* ≤ 0.05). **j**, Venn diagram comparing persulfidated proteins identified in our study against two other datasets (from Fu et al.^22^ and Bithi et al.^5^). **k**, GO term enrichment (biological process) of proteins showing significant decrease in PSSH at least once for all 3 ages based on trend profiles (*n* = 4 animals, Welch’s *t*-test with FDR-adjusted *P* < 0.05 and fold change > 30%). **l**, Hierarchical clustering of proteins for which persulfidation was found to change at least once for all 3 ages (*n* = 4 animals, Welch’s *t*-test with FDR-adjusted *P* < 0.05 and fold change > 30%) into 6 groups based on trend profiles. **m**, Corresponding KEGG pathway enrichment analysis for the clusters shown in **l**. Benjamini-adjusted *P* values were used to filter for significant enrichment. Figure created in BioRender; Filipovic, M. https://biorender.com/kveknhn (2026). mo, months; TCA, tricarboxylic acid; we, weeks.

Aging is characterized as a progressive decline in physiological fitness, driven by accumulation of both random and deterministic deleterious changes^1^. The redox chemistry of sulfur-containing amino acid cysteine^2^ makes it a key target for age-related modifications and a critical component of cellular defense against oxidative damage^3^. Recent studies suggest that cysteine persulfidation (PSSH), an evolutionarily conserved post-translational modification (PTM) of proteins mediated by gasotransmitter hydrogen sulfide (H_2_S), may protect proteins from excessive oxidation and functional loss^4^ (Fig. 1a). Cystathionine γ-lyase (CSE; gene name: *CTH*), is a key enzyme in H_2_S production that regulates PSSH levels^4,5^ and was recently proposed to be a conserved prolongevity gene in RNA sequencing analyses of lifespan-extending interventions in mice^6,7^. This is particularly relevant in the aging brain, in which reduced CSE expression is associated with neurodegenerative diseases^8–10^. Restoring PSSH levels has been shown to extend lifespan, improve healthspan^11^ and mitigate cognitive decline associated with Alzheimer’s disease^8^. However, the traditional view that cysteine modifications primarily affect only protein function may not fully capture their biological impact. Indeed, emerging evidence suggests that protein function can also be regulated through intracellular phase transitions into membraneless compartments, a process known as liquid–liquid phase separation (LLPS)^12,13^. Here we hypothesize that the age-induced switch from PSSH toward oxidation of cysteine to sulfenic acid (PSOH) and finally sulfonic acid (PSO_3_H) affects the ability of proteins to phase separate and promotes their aggregation (Fig. 1a). Such changes may impose additional stress on aging cells, driving them toward pathophysiological states.

## Results

### Aging is associated with a proteome-wide increase in PSOH and decrease in PSSH

Conventional methods for detecting thiol oxidation^14^ often fail to differentiate between PSOH and PSSH^15^, as both modifications are oxidative in nature and intrinsically interconnected, despite their distinct biological roles^2,4,16,17^. These modifications are highly reactive and short-lived; therefore, selective labeling techniques are needed to accurately assess their dynamics (Supplementary Text). To investigate the impact of aging on thiol oxidation, we used selective chemoproteomic approaches to globally detect PSOH and PSSH^4,18^ in the frontotemporal region of mouse brain at 10 weeks, 10 months and 18 months of age (Fig. 1b). We focused on the frontotemporal region because it is particularly vulnerable to age-associated neurodegeneration and exhibits early molecular changes relevant to cognitive decline.

**Extended Data Fig. 1:**
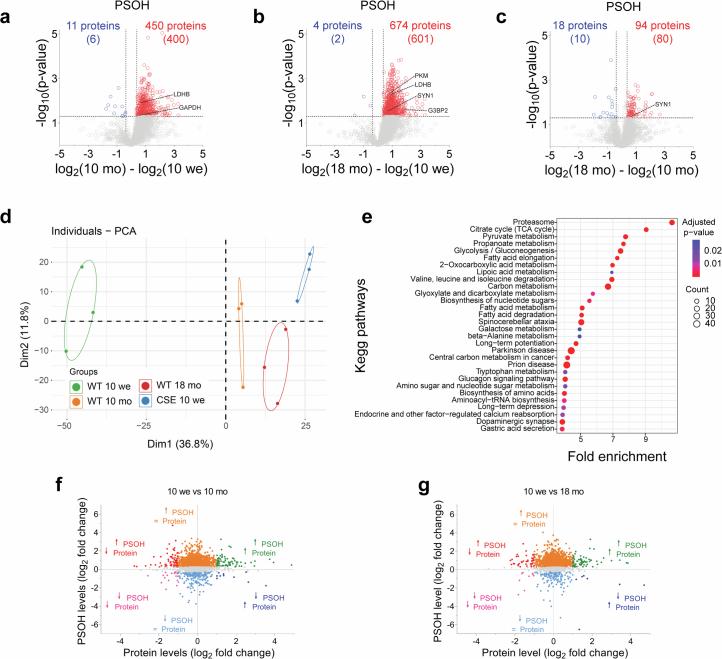
Quantitative sulfenylomics analysis of aging brain. (**a-c**) Volcano plot analysis of PSOH changes (n = 3 animals, Welch’s t-test) in (**a**) 10-month-old mice and (**b**) 18-month-old mice relative to 10-week-old mice and (**c**) 18-month-old mice relative to 10-month-old mice. The number of proteins with significantly altered PSOH levels is indicated, with the number in parentheses reflecting proteins identified through a minimum of 2 unique peptides. (**d**) Principal component analysis (PCA) underscoring the reproducibility of sulfenylome measurements across all replicates. (**e**) KEGG pathway enrichment analysis of proteins showing significant PSOH changes with age. The graph shows the top 30 significant (Benjamini adjusted p-value ≤ 0.05) and most enriched pathways, with the color gradient indicating adjusted p-values and circle size the number of proteins. (**f-g**) Scatter plot analysis of sulfenylome and protein expression relationships. log2 fold changes within the sulfenylome (y-axis) were plotted against total proteome changes (x-axis) for the (**f**) 10-month-old vs. 10-week-old mice and (**g**) 18-month-old vs. 10-week-old mice.

Sulfenylated proteins were identified across most cellular compartments (Fig. 1c). Although PSOH is considered to be a transient modification and an early step in thiol oxidation with multiple fates (Fig. 1a), we observed a steady, age-dependent increase levels of sulfenylated proteins (Fig. 1d, Extended Data Fig. 1a–c and Supplementary Table 1). Principal component analysis (PCA) revealed clear separation between the sulfenylomes of young and old mice (Extended Data Fig. 1d). Notably, a recent study demonstrated that benzo[c][1,2]thiazine-based chemoselective probe (BTD) is highly specific for PSOH, with no side reactivities^19^. Leveraging this probe, we opted for total protein enrichment and identified a substantially higher number of endogenously sulfenylated proteins (Fig. 1e) compared to previous studies that used site-centric peptide enrichment^18,20^.

Proteins with significantly elevated PSOH levels in aging were primarily associated with metabolic processes and protein translation, folding, localization and transport, as well as regulation of phosphorylation, synaptic transmission, response to stimuli, actin and cytoskeleton organization, and neuronal development (Fig. 1f, Extended Data Fig. 1e and Supplementary Table 1). To discern age-dependent patterns, we clustered proteins using the fuzzy *c*-means method. Approximately 20% of sulfenylated proteins exhibited significant changes (false discovery rate (FDR)-adjusted *P* ≤ 0.05) in PSOH levels with age, with about half showing a continuous age-dependent increase (Fig. 1g). These proteins were enriched in critical neuronal pathways (for example, synaptic vesicle cycle, carbon metabolism, proteasome) and highly implicated in neurodegenerative diseases (amyotrophic lateral sclerosis, Parkinson’s disease, spinocerebellar ataxia, Huntington’s disease) (Fig. 1h).

**Extended Data Fig. 2:**
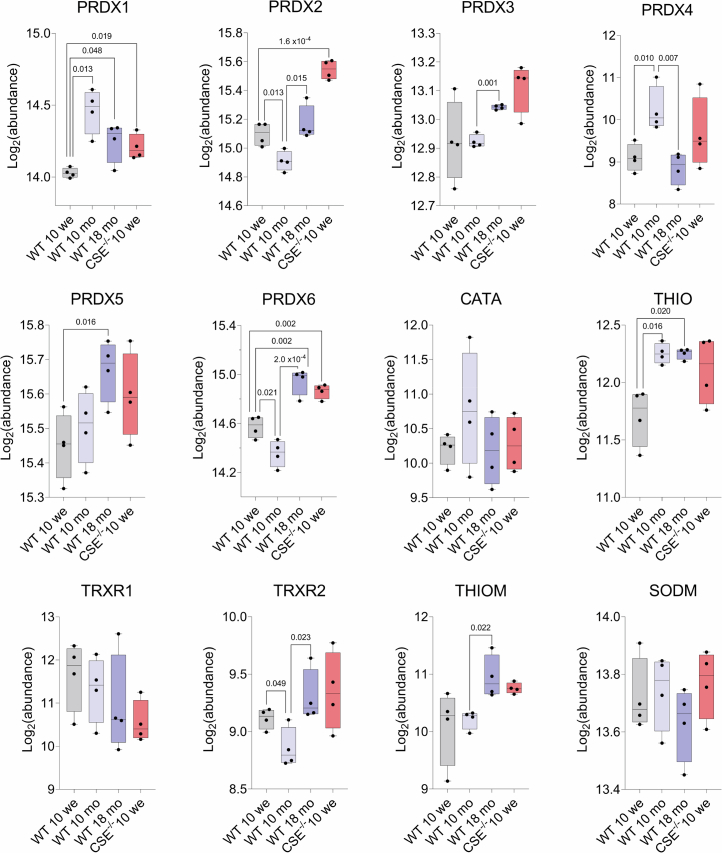
Expression profiles of antioxidant proteins across aging and in Cse^−/−^ mice. Whiskers and box plots of the log2 transformed abundancies of antioxidant proteins identified in the proteome analysis. Box plots show median (centre line), 25th–75th percentiles (box bounds), and min and max values (whiskers). Reported significance levels accompanying the plots are directly correlated with the p-values calculated through the proteome analysis (n = 4, Welch’s t-test for each comparison). PRDX (1-6): peroxiredoxin (1-6); CATA: Catalase; THIO: thioredoxin, THIOM: mitochondrial thioredoxin; TRXR (1-2): thioredoxin reductase (1-2); SODM: mitochondrial superoxide dismutase. Source data

To exclude the possibility that these changes stemmed from variations in protein expression, we performed label-free quantitative proteomic analysis on the same samples (Supplementary Fig. 1 and Supplementary Table 2). Although aging did affect the brain proteome to some extent (Supplementary Fig. 1), consistent with previous reports^21^, the observed changes in sulfenylation were not attributable to alterations in protein expression (Extended Data Fig. 1f,g). Specifically, plotting sulfenylome changes against total proteome changes showed minimal scattering, with only a few PSOH targets following protein expression trends (Extended Data Fig. 1f, g). These findings indicate that the age-related increase in steady-state PSOH levels (Fig. 1d) reflects heightened oxidative stress in the aging brain; this is further supported by changes in the levels of key antioxidant enzymes (Extended Data Fig. 2).

**Extended Data Fig. 3:**
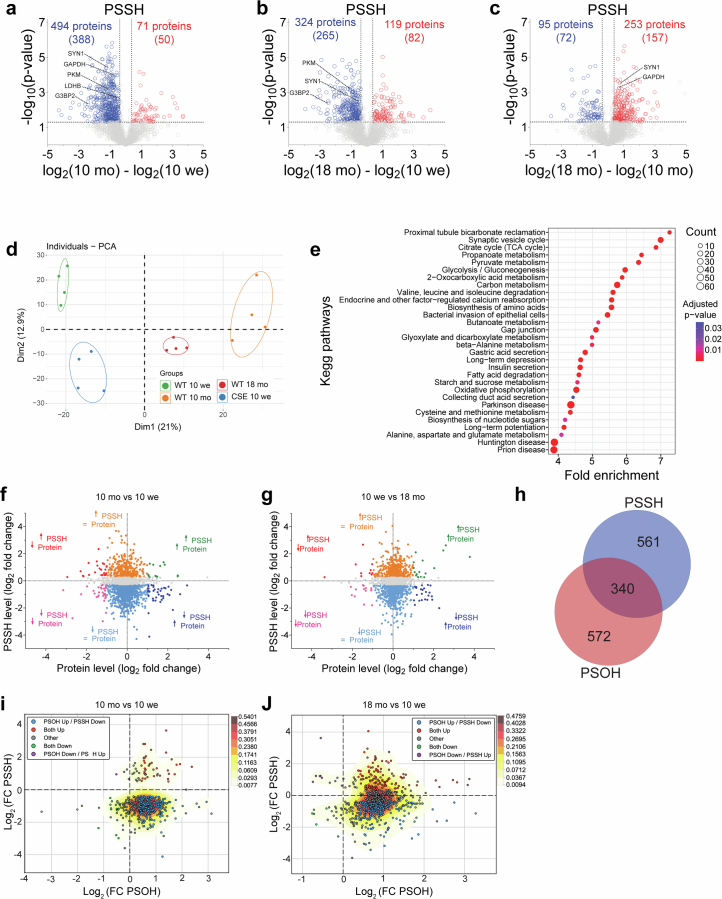
Quantitative persulfidomics analysis of aging brain. (**a-c**) Volcano plot analysis of persulfidome changes in (**a**) 10-month-old mice and (**b**) 18-month-old mice relative to 10-week-old mice and (**c**) 18-month-old mice relative to 10-month-old mice (n = 4, Welch’s t-test). The number of proteins with significantly altered PSSH levels is indicated, with the number in parentheses reflecting proteins identified through a minimum of 2 unique peptides. (**d**) Principal component analysis (PCA) underscoring the reproducibility of persulfidome measurements across all replicates. (**e**) KEGG pathway enrichment analysis of proteins showing significant PSSH changes with age (n = 4 animals, Welsch’s t-test with FDR adjusted p value ≤ 0.05, fold change > 30 %). The graph shows the top 30 significant (Benjamini adjusted p-value ≤ 0.05) and most enriched pathways, with the color gradient indicating adjusted p-values and circle size the number of proteins. (**f-g**) Scatter plot analysis of PSSH and protein expression relationships. log2 fold changes within the persulfidome (y-axis) were plotted against total proteome changes (x-axis) for the (**f**) 10-month-old vs. 10-week-old mice and (**g**) 18-month-old vs. 10-week-old mice. (**h**) Venn diagram comparing sulfenylated and persulfidated proteins found to significantly change with aging. (**i-j**) Log_2_ fold change correlation of proteins significantly changing (Welch´s test, FDR adjusted p-value) and passing 30 % threshold in at least one of the two modifications in (**i**) WT 10 mo vs WT 10 we and (**j**) WT 18 mo vs 10 we. A two-dimensional kernel density estimate (KDE) was used to estimate and represent overall distribution of protein changes. KDE is represented by colour gradient on the scatterplot.

By contrast, PSSH levels predominantly decreased with age, albeit nonlinearly (Fig. 1i, Extended Data Fig. 3a–c and Supplementary Table 3). We identified nearly 2,000 endogenously persulfidated proteins, double the number previously reported using alternative labeling methods^5,22,23^ (Fig. 1j). Although dimedone-based probes can exhibit minor side reactions with highly reactive amino groups^19^, the dimedone-switch method for persulfide labeling included a blocking step with 4-chloro-7-nitrobenzofurazan (NBF-Cl)^4^, which eliminates this possibility. In addition, a precleaning step with neutravidin beads ensured removal of endogenously biotinylated proteins, leaving only those selectively labeled for PSSH^4^ (Fig. 1b).

Similar to sulfenylated proteins, persulfidated proteins were distributed across most cellular compartments (Fig. 1c) and were involved in diverse processes, including metabolism; protein translation, localization and transport; response to stimuli; signal transduction; and neuronal development and organization (Fig. 1k and Extended Data Fig. 3e). PCA of persulfidomes showed clear separation between age groups (Extended Data Fig. 3d). Approximately 40% of persulfidated proteins showed significant changes at one age point or more (Fig. 1l). Unlike the linear or plateauing increase observed for sulfenylation (Fig. 1g), the pattern for persulfidation was more complex, with mid-aged mice showing the most consistent decline (~80% across 5 of 6 clusters; Fig. 1l). However, some proteins exhibited partial recovery in PSSH levels by 18 months (clusters 3–5; Fig. 1l). The pathways particularly enriched in those clusters controlled circadian rhythmicity and autophagy, as well as various neurodegenerative diseases (cluster 4). This mid-life decline was consistent with recent reports of nonlinear dynamics of multiomics profiles in human aging^24^.

These observations were also consistent with the cellular distribution of sulfide-producing enzymes: CSE is localized to neurons, cystathionine beta synthase (CBS) to glial cells, and 3-mercaptopyruvate sulfurtransferase (MPST) to both neurons and astrocytes^4^. Thus, the observed changes in persulfidation reflect cumulative effects across cell types. However, the loss of CSE-regulated persulfidation has profound implications for aging and neurodegeneration, as its depletion shifts the balance toward neurodegenerative events^25^.

Proteins showing age-related PSSH decline were particularly enriched in components of ribosomes, insulin secretion, synaptic vesicle cycle and neurodegenerative diseases, whereas proteins showing decline and partial recovery were associated with ferroptosis, cysteine and methionine metabolism, oxidative phosphorylation and autophagy (Fig. 1m). Notably, changes in the total proteome did not significantly affect the observed age-related decline in PSSH (Extended Data Fig. 3f,g). There was an overlap of ~37% between proteins found to show changes in PSOH and PSSH with aging (Extended Data Fig. 3h), with the majority of these protein showing a decline in PSSH and an increase in PSOH (Extended Data Fig. 3i,j), in agreement with reduced transsulfuration pathway activity and increased reactive oxygen species (ROS) generation observed in aging. To make this complex data analysis easily accessible, we generated an interactive platform that allows comparison across different PTMs and ages and integrates protein search with pathway visualization (https://www.agingredox.org/).

### CSE is a key regulator of the PSOH to PSSH switch in the aging brain

Recent studies have highlighted the critical role of CSE-derived H_2_S and subsequent protein persulfidation in lifespan-extending interventions such as dietary restriction^4,5,26^. CSE has been identified as a prolongevity gene in studies using lifespan-extending interventions in mice^6,7^. In the brain, reduced CSE expression is associated with neurodegenerative conditions, including Alzheimer’s disease and spinocerebellar ataxia^3,8,10^, and mice lacking CSE exhibit a Huntington’ disease-like phenotype^9,27^.

Further analysis of aging mouse brain metabolome^28^ and transcriptome^29^ datasets has confirmed that *Cth* expression decreases in the cerebral cortex of aged mice, leading to accumulation of its substrates, such as homocysteine and cysteine (Supplementary Figs. 2a and 3). Similarly, a significant age-related decline in *CTH* mRNA expression (both isoforms 1 and 2) has been observed in the human frontal cortex of individuals of both sexes^30^ (Supplementary Fig. 2b,c). By contrast, we detected no significant age-related changes in expression of *Mpst* or *Cbs* in aging mice (Supplementary Fig. 2a). However, we did observe notable increases in their expression in the aging human brain (Supplementary Fig. 2d).

**Fig. 2:**
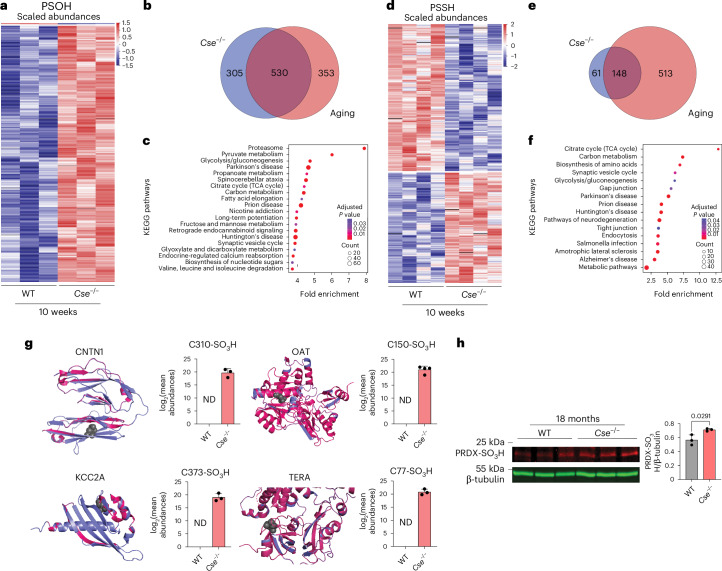
*Cse*^−/−^ mice exhibit higher PSOH and PSO_3_H levels but lower PSSH. **a**, Heatmaps of significant PSOH changes found in *Cse*^−/−^ 10-week-old mice compared to WT 10-week-old mice (*n* = 3 animals; Welch’s *t*-test with FDR-adjusted *P* ≤ 0.05, fold change > 30%). The control 10-week-old mice were the same as in Fig. 1. **b**, Venn diagram comparison of sulfenylated targets found to be significantly affected by age and *Cse* knockout. **c**, KEGG pathway enrichment analysis of proteins showing significant PSOH changes in *Cse*^−/−^(*n* = 3 animals; Welch’s *t*-test with FDR-adjusted *P* ≤ 0.05, fold change > 30%). Benjamini-adjusted *P* ≤ 0.05; color gradient indicates adjusted *P* values, and circle size indicates the number of proteins. **d**, Heatmaps of significant PSSH changes found in *Cse*^−/−^ 10-week-old mice compared to WT 10-week-old mice (*n* = 4 animals; Welch’s *t*-test with FDR-adjusted *P* ≤ 0.05, fold change > 30%). **e**, Venn diagram comparison of persulfidated targets found to be significantly affected by age and *Cse* knockout. **f**, KEGG pathway enrichment analysis of proteins showing significant PSSH changes in *Cse*^−/−^ (*n* = 3 animals; Welch’s *t*-test with FDR-adjusted *P* ≤ 0.05, fold change > 30%). Benjamini-adjusted *P* ≤ 0.05; color gradient indicates adjusted *P* values and circle size the number of proteins. **g**, Prediction of structural changes caused by cysteine sulfonylation found exclusively in *Cse*^−/−^ mice. Modeling was performed using Vienna-PTM. The original crystal structure is depicted in pink and the predicted model in blue. Contactin 1 (CNTN1; PDB 3S97), ornithine aminotransferase (OAT; PDB 2OAT); calcium/calmodulin-dependent protein kinase type II subunit alpha (KCC2A; PDB 1HKX); transitional endoplasmic reticulum ATPase (TERA; PDB 1R7R). Data are presented as mean ± s.d. (*n* = 4). **h**, PRDX sulfonylation levels in 18-month-old WT and *Cse*^−/−^ mice. Intensities were normalized to β-tubulin expression levels. Data are presented as the mean ± s.d. (Student’s *t*-test, *n* = 3 animals). Source data

**Extended Data Fig. 4:**
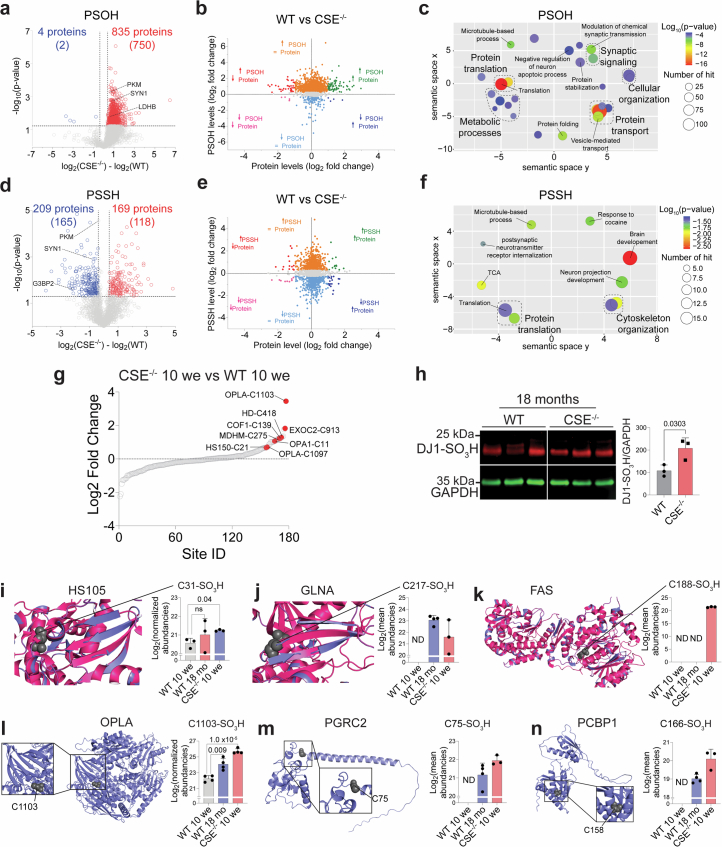
The effect of CSE on persulfidation, sulfenylation and sulfonylation of proteins. (**a**) Volcano plot analysis of PSOH changes in 10-month-old *Cse*^−/−^ mice vs. 10-month-old wild type mice (n = 4 animals, Welch’s t-test, two-sided). (**b**) Scatter plot analysis of PSOH and protein expression relationships. log2 fold changes within the persulfidome (y-axis) were plotted against total proteome changes (x-axis) for the 10-month-old *Cse*^−/−^ and wild type mice. (**c**) GO (Biological process) term enrichment analysis of proteins showing PSOH increase (Welch’s t-test) in *Cse*^−/−^ mice. Enrichment was performed using DAVID and the results visualized through REVIGO. Benjamini adjusted p-value ≤ 0.05. **d**) Volcano plot analysis of PSSH changes in 10-month-old *Cse*^−/−^ mice vs. 10-month-old wild type mice (n = 4, Welch’s t-test). **e**) Scatter plot characterizing log2 fold changes within the persulfidome (y-axis) and protein expression (x-axis) for comparison between 10-month-old *Cse*^−/−^ and wild type mice. **f**) GO (Biological process) term enrichment analysis of proteins showing significant PSSH decrease (Welch’s t-tes, two-sided) in *Cse*^−/−^ mice (Benjamini adjusted p-value ≤ 0.05). **g**) The proteome-wide and site-specific changes in protein sulfonylation in 10-wek old *Cse*^−/−^ vs their wild type littermates (n = 4, Welch’s t-test). **h)** DJ1 (PARK7) sulfonylation levels in 18-month-old WT and *Cse*^−/−^ mice. The intensities were normalized to the GAPDH expression levels. Data are presented as mean ± SD (Student’s t-test, n = 3 animals). (**i-n**) Prediction of the structural changes caused by cysteine sulfonylation that is observed in *Cse*^−/−^ mice. Modeling was performed using VIENNA-PTM. Data are presented as mean ± SD (**i**) Heat shock protein 105 kDa (HS105) is based on the 6GFA.pdb model (Student’s t-test, compared to WT 10 weeks control, n = 4 animals). (**j**) Glutamine synthetase (GLNA) is based on the 2OJW.pdb model. (**k**) Fatty acid synthase (FAS) is based on 5MY2.pdb model. (**l**) 5-oxoprolinase (OPLA) is based on the AF-O14841-F1-model_v4.pdb model (Student’s t-test, compared to WT 10 weeks control, n = 4 animals). (**m**) Membrane-associated progesterone receptor component 2 (PGRC2) based on AF-Q80UU9-F1- model_v4.pdb model. (**n**) Poly(rC)-binding protein 1 (PCBP1) is based on the AF-Q15365-F1- model_v4.pdb model. Source data

To investigate how CSE-derived H_2_S modulates thiol remodeling at the proteome level, we compared PSOH and PSSH levels between wild-type (WT) and *Cse*-knockout (*Cse*^−/−^) mice (Supplementary Table 4). Significantly higher PSOH levels were observed in the brains of 10-week-old *Cse*^−/−^ mice compared to their WT littermates (Fig. 2a and Extended Data Fig. 4a,b). Notably, approximately 200 proteins exhibited higher PSOH levels in brains from 10-week-old *Cse*^−/−^ mice than in those from their 18-month-old WT littermates, suggesting that CSE deficiency results in elevated steady-state oxidative damage (Supplementary Table 4), despite increased levels of some antioxidant enzymes (Extended Data Fig. 2).

A substantial overlap was observed between proteins with elevated PSOH levels in *Cse*^−/−^ mice and those with increased PSOH levels in aging WT littermates (Fig. 2b). Pathways critical for healthy neuronal function were particularly enriched among these proteins (Fig. 2c and Extended Data Fig. 4c). Conversely, a marked reduction in PSSH levels was observed in *Cse*^−/−^ mice (Fig. 2d and Extended Data Fig. 4d,e); approximately 70% of proteins showing decreased PSSH in *Cse*^−/−^ mice exhibited similar declines in aging mice (Fig. 2e), and pathway enrichment analyses revealed patterns consistent with those observed in aging (Fig. 2f and Extended Data Fig. 4f). These findings indicate that CSE has a key role in regulation of the PSOH to PSSH switch in the aging brain and is therefore important in maintenance of redox homeostasis and neuronal health.

### Age-induced PSSH to PSO_3_H transition is more profound in *Cse*^*−/−*^ mice

Consistent with the reaction scheme depicted in Fig. 1a, both aging and *Cse*^−/−^ mice were expected to exhibit increased thiol oxidation, specifically sulfonylation (PSO_3_H). Owing to the lack of established methods for selective labeling and enrichment of PSO_3_H, we analyzed proteome data obtained by lysing tissues with NBF-Cl; this blocks all thiols, including sulfenic acids (Fig. 1b), preventing unwanted cysteine oxidation during sample preparation. Of 645 detected sulfonylated peptides (632 cysteine sites), 382 (388 cysteine sites) were absent from all 10-week-old WT mice, indicating that both aging and CSE deficiency are associated with increased PSO_3_H levels (Supplementary Table 5). We quantified changes in more than 200 peptides that were present in at least three of four measured samples (Extended Data Fig. 4g and Supplementary Table 5). Among these, 179 cysteine sites were common to all samples, 27 were uniquely identified in 18-month-old mice and 30 were unique to *Cse*^−/−^ mice (Supplementary Table 5). These observations were limited by the stochastic nature of data-dependent analysis.

To assess the structural impact of sulfonylation, we selected proteins with known crystal structures that showed significant increases in sulfonylation in aging and/or *Cse*^−/−^ mice. Using Vienna-PTM 2.0, a resource for exploration of PTMs based on molecular dynamics simulations^31^, we predicted the effects of PSO_3_H on these protein structures. Overlaying the original crystal structures with those modeled for PSO_3_H revealed conformational changes in all analyzed proteins (Fig. 2g and Extended Data Fig. 4i–n). In the modeled proteins, sulfonylated cysteines were predominantly located in β-sheet domains or loops preceding β-strands. Immunoblotting for well-defined sulfonylated proteins PRDX^32^ (Fig. 2h) and DJ-1 (ref. ^33^; Extended Data Fig. 4h) further confirmed the critical role of endogenously produced H_2_S in regulation of PSO_3_H levels.

### Age-induced cysteine PTMs contribute to the LLPS of synapsin 1

**Fig. 3:**
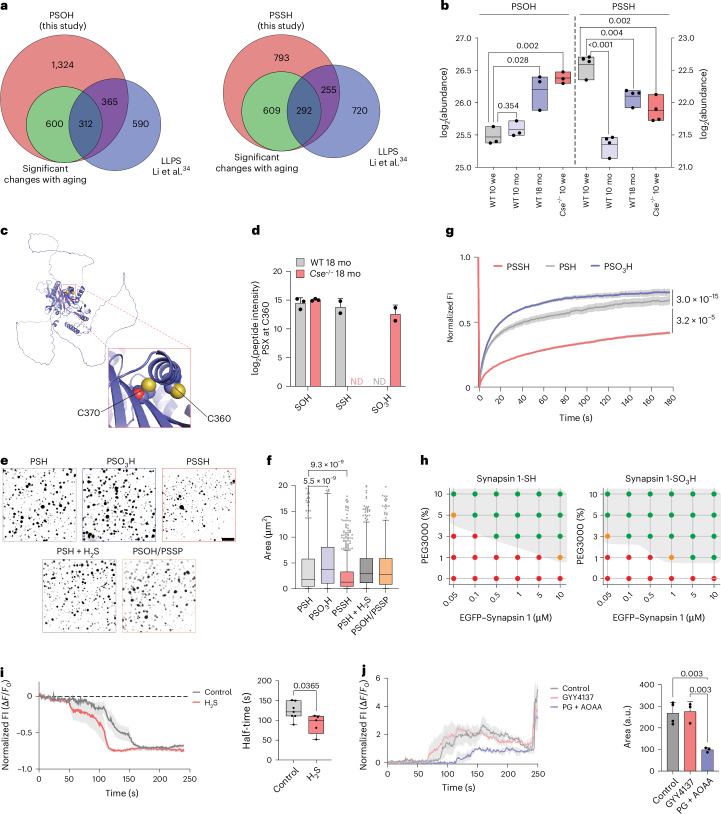
Age-related thiol remodeling of synapsin 1 controls its LLPS and neurotransmitter release. **a**, Venn diagram representing the overlap of sulfenylated (left) and persulfidated (right) proteins identified in our study (all proteins are shown by red circles; those that significantly changed with aging are shown in green) with proteins found to undergo biomolecular condensation by Li et al.^34^. **b**, Age-induced sulfenylation (PSOH) and persulfidation (PSSH) of synapsin 1. Box plots show mean (center line) and minimum and maximum values (box bounds) (PSOH: *n* = 3 animals, PSSH: *n* = 4 animals, Welch’s *t*-test from proteomic analysis). **c**, Structure of synapsin 1, with two of its cysteines (C360 and C370) highlighted. **d**, Changes in sulfenylation (SOH), persulfidation (SSH) and sulfonylation (SO_3_H) of C360 but not C370 were observed in WT and *Cse*^−/−^ 18-month-old mice. Data are presented as the mean ± s.d. (*n* = 3 animals). **e**, Representative microscopy images for EGFP–synapsin 1 condensates (10 µM in the presence of 3% PEG3000) formed under different cysteine modifications. Scale bar, 20 µm. **f**, Distribution of the droplet area in µm^2^ among the different cysteine modifications. Box plots show median (center line), 25th to 75th percentiles (box bounds) and 1.5× interquartile range (whiskers) (*n* = 496 (PSH), *n* = 364 (PSO_3_H), *n* = 840 (PSSH), *n* = 505 (PSH + H_2_S), *n* = 437 (PSOH/PSSP) condensates over 2 independent reconstitutions, one-way analysis of variance (ANOVA), Tukey’s HSD test for multiple comparisons). **g**, FRAP quantification of EGFP–synapsin 1. The line indicates the mean and gray shading the standard error (*n* = 50 (PSH), *n* = 34 (PSO_3_H), *n* = 46 (PSSH) condensates over at least 2 independent reconstitutions; two-way ANOVA, Sidak’s post hoc test). **h**, Summary of the phase separation behavior of the reduced and sulfonylated EGFP–synapsin 1 with increased concentration of PEG3000. **i**, Kinetics of mScarlet–synapsin 1 droplet dissolution in the synapses of primary neurons, treated with 100 µM Na_2_S or untreated, upon stimulation with 92.5 mM KCl. Data are presented as mean values (line) ± s.d. (gray band); box plots show the median (center line), 25th to 75th percentiles (box bounds), and minimum and maximum values (whiskers) (*n* = 3 animals, a few preparations per animal; Student’s *t*-test). **j**, Neurotransmitter release from mouse primary neurons with and without pretreatment with 100 µM GYY4137 or a 1-mM mixture of propargylglycine (PG) and aminooxyacetic acid (AOAA) for 1 h. Synaptic vesicle release was triggered by perfusion of neurons with 92.5 mM KCl; the fluorescence of synaptophysin–pHluorin was used as a readout. Means are shown as lines and s.e.m. as a gray band. The area under the curve was calculated for each measurement and presented as the mean (line) ± s.d. (*n* = 3 animals for GYY4137 and PG + AOAA conditions, *n* = 4 animals for control condition, a few preparations per animal; one-way ANOVA, Tukey’s HSD test for multiple comparisons). FI, fluorescence intensity; ND, not determined. Source data

**Extended Data Fig. 5:**
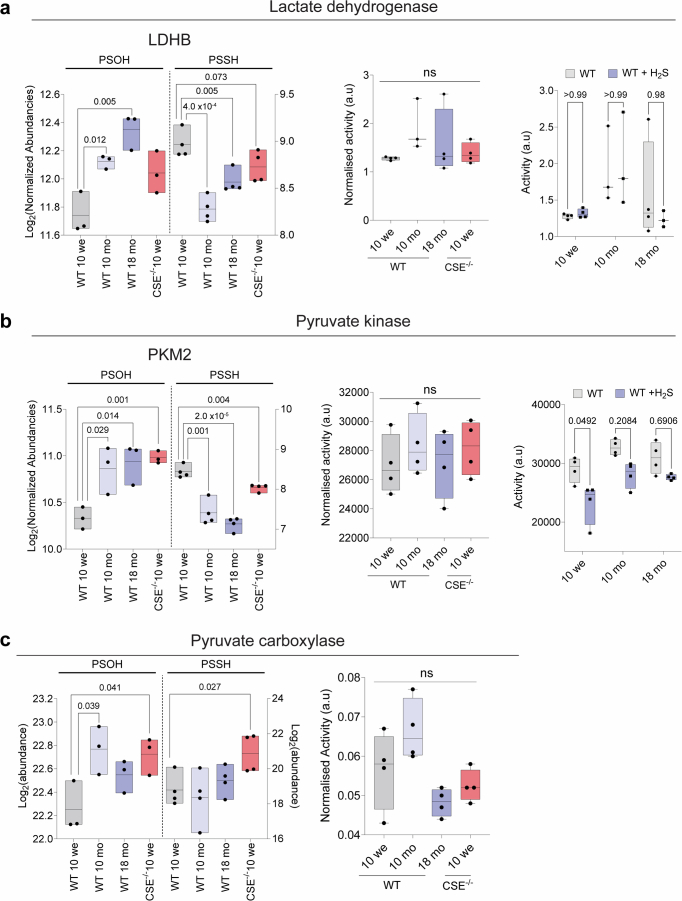
Age-induced thiol remodeling predominantly affects proteins known to undergo LLPS. **a-c**) Age-induced sulfenylation and persulfidation changes of metabolic enzymes show no impact on their activity. Left panel: Box plots show mean (centre line), minimum and maximum values (box bounds) (PSOH: n = 3 animals, PSSH: n = 4 animal). Welch test and FDR adjusted p-values from the proteomic analysis. Activity assays: Box plots show median (centre line), 25th–75th percentiles (box bounds), and minimum to maximum values (whiskers), (two-way ANOVA, Tukey’s HSD Test for multiple comparisons, n = 4).(**a**) Lactate dehydrogenase B (LDHB), (**b**) pyruvate kinase (PKM) and (**c**) pyruvate carboxylase, all key metabolic enzymes, show significant changes of sulfenylation and persulfidation with aging and/or *Cse*^−/−^ (left, Welch’s t-test) but no significant change could be observed in their activity (middle, 1-way Anova, n.s. non-significant) even after addition of 10 μM H_2_S to a freshly prepared brain lysate (right, paired two-way Anova). Source data

To elucidate the mechanisms underlying our observations, we conducted a detailed analysis of the proteomic data. This revealed two major classes of proteins that were particularly susceptible to age-related thiol remodeling: (1) metabolic enzymes; and (2) structural and signaling proteins involved in neuronal structure, synapse organization, vesicle-mediated transport and regulation of neurotransmission. Traditional models of PTM-mediated signaling propose that modifications result in either a gain or a loss of protein function. We selected lactate dehydrogenase (LDHB), pyruvate kinase (PKM) and pyruvate carboxylase, which all exhibited age-dependent changes in PSOH and PSSH levels, and assessed their enzymatic activities in brain lysates. No significant changes in enzyme activity were observed across different ages or in *Cse*^−/−^ mice, even after exogenous H_2_S supplementation (Extended Data Fig. 5). However, we noted that >30% of proteins whose thiol redox status changed with aging overlapped with proteins reported to undergo LLPS^34^ (Fig. 3a).

**Extended Data Fig. 6:**
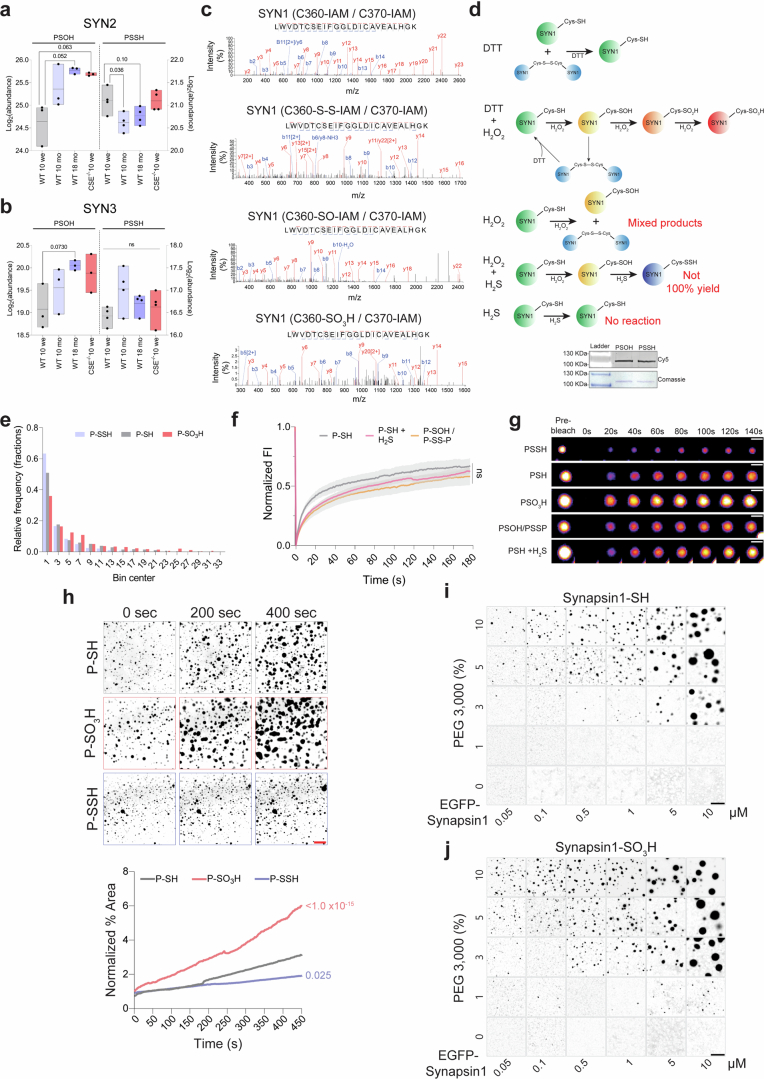
Age-induced changes in synapsin’s thiol oxidation affect its LLPS properties. (**a-b**) Boxplot of the PSOH and PSSH remodelling of (**a**) synapsin 2 and (**b**) synapsin 3 in aging wild-type and *Cse*^−/−^ mouse brain. Box plots show mean (centre line), minimum and maximum values (box bounds) (PSOH: n = 3 animals, PSSH: n = 4 animal). Welch test and FDR adjusted p-values from the proteomic analysis. **c**) Annotated TimsTOF CID MS/MS spectra of synapsin 1 peptides highlighting distinct oxidation states of cysteine 360. Spectra originate from quantified peptides extracted from WT and *Cse*^−/−^ 18-month-old mice, as depicted in Fig. 3c. The y-ions are denoted in red, while the b-ions are indicated in blue. (**d**) Schematic representation of the protocols for preparation of different cysteine modifications, used for the phase separation and FRAP experiments. PSH (100 µM DTT), PSSH (100 µM H_2_O_2_ + 500 µM H_2_S), PSO_3_H (100 µM DTT + 200 µM H_2_O_2_), and PSOH/PSSP (200 µM H_2_O_2_). DTT reduced EFGP-synapsin 1 was also treated with 500 µM H_2_S as a negative control. Inset: In-gel detection of persulfidated and sulfenylated EFGP-synapsin 1, prepared by the protocols depicted in the scheme. Daz-2 and BTD, clicked to Cy5-alkyne or azide were used to visualize PSSH and PSOH, respectively. Coomassie blue staining was used to show the protein load. (**e**) Relative frequency histogram of EFGP-synapsin 1 droplets in relation to their size. (**f**) Quantification of fluorescence recovery after photobleaching. Prior to initiating phase separation, 10 µM EFGP-synapsin 1 samples underwent treatment with various cysteine-modifying agents as depicted in (**d**) Data are presented as mean (line) ± SEM (grey band). Subsequent phase separation was induced by 3% PEG3000 (n = 50 (P-SH), 39 (P-SH + H_2_S), 29 (P-SOH / P-SS-P) condensates over at least 2 individual reconstitutions, two-way ANOVA, Sidak posthoc test, n.s. non-significant). (**g**) Representative microscopy images capturing EGFP-synapsin 1 condensates before and after photobleaching. Scale bar = 2 µm. (**h**) Wetting of EGFP-synapsin 1 condensates (3 individual reconstitutions for PSH and PSSH, and 2 for PSO_3_H), two-way ANOVA, Sidak posthoc test). Scale bar = 20 µm. (**i-j**) Phase diagram of EGFP-synapsin 1 LLPS. Representative images of the LLPS phase diagram formed with (**i**) the reduced or (**j**) sulfonylated EGFP-synapsin 1. The phase diagram was built using increased concentrations of the protein PEG3000. Performed in 2 independent reconstitutions. Scale bar = 5 µm (applies to all panels). Source data

This prompted us to hypothesize that age-induced thiol oxidation remodeling might influence the mesoscale organization of proteins in neurons, thereby regulating their function. Notably, synapsin 1, the most abundant phosphoprotein in nerve terminals and a known phase-separating protein^35^, exhibited an age-dependent increase in PSOH and a decrease in PSSH (Fig. 3b). These changes were as pronounced in 10-week-old *Cse*^−/−^ mice as in their 18-month-old WT littermates (Fig. 3b). Similar age-related effects were observed for other synapsin isoforms (Extended Data Fig. 6a,b).

Synapsin 1 contains three cysteine residues (Fig. 3c). We found that cysteine 360 (C360) existed in all three oxidation states, PSOH, PSSH and PSO_3_H, with the latter detected only in aged *Cse*^−/−^ mice (Fig. 3d and Extended Data Fig. 6c). We estimated that ~3% of cysteine residues were sulfonylated in aged Cse^−/−^ mice. C360 is located within the highly structured ATP-binding domain of synapsin 1, which is responsible for oligomerization of the protein (Fig. 3c). Although PTMs in this region were not expected to directly affect phase separation, a process primarily governed by the intrinsically disordered regions of synapsin 1^35–37^, we designed a protocol to prepare different redox forms of synapsin 1 (Extended Data Fig. 6d) and compared their phase separation behavior. We used H_2_O_2_ treatment of fully reduced synapsin 1 as a method to generate PSOH (and/or PSSP), mimicking intracellular thiol oxidation^38,39^. As in numerous previous studies, cotreatment with H_2_O_2_ and H_2_S was used to transiently generate PSSH^4,16,40,41^, again mimicking the intracellular mechanism for PSSH generation. PSO_3_H was generated by addition of H_2_O_2_ to protein solutions containing small amounts of dithiothreitol (DTT). The role of DTT was to prevent disulfide formation and drive cysteine hyperoxidation. As this strategy can also lead to formation of PSO_2_H, we refer to the product as PSO_*x*_H (where *x* = 2 or 3) (Extended Data Fig. 6d). H_2_S was added to fully reduced protein to produce a negative control for PSSH, whereas treatment of iodoacetamide (IAM)-blocked protein thiols with H_2_O_2_ served as a control for methionine oxidation. In our experimental design, we aimed to mimic the continual formation and interconversion of these oxidative and persulfidated states, analogous to the dynamic conditions in cells, in which fluxes of H_2_O_2_ and/or H_2_S continuously reshape the redox landscape (Supplementary Text).

When incubated with a crowding agent (3% PEG3000), all redox forms of synapsin 1 readily formed condensates (Fig. 3e). However, synapsin 1 PSO_*x*_H formed significantly larger droplets than the fully reduced protein, whereas PSSH resulted in smaller droplets (Fig. 3e,f and Extended Data Fig. 6e). These condensates exhibited dynamic behavior and recovered promptly after photobleaching but with distinct recovery kinetics (Fig. 3g and Extended Data Fig. 6f). The recovery rate and percentage increased for synapsin 1 PSO_*x*_H (11.6 s versus 14.5 s; 15% increase in recovery) but decreased for PSSH (29.9 s versus 14.5 s; 35% decrease in recovery) (Fig. 3g and Extended Data Fig. 6g). From the half-time (*t*_1/2_), we estimated the apparent diffusion coefficients of the protein molecules to be 6.2 × 10^−4^ μm^2^ s^−1^, 7.7 × 10^−4^ μm^2^ s^−1^ and 3.0 ×10^−4^ μm^2^ s^−1^ for PSH, PSO_3_H and PSSH, respectively.

Droplets formed by synapsin 1 treated with H_2_O_2_ (to generate a PSOH/disulfide mix) or H_2_S (expected to be nonreactive) were indistinguishable in terms of size and fluorescence recovery after photobleaching (FRAP) recovery kinetics from those formed by fully reduced synapsin 1 (Fig. 3e,f and Extended Data Fig. 6f). The lack of effect from H_2_O_2_ treatment alone also excluded the possibility that methionine oxidation influenced LLPS, as suggested for ataxin^42^. This was further confirmed by the absence of differences in FRAP between IAM-blocked synapsin treated or not treated with H_2_O_2_ (Supplementary Fig. 4). Observations of droplet wetting revealed clear differences in liquid properties: PSO_*x*_H promoted faster and more extensive wetting compared to PSH, whereas persulfidated synapsin 1 exhibited minimal wetting, with droplets remaining small (Extended Data Fig. 6h). These findings demonstrate that the cysteine redox status of synapsin 1 modulates its phase separation propensity, with sulfonylation enhancing LLPS. Indeed, the phase diagram of synapsin 1 LLPS confirmed that sulfonylated proteins phase separated at lower concentrations of crowding agent and protein (Fig. 3h and Extended Data Fig. 6i,j). Synapsin 1 is present at approximately 100 µM in nerve terminals^35^. Given the phase-diagram shift we observed for PSO_3_H, sulfonylation of about 3% of the protein population would be sufficient to drive the onset of phase separation.

The smaller condensates, slower diffusion and wetting observed for persulfidated synapsin 1 suggest that PSSH restricts phase separation capability. This implies that H_2_S-induced PSSH formation could significantly affect synaptic vesicle dispersion and neurotransmitter release. Consistent with this, exposure of primary neurons to H_2_S accelerated KCl-induced dispersion of synapsin 1 from presynaptic terminal in primary hippocampal neurons (Fig. 3i). Similarly, treatment of primary mouse neurons with slow-releasing H_2_S donor GYY4137 enhanced synapsin 1 dispersion and neurotransmitter release, whereas CSE inhibition delayed release by approximately twofold and reduced overall synaptic vesicle release (Fig. 3j and Supplementary Video 1). These results provide a mechanistic explanation for the previously proposed role of endogenous H_2_S in neurotransmitter release^43–45^, particularly in the context of neurodegenerative diseases associated with reduced CSE expression levels and impaired neurotransmitter release^3,8–10^.

### C73 in G3BP2 is a regulator of SG formation

**Fig. 4:**
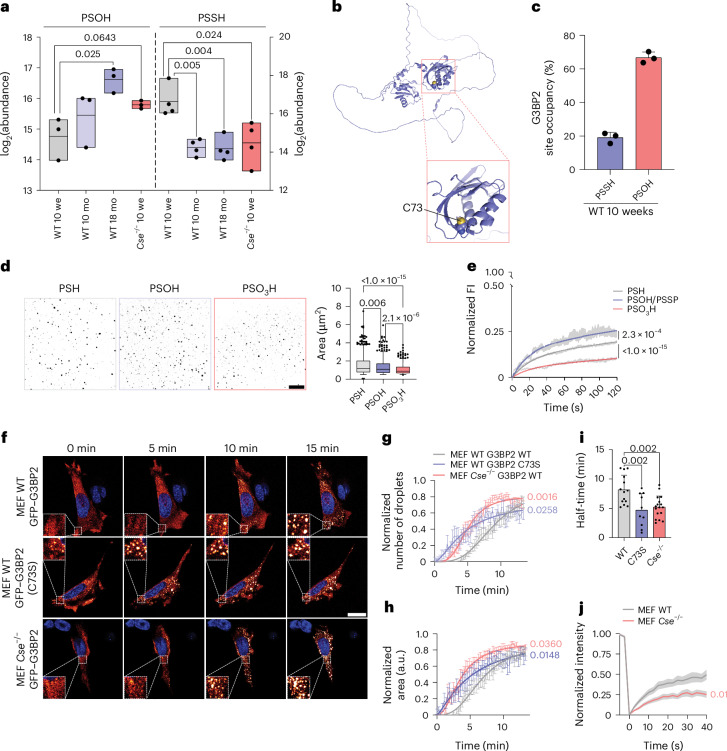
Thiol modifications of G3BP2 affect its biomolecular condensation. **a**, Age-induced sulfenylation (PSOH) and persulfidation (PSSH) of G3BP2. Box plots show mean (center line) and minimum and maximum values (box bounds) (PSOH: *n* = 3 animals, PSSH: *n* = 4 animals, Welch’s *t*-test from proteomic analysis). **b**, G3BP2 AlphaFold-predicted structure (AF-P97379-F1-model_v4.pdb). Cysteine 73 was unique and present in the NTF2-like domain of the protein. **c**, G3BP2 site occupancy of PSSH and PSOH in brain samples from 10-week-old WT animals. Site occupancy was obtained using the protocol depicted in Extended Data Fig. 7h. Data are presented as the mean ± s.d. (*n* = 3). **d**, Phase separation of G3BP2 with different cysteine PTMs. Before phase separation induction, G3BP2 (10 μM) was treated with DTT (100 μM; PSH), H_2_O_2_ (200 μM; PSOH) or a combination of DTT and H_2_O_2_ (100 μM and 200 μM, respectively; PSO_*x*_H) for 30 min at 37 °C. LLPS was induced using 50 ng μl^−1^ of total RNA extract, and images were taken 5 min after induction. Quantification of the droplet area is presented as a box plot with median (center line), 25th to 75th percentiles (box bounds) and 1.5× interquartile range (whiskers), (*n* = 599 (PSH), *n* = 400 (PSO_3_H), *n* = 463 (PSOH/PSSP) condensates over 3 individual reconstitutions; one-way ANOVA, Tukey’s HSD test for multiple comparisons. Scale bar, 20 μm. **e**, FRAP kinetics of G3BP2-Cy3 condensates formed with reduced (PSH), sulfenylated (PSOH) and sulfonylated (PSO_3_H) G3BP2. Colored lines represent the mean of all kinetic fits, and 95% confidence interval errors are represented as a gray band (*n* = 63 (PSH), *n* = 74 (PSO_3_H), *n* = 56 (PSOH/PSSP) condensates over 3 individual reconstitutions; two-way ANOVA, Sidak’s post hoc test). **f**, Arsenite (500 μM) stress-induced SG formation in WT MEFs expressing either WT or the C73S mutant of GFP–G3BP2 or in *Cse*^−/−^ MEFs expressing the WT GFP–G3BP2 (representative confocal images of the real-time recording, *n* = 5 biological replicates). Scale bar, 20 μm. **g**,**h**, Quantification of number (**g**) and size (**h**) of G3BP2 SGs following arsenite treatment. Mean and nonfits are represented by a continuous line, and bars represent the standard error (*n* = 18 (MEFs WT–G3BP2 WT), *n* = 11 (MEFs WT–G3BP2 C73S) and *n* = 15 (MEFs *Cse*^−/−^–G3BP2 WT) cells from at least 5 biological replicates; two-way ANOVA; *P* value indicates comparison to the WT MEFs expressing G3BP2 WT (control)). **i**, *t*_1/2_ values for G3BP2 SG formation calculated from nonlinear regression. Data are presented as mean ± s.d. with individual values (*n* = 18 (MEFs WT–G3BP2 WT), *n* = 11 (MEFs WT–G3BP2 C73S) and *n* = 15 (MEFs *Cse*^−/−^–G3BP2 WT) cells from at least 5 biological replicates; one-way ANOVA, Tukey’s HSD test for multiple comparisons). **j**, In cellulo FRAP kinetics of GFP–G3BP2 in WT or *Cse*^−/−^ MEFs. Standard error is represented as a gray band (*n* = 10 cells from at least 5 biological replicates, with average of 1–2 condensate(s) per cell; two-way ANOVA, Sidak’s post hoc test). Source data

**Extended Data Fig. 7:**
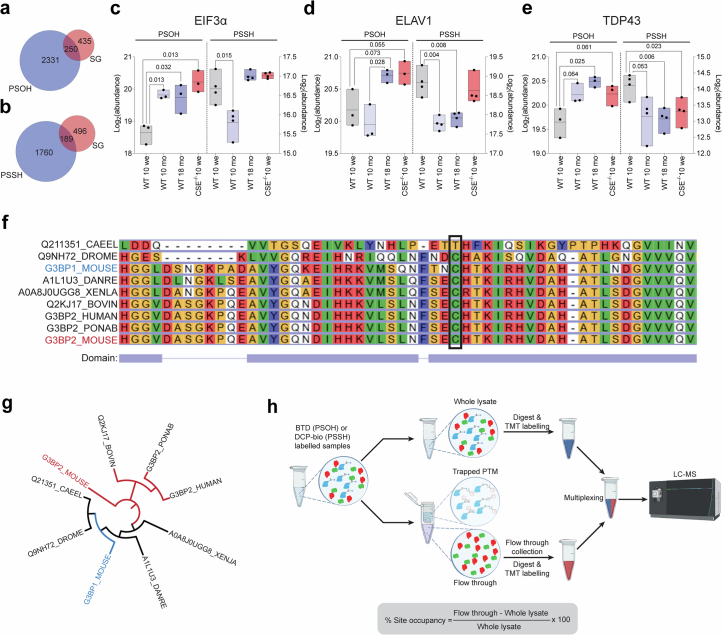
Age-induced thiol remodelling affects stress granule proteins. (**a-b**) Venn diagram representing the overlap of proteins present in stress granules (SG) and those identified in (**a**) PSOH and (**b**) PSSH data sets. (**c-e**) Boxplot of the age-induced PSOH and PSSH changes of (**c**) eIF3α, (**d**) ELAV1 and (**e**) TDP-43. Box plots show mean (centre line), minimum and maximum values (box bounds) (PSOH: n = 3 animals, PSSH: n = 4 animal). Welch test and FDR adjusted p-values from the proteomic analysis. (**f**) Sequence alignment of G3BP2 focused of the cysteine 73 region. The conserved cysteine residue, positioned at C73 in the mouse G3BP2 sequence, is highlighted with a black rectangle. Conservation of this cysteine across species underlines its potentially important role in the protein’s function, such as its phase separation potential. (**g**) Circular phylogenetic cladogram build using G3BP2 sequences alignment from a range of species: *Mus musculus* (MOUSE), *Bos taurus* (BOVIN), *Pongo abelii* (PONAB), *Homo sapiens* (Human*), Xenopus laevis* (XENA), *Danio rerio* (DANRE), Drosophila melanogaster (DROME), and *Caenorhabditis elegans* (CAEEL). For contextual comparison, G3BP1 from *Mus musculus* is also included. (**h**) Schematic representation of the sample preparation to determine PSOH and PSSH site occupancy in 10 week-old WT mice brain. Shortly, lysate labelled for either PSOH (BTD – UV-cleavable probe) or PSSH (DCP-Bio1) were readjusted across replicates. For each replicate, an equal amount of protein was either directly trypsinized for the whole lysate assessment or subjected to incubation with streptavidin beads. Flow through was then collected and trypsinized. Both whole lysate and flow-through were labelled with TMT, pulled together and measured using LC-MS. The site occupancy of G3BP2 was then calculated for each replicates following the defined formula. Figure created in BioRender; Filipovic, M. https://biorender.com/kveknhn (2026). Source data

In addition to the molecular-crowding-induced phase separation seen in synapsin 1, proteins undergo condensate formation when interacting with RNAs^46–51^. Stress granules (SG), a subset of such ribonucleoprotein granules, are initiated by various stressors to arrest translation initiation and protect RNAs^52,53^. Prompted by the observation that the thiol oxidation status of synapsin 1 affected its ability to phase separate, we overlapped the datasets obtained for aging PSSH and PSOH with proteomes of SGs and found that approximately one-third of all proteins reported to be associated with SGs showed significantly lower PSSH levels and significantly higher PSOH levels (Extended Data Fig. 7a,b). These included several well-defined SG proteins (Extended Data Fig. 7c–e), but we focused our analysis on Ras GTPase-activating protein-binding protein 2 (G3BP2), a key initiator of SG formation^48,49^. G3BP2 displayed an increase in PSOH and a similar decrease in PSSH levels, caused by aging or absence of CSE (Fig. 4a).

G3BP2, the predominant paralog expressed in brain^54^, contains a single cysteine residue (C73) located within the structured β-sheet domain (Fig. 4b), which appeared early in evolution (Extended Data Fig. 7f,g). We also designed an experiment to estimate the steady-state occupancy of C73 by PSSH and PSOH, showing that in the frontotemporal regions of the brains of 10-week-old mice, ~65% of C73 exists in the form of PSOH and ~20% as PSSH (Fig. 4c and Extended Data Fig. 7h), suggesting important roles of these modifications in G3BP2 function.

**Extended Data Fig. 8:**
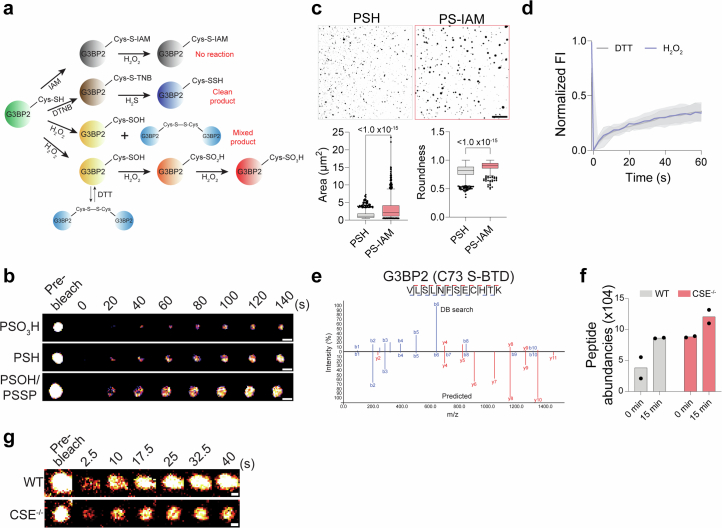
Sulfenylation of C73 modulates G3BP2 phase separation. (**a**) Schematic illustration of G3BP2 treatment used to prepare various cysteine modifications: PS-TNB, PSSH, PSH, and PS-IAM, PSOH and PSO_x=2,3_H. DTNB: 5,5′-dithiobis(2-nitrobenzoic acid). IAM: iodoacetamide. (**b**) Representative microscopy images for G3BP2-Cy3 condensates (10 μM) formed under different cysteine modifications (PSH, PSOH, and PSO_x_H) prepared as shown in (**a**). Related to Fig. 4e, n = 3 independent reconstitutions, at least 56 droplets analysed per conditions. LLPS was induced using 50 ng/μl of total RNA extract. (**c**) Representative images of G3BP2-Cy3 condensates (10 μM) upon 30 min treatment with 20 µM IAM compared to not treated. Quantification of droplet area is shown below. Box plots show median (centre line), 25th–75th percentiles (box bounds), and 1.5× IQR (whiskers) (n = 1109 (PSH) and 1250 (PS-IAM) condensates over 3 independent reconstitutions, Student t-test). Scale bar = 20 μm. (**d**) Representative images and quantification of FRAP assay performed in G3BP2-Cy3 pre-blocked with IAM and treated as for Fig. 4e. Data are presented as mean (line) ± SD (grey band) (n = 23 (DTT) and 54 (H_2_O_2_) condensates, two-way ANOVA, Sidak posthoc test, non-significant). (**e**) Annotated MS/MS spectra of G3BP2 peptides highlighting the BTD trapped cysteine 73 SOH upon arsenite treatment. (**f**) Changes in abundancy of G3BP2 C73-BTD peptide (depicted in (**e**)) in WT and *Cs*e^−/−^ MEFs treated or not with arsenite. (n = 2 biological replicates). Data are presented as mean with individual values as dots. (**g**) Representative images of *in-cellulo* FRAP of GFP-G3BP2 in WT and *Cse*^−/−^ MEFs. Quantification is shown in Fig. 4j. Source data

To address the potential effects of cysteine oxidation on G3BP2 phase separation, we first tested PSOH and PSO_*x*_H forms of the recombinant protein (Extended Data Fig. 8a). Upon addition of RNA, droplet formation was observed in both cases, with G3BP2 PSO_*x*_H forming significantly smaller condensates (Fig. 4d) that showed slower FRAP and a lower percentage of recovery (*t*_1/2_ = 55.0 s versus 44.5 s; 42% decrease) (Fig. 4e and Extended Data Fig. 8b). By contrast, granules formed by G3BP2 PSOH showed faster kinetics and a higher percentage of FRAP (*t*_1/2_ = 37.6 s versus 44.5 s; 25% increase) (Fig. 4e and Extended Data Fig. 8b), suggesting that sulfenylation enhanced the diffusion rate of G3BP2. This effect was not observed in IAM-blocked G3BP2, confirming that it was caused by cysteine modifications rather than methionine oxidation (Extended Data Fig. 8c,d).

As no proper amino acid mimetic exists for PSOH (PSOH is expected to be deprotonated at physiological pH owing to its p*K*_a_ of ~7)^38^, the closest approximation is a cysteine-to-serine mutation. To test whether PSOH influenced SG formation in vivo, we expressed GFP-tagged WT or C73S mutant G3BP2 in mouse embryonic fibroblasts (MEFs) and monitored their response to arsenite-induced stress. All cells expressing C73S-G3BP2 exhibited small preformed SGs even in the absence of stress (Fig. 4f and Supplementary Video 2); these continued to grow in size and number significantly faster than WT granules following arsenite exposure, although the normalized number and size of droplets per cell after 15 min of arsenite stress were comparable between the two conditions (Fig. 4g–i and Supplementary Video 2). These data suggest that the total pool of G3BP2 was unaffected but its biomolecular condensation kinetics were altered.

A similar kinetic profile and behavior were observed in *Cse*^−/−^ MEFs (Fig. 4f–i and Supplementary Video 2), in which BTD trapping followed by immunopulldown and MS analysis revealed that untreated *Cse*^−/−^ MEFs contained levels of PSOH comparable to those of WT cells treated with arsenite (Extended Data Fig. 8e,f). FRAP of G3BP2 SGs was significantly slower in *Cse* ^−/−^ MEFs than in WT cells (Fig. 4k, Extended Data Fig. 8g and Supplementary Video 3). This could be explained by higher levels of protein cysteine hyperoxidation in these cells, as reported previously^4^, consistent with in vitro observations for PSO_*x*_H G3BP2 condensates (Fig. 4e).

### G3BP2 persulfidation leads to dissolution of SGs

**Fig. 5:**
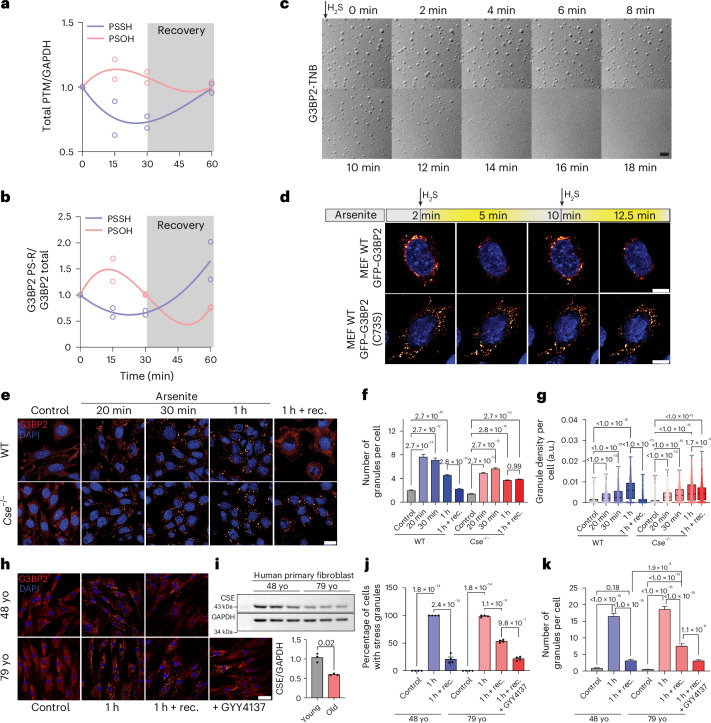
Persulfidation of G3BP2 leads to SG disassembly. **a**,**b**, Global (**a**) and G3BP2 (**b**) PSOH and PSSH changes in MEFs treated with 500 μM NaAsO_2_ for 15 min and 30 min. After 30 min, NaAsO_2_ was removed, and cells were allowed to recover for 30 min. BTD labeling was used for PSOH, and the dimedone-switch method with DCP-Bio1 was used for PSSH (*n* = 2 biological replicates). **c**, Real-time dissolution of G3BP2 condensates initiated by protein persulfidation; 20 µM of G3BP2 was incubated with 40 µM of DTNB and 50 ng µl^−1^ of RNA for 10 min before addition of 50 µM of H_2_S and recording (representative differential interference contrast images of the dissolution of 3 separate reconstitutions are shown). Related to Supplementary Video 4. Scale bar, 10 μm. **d**, Representative images of confocal live cell monitoring of GFP–G3BP2 condensate dissolution upon addition of H_2_S in WT MEFs expressing GFP–G3BP2 WT or GFP–G3BP2 (C73S). Na_2_S (100 μM) was added 15 min after SG formation was initiated with arsenite (500 μM). *n* = 3 biological replicates. Results are related to those shown in Supplementary Video 5 and 6. Scale bar, 10 μm. **e**–**g**, Representative images (**e**) and quantification of the numbers (**f**; data are presented as mean ± s.e.m.) and density (**g**; data are presented as box plots with median (center line), 25th to 75th percentiles (box bounds), and minimum and maximum values (whiskers)) of G3BP2 condensates formed in WT and *Cse*^−/−^ MEFs upon 500 μM arsenite treatment. Scale bar, 20 μm. Recovery was conducted for 1 h. *n* = 3 biological replicates; at least 550 cells were quantified per condition; one-way ANOVA, Tukey’s HSD test for multiple comparisons. **h**, Representative images of human primary fibroblasts from a 48-year-old and a 79-year-old participant exposed to 500 μM arsenite over time. Recovery was conducted for 1 h; 200 μM of GYY4137 was used on the cell line from the 79-year-old individual (*n* = 3 independent replicates, several images per replicate). Scale bar, 20 μm. **i**, Western blot showing decreased CSE levels in human fibroblasts (normalized to GAPDH levels). Data are presented as the mean ± s.d. (*n* = 3 biological replicates, Welch’s *t*-test). **j**,**k**, Quantification of numbers (**j**) and density (**k**) of the G3BP2 condensates in human primary cell lines (*n* = 4 biological replicates, at least 300 cells were quantified per condition; one-way ANOVA, Tukey’s HSD test for multiple comparisons). Data are presented as the mean ± s.d. in **j** and mean ± s.e.m. in **k**. Rec., recovery; yo, years old. Source data

**Extended Data Fig. 9:**
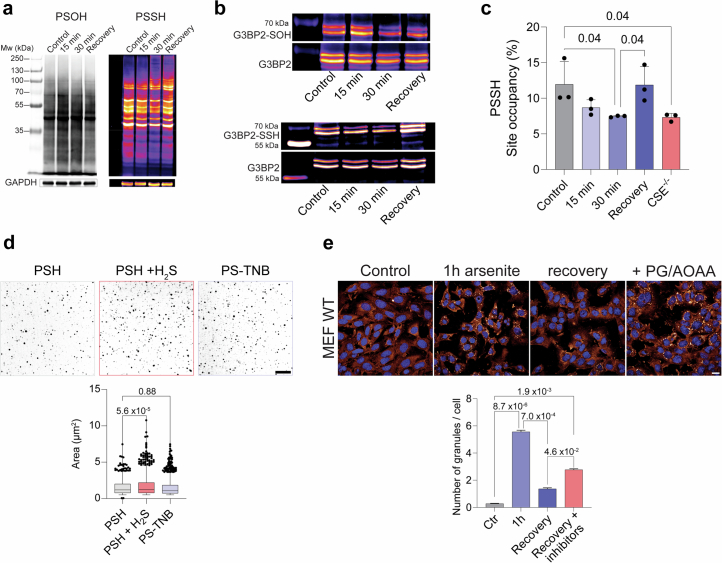
PSOH/PSSH changes regulate G3BP2-mediated SG dynamics. (**a**) Representative gels for global PSOH and PSSH changes in MEF cells treated with 500 μM NaAsO_2_ for 15 and 30 min. After 30 min NaAsO_2_ was removed, and cells were allowed to recover for 30 min (recovery). BTD-labelling was used for PSOH, and the dimedone-switch method with DCP-Bio1 was used for PSSH. Quantification is shown in Fig. 5a. (**b**) Representative western blot of G3BP2 sulfenylation and persulfidation upon the same treatment as in (**a**). Quantification is shown in Fig. 5b. (**c**) Changes in G3BP2-persulfidation occupancy of C73 upon arsenite treatment and recovery in WT MEF. Untreated *Cse*^−/−^ MEF was used for comparison. Site occupancy was calculated using sum of all cysteine peptides with different modifications. Absence of the value was considered as 0. Spectra of all identified peptides are shown in Supplementary Fig. 7. Data are presented as mean ± SD (n = 3 biological replicates, one-way ANOVA, Tukey’s HSD Test for multiple comparisons). (**d**) Representative images of G3BP2-Cy3 condensates (10 μM) upon 30 min treatment with H_2_S or DTNB. Quantification of droplet area is presented as box plots show median (centre line), 25th–75th percentiles (box bounds), and 1.5× IQR (whiskers)(n = 599 (PSH), 1046 (PSH + H_2_S), 1649 (PS-TNB) condensates over 3 independent reconstitution, one-way ANOVA, Dunnet’s test for multiple comparisons against PSH condition). Scale bar = 20 μm. (**e**) Representative images of G3BP2 condensates upon arsenite treatment and recovery in WT MEF. 15 min before recovery, cells were treated or not with 1 mM cocktail of H_2_S production inhibitors (propargylglycine, PG and aminooxyacetic acid, AOAA), treatment was kept during recovery time (1 h). Quantification of SG number per cell is shown below (n = 3 biological replicates at least 700 cells were quantified, One-way ANOVA, Tukey’s HSD Test for multiple comparisons. Data are presented as mean ± SEM.). Scale bar = 20 μm. Source data

Our previous work identified a connection between PSOH and PSSH in which a signaling wave of PSOH was followed by a resolving wave of PSSH^4^. To understand the role of PSSH in G3BP2 SG dynamics, we examined changes in PSOH and PSSH in cells exposed to arsenite, as well as during recovery from stress. Sulfenylation profiles showed that arsenite treatment caused a global increase in cellular PSOH, which subsequently declined during the recovery phase, whereas PSSH levels were significantly reduced during stress but increased markedly during recovery (Fig. 5a and Extended Data Fig. 9a). We used BTD or dimedone-switch labeling followed by immunopulldown of G3BP2 to assess the kinetics of sulfenylation and persulfidation, respectively; these changes mirrored the global cellular trend (Fig. 5b and Extended Data Fig. 9b). Proteomic analysis showed that 10–15% of G3BP2’s cysteine was present in the persulfidated form (Extended Data Fig. 9c and Supplementary Fig. 5). These observations suggest that C73 may function as a redox switch, converting droplet-stimulating PSOH to droplet-destabilizing PSSH.

To test this hypothesis, we first used purified protein. As G3BP2 contains only one cysteine, the most efficient way to generate the persulfide is to first produce the G3BP2-TNB adduct using Ellman’s reagent and then form PSSH by addition of near-equimolar H_2_S (refs. ^37,55^) (Extended Data Fig. 8a). Preformed droplets of G3BP2-TNB (indistinguishable in size from control G3BP2; Extended Data Fig. 9d) began to dissolve upon H_2_S addition and fully disappeared as the reaction progressed, indicating that persulfidation dissolved G3BP2 condensates (Fig. 5c and Supplementary Video 4). This effect was not observed when H_2_S was added to fully reduced protein (Extended Data Fig. 9e).

Next, we monitored H_2_S-induced dissolution of G3BP2 granules in cellulo. MEFs expressing either WT or C73S GFP-tagged G3BP2 were exposed to 500 µM arsenite stress, followed by H_2_S addition. Upon H_2_S treatment, immediate dissolution of SGs was recorded in real time (Fig. 5d and Supplementary Video 5). Owing to the continued presence of a stressor, and the rapid consumption of H_2_S by cells^56^ and its loss to the gas phase^57^, SGs reformed over time; however, complete dissolution was reinduced by a second bolus of H_2_S (Fig. 5d and Supplementary Video 5). No SG dissolution was observed in cells expressing C73S-G3BP2 mutant, confirming that this was a cysteine-redox-mediated process (Fig. 5d and Supplementary Video 6). The reversible nature of the process, together with the dynamic PSOH/PSSH changes observed during stress and recovery (Fig. 5b), suggests that endogenously produced H_2_S has an important role in regulation of SG formation and duration.

As we have demonstrated, PSSH levels of G3BP2 decline in the aging brain and even more markedly in *Cse*-knockout animals, potentially impairing the ability of cells to dissolve SGs. Addition of CSE inhibitors during the recovery phase, when the stressor had been removed, slowed recovery and maintained high SG levels, supporting the hypothesis that CSE-dependent persulfidation has an essential role in SG disassembly (Extended Data Fig. 9f,g). Indeed, whereas SG dissolved in WT cells 1 h after stress removal, their number and size remained unchanged in *Cse*^−/−^ MEFs (Fig. 5e–g).

**Extended Data Fig. 10:**
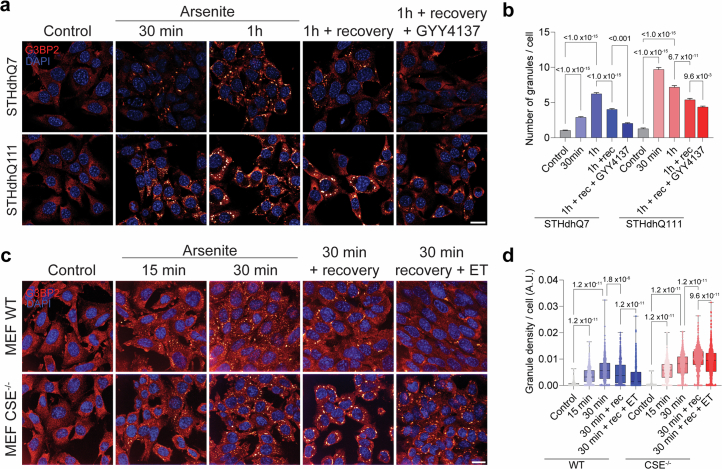
H_2_S donors dissolve SG in cells. (**a**) Representative images and (**b**) granule number per cells quantification of G3BP2 condensates upon 500 µM arsenite treatment and recovery (1 h) in STHdhQ7 and STHdhQ111 cells. During recovery, cells were treated or not with 200 µM of GYY4137. 450 cells quantified per conditions in 2 biological replicates with at least 6 images per replicate, One-way ANOVA, Tukey’s HSD Test for multiple comparisons. Data are presented as mean ± SEM. (**c**) Representative images and (**d**) granule density per cells quantification of G3BP2 condensates upon 500 µM arsenite treatment and recovery (30 min) in WT and *Cse*^−/−^ MEF. For the recovery, the cells were treated or not with 400 µM ergothioneine (ET). Ergothioneine has been shown to induce H_2_S production in a CSE-dependent manner. Box plots show median (centre line), 25th–75th percentiles (box bounds), and min to max values (whiskers). At least 500 cells were quantified per condition in 3 biological replicates with at least 6 images per relicate. One-way ANOVA, Tukey’s HSD Test for multiple comparisons. Source data

We further tested this hypothesis in a more brain-relevant cell type, striatal neurons (STHdh cells). STHdh cells derived from Huntington’s disease mice (*STHdh*^*Q111/Q111*^) have barely detectable CSE and PSSH levels relative to WT-derived *STHdh*^*Q7/Q7*^ cells^4^. Arsenite stress caused a time-dependent increase in SG number and size in *STHdh*^*Q7/Q7*^ cells, which was reversed upon recovery (Extended Data Fig. 10a,b). Addition of H_2_S donor GYY4137 during recovery almost completely dissolved the remaining SG. By contrast, *STHdh*^*Q111/Q111*^ cells displayed stronger and faster responses to arsenite stress and contained significant numbers of SGs even after 1 h of recovery (Extended Data Fig. 10a,b), akin to Cse^−/−^ MEFs. Although GYY4137 reduced SG numbers in these cells, this effect was modest, consistent with a limited benefit of H_2_S donors in cells that are generally unable to convert PSOH to PSSH and instead accumulate higher PSO_*x*_H levels^4^ (Fig. 1a). To confirm that these effects also occurred in aging cells, we used human primary fibroblasts from a 48-year-old donor and a 79-year-old donor (Fig. 5h). Fibroblasts from the older donor exhibited clearly reduced CSE expression (Fig. 5i), corroborating our observations that aging leads to CSE decline. When exposed to arsenite stress and subsequently allowed to recover, fibroblasts from the older donor showed markedly impaired SG dissolution compared to those from the younger donor, an effect that was rescued by GYY4137 (Fig. 5j,k).

These results confirm the importance of endogenous H_2_S production in maintenance of normal SG kinetics and highlight the therapeutic potential of exogenous H_2_S donors for dissolving pathological condensates. We recently described a mechanism by which ergothioneine (ET) acts as a prolongevity drug with long-term healthspan benefits, serving as an alternative substrate for CSE, producing H_2_S and increasing protein persulfidation^11^. As a proof of concept, we compared the ability of MEFs treated with ET to recover from stress. ET treatment decreased the size and number of SGs, but only in WT cells. *Cse*^−/−^ cells, which are unable to metabolize ET to H_2_S, were unaffected by the treatment (Extended Data Fig. 10c,d).

### Age-induced redox disbalance leads to protein aggregation

The inability of cells to dissolve biomolecular condensates is considered to be a key feature of aging and a driver of pathological protein aggregation^52,58,59^. TAR DNA-binding protein 43 (TDP-43) is a highly conserved nuclear RNA/DNA-binding protein that is involved in the regulation of RNA processing and is normally localized to the nucleus. Accumulation of TDP-43 aggregates in the cytosol of neurons is a common feature of many neurodegenerative diseases, including amyotrophic lateral sclerosis, frontotemporal dementia and Alzheimer’s disease^60–64^. TDP-43 is a component of SGs, and its predominant cytoplasmic localization is often interpreted as a sign of pathogenicity^60,64^. We assessed TDP-43 levels in WT and *Cse*^−/−^ cells and found them to be unchanged; however, the TDP-43 cytosol/nucleus ratio was significantly higher in *Cse*^−/−^ cells and increased further under arsenite stress (Supplementary Fig. 6a,b). Colocalization studies of G3BP2-positive SGs with TDP-43, TIA-1 and EIF4G showed marked differences between WT and *Cse*^−/−^ cells exposed to stress (Supplementary Fig. 6c–e). *Cse*^−/−^ cells exhibited larger G3BP2-, TDP-43- and EIF4G-positive granules (Supplementary Fig. 6d), although their numbers of SGs were not necessarily higher (Supplementary Fig. 6e), suggesting that impaired granule dissolution could create nucleation centers for protein aggregation. We therefore performed filter trap experiments and confirmed that TDP-43 was more aggregated in *Cse*^−/−^ cells (Supplementary Fig. 7a).

**Fig. 6:**
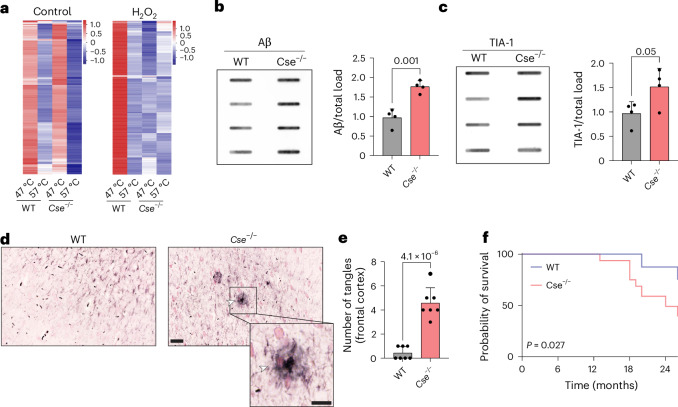
Age-induced thiol remodeling increases protein aggregability. **a**, Thermal proteome stability assay between WT and *Cse*^−/−^ MEFs (*n* = 2 biological replicates). **b**,**c**, Filter trap experiments using 18-month-old brain WT and *Cse*^−/−^ mice lysate with antibodies against amyloid-β (Aβ) (**b**) and TIA-1 (**c**) (*n* = 4 animals, Welch’s *t*-test; data are presented as the mean ± s.d.). **d**, Gallyas silver staining of brain sections from WT and *Cse*^−/−^ 18-month-old mice for neuritic-like plaques. Global stained sections are shown in Supplementary Fig. 8. The arrow points to an example of a neuritic-like plaque observed in the *Cse*^−/−^ brain section. Scale bar, 40 µm (20 µm in zoomed image). **e**, Quantification of tangles observed in cortex, shown as a histogram. Seven images from four different animals were analyzed. Data are presented as the mean ± s.d. (*n* = 4 animals, Welch’s *t*-test). **f**, Survival rates of *Cse*^−/−^ versus WT mice. *n* = 16 animals per group (two individual lifespan experiments of 8 animals per group in each experiment). Kaplan–Meier survival analysis and Mantel–Cox log-rank test were performed. Source data

In light of our proteomics findings that aging was associated with cysteine (hyper)oxidation, whereas beneficial persulfidation was reduced, and that cysteine (hyper)oxidation drove biomolecular condensation, we hypothesized that aging, like *Cse* knockout, would result in stronger protein aggregation. Molecular dynamics modeling for some sulfonylated targets revealed that cysteine residues were typically located within or at the edges of β-sheets, leading to significant conformational changes (Fig. 2g). If these changes mimicked phosphorylation-driven effects (and hyperphosphorylation of certain proteins is linked to neurodegenerative diseases), then proteins with hyperoxidized cysteine residues might be more prone to aggregation. To test this hypothesis, we treated WT and *Cse*^−/−^ MEFs with H_2_O_2_ and performed a thermal proteome profiling stability assay, focusing on the beginning and end of the melting curve (47 °C versus 57 °C). The proteome of *Cse*^−/−^ MEFs, which have reduced H_2_S production to counteract thiol hyperoxidation^4^, was significantly destabilized by H_2_O_2_, with the majority of identified proteins losing their stability and aggregating from the solution even at 47 °C (Fig. 6a and Supplementary Table 6). These findings support the notion that the age-related shift from persulfidation to sulfonylation enhances the aggregation potential of proteins as well as affecting their LLPS propensity.

We therefore examined the levels of well-known neurodegenerative culprit amyloid-β in WT and *Cse*^−/−^ brain samples using a filter trap assay and found significantly higher amyloid-β aggregation in 18-month-old *Cse*^−/−^ mice compared with WT mice (Fig. 6c). There was no significant effect on aggregation of G3BP2 and TDP-43, although aggregation of both showed an upward trend in *Cse*^−/−^ mice (Supplementary Fig. 7b,c). By contrast, TIA-1, an SG component and prion-like protein, was significantly more abundant in the aggregated fraction (Fig. 6d). Notably, levels of intramolecular disulfides did not differ between WT and *Cse*^−/−^ samples, indicating the importance of other redox cysteine PTMs (Supplementary Fig. 7d).

To assess potential neurodegenerative pathology, we examined brain sections from 18-month-old *Cse*^−/−^ mice and WT littermates for the presence of neuritic-like plaques through Gallyas silver staining. As expected, WT mice showed almost no positive staining, with only one plaque observed in the cortex for three of seven analyzed brain sections (Fig. 6e). By contrast, *Cse*^−/−^ mice exhibited numerous neuritic-like plaques throughout the brain, including in the frontotemporal region, cerebellum and hippocampus (Fig. 6e and Supplementary Fig. 8). No plaques were observed in 2-month-old *Cse*^−/−^ mice, indicating that formation of these plaques is age-dependent (Supplementary Fig. 9). Further investigation, to be reported elsewhere, indicated that these tangles in 18-month-old *Cse*^−/−^ mice contained pathological phospho-tau^65^.

These findings suggest that aging combined with reduced H_2_S production drives significant thiol remodeling. This may have widespread implications for cellular survival in *Cse*^−/−^ mice. These effects are unlikely to be restricted to the brain and may be widespread, as G3BP-dependent SG assembly and disassembly have a universal role in cellular stress protection. With aging, impaired resolution of biomolecular condensates can disrupt cellular homeostasis and contribute to a decline in healthspan, ultimately affecting overall lifespan. Indeed, *Cse*^−/−^ mice exhibited a higher mortality rate before 18 months of age. Comparative lifespan analysis revealed that *Cse*^−/−^ mice had a significantly shorter lifespan than their WT littermates (Fig. 6f).

## Discussion

The origins of life are deeply intertwined with the presence of H_2_S, which, alongside cyanide, had a pivotal role in the genesis of biomolecules^66^. Cysteine, in turn, catalyzed the first peptide syntheses^67^, whereas LLPS and biocondensate formation have been fundamental in shaping the evolution of life as we know it^68–70^. These fundamental chemical and biophysical processes remain essential for cellular function and homeostasis. This study reveals the transformative potential of cysteine PTMs on protein LLPS, highlighting the regulatory role of protein persulfidation and establishing a biochemical link between aging and phase separation.

The aging brain appears to be overwhelmed by oxidative stress, as evidenced by our sulfenylome and sulfonylome data. The role of ROS in aging has been the subject of both interest and controversy^1,71–73^. Our findings demonstrate that steady-state levels of PSOH, formation of which represents the first thiol oxidation step in the reaction with ROS, increase continuously with aging, eventually leading to formation of PSO_3_H. This process is partially mitigated by endogenous H_2_S production. Notably, protein persulfidation promotes dispersion of phase-separated condensates, whereas cysteine oxidation drives enhanced phase separation, ultimately leading to protein aggregation. In the aging brain, these changes could lead to (1) compromised synaptic release due to disrupted synapsin 1–synaptic vesicle condensates, (2) increased and unresolved phase separation of G3BP2 and (3) accumulation and aggregation of proteins.

Modeling the effects of cysteines using serine or glutamate mutations produced opposite shifts in the hydrophobic character of the corresponding local environment: hydrophobicity decreased twofold around G3BP2 C73 but increased approximately twofold around synapsin 1 C360 (Supplementary Fig. 10). These divergent behaviors indicate that local packing effects are highly context-specific and that no single physicochemical parameter (for example, hydrophobicity) uniformly explains the functional outcomes. Likewise, because PSSH, PSOH, and PSO_x_H modifications are all expected to be deprotonated at physiological pH, changes in net charge cannot account for the markedly different effects of each oxidation state.

Instead, we propose that persulfidation at thiolate hotspots acts as a general antisticker modification. By extending the cysteine side chain with an additional sulfur atom and increasing local solvation, polarizability and conformational flexibility^37^, persulfidation disrupts thiolate-dependent contacts and introduces dynamic S–S exchange. These features collectively reduce the effective multivalency of the interaction network. In G3BP2, this could manifest as weakened couplings among the NTF2-like domain, low-complexity regions and RNA, whereas in synapsin 1 it could diminish the cohesive interactions required for crowding-enhanced condensation with synaptic vesicles. In both systems, the consequence is dissolution of the condensate. Future detailed computational studies will provide physicochemical explanations for these observations. By contrast, irreversible overoxidation to cysteine PSO_3_H introduces a rigid, strongly electronegative SO_3_H group that acts as a local anchoring point, akin to phosphorylation^74^; this enhances cohesion and promotes larger, more persistent droplets. Taken together, these observations indicate that the behaviors of cysteine redox PTMs are best understood through their differential effects on sticker strength rather than through global physicochemical variables such as net charge^75,76^.

**Fig. 7:**
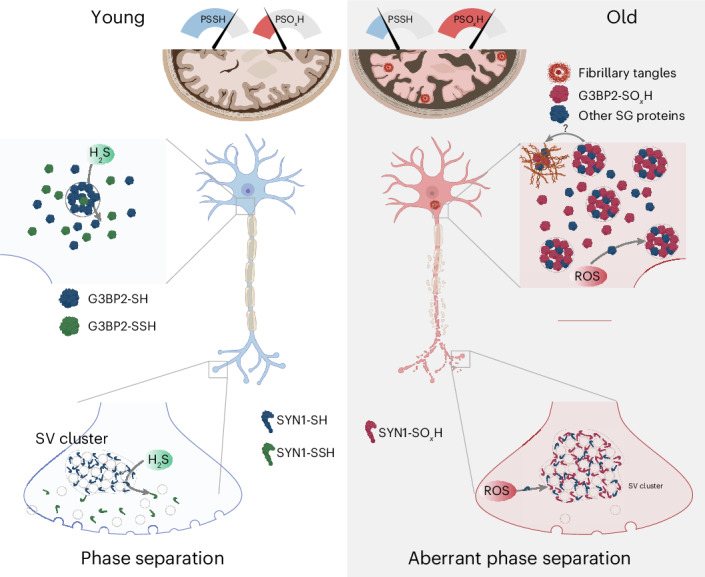
Age-dependent redox remodeling and its impact on phase separation in the aging brain. In young cells, abundant PSSH maintains dynamic, reversible condensates that enable efficient dissolution of SGs and synaptic vesicle clusters. With aging, loss of PSSH and accumulation of PSO*ₓ*H shift these proteins toward more persistent condensates, promoting aberrant phase separation and predisposing cells to pathological aggregation. SV, synaptic vesicle. Figure created in BioRender; Filipovic, M. https://biorender.com/kveknhn (2026).

A hallmark of cellular homeostasis is the ability to assemble SGs rapidly in response to stress to safeguard RNA and to disassemble them efficiently once the stress has been resolved^52,77^. With age, however, declining persulfidation and a concomitant increase in PSOH and PSO_*x*_H species impair this resolution step: SGs persist even after the stressor is removed (Fig. 7). As oxidized proteins remain sequestered within condensates for prolonged periods, their altered interactions promote aberrant stabilization and eventually aggregation, establishing a self-reinforcing pathological loop. This defect is exemplified by *Cse*^−/−^ cells, which fail to dissolve SGs long after stress withdrawal, a phenotype that can be rescued by supplying an H_2_S donor. Thus, the age-associated loss of persulfidation predisposes proteins to prolonged LLPS and conformational states that favor aggregation, consistent with our observations of elevated protein aggregation in the *Cse*^−/−^ mouse brain. These findings indicate that *Cse*^−/−^ mice provide a valuable model for studying age-associated neurodegeneration. Notably, even short treatment of *Cse*^−/−^ cells with H_2_O_2_ resulted in a global leftward shift in their proteome melting curves, indicating increased protein aggregability.

This thiol-based tuning of condensate dynamics is likely to represent an endogenous regulatory mechanism that extends beyond classical redox signaling and has the potential to influence broad cellular and metabolic processes. More broadly, these observations help to explain why thiol signaling rarely conforms to a one-modification, one-target, one-function paradigm: the outcome of cysteine oxidation depends on how each modification alters the physical interaction landscape of the protein rather than on a singular biochemical switch. However, age-induced remodeling of these thiol modifications shifts the balance toward unresolved biomolecular condensation, ultimately leading to a global collapse of cellular homeostasis and predisposing proteins to pathological aggregation (Fig. 7).

Emerging H_2_S-releasing medicines could therefore have potential therapeutic applications in strategies to restore the balance of age-related thiol oxidation and its effects on LLPS in neurodegenerative disorders. For example, we have demonstrated that ET, an alternative substrate of CSE that restores PSSH^11^, promotes SG dissolution. Notably, ET intake has been linked to reduced cognitive decline in patients with Alzheimer’s disease^78^. Persulfidation could thus represent a unifying, reversible mechanism that counteracts LLPS at thiolate hotspots across diverse protein condensate systems.

## Methods

### Mice

WT and *Cse* KO C57BL/6 mice^79^ were bred and housed in ventilated cages, under specific-pathogen-free, temperature-controlled (22 °C) and 12 h light/dark cycle conditions in full compliance with the guidelines of the Federation of Laboratory Animal Science Association recommendations in the Laboratory Animal Unit of Biomedical Research Foundation of the Academy of Athens and allowed unrestricted access to a chow diet and water. Per Greek regulations, no individual license is required for experiments in which animals are not treated. All experimental procedures reported here were approved by the Veterinary Authority of the Prefecture of Athens, in accordance with National Registration (Presidential Decree 56/2013), harmonized with European Directive 2010/63/EU. All studies were performed on male mice at the indicated ages.

### Cell culture

*Cse*^−/−^ MEFs were from B. Paul (Johns Hopkins University) and were generated from *Cse*^+/+^ and *Cse*^−/−^ mice and immortalized using SV40T antigen^27^. MEFs were cultured in Dulbecco’s modified Eagle medium (DMEM) supplemented with 2 mM l-glutamine, 1% penicillin–streptomycin, and 10% fetal bovine serum at 37 °C and 5% CO_2_. Human primary fibroblasts (AG1449, from a 48-year-old donor; and AG16359, from a 79-year-old donor) were obtained from the Coriell Institute for Medical Research. Cells were cultured following the same conditions used for MEFs, with the exception that no antibiotics were added to the medium. Striatal progenitor cell line *STHdh*^*Q7/Q7*^, expressing WT huntingtin, and *STHdh*^*Q111/Q111*^, expressing mutant huntingtin harboring 111 glutamine repeats (referred to as Q7 and Q111 cells, respectively) were maintained in DMEM (low glucose, no pyruvate) supplemented with 2 mM l-glutamine, 1% penicillin–streptomycin, and 10% fetal bovine serum at 33 °C and 5% CO_2_.

### Persulfidation enrichment for proteomic analysis

Brain frontal cortexes were lysed in cold HENS buffer (50 mM HEPES, 1 mM EDTA, 0.1 mM neocuproine, 1% IGEPAL, 2% SDS, pH 7.4) supplemented with 5 mM NBF-Cl. Frozen tissue was disrupted in lysis buffer using a TissueLyser II (Qiagen) and 5-mm stainless steel beads. Persulfidation enrichment was performed using a previously described method^4^. Enriched proteins were eluted by incubating beads with 2.25 M ammonia solution for 16 h and collected the following morning for lyophilization. Lyophilized samples were dissolved in digestion buffer (50 mM ammonium bicarbonate (ABC), 1 mM calcium chloride) and digested overnight using trypsin at a trypsin/protein ratio of 1:20. Desalting was performed on a Supel-Select HLB SPE Tube. Eluates were evaporated under vacuum until dry. Peptides were dissolved in 0.1% trifluoroacetic acid (TFA), and digestion and desalting quality control was performed using an Dionex UltiMate 3000 (RSLC) system with a PepSwift Monolithic Trap 200 µm × 5 mm (Thermo Fisher Scientific).

### Sulfenylation enrichment for proteomic analysis

Brain frontal cortexes were lysed in cold HEN buffer supplemented with 0.5 mM BTD synthesized in-house following an established protocol^20^. Lysates were incubated for 2 h at 37 °C, followed by 2 methanol/chloroform precipitations. Sulfenylation enrichment was carried out using a modified version of a method from Fu et al.^18^. In brief, samples were resuspended in HEPES + 2% SDS and treated with 10 mM DTT for 1 h at 37 °C, followed by 40 mM IAM for 30 min at room temperature (RT). Excess DTT and IAM were removed by methanol/chloroform precipitation, and proteins were resuspended in 50 mM HEPES pH 7.0, 2% SDS. Then, 1.5 mg samples of proteins were incubated for 2 h at 37 °C with 0.2 mM Az ultraviolet (UV)-cleavable-biotin, 1 mM ascorbic acid and 0.1 mM copper–TBTA to perform the click chemistry reaction. Precipitated proteins were dissolved in neutravidin binding buffer (50 mM NaOAc, pH 4.5) and 0.2% SDS. Protein concentration was performed, and 0.9 mg of proteins were incubated with Pierce High Capacity NeutrAvidin Agarose beads. After 3 h of mixing at RT, beads were loaded onto columns and washed with neutravidin washing buffer (50 mM NaOAc and 2 M NaCl, pH 4.5). Sulfenylated proteins were then released by incubation of beads for 3 h under UV light (365 nm) in 25 mM ABC buffer. Supernatants were collected and lyophilized. Samples were then digested and desalted as detailed above. Peptides were dissolved in 0.1% TFA, and digestion quality control was performed as described.

### LC–MS/MS analysis

#### Persulfidome

Peptides were dissolved in 0.1% TFA before being analyzed by high-resolution liquid chromatography coupled with tandem mass spectrometry (LC–MS/MS) using an Ultimate 3000 Nano Ultra High-Pressure Chromatography (UPLC) system (Thermo Fisher Scientific) coupled with a Thermo LTQ Velos mass spectrometer via an EASY-spray (Thermo Fisher Scientific). Peptide separation was carried out with an Acclaim PepMap 100 C18 column (Thermo Fisher Scientific) using a 140-min nonlinear gradient from 7% to 80% B (0 min, 7% B; 15 min, 17% B; 30 min, 30% B; 120 min, 65% B; 140 min 80% B; B: 84% acetonitrile, 0.1% formic acid) at a flow rate of 250 nl min^−1^. The LTQ Velos was operated in data-dependent acquisition (DDA) mode, and MS1 survey scans were acquired from *m*/*z* 300 to 2000 at a resolution of 60,000 using the Orbitrap mode. The 10 most intense peaks with charge state ≥2 were fragmented using collision-induced dissociation (CID) set to 35% and fragmentation analyzed using IonTrap at a standard scan rate. The dynamic exclusion was set to 20 s.

#### Sulfenylome

Peptides were analyzed by high-resolution LC–MS/MS using an Dionex UltiMate 3000 RSLC system (Thermo Fisher Scientific) coupled with an Orbitrap Eclipse Tribrid mass spectrometer via an EASY-spray (Thermo Fisher Scientific). Peptide separation was carried out with an Acclaim PepMap 100 C18 column (Thermo Fisher Scientific) using a 65-min linear gradient from 3% to 35% of B (84% acetonitrile, 0.1% formic acid) at a flow rate of 250 nl min^−1^. The Orbitrap Eclipse was operated in DDA mode, and MS1 survey scans were acquired from *m*/*z* 300 to 1,500 at a resolution of 120,000 using the Orbitrap mode. MS2 scans were carried out for 3 s with high-energy CID (HCD) at 32% using the normal-speed IonTrap mode.

Data were evaluated with PEAKS ONLINE software using 15 ppm for precursor mass tolerance, 0.5 Da for fragment mass tolerance, specific tryptic digest and a maximum of 3 missed cleavages. For persulfidation detection, NBF (+163.00961594 Da) on C, K and R; DCP (+168.0786442 Da) on C; amino-terminal acetylation (+42.010565 Da); and methionine oxidation (+15.994915 Da) were added as variable modifications. For sulfenylation detection, the BTD–UV-cleavable probe (cleaved, +418.1311 Da), carbamidomethylation (+ 57.021464 Da) on C, N-terminal acetylation (+ 42.010565 Da) and methionine oxidation (+15.994915 Da) were added as variable modifications; FDR was controlled at 1% at both the peptide-spectrum match (PSM) and protein levels. Quantification was carried out at the MS1 level, and the match between run function with default filtering parameters was used. Proteins with at least one unique peptide were considered for quantification.

#### Total proteome analysis

Brain frontal cortexes were disrupted in lysis buffer (50 mM TRIS, 150 mM NaCl, 2% SDS, 1% IGEPAL, pH 7.8) using a Tissuelyser II and 20 1.4-mm ceramic beads (Qiagen, cat. no. 13113). Lysates were incubated with benzonase following the manufacturer’s recommendation for 30 min and precipitated. Samples were then resuspended in 50 mM HEPES with 2% SDS and incubated with 10 mM DTT at RT, followed by a further incubation with 50 mM IAM for 1 h at 37 °C. Precipitation was used to stop the reaction and concentrate proteins. For tryptic digestion, samples were resuspended in 6 M guanidine hydrochloride. Then, 250-µg samples of protein were digested overnight at 37 °C using trypsin, with a 1:20 trypsin/sample ratio (Promega, V5117). The next day, peptides were desalted and evaporated under vacuum until dry as described above.

Peptides were dissolved in 0.1% TFA, and digestion quality control was performed as described above. Peptides were analyzed by high-resolution LC–MS/MS using an Dionex UltiMate 3000 RSLC system (Thermo Fisher Scientific) coupled to a timsTOF Pro (Bruker) equipped with a CaptiveSpray source. Peptide separation was carried out with an Acclaim PepMap 100 C18 column (Thermo Fisher Scientific) using a 65-min linear gradient from 3% to 35% of B (84% acetonitrile, 0.1% formic acid) at a flow rate of 250 nl min^−1^. The timsTOF Pro (Bruker) was operated in PASEF mode using Compass Hystar 5.0.36.0. The TIMS settings were as follows: mass range, 100–1,700 *m*/*z*; 1/*K*_0_ start 1.6 Vs cm^−2^, end 1.6 Vs cm^−2^; ramp time, 100 ms; lock duty cycle to 100%; capillary voltage, 1,500 V, dry gas 3 l min^−1^; dry temperature, 180 °C. The PASEF settings were: 10 MS/MS scans; charge range, 0–5; active exclusion for 0.4 min; scheduling target intensity, 20,000; intensity threshold, 2,500; CID collision energy, 49 eV.

Data were evaluated with PEAKS ONLINE software using 20 ppm for precursor mass tolerance, 0.5 Da for fragment mass tolerance, specific tryptic digest and a maximum of 3 missed cleavages. Carbamidomethylation (+57.021464 Da) on C, N-terminal acetylation (+42.010565 Da) and methionine oxidation (+15.994915 Da) were added as variable modifications; FDR was controlled at 1% at both PSM and protein levels. Data were normalized based on the total ion count (TIC). Quantification was carried out at the MS1 level, and the match between run function with default filtering parameters was used. Proteins with at least one unique peptide were considered for quantification.

#### Direct detection of sulfonylated peptides using LC/MS

First, 50 µg of each of the NBF-labeled samples dissolved in PBS with 2% SDS (‘PSSH enrichment for proteomic analysis’) was trypsinized overnight as described above. Samples were subjected to desalting and Pierce Detergent Removal columns (ThermoFisher, cat. no. 87777). Peptides were evaporated under vacuum until dry as described above.

Peptides were dissolved in 0.1% TFA, and digestion quality was assessed as previously described. Peptides were analyzed by high-resolution LC–MS/MS using a Dionex UltiMate 3000 RSLC system (Thermo Fisher Scientific) coupled to an Orbitrap Eclipse Tribrid mass spectrometer via an EASY-spray (Thermo Fisher Scientific). Peptide separation was carried out with an Acclaim PepMap 100 C18 column using a 160-min linear gradient from 3% to 35% of B (84% acetonitrile, 0.1% formic acid) at a flow rate of 250 nl min^−1^. Parameters were set as described above.

Data were evaluated with PEAKS ONLINE software using 15 ppm for precursor mass tolerance, 0.5 Da for fragment mass tolerance, specific tryptic digest and a maximum of 3 missed cleavages. NBF (+163.00961594 Da) on C, K and R; ‘Cysteine oxidation to cysteic acid’ (–SO_3_H, +47.984745 Da, Unimod ID: 345) on C; N-terminal acetylation (+42.010565 Da); and methionine oxidation (+15.994915 Da) were added as variable modifications. FDR was controlled at 1% at both PSM and protein levels.

### Proteomic bioinformatic analysis

#### Persulfidation and sulfenylation

Persulfidation and sulfenylation datasets were searched against the UNIPROT mouse database and filtered at three of four and two of three quantifiable values per group, respectively. Datasets were further filtered for proteins that contained at least one cysteine to avoid false discovery and normalized using the EigenMS R script^80^. Each group was then tested against the others using Welch’s *t*-test, and *P* values were corrected using permutation-based FDR. All statistical tests were performed using Perseus^81^. Proteins with FDR-adjusted *P* ≤ 0.05 and fold change greater than 30% were considered to show significant changes and were included in downstream analyses.

Kyoto Encyclopedia of Genes and Genomes (KEGG) pathway enrichment^82^ and gene ontology (GO) term enrichment^83,84^ analyses were performed against the whole mouse genome background already integrated in the DAVID bioinformatics webtool^85,86^. Enriched KEGG pathways and GO terms were visualized using an R script developed in-house. Further semantic space analysis of GO term enrichment was performed using the REVIGO webtool^87^, and the results were plotted using R.

Protein localization was retrieved using QuickGO^88^ and searched against seven localizations from GO slims (cytoskeleton, Golgi apparatus, plasma membrane, nucleus, cytosol, endoplasmic reticulum and mitochondrion). Proteins with multiple localizations were included in all the compartments with which they were associated. Correlation scatter plots and kernel density estimations were generated using an in-house Python script.

#### Total proteome

A total proteome search was performed against the UniProt mouse database; the results were filtered at three of four quantifiable values and normalized by the TIC. Statistical tests were performed as described above for the persulfidation and sulfenylation datasets. Proteins were considered to have undergone significant change if they had an adjusted *P* < 0.05 and fold change ≥50%. Downstream analysis was performed as described previously.

#### Sulfonylated cysteine peptide analysis

Quantification of sulfonylated peptides was performed following protocols recently developed for protein cyanylation^89^. Quantifiable sulfonylated cysteine peptides were filtered for three of four valid values for each group; protein quantification of the dataset was also performed. For each protein, the ratio of the mean value of each group was used to normalize the peptide quantification. For each peptide, the sulfonylated cysteine position in the protein was retrieved using an R script developed in-house. Peptides exclusively present in WT 18-month-old or *Cse*^−/−^ 10-week-old mice for at least three of four values were extracted; these are presented in a separate Excel sheet in Supplementary Tables.

### Immunoblotting

Cells or tissues were lysed in HEN buffer supplemented with 20 mM IAM and incubated at 37 °C for 2 h to allow complete cysteine blocking. Excess blocking reagent was removed by precipitation, as described above. Samples were then resuspended in HEPES + 2% SDS and readjusted using a DC protein assay. One equivalent of Laemmli (4×) buffer supplemented with 10% β-mercaptoethanol was added to three sample equivalents for SDS–PAGE, followed by boiling at 95 °C for 5 min protected from light. Equal amounts of proteins were resolved by SDS–PAGE using a standard protocol. Samples were then immunoblotted with the following antibodies: anti-PRDX-SO3 (Abcam, ab16830), 1:4,000; anti-oxDJ-1 (Sigma-Aldrich, MABN1773), 1:2000; anti-GAPDH-AF488 (Sigma-Aldrich, MAB374-AF488), 1:20,000; anti-TDP-43 (Proteintech, 10782-2-AP), 1:1,000; anti-β-tubulin (Sigma-Aldrich, T0198), 1:5,000; anti-rabbit-AF680 (Abcam, ab175773), 1:20,000; and anti-mouse-AF680 (Abcam, ab175775), 1:20,000.

### Filter trap assay

#### Cell lines

MEFs or STHdh cells were collected by scraping in cold PBS. Lysis was carried out by liquid homogenization with a 23-G needle and syringe in filter trap lysis buffer (150 mM NaCl, 50 mM HEPES, 1 mM EDTA, 1% Triton X-100, protease inhibitors (cOmplete, Mini, EDTA-free Protease Inhibitor Cocktail, Roche). Protein concentration was determined by Pierce BCA assay (Thermo Fisher Scientific, 23227), and 0.5% SDS (final concentration) was added to samples. Acetate cellulose blotting membrane (Cytiva, OE66 10404180) and Whatman filter paper were presoaked in PBS and equilibrated by 3 washes in lysis buffer containing 0.5% SDS following cassette assembly. After loading of 120 µg of protein, the membrane was washed 5 times in lysis buffer containing 0.2% SDS, followed by standard immunoblotting. The membrane was probed with anti-TDP-43-AF790 (1:500; Santa Cruz Biotechnology, sc-376311) and anti-G3BP2 (1:600; Proteintech, 16276-1-AP), followed by mouse anti-rabbit IgG-CFL 488 (Santa Cruz Biotechnology, sc-516248). Equal loading was ensured by SDS–PAGE, followed by immunoblotting using anti-GAPDH(6C5)-AF488 (Merck, MAB374).

#### Brain tissue

Brain frontal cortexes from WT and *Cse*^−/−^ mice at 18 months were lysed in cold filter trap lysis buffer. Disruption of frozen tissue was carried out in lysis buffer using an Omni Bead Ruptor 12 bead mill homogenizer (Camlab) and 1.4-mm ceramic beads. Lysates were centrifuged at 2,000*g* for 10 min at 4 °C. The filter trap assay was then performed as described for the cell lines. The membranes were probed with anti-TDP-43-AF790 (1:500; Santa Cruz Biotechnology, sc-376311), anti-TIA-1/TIAR(D-9)AF-647 (1:250; Santa Cruz Biotechnology, sc-48371), anti-GAPDH(6C5)-AF488 (1:250; Merck, MAB374) and mouse anti-Tau(D-8) (1:300; Santa Cruz Biotechnology, sc-166060), followed by m-IgG Fc BP-CFL555 (1:1,000; Santa Cruz Biotechnology, sc-533654) and mouse beta-amyloid(b-4) (1:300; Santa Cruz Biotechnology, sc-28365), then goat anti-mouse IgG(H+L)AF790 (1:1,000; Invitrogen, A11375) and anti-G3BP2, (1:600; Proteintech, 16276-1-AP), and finally mouse anti-rabbit IgG-CFL 680 (1:1,000; Santa Cruz Biotechnology, sc-516252). Equal loading was ensured by SDS–PAGE followed by immunoblotting using anti-GAPDH(6C5)-AF488 (Merck, MAB374).

The same lysates were also incubated with 1× Laemmli buffer with or without 10% β-mercaptoethanol. The samples prepared with β-mercaptoethanol were used as loading controls for the filter trap assays, whereas those prepared in nonreducing Laemmli buffer were run to assess global differences in disulfide bond formation between WT and *Cse*^*−/−*^ mice. Gradient SDS–PAGE followed by Coomassie Brilliant Blue staining was performed for visualization.

### LDH, PKM and pyruvate carboxylase activity assays

For all activity assays, the frozen tissues were disrupted in mild lysis buffer (25 mM HEPES, 250 mM sucrose, 20 mM MgCl_2_, 1 mM EDTA, pH 7.4) using a TissueLyser II and 5-mm stainless steel beads. Samples were then centrifuged at 14,000*g* for 15 min at 4 °C, and the supernatant was collected. Protein concentrations were determined, and samples were readjusted to comprise equal amounts and split into parts for LDH (Sigma, MAK066), PKM (Sigma, MAK072) and pyruvate carboxylase (Abcam, ab289840) activity assays, carried out following the manufacturers’ protocols.

### Direct detection of cysteine PTMs of synapsin 1 by LC–MS/MS

Brain tissues were lysed using the same protocol as for immunoblotting. A modified in-gel digestion protocol from was used to identify synapsin 1 cysteine peptides^90^. Equal amounts of protein were loaded and separated by SDS–PAGE. Gels were then stained for 2 min with Coomassie Brilliant Blue solution (1.2 mM Coomassie Brillant Blue, 10% acetic acid, 40% methanol in distilled water) and destained overnight in a decoloration solution (25% methanol, 8% acetic acid in distilled water). Once destained, the band corresponding to the size of the protein of interest (±5 kDa) was excised. Coomassie Brilliant Blue was removed by alternating washes with 50 mM ABC, 25 mM ABC and 50% acetonitrile each for 10 min at 37 °C, 450 rpm. Once free from the stain, gel pieces were dried using a speed vacuum concentrator. Gel pieces were then incubated for 10 min at 4 °C with 50 mM ABC, 1 mM CaCl_2_ and trypsin at 12.5 ng µl^−1^. Excess solution was removed, and samples were trypsinized for 16 h at 37 °C, 750 rpm. The following day, peptides were extracted by incubation of the gel pieces twice in 0.1% TFA for 15 min at 37 °C, 450 rpm. This step was followed by two incubations with 0.1% TFA + 60% acetonitrile for 15 min at 37 °C, 450 rpm. Extracts were combined and peptides evaporated to dryness.

Peptides were dissolved in 0.1% TFA, and digestion was controlled using the previously described method. Peptides were analyzed using a Dionex UltiMate 3000 RSLC system (Thermo Fisher Scientific) coupled to a timsTOF Pro (Bruker) equipped with a CaptiveSpray source. Peptide separation was carried out with an Acclaim PepMap 100 C18 column (Thermo Fisher Scientific) using a 120-min linear gradient from 3% to 35% of B (84% acetonitrile, 0.1% formic acid) at a flow rate of 250 nl min^−1^. The timsTOF Pro (Bruker) was operated as previously described.

Data were evaluated with PEAKS ONLINE software using 20 ppm for precursor mass tolerance, 0.5 Da for fragment mass tolerance, specific tryptic digest and a maximum of 3 missed cleavages. The entire mouse database was used for the search. SO-IAM (+73.020464 Da), SS-IAM (+89.086464 Da), ‘Cysteine oxidation to cysteic acid’ (–SO_3_H, +47.984745 Da, Unimod ID: 345), carbamidomethylation (+57.021464 Da) on C, N-terminal acetylation (+42.010565 Da) and methionine oxidation (+15.994915 Da) were added as variable modifications; PSM was performed, and proteins were filtered at FDR 1%. Data were normalized based on the TIC.

### Persulfidated G3BP2 immunopulldown and detection by LC–MS

WT and *Cse*^−/−^ MEFs at 90% confluency in a 10-mm dish were treated with 500 µM NaAsO_2_ or left untreated for the indicated times. For recovery, cells were collected 1 h after the medium was changed and removed from NaAsO_2_. Samples were lysed in HEN supplemented with 5 mM NBF-Cl. After 2-h incubation at 37 °C, proteins were precipitated using methanol/chloroform. Samples were then redissolved in 2% SDS and incubated with 100 µM Daz-2 for 1.5 h at 37 °C before precipitation. Samples were redissolved in PBS 0.1% SDS, and equal amounts of proteins were incubated for 1 h with 1 μg of G3BP2 antibody. SureBeads magnetic beads attached to protein A were added to samples, followed by incubation at 4 °C overnight. Samples were further incubated for 1 h at RT, and beads were washed 3 times with PBS 0.01% Tween and 4 times with PBS. The beads containing the G3BP2 pulldown were then resuspended in 50 mM ABC and 0.0125 µg µl^−1^ trypsin. Samples were trypsinized for 16 h at 37 °C. Supernatants were then collected, and peptides were desalted and dried as previously described. Peptides were dissolved in 0.1% TFA, and digestion quality control was performed as previously described.

Peptides were analyzed by high-resolution LC–MS/MS using an Ultimate 3000 Nano UPLC system (Thermo Fisher Scientific) coupled with an Orbitrap Eclipse Tribrid mass spectrometer via an EASY-spray (Thermo Fisher Scientific). Peptide separation was carried out with an Acclaim PepMap 100 C18 column (Thermo Fisher Scientific) using a 65-min linear gradient from 3% to 35% of B (84% acetonitrile, 0.1% formic acid) at a flow rate of 250 nl min^−1^. The Orbitrap Eclipse was operated in DDA mode, and MS1 survey scans were acquired from *m*/*z* 300 to 1,500 at a resolution of 120,000 using Orbitrap mode. MS2 scans were carried out for 3 s with HCD at 32% using the normal-speed IonTrap mode.

Data were evaluated with PEAKS ONLINE software using 15 ppm for precursor mass tolerance, 0.05 Da for fragment mass tolerance, specific tryptic digest and a maximum of 3 missed cleavages. The entire mouse database was used for the search. Pure S-Daz-2 (+193.0851266 Da); SO-NBF (+178.9967); NBF (+163.001791) on C, K and R; N-terminal acetylation (+42.010565 Da) and methionine oxidation (+15.994915 Da) were added as variable modifications; PSM was performed, and proteins were filtered at FDR 1%. Data were normalized based on the TIC.

### Sulfenylated G3BP2 immunopulldown and detection by LC–MS

WT and *Cse*^−/−^ MEFs at 90% confluency in a 10-mm dish were treated 500 µM NaAsO_2_ or left untreated for the indicated times. Samples were lysed in HEN supplemented with 0.5 mM of BTD. After 2-h incubation at 37 °C, proteins were precipitated using methanol/chloroform. Immunopulldown processing and LC–MS measurements were carried out the using the protocol described above for detection of persulfidated G3BP2.

Data were evaluated with PEAKS ONLINE software using 15 ppm for precursor mass tolerance, 0.05 Da for fragment mass tolerance, specific tryptic digest and a maximum of 3 missed cleavages. The entire mouse database was used for the search. Pure BTD (+245.045 Da), N-terminal acetylation (+42.010565 Da) and methionine oxidation (+15.994915 Da) were added as variable modifications; PSM was performed, and proteins were filtered at FDR 1%. Data were normalized based on the TIC.

### G3BP2 site occupancy in 10-week-old WT mouse brain

Brain lysates from 10-week-old WT mice that had undergone persulfidation or sulfenylation enrichment and been labeled with DCP-bio1 (for PSSH) and biotin–UV-cleavable probe (for PSOH) were readjusted so they comprised the same amounts. For each replicate, readjusted lysates were separated into two parts of equal amounts: one part was kept for whole-lysate measurements; the other part was incubated with neutravidin beads for 2 h at RT. After incubation, samples were placed into a Micro-Spin Columns (Thermo Fisher, cat. no. 89879) and spun down at 500*g* for 1 min. The flow-through was collected, and beads were washed twice with 2 volumes of resin with PBS and 0.1% SDS. Flow-through and washes were combined to form the flow-through fraction. Both whole lysate and flow-through were trypsinized as previously described and labeled with tandem mass tag (TMT) 6-plex (Thermo Fisher) following manufacturer protocol. Whole lysate and flow-through for all replicates were labeled with different TMT channels and multiplexed. The multiplexed samples were then cleaned of SDS and TMT using Detergent Removal Spin Columns (Thermo Fisher, cat. no. 87777), followed by a desalting step as previously described. Finally, samples were fractionated using a Pierce High pH Reversed-Phase Peptide Fractionation Kit.

Peptides were analyzed by high-resolution LC–MS/MS using an Ultimate 3000 Nano UPLC system (Thermo Fisher Scientific) coupled with an Orbitrap Eclipse Tribrid mass spectrometer via an EASY-spray (Thermo Fisher Scientific). Peptide separation was carried out with an Acclaim PepMap 100 C18 column using a 90-min linear gradient from 3% to 35% of B (84% acetonitrile, 0.1% formic acid) at a flow rate of 250 nl min^−1^. The Orbitrap Eclipse was operated in DDA mode, and MS1 survey scans were acquired from *m*/*z* 300 to 1,500 at a resolution of 120,000 using Orbitrap mode. MS2 scans were carried out for 3 s with HCD at 32% using Orbitrap mode with a resolution of 50,000.

RAW files were analyzed using FragPipe with TMT10 workflow parameters; 20 ppm was used for precursor mass tolerance and fragment mass tolerance. For persulfidation, NBF (+163.0096 Da) on C, K and R was added as a variable modification. For sulfenylation, the default workflow parameters were used for the search. FDR was controlled at 1% at both PSM and protein levels.

### EGFP–synapsin 1 in vitro phase separation

Recombinant full-length EGFP–synapsin 1 was expressed in a mammalian expression system and purified as previously described^91,92^. Before the experiment, EGFP–synapsin 1 was cleaned up from TCEP using a dialysis column, and the buffer was replaced with phase separation buffer (25 mM Tris, 150 mM NaCl, pH 7.4). The hyperoxidized (PSO_*x*_H) form of EGFP–synapsin 1 was obtained by treating the protein with 100 µM DTT and 200 µM H_2_O_2_ at 37 °C for 30 min. Thiol quantification based on Ellman’s assay confirmed full cysteine oxidation^93^. Persulfidated EGFP–synapsin 1 was formed by treating the protein with 100 µM H_2_O_2_ and 500 µM H_2_S for 5 min at 37 °C before addition of polyethylene glycol (PEG). Respective controls were carried out using the same DTT, H_2_O_2_ and H_2_S concentrations. For the FRAP experiment, 3% w/v PEG (PEG3000) was used to trigger phase separation of 10 µM EGFP–synapsin 1. Photobleaching was performed after 5-min incubation with PEG, and recovery was recorded for 10 min. Two to three independent preparations, done on separate days and with separate protein batches, were used per thiol modification.

To further confirm that cysteine modifications were responsible for the observed effect, we repeated the DTT + H_2_O_2_ and H_2_O_2_ + H_2_S treatments as previously described using GFP–synapsin 1 in which all cysteines had been preblocked with 300 µM IAM for 20 min at 37 °C. Synapsin 1 PSOH and PSSH formation were verified by monitoring in-gel fluorescence of modified proteins after chemoselective labeling with BTD coupled to Cy5 (ref. ^94^) and Daz-2 coupled to Cy5 (ref. ^4^), respectively.

### Primary neuron experiments

Primary hippocampal neurons were prepared from newborn pups (postnatal day 0 or 1), and exocytosis assays were performed as described recently^6^. Neurons were preincubated with GYY4137 (100 µM) or propargylglycine (1 mM) and aminooxiacetic acid (1 mM). During imaging, neurons were first perfused with Tyrode’s solution supplemented with 2.5 mM KCl (low KCl); subsequently, to stimulate exocytosis, they were perfused with Tyrode’s solution supplemented with 92.5 mM KCl (high KCl) for 5 min; finally, neurons were briefly perfused with Tyrode’s solution containing 50 mM NH4Cl to alkalize the sample and visualize the total fluorescence from synaptophysin–pHluorin.

### G3BP2 in vitro phase separation

Total RNA from *STHdh*^*Q7/Q7*^ cells was extracted using an RNeasy Midi Kit following the manufacturer’s protocol. Total RNA was resuspended in RNase-free water, and concentration was assessed using a NanoDrop.

G3BP2 was purchased from Biomol (cat. no. 349926). First, isolated G3BP2 was labeled with a Cyanine3 NHS ester probe. The protein buffer was briefly exchanged with labeling buffer (100 mM sodium bicarbonate, 50 mM NaCl, pH 8.0) using Micro Bio- Spin 6 columns following the manufacturer’s protocol. The protein was then incubated for 1 h at RT with the Cyanine3 NHS ester probe at a ratio of 1:3 (protein/Cyanine3). After incubation, excess probe was removed using Micro Bio-Spin 6 columns, and the buffer was exchanged with phase separation buffer. To minimize the effect of the Cy3- label on phase separation, the labeled proteins were mixed with nonlabeled G3BP2 at a 1:9 ratio (labeled/nonlabeled). For hyperoxidized (PSO_x_H) G3BP2, 10 μM of protein was incubated in the presence of 100 μM DTT and 200 μM H_2_O_2_ for 30 min at 37 °C. Thiol quantification based on Ellman’s assay confirmed full cysteine oxidation^93^^.^ The reduced (PSH) and oxidized (PSOH/PSSP) G3BP2s were generated using the same concentration of DTT or H_2_O_2_, respectively. To further confirm that cysteine modifications were responsible for the observed effect, we repeated the DTT or H_2_O_2_ treatments as previously described using Cy3–G3BP2 in which cysteine had been preblocked with 300 µM IAM for 20 min at 37 °C.

For all experiments, G3BP2 phase separation was triggered by incubation with 50 ng µl^−1^ of total RNA extract for 7 min prior to FRAP. Recovery was recorded for 10 min after photobleaching.

#### Persulfidation-driven dissolution of G3BP2 droplets

First, 20 µM of nonlabeled G3BP2 was incubated with 40 µM of DTNB and 50 ng µl^−1^ of RNA for 10 min before addition of 50 µM of H_2_S. Upon H_2_S addition, recording was started and dissolution was acquired for 20 min. In a separate experiment, the yield of PSSH was estimated by monitoring TNB absorbance at 412 nm, following addition of H_2_S. A yield of ~95% was obtained, in agreement with our previous report using the same protocol^4,55^. The dissolution experiment was performed in three separate reconstitutions. Images were acquired using an Evident CSU-W1 spinning disk confocal microscope equipped with a Teledyne Prime BSI sCMOS camera with a ×60 silicone objective lens.

### G3BP2 WT and C73S mutant plasmid construction

The pDEST-CMV-N-EGFP-wtG3BP2 plasmid was built using the gateway cloning method^95^. The human G3BP2 sequence from the pENTR4_G3BP2 plasmid was transferred into the pDEST-CMV-N-EGFP plasmid using Gateway LR Clonase following the manufacturer’s instructions. Both plasmids were obtained through Addgene. pENTR4_G3BP2 was a gift from T. Tuschl (Addgene plasmid no. 127105). pDEST-CMV-N-EGFP was a gift from R. Ketteler (Addgene plasmid no. 122842).

The C73S point mutation was created following an established protocol^95^. Plasmids were first tested for the mutation using PCR primers that allowed the elongation of a smaller fragment when the mutation was present (forward primer: GACTTCAAGGAGGACGG; reverse primer: GTCTCAGCGACGCTG; reverse middle primer: ACATGACGAATTTTAGTATGACT). Positive clones were then sent for sequencing for further validation. Both pDEST-CMV-N-EGFP-wtG3BP2 and pDEST-CMV-N-EGFP-C73S-G3BP2 plasmids were amplified and extracted using a PureLink HiPure Plasmid Maxiprep Kit (Invitrogen, K210007) before use.

### Plasmid transfection and G3BP2 live cell microscopy

*Cse*^−/−^ and WT MEFs were transfected with pDEST-CMV-N-EGFP-C73S-G3BP2 or pDEST-CMV-N-EGFP-wtG3BP2 using a reverse transfection protocol. Briefly, the transfection solution was prepared by mixing 1 part of plasmid DNA (in micrograms) with 3 parts of TransIT-2020 Transfection Reagent (Mirius, MIR5400) in Opti-MEM I Reduced Serum Medium (Gibco, cat. no. 31985062) and incubated for 30 min. Cells were trypsinized for 3 min, followed by quenching with DMEM supplemented with 10% fetal bovine serum. After incubation, the transfection solution was added into a glass-bottomed µ-slide with 8 wells at 50 ng of DNA per well (IBIDI, cat. no. 80807). Then, 15,000 cells were seeded in each well on top of the transfection reagent, followed by incubation for 24 h. The next day, the medium was changed 2 h before the start of the experiment to allow cell recovery from transfection stress.

Live cell microscopy was carried out using a Leica SP8 LIGHTNING or Nikon AX R NSPARC laser scanning confocal microscope with a ×63 water or oil objective, respectively. Environmental control (37 °C, 5% CO_2_) and adaptive focus control were used.

### G3BP2 live cell microscopy

For measurements of SG formation, cells were treated with 5 µg ml^−1^ Hoechst 33342 for 5 min. Medium containing Hoechst 33342 was washed 2 times and replaced with FluoroBrite DMEM supplemented with 2 mM l-glutamine, 1% penicillin–streptomycin and 10% fetal bovine serum. Cells (WT or *Cse*^−/−^ MEFs) transformed with WT G3BP2 or (mutant) C73S-G3BP2 were treated with 500 µM sodium metaarsenite (NaAsO_2_), and one image was taken every 16 s for 20 min. The number and size of SGs were quantified using Fiji.

#### Live H_2_S treatment

For in cellulo H_2_S dissolution of G3BP2 condensates, WT MEFs were treated for 15 min with 500 µM NaAsO_2_ to allow formation of G3BP2 condensates. Cells were then recorded every 16 s, and Na_2_S was injected at a final concentration of 100 µM. After the condensate had reformed, a second injection of H_2_S at the same concentration was performed. The number and size of SGs were quantified using Fiji.

#### In cellulo FRAP

WT and *Cse*^−/−^ MEFs were treated with 500 µM NaAsO_2_ as previously described. After 15 min, photobleaching was induced using 4 pulses at 100% laser power, and recovery was imaged every 3 s. FRAP analysis was performed using Fiji.

### Immunofluorescence of G3BP2 under NaAsO_2_ treatment

MEF, STHdh cells or human fibroblasts were split in a 35-mm glass-bottomed µ-dish (IBIDI, cat. no. 81158). For all experiments, cells were treated with 500 µM of sodium(meta)arsenite (NaAsO_2_). GYY4737 was used at 200 µM during the 1-h recovery. For ET experiments, WT or *Cse*^−/−^ MEFs were pretreated with 400 µM ET for 2 h before treatment with 500 µM NaAsO_2_. ET was omitted during stress induction to prevent cross-reaction with arsenite. The medium was supplemented again with ET during recovery.

#### Fixation and immunofluorescence

Before fixation, cells were washed twice with warm PBS and fixed with 2% PFA for 10 min, followed by 10 min with 4% PFA. Cells were washed three times with cold PBS to remove PFA. For immunofluorescence, the permeabilization step was performed in PBS 0.1% Triton X-100 for 20 min. Cells were incubated for 1 h in blocking buffer (1% BSA, 22.52 mg ml^−1^ glycine, 0.1% Tween in PBS) followed by 2-h incubation at 37 °C with anti-G3BP2, (1:600; Proteintech, 16276-1-AP). Cells were then washed and incubated with secondary antibody (mouse anti-rabbit IgG-CFL 647; Santa Cruz Biotechnology, sc-516251) for 2 h at 37 °C. Cells were washed again and stained with 4’,6-Diamidin-2-phenylindol dihydrochloride (DAPI; Thermo Fisher Scientific) before being imaged, according to the manufacturer’s protocol.

#### Imaging

Imaging was performed using either a Leica SP8 LIGHTNING confocal microscope with a ×63 oil objective and a resolution of 2,048 × 2,048 or an Evident CSU-W1 spinning disk confocal microscope equipped with a Teledyne Prime BSI sCMOS camera with a 100 × 1.45 NA oil UPLXAPO100XO objective lens.

### Immunofluorescence for colocalization of SG proteins

MEFs were seeded into eight-well glass-bottomed µ-slides (IBIDI, cat. no. 80807) and treated with NaAsO_2_ for the indicated time periods, followed by washing and fixation, permeabilization and blocking as previously described. Samples were incubated with anti-G3BP2, (1:600; Proteintech, 16276-1-AP) for 2-h incubation at 37 °C, followed by washing and incubation for 2 h at 37 °C with the following secondary antibodies: mouse anti-rabbit IgG-CFL 488 (Santa Cruz Biotechnology, sc-516248), mouse anti-TDP-43-AF790 (1:250; Santa Cruz Biotechnology, sc-376311), mouse anti-eIF4G-AF594 (1:250; Santa Cruz Biotechnology; sc-133155) and mouse anti-TIA-1/TIAR(D-9)AF-647 (1:250; Santa Cruz Biotechnology, sc-48371). DAPI (Thermo Fisher Scientific) staining was performed before imaging, according to the manufacturer’s protocol. Images were acquired on a Leica STELLARIS confocal microscope with a White Light Laser, using a HC PL APO CS2 ×100/1.40 oil objective lens and a resolution of 1,024 × 1,024.

### Image analysis

All images were converted to TIFs using Fiji software. TIFs where then analyzed using a Python script developed in-house. For datasets containing *Z* stacks, average intensity projection was used to produce a *Z*-projected image used for further analysis. Cell segmentation was performed using the Cellpose deep-learning framework (cyto3 model). To improve segmentation, we used a composite image of DAPI signal and protein spread in the cytoplasm. Before segmentation, images were downscaled to 256 × 256 resolution to match Cellpose requirements. The resulting masks were then upscaled to the original resolution using nearest-neighbor interpolation, producing per-cell masks that were used throughout the quantification pipeline.

Granule detection was performed independently for each protein if applicable, using an automated multistep procedure that included: (1) white top-hat filtering (disk radius = 5 px); (2) global thresholding using the Otsu threshold method, with weighting by a constant factor to suppress weak background puncta; (3) particle analysis performed on the binary mask using morphological size filtering followed by connected-component labeling (scikit-image). This approach ensured robust granule detection across different intensities and imaging conditions without manual parameter tuning. To determine the number and size of granules per cell, overlapping granules with the segmented cells were measured, and three metrics were computed: the granule count, the total granule area, and the granule density per cell (total granule area/cell area).

For the granule colocalization analysis, cell segmentation and thresholding were performed as described above. A condensate restricted colocalization analysis was performed to avoid misleading whole-cell correlations. All colocalization metrics were computed only within granule pixels for each pair of proteins. For each segmented cell and pair of proteins, an object-based Mander’s coefficient was calculated to quantify the proportion of granules spatially co-occurring, independent of the pixel intensity.

### Western blot detection of global and G3BP2 PSSH and PSOH levels under NaAsO_2_ treatment

WT MEFs were treated with 500 µM of NaAsO_2_ or left untreated for the indicated times. Cells were lysed in HEN buffer supplemented with 5 mM NBF-Cl for persulfidation or 0.5 mM BTD for sulfenylation. Sample preparation and enrichment steps for both modifications were performed as previously described except for the elution step. Briefly, after incubation and washing steps, Pierce High Capacity NeutrAvidin Agarose beads were resuspended in 50 mM HEPES, 1× Laemmli buffer, and boiled at 95 °C for 10 min to release enriched proteins. Part of each sample was taken before enrichment to be used as a loading control. Both loading controls and enriched fractions were then run on a 10% acrylamide gel and transferred to a nitrocellulose membrane. Streptavidin conjugated with DyLight 633 was used to detect global persulfidation and sulfenylation levels. Anti-G3BP2 antibody (1:4,000) and secondary anti-rabbit IgG H&L Alexa Fluor 680 (1:20,000) and anti-GAPDH-AF488 (Sigma-Aldrich, MAB374-AF488; 1:2,000) were used for immunoblotting.

### Thermal proteome profiling of WT and *Cse*^−/−^ MEFs under H_2_O_2_ treatment

WT and *Cse*^−/−^ MEFs were trypsinized and washed three times in cold serum-free DMEM. After the last centrifugation, cells were resuspended in 1.2 ml of DMEM supplemented with 10% fetal calf serum and treated for 60 min with 500 µM H_2_O_2_ on ice. After 60 min, equal numbers of cells were aliquoted into different tubes and gently centrifuged. Cell pellets were resuspended in 20 µl serum-free media and heated at 25 °C, 47 °C or 57 °C for 3 min. Cells were brought back to RT for 3 min and lysed with freeze–thaw cycles. Extracted proteins were subjected to ultracentrifugation at 100,000*g* for 20 min at 4 °C. The volume corresponding to 50 µg in the cells exposed to 25 °C was used for all samples. Proteins were subsequently denatured using 3 M Gu-HCl for reduction and alkylation steps with 500 µM DTT and 5 mM IAM for 30 min at 37 °C, respectively, then subjected to tryptic digestion. Peptides were desalted as previously described. Eluates were evaporated until dry and resuspended in 100 mM triethylammonium bicarbonate for TMT labeling. Samples were labeled using a TMTpro 16-plex Label Reagent Set; they were then combined, desalted and dried as previously described and subjected to LC/MS analysis with an Orbitrap Eclipse Tribrid mass spectrometer via an EASY-spray (Thermo Fisher Scientific).

Data were evaluated with FragPipe software^96^ using 10 ppm for precursor mass tolerance, 0.02 Da for fragment mass tolerance, specific tryptic digest and a maximum of 3 missed cleavages. Carbamidomethylation (+57.021464 Da), N-terminal acetylation (+42.010565 Da) and methionine oxidation (+15.994915 Da) were added as variable modifications. TMTpro (+304.207146 Da) was added as a fixed modification on K and peptide N-terminal; PSM was performed, and proteins were filtered at FDR 1%. TMT quantification was performed at the MS2 level.

### Brain sectioning and Gallyas staining

Brains were extracted and fixed in 4% paraformaldehyde solution for 24 h at 4 °C, dehydrated in ethanol (30–100%) and cleared with xylene. After being embedded in Histowax (Histolab Product AB), tissue blocs were sectioned to 5-μm thickness on a rotary microtome (RM 2125RT Leica Microsystems). Gallyas silver staining (Morphisto) was performed following the manufacturer’s protocol.

### Statistics and reproducibility

All cell and animal experiments were performed with at least three biological replicates unless stated otherwise. Male mice were randomly chosen when collected at different ages. Biological replicates were defined as independent experiments performed on separate cell cultures or individual animals. No statistical method was used to predetermine sample size. No data were excluded from the analyses. Phase separation assays were performed with at least two reconstitutions from two independent protein purifications and several measurements per reconstitution. For proteomic data analysis, two-sided Welch’s *t*-test and permutation-based FDR were used for *P* values; an adjusted *P* value of <0.05 was considered to indicate significance. The DAVID web platform was used for pathway enrichment analysis, with statistical significance assessed using the EASE score, a modified one-sided Fisher’s exact test. *P* values were corrected for multiple comparisons using the Benjamini–Hochberg method. All statistical tests were performed two-sided. Statistical analysis was performed in Perseus (1.6.15.0) for proteomic analysis and GraphPad Prism 11 for other analyses. *P* values are reported to the numerical precision of the software; values below 1.0 × 10^−^^15^ are indicated as *P* < 1.0 × 10^−^^15^. The experiments were not randomized, and the investigators were not blinded to allocation during experiments or outcome assessment.

### Reporting summary

Further information on research design is available in the Nature Portfolio Reporting Summary linked to this article.

## Online content

Any methods, additional references, Nature Portfolio reporting summaries, source data, extended data, supplementary information, acknowledgements, peer review information; details of author contributions and competing interests; and statements of data and code availability are available at 10.1038/s41594-026-01857-w.

## Supplementary information

Supplementary Information Supplementary text, references, Figs. 1–10, uncropped gels and blots.

Reporting Summary

Peer Review File

Supplementary Table Supplementary tables containing results of proteomics data analysis.

Supplementary Video 1 Live imaging of mScarlet–synapsin 1 and pHluorin in the synapses of activated primary neurons without pretreatment or pretreated with GYY4137 or propargylglycine and aminooxyacetic acid.

Supplementary Video 2 Formation of WT and C73S mutant GFP–G3BP2 condensates in WT and *Cse*^*−/−*^ MEFs.

Supplementary Video 3 GFP–G3BP2 FRAP in WT and *Cse*^*−/−*^ MEFs.

Supplementary Video 4 In vitro dissolution of G3BP2-TNB condensates upon H_2_S addition.

Supplementary Video 5 In cellulo dissolution of WT GFP–G3BP2 condensates using H_2_S in WT MEFs.

Supplementary Video 6 In cellulo dissolution of C73S mutant GFP–G3BP2 condensates using H_2_S in WT MEFs.

Supplementary Data Source data for Supplementary figures.

## Source data

Source Data Fig. 2 Statistical source data.

Source Data Fig. 3 Statistical source data.

Source Data Fig. 4 Statistical source data.

Source Data Fig. 5 Statistical source data.

Source Data Fig. 5 Unprocessed western blots.

Source Data Fig. 6 Statistical source data.

Source Data Fig. 6 Unprocessed western blots.

Source Data Extended Data Fig. 2 Statistical source data.

Source Data Extended Data Fig. 4 Statistical source data.

Source Data Extended Data Fig. 4 Unprocessed western blots.

Source Data Extended Data Fig. 5 Statistical source data.

Source Data Extended Data Fig. 6 Statistical source data.

Source Data Extended Data Fig. 6 Unprocessed western blots.

Source Data Extended Data Fig. 7 Statistical source data.

Source Data Extended Data Fig. 8 Statistical source data.

Source Data Extended Data Fig. 9 Statistical source data.

Source Data Extended Data Fig. 9 Unprocessed western blots.

Source Data Extended Data Fig. 10 Statistical source data.

## Data Availability

Data and materials can be obtained from the corresponding authors upon request. All proteomic data are available through PRIDE^97^ under accession codes PXD045785 (sulfenylome and total proteome); PXD045806 (persulfidation and sulfonylation); and PXD045820 (TPP of WT and *Cse*^−/−^ MEFs upon H_2_O_2_ stress). Gallyas silver staining images of sections from 18-month-old WT and *Cse*^−/−^ mice are available via BioImage Archive^98^ (10.6019/S-BIAD1604). Pathological phospho-tau tangle and amyloid-β deposit formation in WT and CSE-deficient mouse brain are available via Figshare at 10.6084/m9.figshare.32817593(ref. ^65^). An online platform for interactive data exploration is available at https://www.agingredox.org/. Source data are provided with this paper.
