# Supplementary material for: Protein thiol alterations drive pathologic liquid–liquid phase separation in the aging brain
Source: Nat Struct Mol Biol. 2026 Jul 30;33(8):1252–65. doi: 10.1038/s41594-026-01857-w (PMC13468144; doi:10.1038/s41594-026-01857-w)
Supplement: Supplementary file 1 [file 41594_2026_1857_MOESM16_ESM.pdf]

Fig. 5i

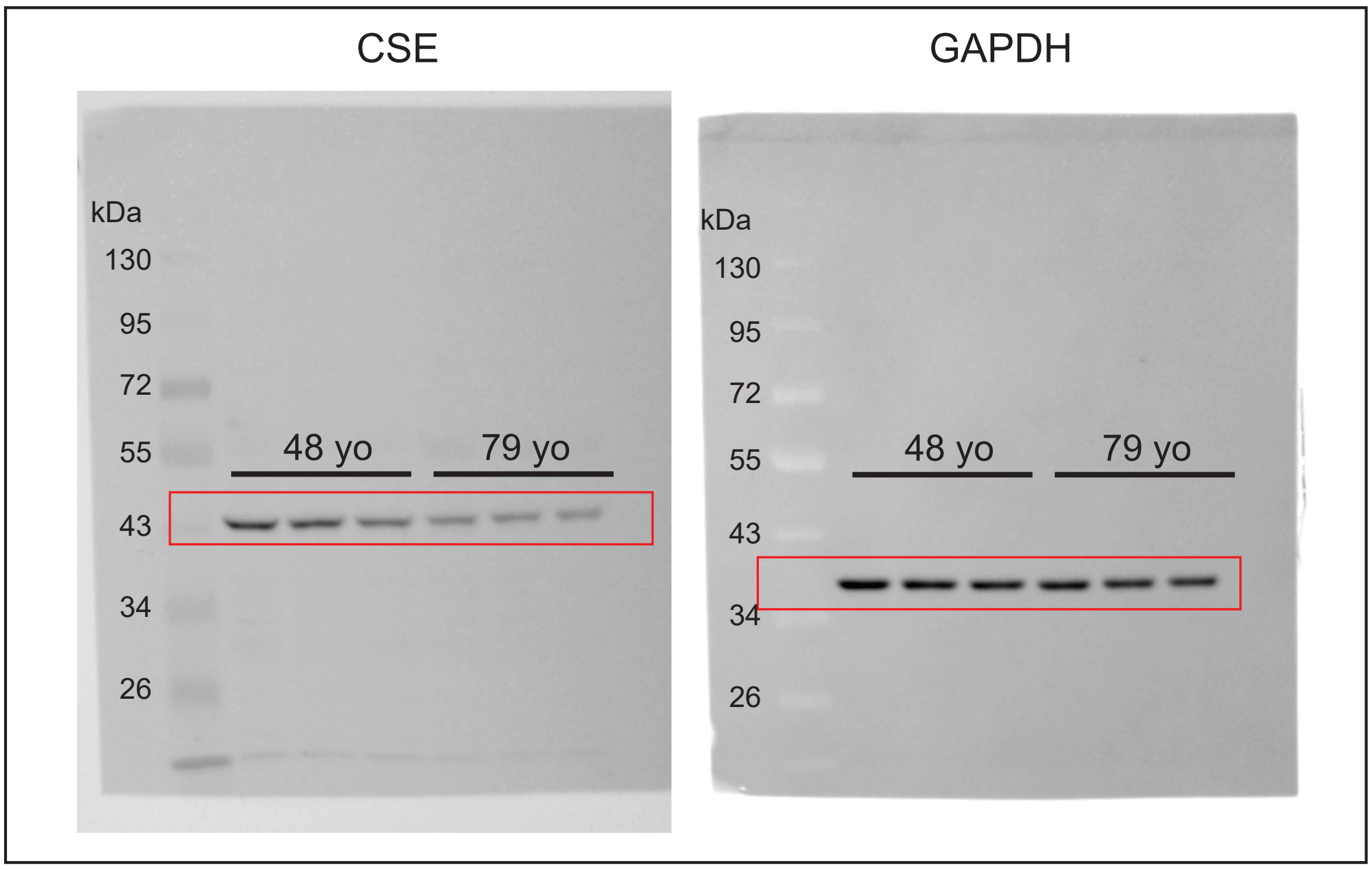
CSE
GAPDH
kDa
130
95
72
55
43
34
26
48 yo
79 yo
kDa
130
95
72
55
43
34
26
48 yo
79 yo
