# Supplementary material for: Protein thiol alterations drive pathologic liquid–liquid phase separation in the aging brain
Source: Nat Struct Mol Biol. 2026 Jul 30;33(8):1252–65. doi: 10.1038/s41594-026-01857-w (PMC13468144; doi:10.1038/s41594-026-01857-w)
Supplement: Supplementary file 2 [file 41594_2026_1857_MOESM18_ESM.pdf]

Fig. 6b

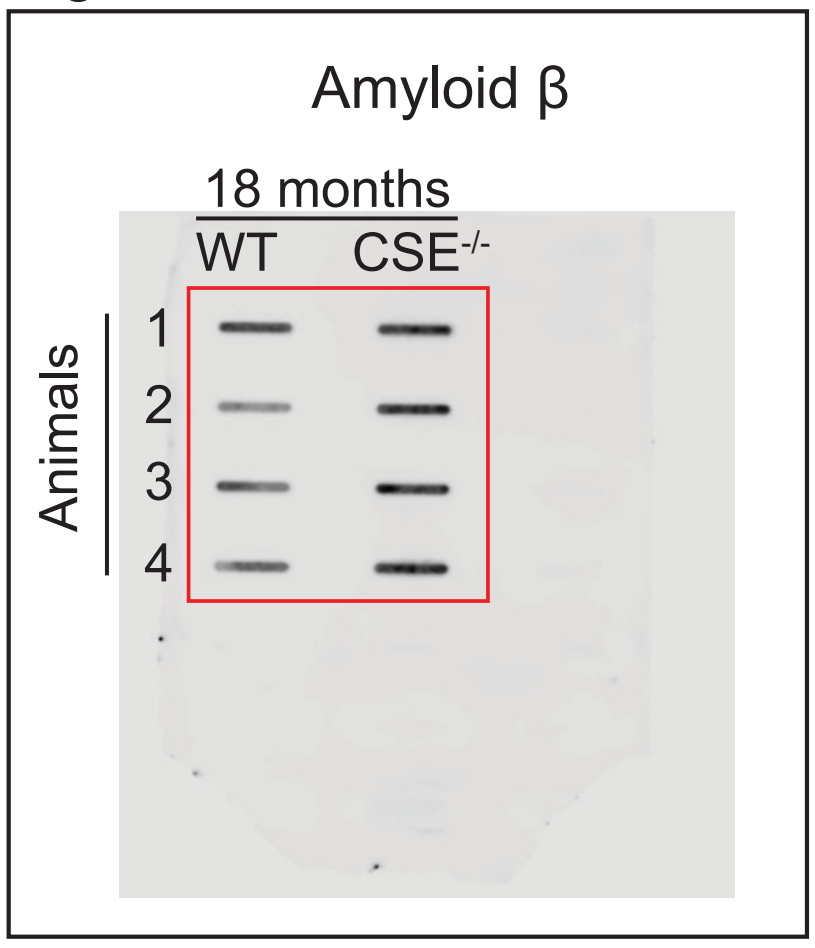
Amyloid β
18 months
WT
CSE$^{-/-}$
Animals
1
2
3
4

Fig. 6c

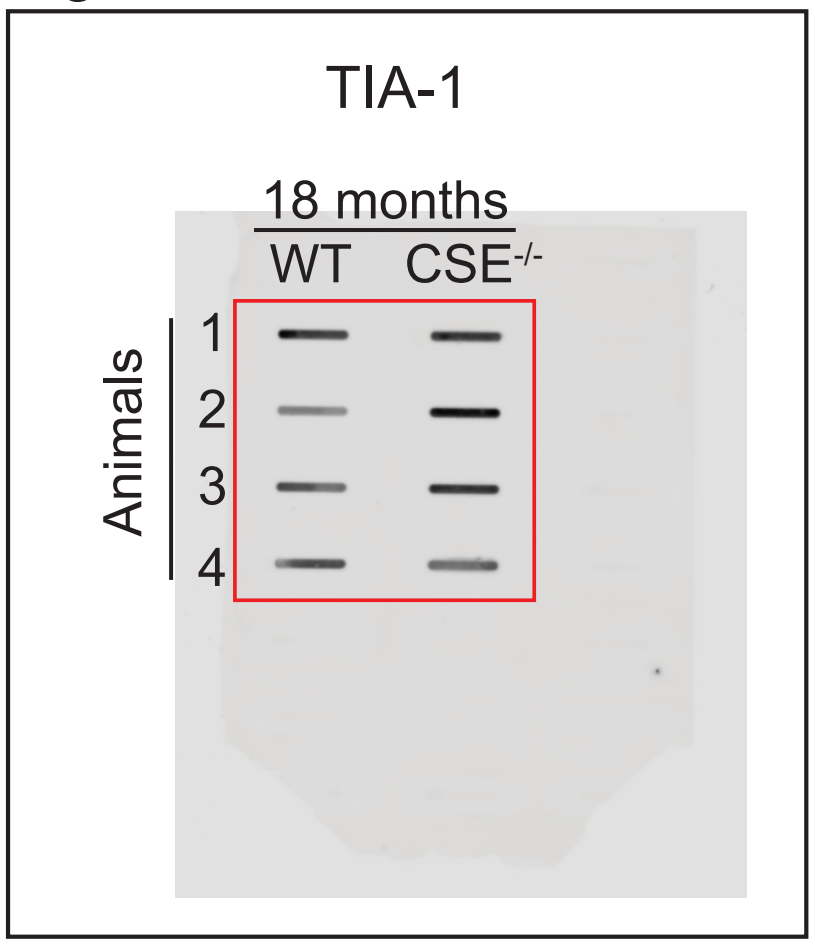
TIA-1
18 months
WT
CSE$^{-/-}$
Animals
1
2
3
4
