# Supplementary material for: Protein thiol alterations drive pathologic liquid–liquid phase separation in the aging brain
Source: Nat Struct Mol Biol. 2026 Jul 30;33(8):1252–65. doi: 10.1038/s41594-026-01857-w (PMC13468144; doi:10.1038/s41594-026-01857-w)
Supplement: Supplementary file 3 [file 41594_2026_1857_MOESM1_ESM.pdf]

nature structural & molecular biology

Supplementary information

https://doi.org/10.1038/s41594-026-01857-w

Protein thiol alterations drive pathologic liquid–liquid phase separation in the aging brain

In the format provided by the authors and unedited

Contents

Supplementary text.. 2

Supplementary References:.. 4

Supplementary Fig. 1. Total proteome changes in aging mice... 6

Supplementary Fig. 2. Changes in mRNA levels of H_2S producing enzymes in mouse and human brain during aging.. 7

Supplementary Fig. 3. Changes in CSE substrate levels in olfactory bulb and cerebral cortex, based on aging mouse metabolome atlas.. 8

Supplementary Fig. 4. LLPS properties of IAM-modified synapsin-1 are not affected by the protocol to generate PSSH or PSO_xH... 9

Supplementary Fig. 5. Peptide fragmentation analysis of a cysteine-containing G3BP2 peptide.. 10

Supplementary Fig. 6. Colocalization of different SG proteins in WT and *Cse*$^{-/-}$ MEF exposed to arsenite stress.. 12

Supplementary Fig. 7. Age- and CSE-dependent accumulation of aggregated stress-granule proteins.. 13

Supplementary Fig. 8. Old *Cse*$^{-/-}$ mice exhibit multiple Gallyas silver–stained neuritic-like plaques.. 14

Supplementary Fig. 9. 10-week-old Cse$^{-/-}$ mice do not exhibit positive Gallyas silver–stained neuritic-like plaques.. 15

Supplementary Fig. 10. Local microenvironments of redox-active cysteines in G3BP2 and synapsin-1... 16

Uncropped blot from supplementary figures... 17

Supplementary text

Thiol oxidation to sulfenic acid (PSOH) represents only the initial step in a series of oxidative reactions that can occur within cells.[1–3] PSOH can react with other thiols to form disulfides (PSSP), either with protein thiols or with low–molecular weight thiols such as cysteine or glutathione. Alternatively, PSOH can react with H_2S to generate persulfides (PSSH)[4,5] or, in some cases, with cyanide to form thiocyanates (PSCN).[6] Under conditions of sustained oxidative stress, PSOH can undergo further oxidation to sulfinic (PSO_2H) and sulfonic (PSO_3H) acids.[3,7]

Persulfides are likewise highly reactive species.[4,8–12] The presence of both a nucleophilic and an electrophilic sulfur atom renders them intrinsically unstable, as they can react with themselves to form trisulfides (PSSSP). PSSH can also react with other thiols to form disulfides or with ROS, acting as stronger nucleophiles than their corresponding thiols and reacting with ROS at least an order of magnitude faster, to form S-sulfonates ($PSSO_3H$).[5]

The multiple and competing reaction pathways available to PSOH and PSSH necessitate the use of chemoselective methods to capture these modifications. At the same time, this chemical complexity means that any labelling and quantification strategy can only report their steady-state levels within cells, rather than their full dynamic range.

The significance of chemoselective labelling and enrichment of each cysteine PTM is highlighted by the limitations of approaches using classical thiol-blocking reagents, such as iodoacetamide or N-ethylmaleimide-based probes. These reagents often lack specificity because they label not only PSH and PSSH but also PSOH, resulting in adducts that are cleavable by DTT and TCEP.[13] Consequently, methods designed to detect PSSH[14,15] or “global thiol oxidation”[16] cannot differentiate between PSSH and PSOH.[17] Thus, it is possible to observe “no change” in thiol oxidation while in fact having significant shift from PSOH to PSSH (based on scheme in Fig 1a). Moreover, recent studies have shown that the disulfide adducts formed between commonly used thiol-blocking reagents and PSSH are unstable and can undergo a chemical transformation from disulfide to thioether. This finding casts doubt on the validity of all site-specific PSSH quantifications conducted to date.[18]

We therefore focused on enriching labelled proteins and then digesting them, to ensure much larger coverage of proteins affected by persulfidation and sulfenylation, as indeed demonstrated in Fig. 1e,j. This comes with a limitation that specific cysteine sites are not always detectable and instead have to be addressed on a protein-by-protein basis. However, our general approaches were not intended to quantify the percentage of occurrence of specific cysteine PTMs. Instead, they provide a fold-change comparison to the control sample. Although some studies use the ratio of (modified cysteine)/(TCEP-reduced cysteine) as a measure of site occupancy,[16] this is in strict chemical terms an incorrect approach. It is impossible to use a single method to selectively label all modifications simultaneously, and achieving a fully reduced cysteine proteome is not technically feasible. Our data with IAM blocking, or NBF-Cl blocking suggest that a simple thiol blocking already results in detection of PSX, PSOX, PSSX, PSO_3H, where X is a blocking reagent, and in case of NBF-Cl in peptides containing K and R also labelled with NBF. Furthermore, recent study shows that even 90%-reduced proteomes can house vast numbers of unique, functionally diverse oxiforms.[19]

Namely, modifications such as PSOH, PSSH, S-nitrosation and disulfides are cleavable with reducing agents such as DTT or TCEP.[20] However, cysteine is a site of plethora of PTMs. For example, sulfinylation and sulfonlyation, are irreversible and cannot be reduced back.[21] There are reports of cysteine palmitoylation[22], prenylation,[23] and acetylation,[24] all of which require harsh chemical cleavage with hydroxylamine. Furthermore, cysteines are targets of variety of naturally occurring electrophiles such as HNE,[25,26] itaconate,[27] fumarate[28] etc. Recent report has suggested cyanylation as a new and widespread cysteine PTM, while Pappenheim et al, 2022 reported widespread occurrence of covalent lysine-cysteine redox switches.[29] Therefore, the relative change between the conditions remains the only reliable way of reporting cysteine PTMs. In fact, even for the phosphoproteome, where almost full de-phosphorylation could be achieved, it is very difficult to calculate and estimate the occupancy of each S, T or Y.[30,31] Recent studies have challenged the importance of determining site occupancy in assigning the biological role of specific PTMs, revealing that the average site occupancy for ubiquitination is only 0.005%,[32] while acetylation occupies 0.1% of lysine residues,[33] despite both modifications being essential for cellular function. Having observed that many proteins undergoing age-induced thiol

remodelling also undergo LLPS highlights the importance of the local concentration of modified species, rather than global assessments of site occupancy, which may remain low.

Supplementary References:

1. Gupta, V. & Carroll, K. S. Sulfenic acid chemistry, detection and cellular lifetime. *Biochim Biophys Acta Gen Subj* 1840, 847–875 (2014).
2. Shi, Y. & Carroll, K. S. Activity-Based Sensing for Site-Specific Proteomic Analysis of Cysteine Oxidation. *Acc Chem Res* 53, 20–31 (2020).
3. Paulsen, C. E. & Carroll, K. S. Cysteine-mediated redox signaling: Chemistry, biology, and tools for discovery. *Chem Rev* 113, 4633–4679 (2013).
4. Cuevasanta, E. *et al.* Reaction of Hydrogen Sulfide with Disulfide and Sulfenic Acid to Form the Strongly Nucleophilic Persulfide. *Journal of Biological Chemistry* 290, 26866–26880 (2015).
5. Zivanovic, J. *et al.* Selective Persulfide Detection Reveals Evolutionarily Conserved Antiaging Effects of S-Sulfhydration. *Cell Metab* 30, 1152-1170.e13 (2019).
6. Zuhra, K. *et al.* Regulation of mammalian cellular metabolism by endogenous cyanide production. *Nat Metab* https://doi.org/10.1038/s42255-025-01225-w (2025) doi:10.1038/s42255-025-01225-w.
7. Akter, S. *et al.* Chemical proteomics reveals new targets of cysteine sulfinic acid reductase. *Nat Chem Biol* 14, 995–1004 (2018).
8. Yadav, P. K. *et al.* Biosynthesis and Reactivity of Cysteine Persulfides in Signaling. *J Am Chem Soc* 138, 289–299 (2016).
9. Filipovic, M. R., Zivanovic, J., Alvarez, B. & Banerjee, R. Chemical Biology of H2S Signaling through Persulfidation. *Chem Rev* 118, 1253–1337 (2018).
10. Vignane, T. & Filipovic, M. R. Emerging Chemical Biology of Protein Persulfidation. *Antioxid Redox Signal* 39, 19–39 (2023).
11. Cuevasanta, E. *et al.* Kinetics of formation and reactivity of the persulfide in the one-cysteine peroxiredoxin from Mycobacterium tuberculosis. *Journal of Biological Chemistry* 294, 13593–13605 (2019).
12. Benchoam, D. *et al.* Acidity and nucleophilic reactivity of glutathione persulfide. *Journal of Biological Chemistry* 295, 15466–15481 (2020).
13. Reisz, J. A., Bechtold, E., King, S. B., Poole, L. B. & Furdui, C. M. Thiol-blocking electrophiles interfere with labeling and detection of protein sulfenic acids. *FEBS Journal* 280, 6150–6161 (2013).
14. Bithi, N. *et al.* Dietary restriction transforms the mammalian protein persulfidome in a tissue-specific and cystathionine γ-lyase-dependent manner. *Nat Commun* 12, (2021).
15. Gao, X.-H. *et al.* Quantitative H2S-mediated protein sulfhydration reveals metabolic reprogramming during the integrated stress response. *Elife* 4, e10067 (2015).
16. Xiao, H. *et al.* A Quantitative Tissue-Specific Landscape of Protein Redox Regulation during Aging. *Cell* 180, 968-983.e24 (2020).
17. Vignane, T. & Filipovic, M. R. Emerging Chemical Biology of Protein Persulfidation. *Antioxid Redox Signal* 39, 19–39 (2023).

18. Schilling, D. *et al.* Commonly Used Alkylating Agents Limit Persulfide Detection by Converting Protein Persulfides into Thioethers. *Angewandte Chemie - International Edition* 61, 1–13 (2022).
19. Cobley, J. N., Chatzinikolaou, P. N. & Schmidt, C. A. The nonlinear cysteine redox dynamics in the i-space: A proteoform-centric theory of redox regulation. *Redox Biol* 81, 103523 (2025).
20. Paulsen, C. E. & Carroll, K. S. Cysteine-mediated redox signaling: Chemistry, biology, and tools for discovery. *Chem Rev* 113, 4633–4679 (2013).
21. Akter, S. *et al.* Chemical proteomics reveals new targets of cysteine sulfinic acid reductase. *Nat Chem Biol* 14, 995–1004 (2018).
22. Martin, B. R., Wang, C., Adibekian, A., Tully, S. E. & Cravatt, B. F. Global profiling of dynamic protein palmitoylation. *Nat Methods* 9, 84–89 (2012).
23. Storck, E. M. *et al.* Dual chemical probes enable quantitative system-wide analysis of protein prenylation and prenylation dynamics. *Nat Chem* 11, 552–561 (2019).
24. James, A. M., Smith, A. C., Smith, C. L., Robinson, A. J. & Murphy, M. P. Proximal Cysteines that Enhance Lysine N-Acetylation of Cytosolic Proteins in Mice Are Less Conserved in Longer-Living Species. *Cell Rep* 24, 1445–1455 (2018).
25. Parvez, S., Long, M. J. C., Poganik, J. R. & Aye, Y. Redox Signaling by Reactive Electrophiles and Oxidants. *Chem Rev* 118, 8798–8888 (2018).
26. Liu, J. *et al.* Organ-specific electrophile responsivity mapping in live C. elegans. *Cell* 187, 7450-7469.e29 (2024).
27. Qin, W. *et al.* S-glycosylation-based cysteine profiling reveals regulation of glycolysis by itaconate. *Nat Chem Biol* 15, 983–991 (2019).
28. Blewett, M. M. *et al.* Chemical proteomic map of dimethyl fumarate–sensitive cysteines in primary human T cells. *Sci Signal* 9, (2016).
29. Rabe von Pappenheim, F. *et al.* Widespread occurrence of covalent lysine–cysteine redox switches in proteins. *Nat Chem Biol* 18, 368–375 (2022).
30. Wu, R. *et al.* A large-scale method to measure absolute protein phosphorylation stoichiometries. *Nat Methods* 8, 677–683 (2011).
31. Dickhut, C., Feldmann, I., Lambert, J. & Zahedi, R. P. Impact of Digestion Conditions on Phosphoproteomics. *J Proteome Res* 13, 2761–2770 (2014).
32. Prus, G., Satpathy, S., Weinert, B. T., Narita, T. & Choudhary, C. Global, site-resolved analysis of ubiquitylation occupancy and turnover rate reveals systems properties. *Cell* 187, 2875-2892.e21 (2024).
33. Hansen, B. K. *et al.* Analysis of human acetylation stoichiometry defines mechanistic constraints on protein regulation. *Nat Commun* 10, 1055 (2019).
34. Schaum, N. *et al.* Ageing hallmarks exhibit organ-specific temporal signatures. *Nature* 583, 596–602 (2020).
35. Trabzuni, D. *et al.* Widespread sex differences in gene expression and splicing in the adult human brain. *Nat Commun* 4, 2771 (2013).
36. Ding, J. *et al.* A metabolome atlas of the aging mouse brain. *Nat Commun* 12, 6021 (2021).

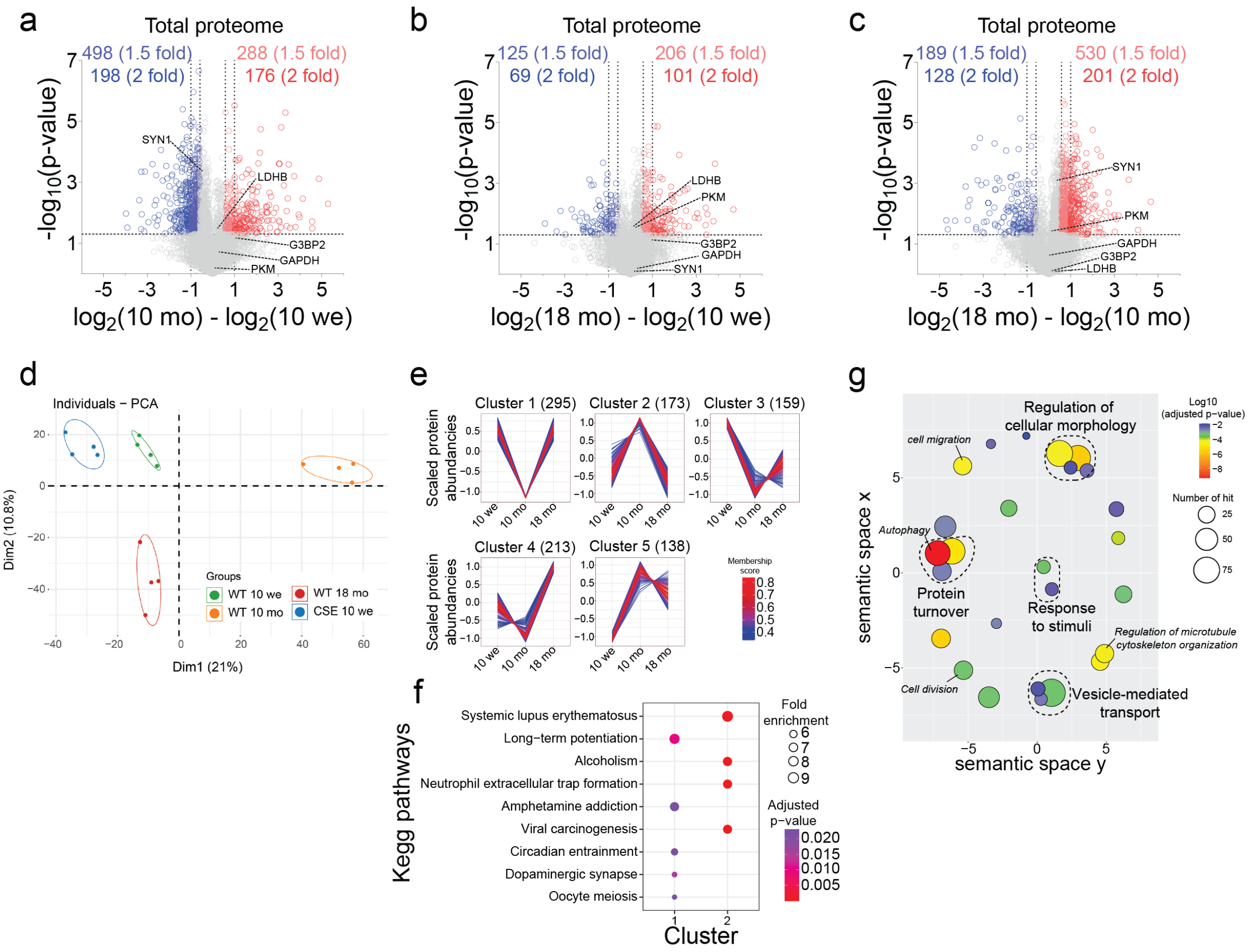

Supplementary Fig. 1. Total proteome changes in aging mice. (**a-c**) Volcano plot analysis of total proteome changes (Welch's t-test, n = 4 animals) in (**a**) 10-month-old mice vs. 10-week-old mice (**b**) 18-month-old mice vs. 10-week-old mice and (**c**) 18-month-old mice vs. 10-month-old mice (**d**) Principal component analysis underscoring the reproducibility of total proteome measurements across all replicates. (**e**) Hierarchical cluster analysis of proteins displaying significant changes (n = 4 animals, Welch's t-test with FDR adjusted p value ≤ 0.05, fold change > 30 %) at least at one time point during aging and KEGG pathway enrichment analysis of protein clusters. (**f**) Only clusters 1 and 2 display significant enrichment in KEGG pathway terms (Benjamini corrected p-value ≤ 0.05). (**g**) GO term (biological process) enrichment analysis of the proteins displaying age-related changes (n = 4 animals, Welch's t-test with FDR adjusted p value ≤ 0.05, fold change > 30 %). REVIGO was used to plot the enrichment analysis performed in DAVID. Significant GO terms passed the Benjamini adjusted p-value threshold of 0.01. Circle dimensions denote the protein count within specific GO terms, while color gradients represent the degree of significance.

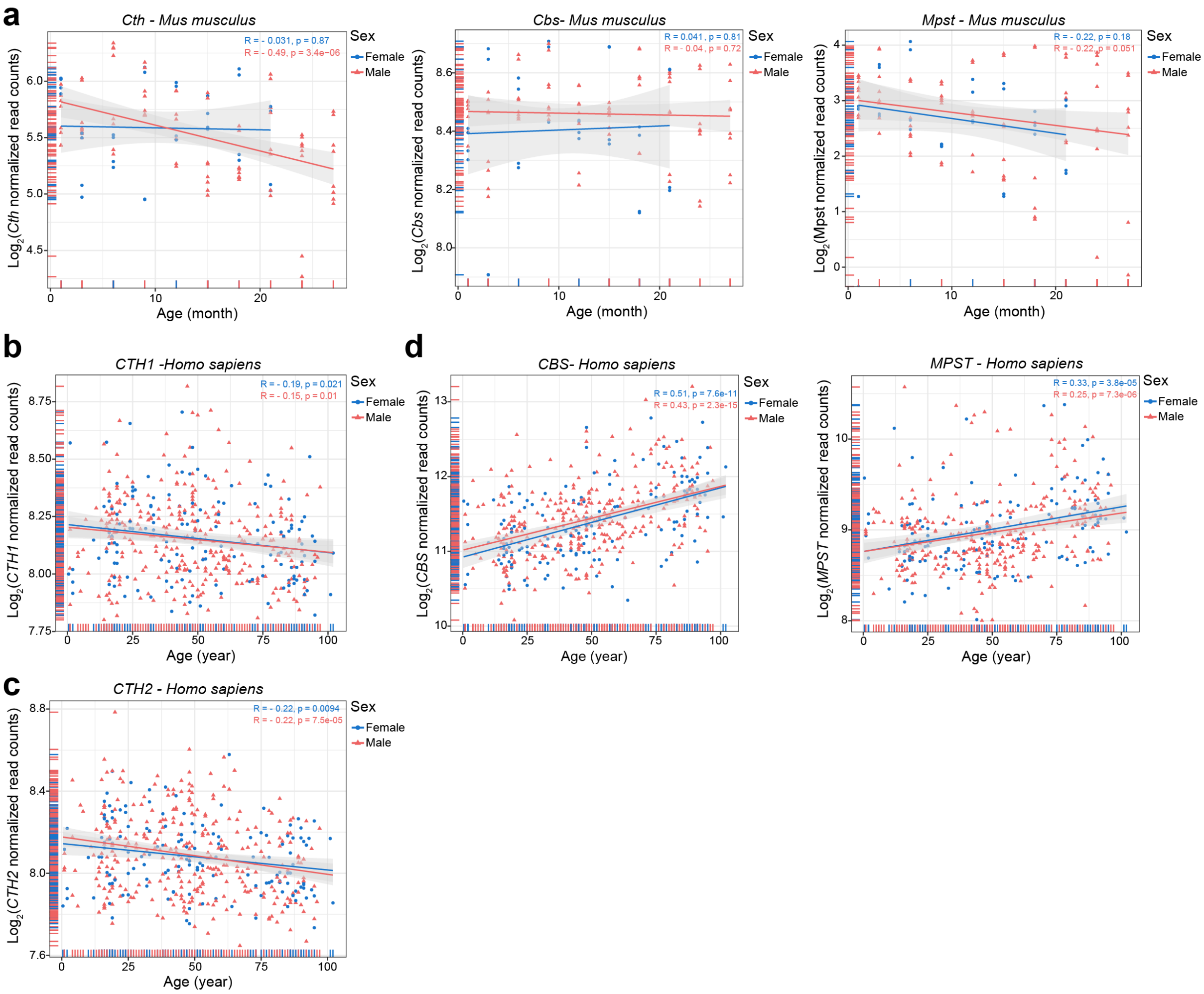

Supplementary Fig. 2. Changes in mRNA levels of H_2S producing enzymes in mouse and human brain during aging. (**a**) Plot of *Cth*, *Cbs* and *Mpst* changes in aging brain of male and female mice (based on dataset GSE132040;[34] 56 animals: 40 males and 16 females). (**b-c**) Age-induced changes in gene expression levels of two *CTH* isoforms (**b**) *CTH1* and (**c**) *CTH2* in human frontal cortex. (**d**) Age-induced changes in expression levels of *CBS* and *MPST* in human frontal cortex (based on dataset GSE36192;[35] 455 subjects: 310 males and 145 females). The linear model and Pearson's correlation R^2 and p-value were calculated using R.

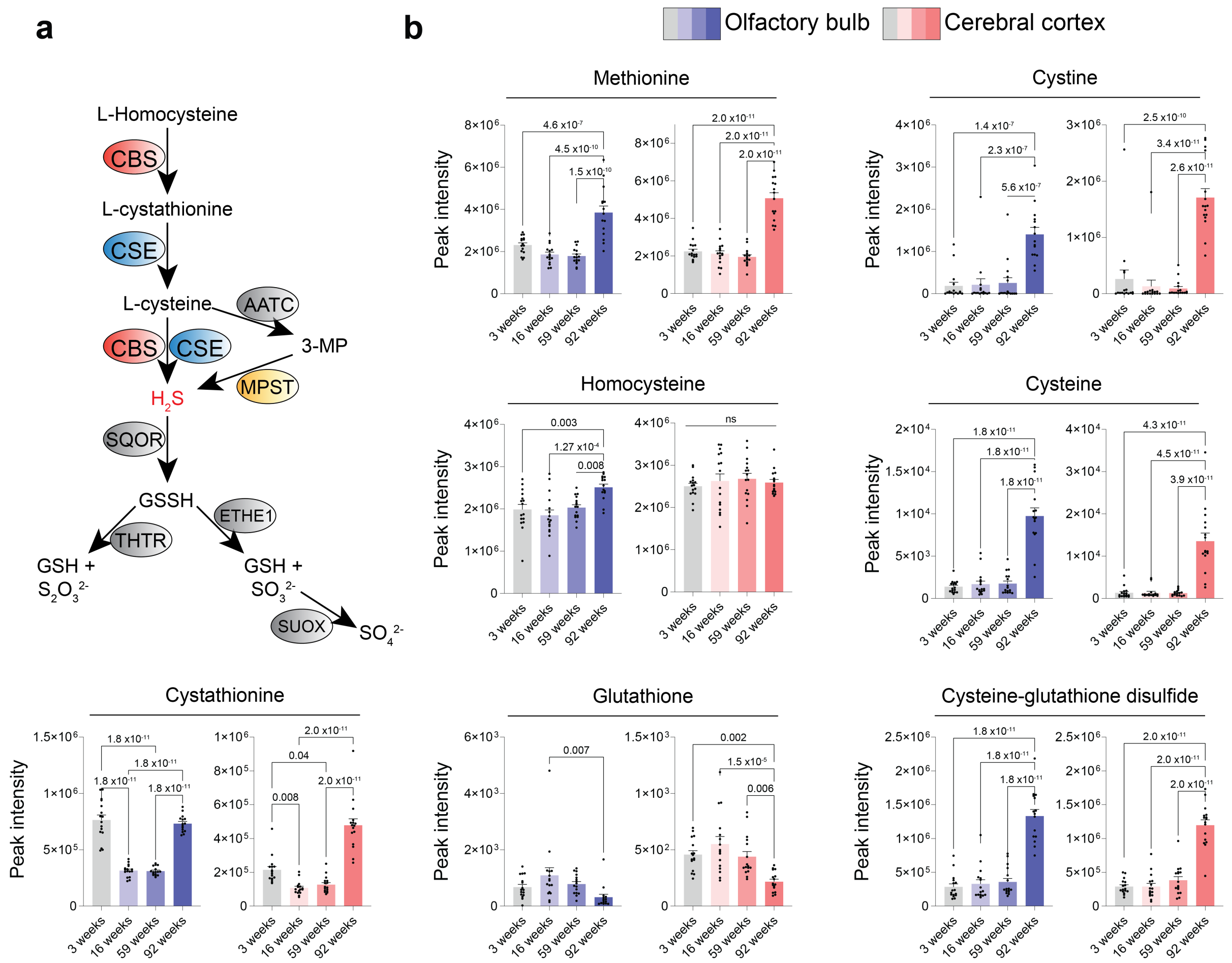

Supplementary Fig. 3. Changes in CSE substrate levels in olfactory bulb and cerebral cortex, based on aging mouse metabolome atlas. (**a**) Transsulfuration pathway. (**b**) Age-induced changes in methionine, cystine, homocysteine, cysteine, glutathione, cystathionine and cysteine-glutathione disulfide, based on Ding et al, 2021[36] metabolome atlas of the aging mouse brain.[36] Data are presented as mean ± SEM with individual dots corresponding to each animal. One-way ANOVA, tukey's HSD Test for multiple comparisons, n = 16 animals.

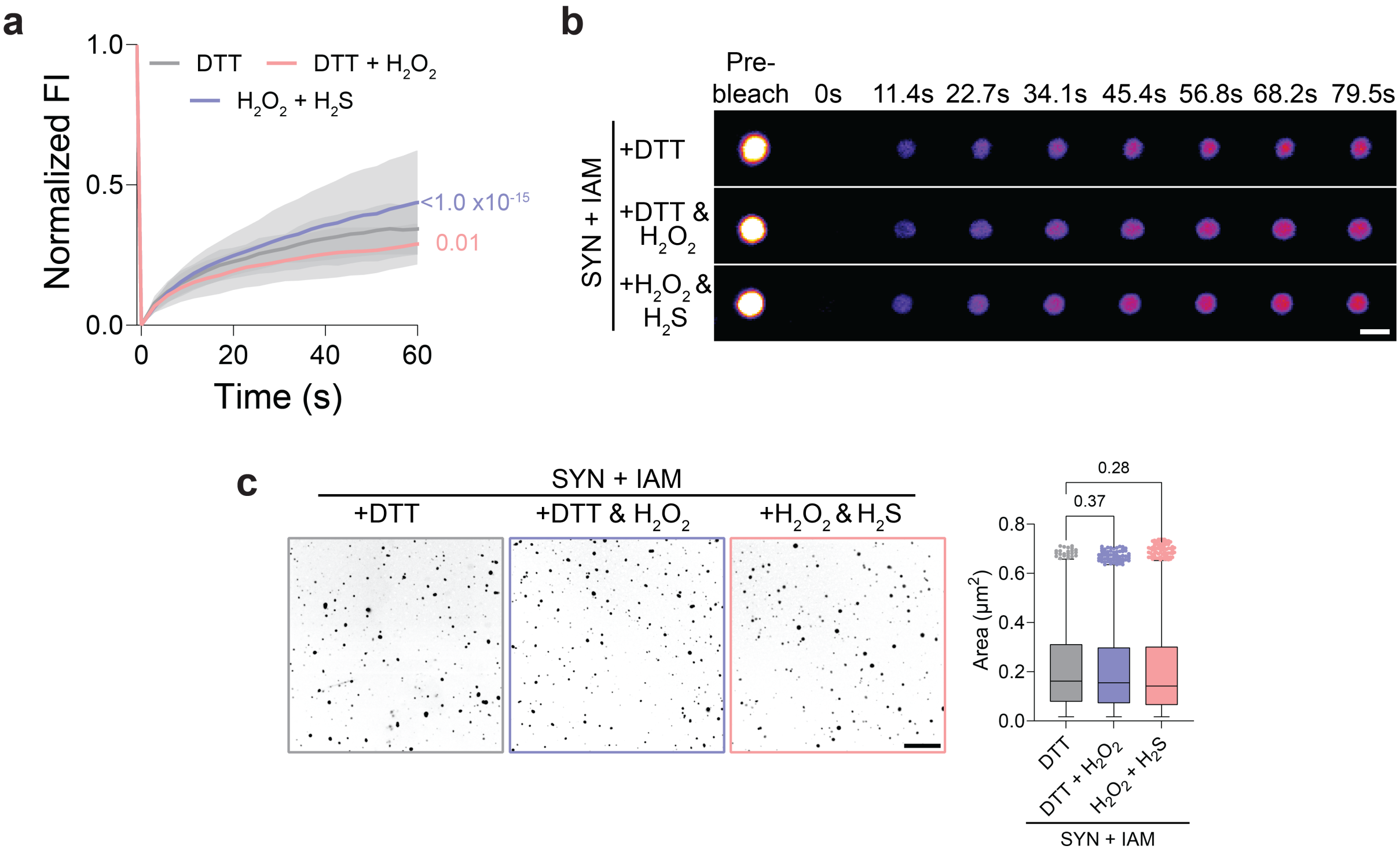

Supplementary Fig. 4. LLPS properties of IAM-modified synapsin-1 are not affected by the protocol to generate PSSH or PSO$_x$H. (**a**) FRAP quantification and (**b**) representative images of EGFP-SYN1 (10 µM + 3% PEG3000) pre-blocked with IAM (300 µM) and treated with DTT, DTT + H_2O_2 or H_2O_2 + H_2S as for Figure 3g. Means are shown as line and SD as a shade of grey (n = 105 (+DTT and DTT + H_2O_2), 97 (H_2O_2 + H_2S) condensates from 3 individual reconstitutions, two-way ANOVA, Sidak posthoc test). (**c**) Representative images and quantification of EGFP-SYN1 droplet area (in µm). The protein was pre-blocked with IAM and treated as for (a-b). Data presented as box plots with median (centre line), 25th–75th percentiles (box bounds), and min and max values (whiskers), (n = 1437 (+DTT), 2471 (DTT + H_2O_2), 2937 (H_2O_2 + H_2S) condensates over 3 individual reconstitutions, one-way ANOVA, Tukey's HSD Test for multiple comparisons.)

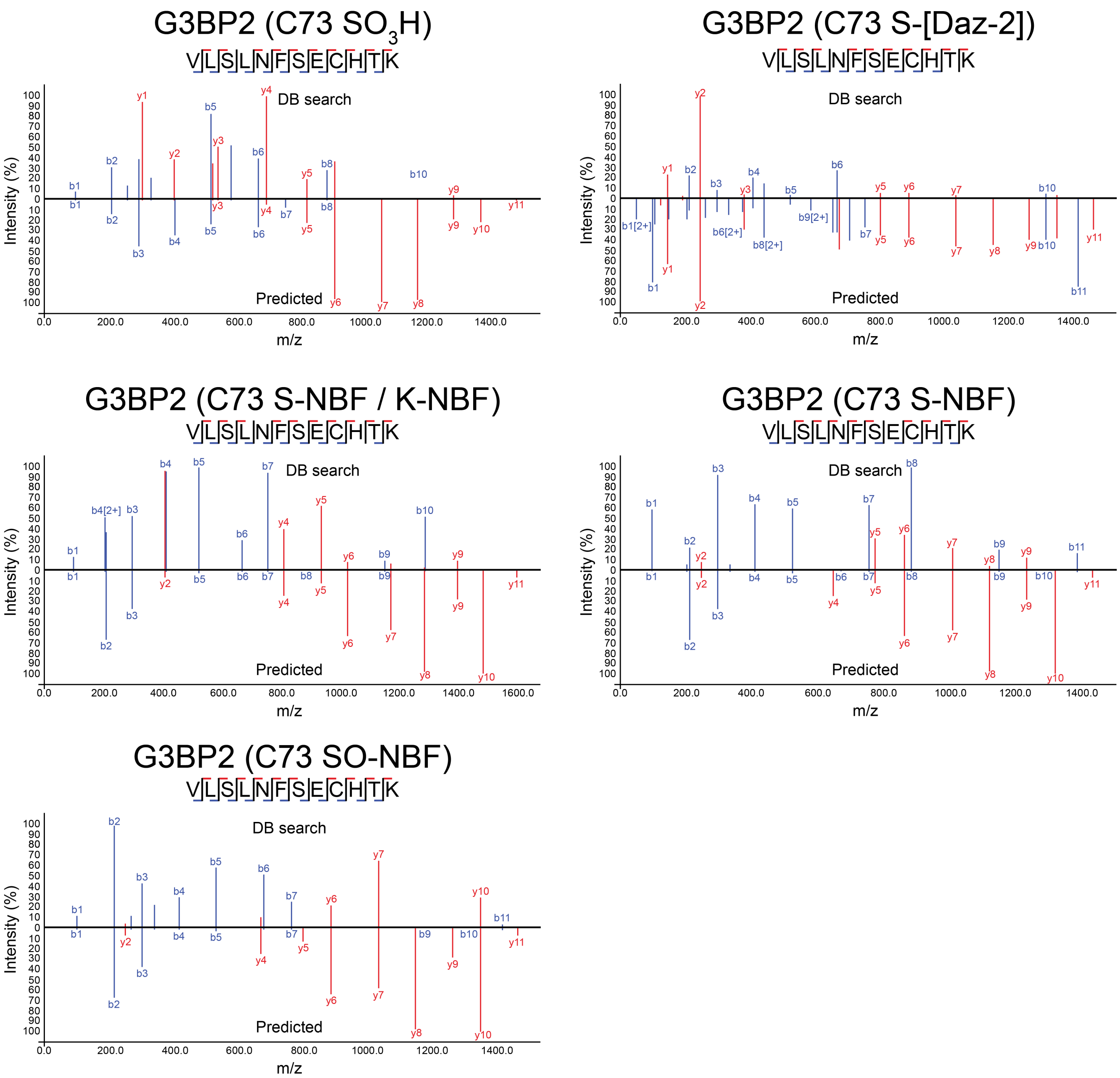

Supplementary Fig. 5. Peptide fragmentation analysis of a cysteine-containing G3BP2 peptide. WT and MEF cells were treated with 500 μM arsenite, and lysates were labeled for persulfidation using the dimedone switch method. G3BP2 was then immunopulled down and analyzed by LC–MS. Observed and predicted peptide fragmentation patterns are shown. The peptide containing a Daz-labeled cysteine indicates persulfidation, whereas PSO–NBF represents sulfenylation trapped with NBF-Cl. Sulfonylation was detected as direct cysteine tri-oxidation (+48 Da). In addition, two peptides containing either both cysteine and lysine blocked with NBF, or only cysteine blocked, were identified as measures of reduced cysteine.

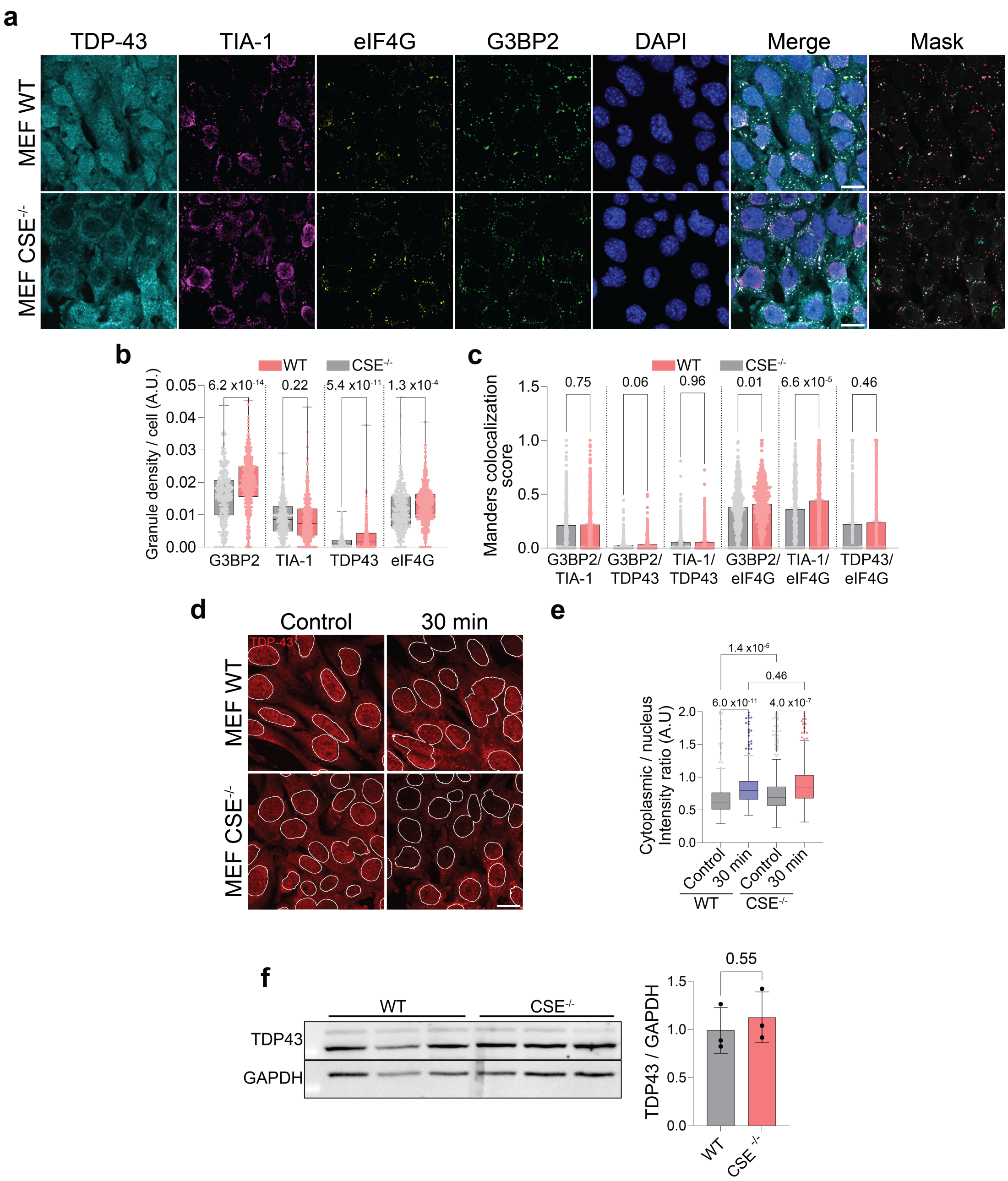

a
TDP-43
TIA-1
eIF4G
G3BP2
DAPI
Merge
Mask
MEF WT
MEF CSE-/-
b
WT
CSE-/-
Granule density / cell (A.U.)
6.2 x10-14
0.22
5.4 x10-11
1.3 x10-4
G3BP2
TIA-1
TDP43
eIF4G
c
Manders colocalization score
0.75
0.06
0.96
0.01
6.6 x10-5
0.46
G3BP2/ TIA-1
G3BP2/ TDP43
TIA-1/ TDP43
G3BP2/ eIF4G
TIA-1/ eIF4G
TDP43/ eIF4G
d
Control
30 min
TDP-43
MEF WT
MEF CSE-/-
e
1.4 x10-5
0.46
6.0 x10-11
4.0 x10-7
Cytoplasmic / nucleus Intensity ratio (A.U)
Control
30 min
WT
CSE-/-
f
WT
CSE-/-
TDP43
GAPDH
0.55
TDP43 / GAPDH

Supplementary Fig. 6. Colocalization of different SG proteins in WT and *Cse*$^{-/-}$ MEF exposed to arsenite stress. (**a**) Representative immunofluorescent images of colocalization of G3BP2, TDP-43, eiF4G and TIA-1 proteins in WT and *Cse*$^{-/-}$ MEF treated with 500 µM arsenite for 30 min. Mask images represent the detected condensates for each channel used for quantification and colocalization analysis. G3BP2 is represented in red, TIA-1 in green, eIf4G in blue and TDP-43 in cyan colour. Scale bar = 20 µM (applies to all panels). (**b**) Granule density quantification for G3BP2, TDP-43, eiF4G and TIA-1. Data presented as box plots with median (centre line), 25th–75th percentiles (box bounds), and min and max values (whiskers) with individual data points (n = 3 biological replicates, at least 3050 cells analyzed per condition, Welch´s t-test comparison between WT and *Cse*$^{-/-}$ performed independently for each protein). (**c**) Quantification of Manders colocalization coefficient calculated per cell for each indicated pair of proteins. Colocalization was performed on defined ROI rather than whole cell. Data presented as box plots with median (centre line), 25th–75th percentiles (box bounds), and min and max values (whiskers) with individual data points (n = 3 biological replicates, at least 300 cells quantified per conditions, Welch´s t-test comparison between WT and *Cse*$^{-/-}$ performed independently for each pair of proteins). (**d-e**) Representative immunofluorescence images (**d**) of TDP43 cytoplasmic and nuclear localization. White lines indicate nucleus overlay. (**e**) Data presented as box plots with median (centre line), 25th–75th percentiles (box bounds), and min and max values (whiskers) with individual data points (n = 3 biological replicates, at least 300 cells quantified, one-way ANOVA, Tukey’s HSD Test for multiple comparisons). (**f**) Western blot of TDP43 levels in WT and *Cse*$^{-/-}$ MEF. TDP43 levels were normalized to GAPDH loading control. Data are presented as mean ± SD with individual data points, (n = 3 biological replicates, Welch´s t-test).

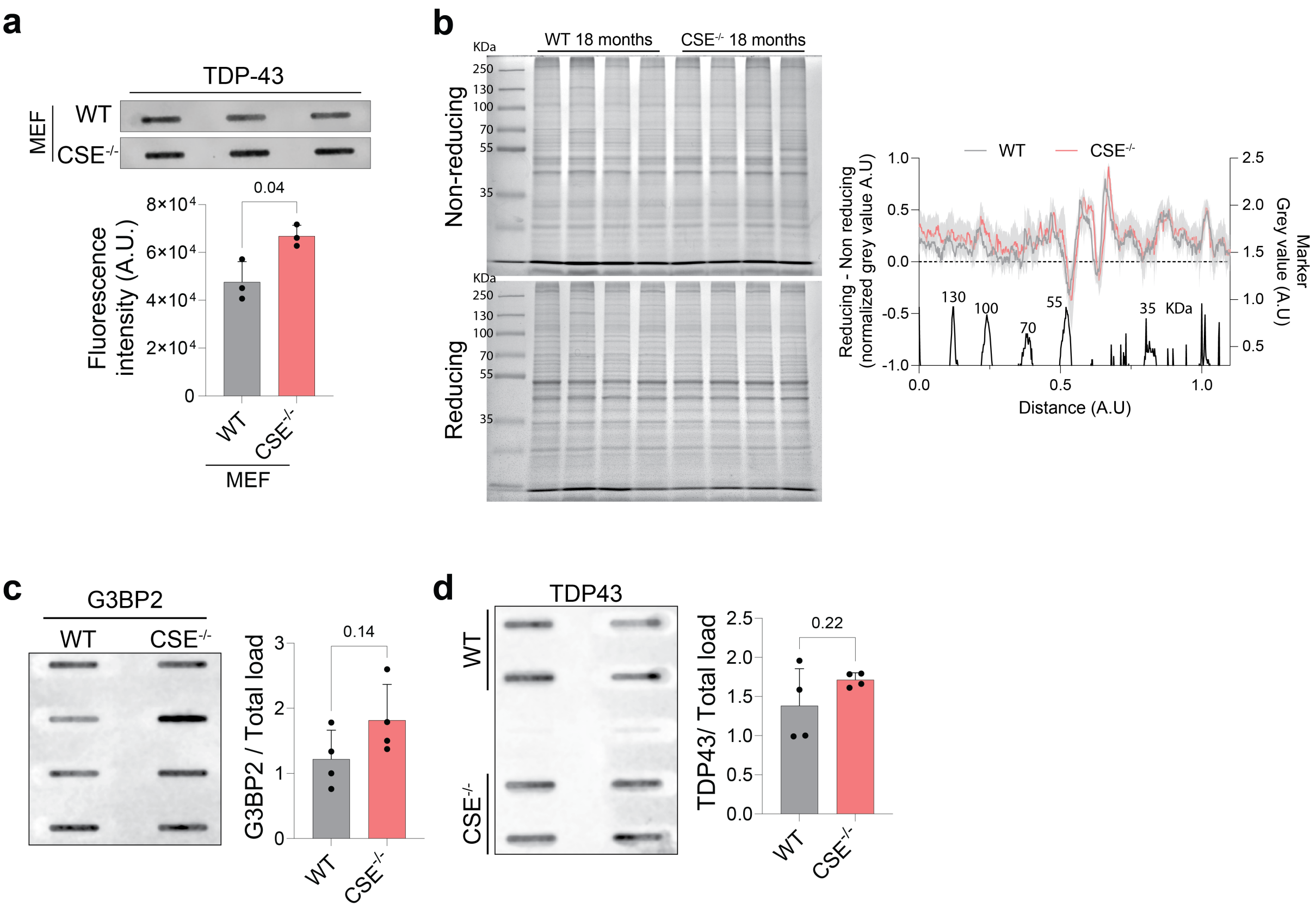

Supplementary Fig. 7. Age- and CSE-dependent accumulation of aggregated stress-granule proteins. (**a**) Filter trap assay detecting aggregated TDP-43 in lysates from WT and Cse$^{-/-}$ MEFs. Data are presented as mean ± SD with individual data point, (n = 3 biological replicates; Welch's *t*-test). (**b**) Coomassie staining of lysates from 18-month-old WT and Cse$^{-/-}$ mouse brains under reducing (+β-mercaptoethanol) and non-reducing (−β-mercaptoethanol) conditions. Grey value profiles were generated for each lane and used to calculate Δ profiles for WT and Cse$^{-/-}$ samples. Data are presented as mean (solid line) ± SD (grey bands), n = 4 animals. The black line represents the molecular weight marker profile, with marker sizes indicated in kDa. (c,d) Filter trap assays performed on lysates from 18-month-old WT and Cse$^{-/-}$ mouse brains using antibodies against (**c**) G3BP2 and (**d**) TDP-43. Signal intensities were normalized to total protein loading shown in (**b**) (n = 4 biological replicates; Welch's *t*-test). Data are presented as mean ± SD with individual data point.

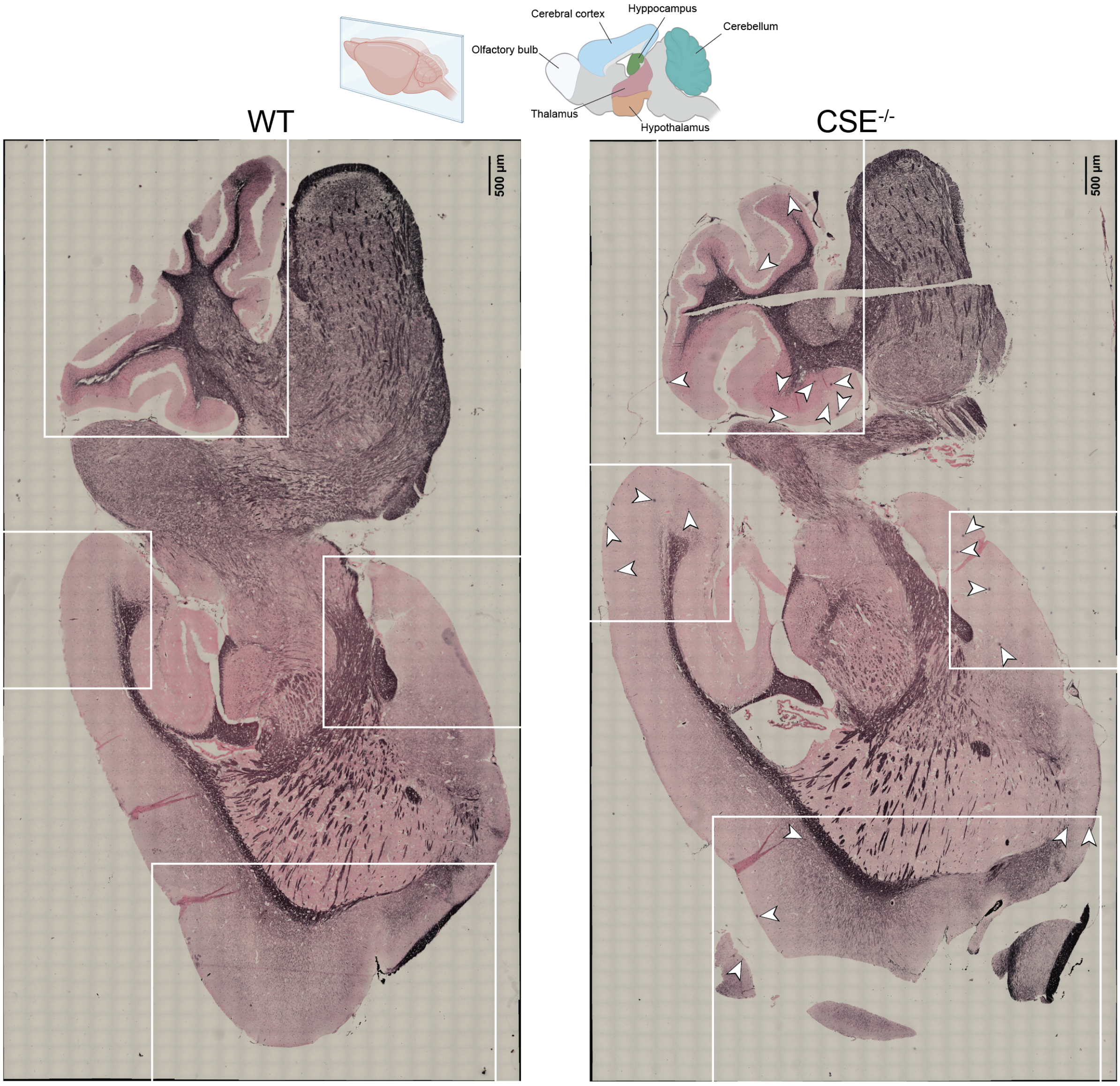

Supplementary Fig. 8. Old *Cse*$^{-/-}$ mice exhibit multiple Gallyas silver–stained neuritic-like plaques. Representative microscopy images of Gallyas staining of the sagittal brain section in 18-month-old WT and *Cse*$^{-/-}$ mice. Fibrillary tangles (white arrows) were present in different regions of the *Cse*$^{-/-}$ mice such as the hypothalamus, the cerebral cortex, and the cerebellum (indicated by the white boxes). WT mice did not show any detectable lesion (n = 4 animals, scale bar = 500 µM).

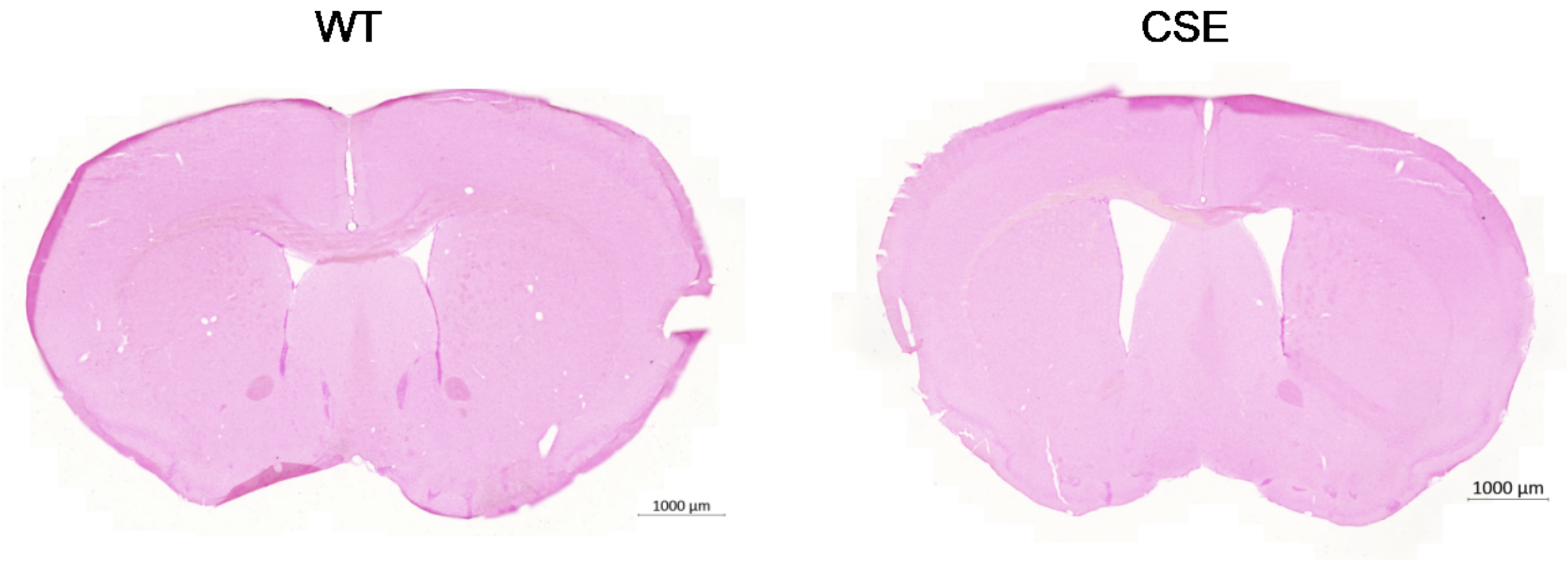

Supplementary Fig. 9. 10-week-old Cse$^{-/-}$ mice do not exhibit positive Gallyas silver–stained neuritic-like plaques. Representative microscopy images of Gallyas staining of the frontal plane brain section in 10-week-old WT and *Cse*$^{-/-}$ mice. (n = 4 animals, scale bar = 1000 µM).

a

G3BP2 - C73

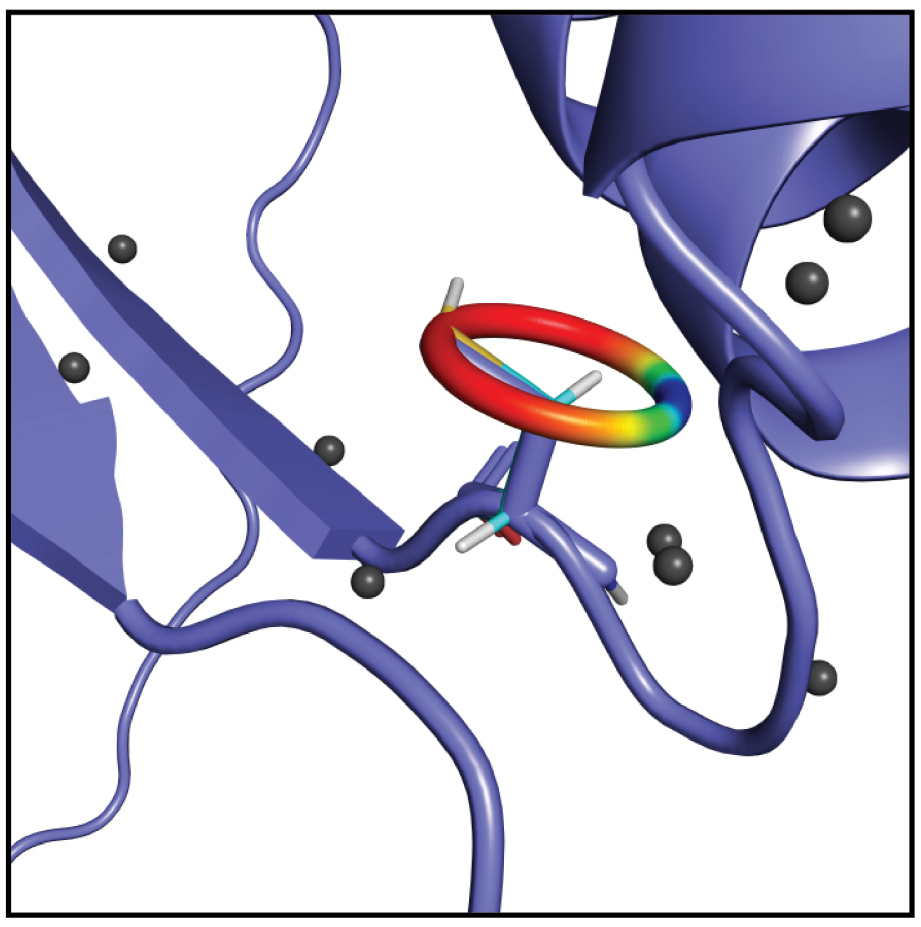

G3BP2	WT	C/S	C/E
Total sasa	342.229	496.19	459.206
Hydrophobic sasa	50.806	36.747	16.164
Hydrophobicity	0.148456	0.074058	0.035200

b

Synapsin 1 - C360

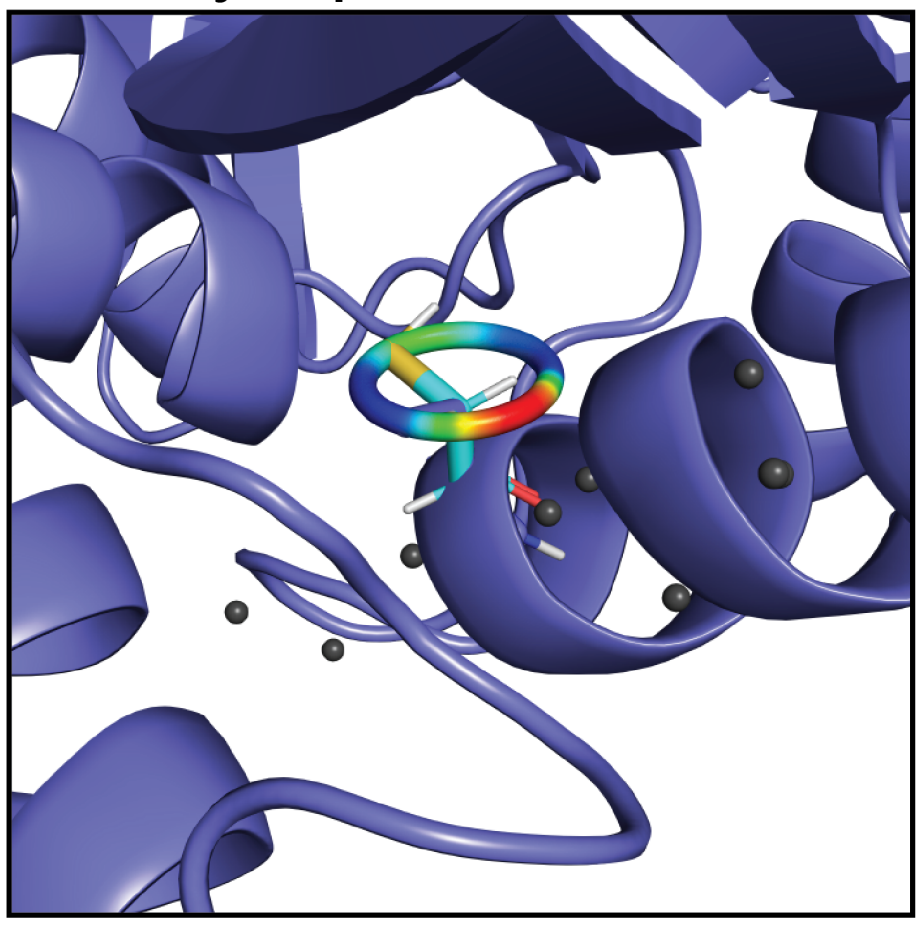

Synapsin-1	WT	C/S	C/E
Total sasa	192.914	200.261	227.544
Hydrophobic sasa	19.009	35.761	48.46
Hydrophobicity	0.098536	0.178572	0.212970

Supplementary Fig. 10. Local microenvironments of redox-active cysteines in G3BP2 and synapsin-1. (**a–b**) The local cysteine environments of (a) G3BP2 and (**b**) synapsin-1 are shown in PyMOL. To assess how mutations that mimic oxidized states alter the physicochemical microenvironment, AlphaFold models of the corresponding C→S and C→E variants were generated, and the solvent-accessible surface area (SASA) and hydrophobic SASA within an 8 Å sphere surrounding the cysteine Cα atom were quantified. The resulting hydrophobicity indices (hydrophobic SASA / total SASA) are summarized in the tables below each structural panel.

Supplementary fig. 6f

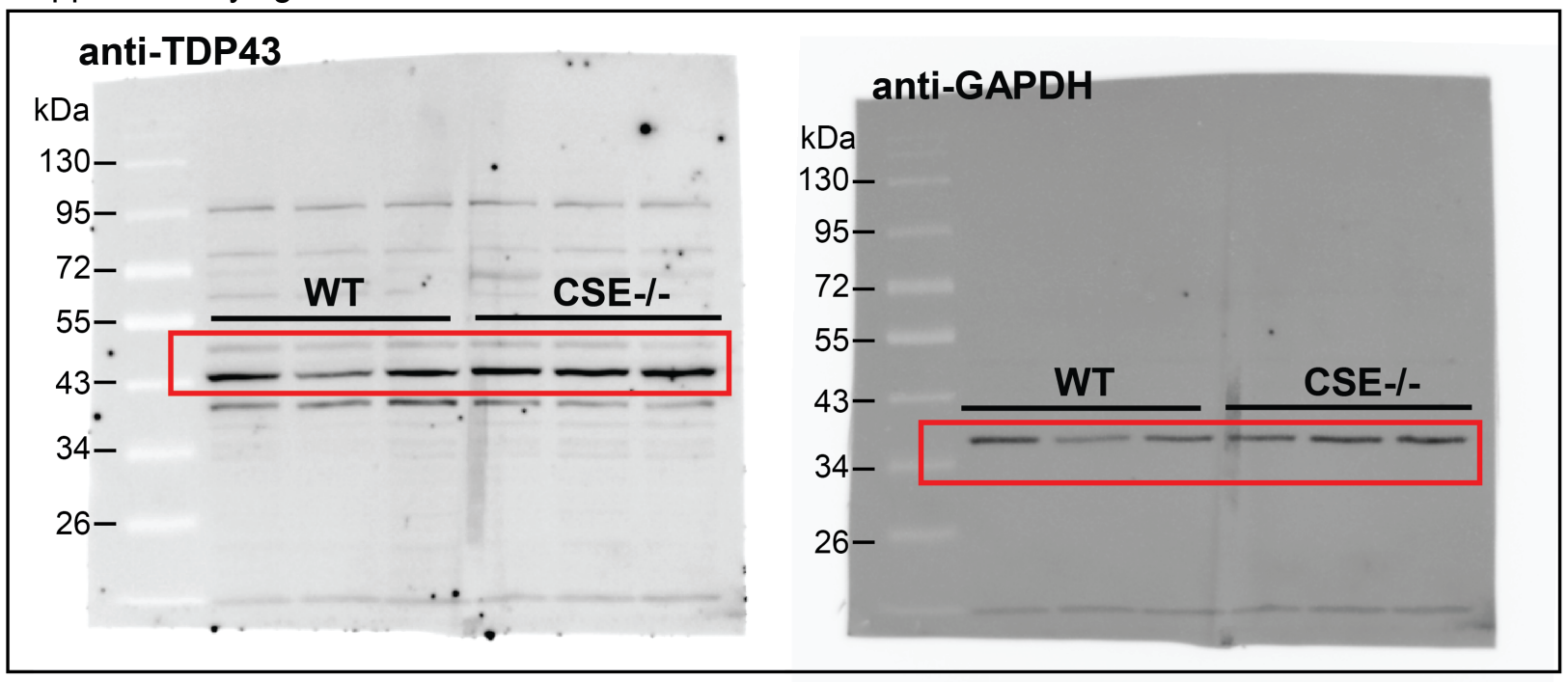

Supplementary fig. 7a

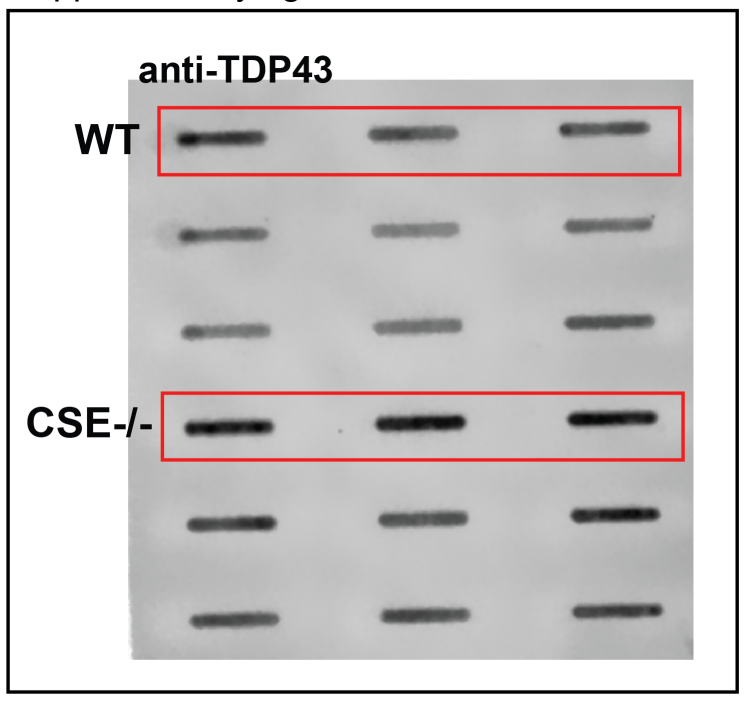

Supplementary fig. 7b

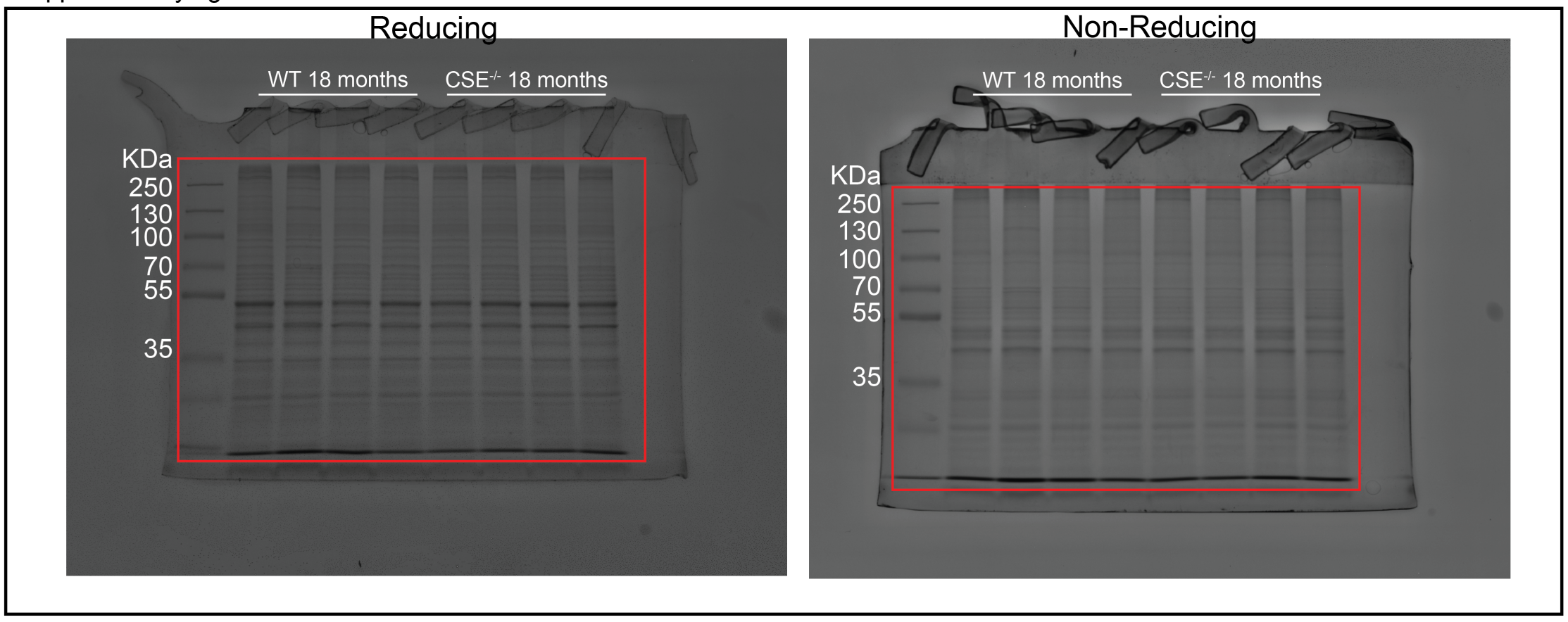

Supplementary fig. 7c

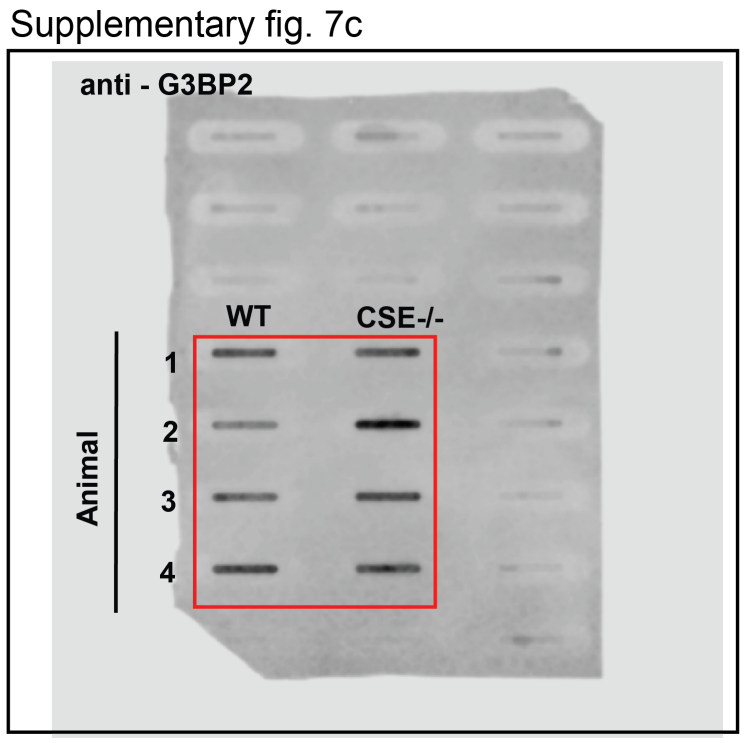

Supplementary fig. 7d

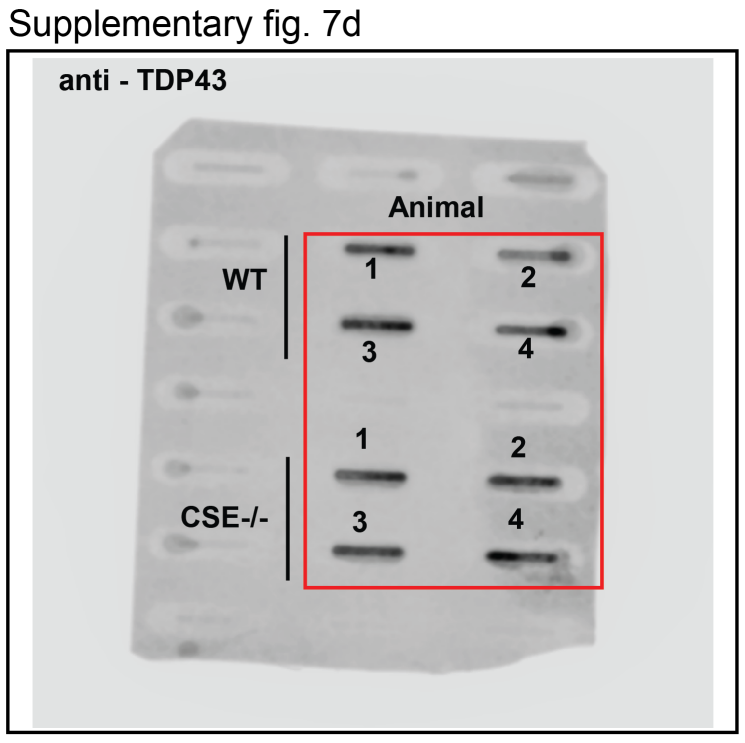

Uncropped blot from supplementary figures
