# Supplementary material for: Protein thiol alterations drive pathologic liquid–liquid phase separation in the aging brain
Source: Nat Struct Mol Biol. 2026 Jul 30;33(8):1252–65. doi: 10.1038/s41594-026-01857-w (PMC13468144; doi:10.1038/s41594-026-01857-w)

Extended data Fig. 6a - PSOH

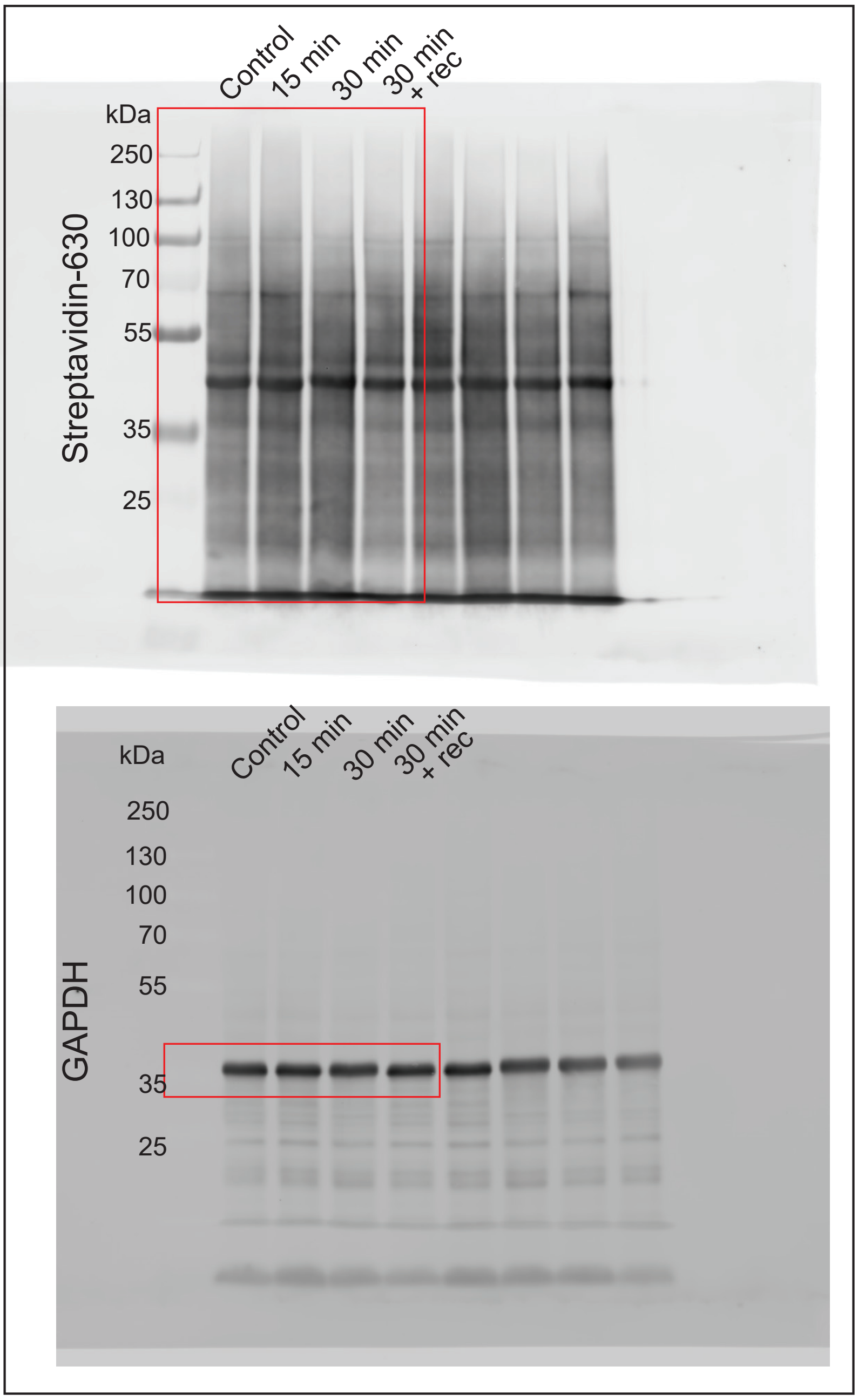

Extended data Fig. 6a - PSSH

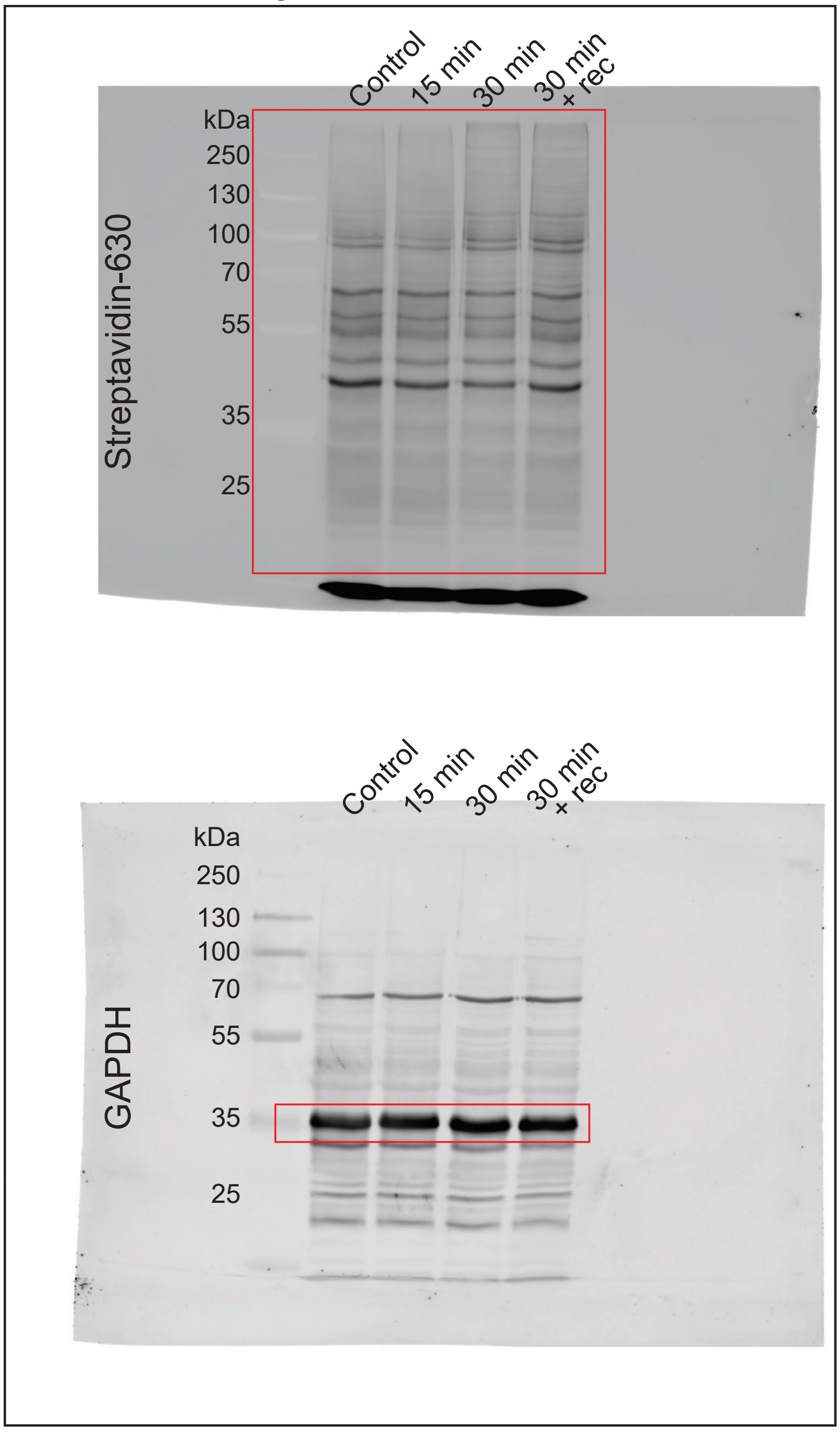

Extended data Fig. 6b - PSOH

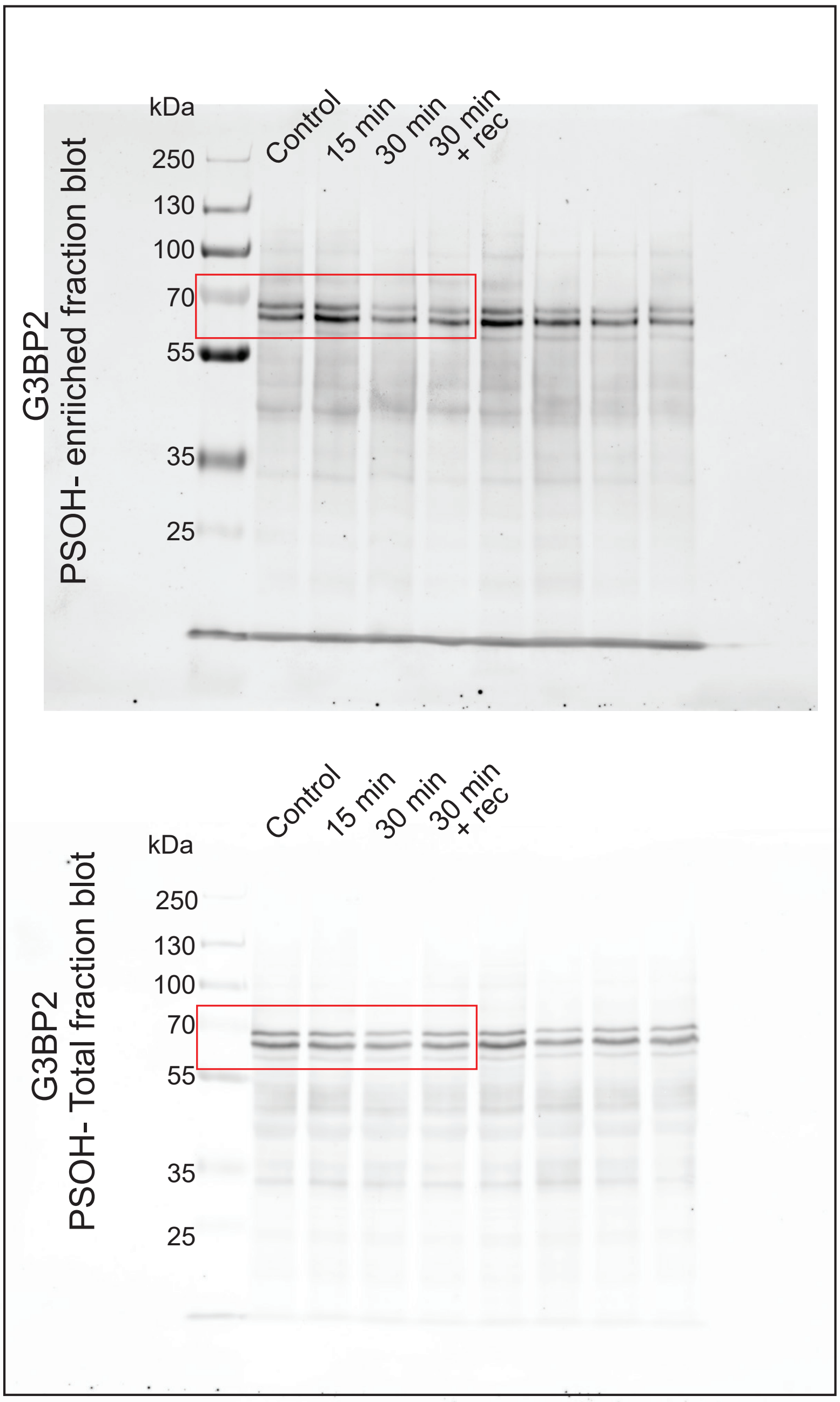

Extended data Fig. 6b - PSSH

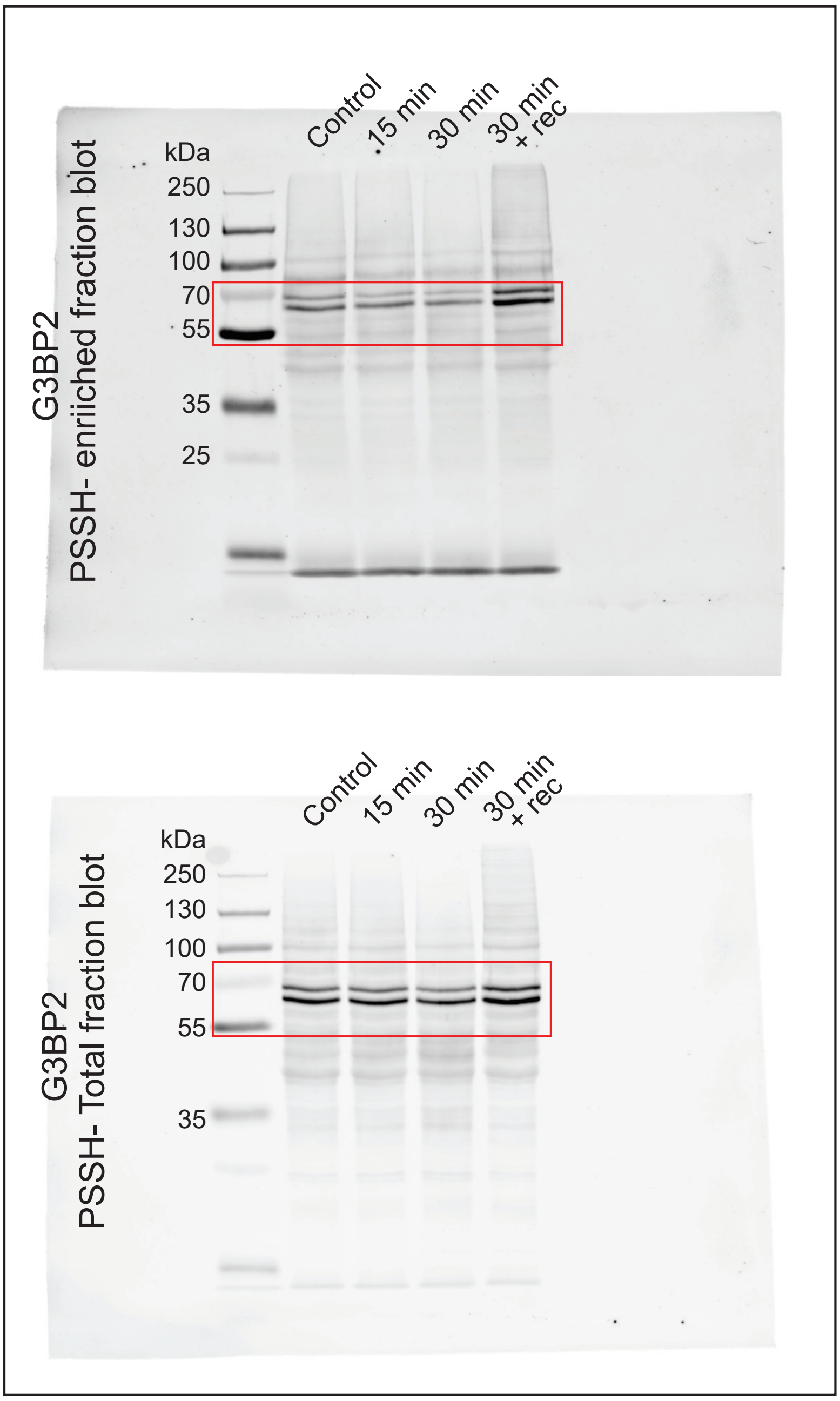

Supplement: Supplementary file 6 [file 41594_2026_1857_MOESM28_ESM.pdf]
