# Supplementary material for: Protein thiol alterations drive pathologic liquid–liquid phase separation in the aging brain
Source: Nat Struct Mol Biol. 2026 Jul 30;33(8):1252–65. doi: 10.1038/s41594-026-01857-w (PMC13468144; doi:10.1038/s41594-026-01857-w)
Supplement: Supplementary file 7 [file 41594_2026_1857_MOESM2_ESM.pdf]

nature portfolio

Corresponding author(s): Milos R. Filipovic , Thibaut Vignane

Last updated by author(s): May 5, 2026

Reporting Summary

Nature Portfolio wishes to improve the reproducibility of the work that we publish. This form provides structure for consistency and transparency in reporting. For further information on Nature Portfolio policies, see our Editorial Policies and the Editorial Policy Checklist.

Statistics

For all statistical analyses, confirm that the following items are present in the figure legend, table legend, main text, or Methods section.

n/a	Confirmed	
☐	☒	The exact sample size (*n*) for each experimental group/condition, given as a discrete number and unit of measurement
☐	☒	A statement on whether measurements were taken from distinct samples or whether the same sample was measured repeatedly
☐	☒	The statistical test(s) used AND whether they are one- or two-sided<br>*Only common tests should be described solely by name; describe more complex techniques in the Methods section.*
☐	☒	A description of all covariates tested
☐	☒	A description of any assumptions or corrections, such as tests of normality and adjustment for multiple comparisons
☐	☒	A full description of the statistical parameters including central tendency (e.g. means) or other basic estimates (e.g. regression coefficient) AND variation (e.g. standard deviation) or associated estimates of uncertainty (e.g. confidence intervals)
☐	☒	For null hypothesis testing, the test statistic (e.g. *F*, *t*, *r*) with confidence intervals, effect sizes, degrees of freedom and *P* value noted<br>*Give P values as exact values whenever suitable.*
☒	☐	For Bayesian analysis, information on the choice of priors and Markov chain Monte Carlo settings
☒	☐	For hierarchical and complex designs, identification of the appropriate level for tests and full reporting of outcomes
☐	☒	Estimates of effect sizes (e.g. Cohen's *d*, Pearson's *r*), indicating how they were calculated

Our web collection on statistics for biologists contains articles on many of the points above.

Software and code

Policy information about availability of computer code

Data collection	The persulfidome was collected using a Thermo LTQ Velos Mass Spectrometer (Thermo Fisher); Sulfenylome, solfonylated peptides and the thermal proteome profiling were collected using Orbitrap Eclipse™ Tribrid™ Mass Spectrometer (Thermo Fisher) and its associated Xcalibur Software. Total proteome, condensate detection and direct detection of cysteine PTMs were collected using a timsTOF Pro (Bruker) and its associated software. Images of in-vitro phase separation of EGFP-synapsin1 and G3BP2 as well as the FRAP experiments were collected on an Eclipse Ti Nikon Spinning Disk Confocal CSU-X microscope with the NIS-Elements imaging software. Immunofluorescence images were acquired using a Leica SP8 LIGHTNING Confocal microscope associated with the LASX software or an Evident CSU-W1 spinning disk confocal microscope with cellSens Dimensions software. The latest was also used for G3BP2 condensate dissolution.
Data analysis	PEAKS ONLINE or Fragpipe (v21.0) softwares were used for the label free quantification analysis and the TMT quantification analysis respectively. The Perseus software (1.6.15.0) was used for proteomic statistical analysis. R (v4.4) and R studio were used for proteomic analysis and visualization.For KEGG and GO term enrichment analysis, DAVID webtool was used. Fiji (ImageJ v1.54p) and Spyder 5 (python 3.13) were used for microscopy images and western blot quantification. Graphpad prism 11 was used to performed statistical analysis and graph for data visualization.

For manuscripts utilizing custom algorithms or software that are central to the research but not yet described in published literature, software must be made available to editors and reviewers. We strongly encourage code deposition in a community repository (e.g. GitHub). See the Nature Portfolio guidelines for submitting code & software for further information.

Data

Policy information about availability of data

All manuscripts must include a data availability statement. This statement should provide the following information, where applicable:

- Accession codes, unique identifiers, or web links for publicly available datasets
- A description of any restrictions on data availability
- For clinical datasets or third party data, please ensure that the statement adheres to our policy

All raw data not included in this study are available from the corresponding author upon reasonable request. All proteomic data are available through PRIDE under code numbers: PXD045785 (Sulfenylome and total proteome); PXD045806 (Persulfidation and Sulfonylation); PXD045820 (TPP of WT and Cse-/- MEFs upon H2O2 stress). Galyas silver staining images of 18-month-old WT and Cse-/- are available on BioImage Archive98 repository (DOI: 10.6019\\S-BIAD1604; https://www.ebi.ac.uk/biostudies/bioimages/studies/S-BIAD1604?key=2c830e02-9dfe-429f-a38f-fbe2336e7326).

Research involving human participants, their data, or biological material

Policy information about studies with human participants or human data. See also policy information about sex, gender (identity/presentation), and sexual orientation and race, ethnicity and racism.

Reporting on sex and gender

Use the terms sex (biological attribute) and gender (shaped by social and cultural circumstances) carefully in order to avoid confusing both terms. Indicate if findings apply to only one sex or gender; describe whether sex and gender were considered in study design; whether sex and/or gender was determined based on self-reporting or assigned and methods used.
Provide in the source data disaggregated sex and gender data, where this information has been collected, and if consent has been obtained for sharing of individual-level data; provide overall numbers in this Reporting Summary. Please state if this information has not been collected.
Report sex- and gender-based analyses where performed, justify reasons for lack of sex- and gender-based analysis.

Reporting on race, ethnicity, or other socially relevant groupings

Please specify the socially constructed or socially relevant categorization variable(s) used in your manuscript and explain why they were used. Please note that such variables should not be used as proxies for other socially constructed/relevant variables (for example, race or ethnicity should not be used as a proxy for socioeconomic status).
Provide clear definitions of the relevant terms used, how they were provided (by the participants/respondents, the researchers, or third parties), and the method(s) used to classify people into the different categories (e.g. self-report, census or administrative data, social media data, etc.)
Please provide details about how you controlled for confounding variables in your analyses.

Population characteristics

Describe the covariate-relevant population characteristics of the human research participants (e.g. age, genotypic information, past and current diagnosis and treatment categories). If you filled out the behavioural & social sciences study design questions and have nothing to add here, write "See above."

Recruitment

Describe how participants were recruited. Outline any potential self-selection bias or other biases that may be present and how these are likely to impact results.

Ethics oversight

Identify the organization(s) that approved the study protocol.

Note that full information on the approval of the study protocol must also be provided in the manuscript.

Field-specific reporting

Please select the one below that is the best fit for your research. If you are not sure, read the appropriate sections before making your selection.

☒ Life sciences ☐ Behavioural & social sciences ☐ Ecological, evolutionary & environmental sciences

For a reference copy of the document with all sections, see nature.com/documents/nr-reporting-summary-flat.pdf

Life sciences study design

All studies must disclose on these points even when the disclosure is negative.

Sample size

No statistical methods were used to predetermine sample sizes. For most experiments, a sample size of 3–4 was selected based on pilot studies indicating that a minimum of three animals was sufficient to achieve reproducible results with limited variability. A sample size of $n \geq 3$ was sufficient for statistical analysis. Experiments with fewer than three reconstitutions were constrained by limited yields of purified protein.

Data exclusions

No data point were excluded from our analysis.

Replication

All cell and animal experiments have been performed with at least 3 biological replicates. In-vitro assays were performed with at least 2 reconstitution and several measurements per reconstitution. Lifespan of CSE-/- was performed twice with 8 animals per group each time (Total number 16 animals).

Randomization

Mice were randomly chosen when collected at different ages. To avoid batch effect, proteomic samples were processed in batches containing

Randomization	no more than 1 replicate and measured in a randomized way to avoid memory effect of the reverse-phase columns.
Blinding	The investigator were not blinded to the conditions of the experiments.

Reporting for specific materials, systems and methods

We require information from authors about some types of materials, experimental systems and methods used in many studies. Here, indicate whether each material, system or method listed is relevant to your study. If you are not sure if a list item applies to your research, read the appropriate section before selecting a response.

Materials & experimental systems

n/a	Involved in the study
☐	☒ Antibodies
☐	☒ Eukaryotic cell lines
☒	☐ Palaeontology and archaeology
☐	☒ Animals and other organisms
☒	☐ Clinical data
☒	☐ Dual use research of concern
☒	☐ Plants

Methods

n/a	Involved in the study
☒	☐ ChIP-seq
☒	☐ Flow cytometry
☒	☐ MRI-based neuroimaging

Antibodies

Antibodies used

anti-G3BP2 (Proteintech®, 16276-1-AP, 1:4000 for WB, 1:600 for IF)
anti-Cth (Proteintech®, 12217-1-AP, 1:1000),
anti-Cth (Santa Cruz Biotechnology, INC.; sc-374249, 1:750),
anti-eIF2α 1 (Invitrogen, AHO0802, 1:1000),
anti-TDP43 (Proteintech®, 10782-2-AP, 1:1000),
anti-GAPDH-AF488 (Sigma-Aldrich, MAB374-AF488, 1:2000),
anti-GAPDH(6C5)-AF488 (Merck, MAB374, 1:2000),
anti-Prdx-SO3 (Abcam, #ab16830, 1:4000),
anti-oxDJ-1 (Sigma-Aldrich, #MABN1773, 1:2000),
anti-β-Tubulin (Sigma-Aldrich, #T0198, 1:20000),
anti-beta-amyloid(b-4) (Santa Cruz Biotechnology, INC.; sc-28365,1:300),
anti-Tau(D-8) (Santa Cruz Biotechnology, INC.; sc-166060, 1:300),
anti-TDP43-AF790 (Santa Cruz Biotechnology, INC.; sc-376311, 1:250 for IF)
anti-eIF4G-AF594 (Santa Cruz Biotechnology, INC.; sc-133155, 1:250 for IF)
anti-TIA-1/TIAR(D-9)AF-647 (Santa Cruz Biotechnology, INC.; sc-48371, 1:250 for IF)

*WB: Westernblot, IF: immunofluorescence

Validation

anti-G3BP2 (16276-1-AP). Validated for WB and IF in mouse samples.
https://www.citeab.com/antibodies/2887117-16276-1-ap-g3bp2-polyclonal-antibody?des=becb57432648619e
anti-Cth (12217-1-AP). Validated for WB in mouse samples.
https://www.citeab.com/antibodies/81731-12217-1-ap-gamma-cystathionase-polyclonal-antibody?des=76208387e67f22bf
anti-Cth (sc-374249). Suppliers website: CTH Antibody (F-1) is recommended for detection of CTH isoforms 1 and 2 of mouse, rat and human origin by WB, IP, IF, IHC(P) and ELIS.
anti-eIF2α 1 (AHO0802). Validated for WB and IF in mouse samples.
https://www.citeab.com/antibodies/2402940-aho0802-eif2s1-monoclonal-antibody-eif2-alpha?des=fd89f509c14c98aa
anti-TDP43 (10782-2-AP). Validated for WB in mouse samples.
https://www.citeab.com/antibodies/80951-10782-2-ap-tdp-43-polyclonal-antibody?des=038174b1546a3102
anti-GAPDH (MAB374). Validated by knockdown. Reactivity mouse and human.
https://www.citeab.com/antibodies/226212-mab374-anti-glyceraldehyde-3-phosphate-dehydrogenase?des=9dc239fd3cc26a47
anti-Prdx-SO3 (#ab16830). For mouse samples.
Validated with biochemical experiment : https://pubmed.ncbi.nlm.nih.gov/29048427/.
https://www.citeab.com/antibodies/745811-ab16830-anti-peroxiredoxin-so3-antibody#images-and-citations
anti-oxDJ-1 (#MABN1773). Reaction in mouse samples.
https://www.citeab.com/antibodies/3289869-mabn1773-anti-oxdj-1-antibody-cys106-clone-m149?des=2385238c8ee80089
anti-β-Tubulin (#T0198). Validated fore mouse and human samples reactivity.
https://www.citeab.com/antibodies/2281299-t0198-anti-tubulin-antibody-mouse-monoclonal?des=543d41167e0fed37
anti-beta-amyloid(b-4) (sc-28365). Validated for mouse samples reactivity.
https://www.citeab.com/antibodies/786904-sc-28365-amyloid-antibody-b-4?des=f0ab63578805b014
anti-Tau(D-8) (sc-166060). Validated for mouse samples reactivity.
https://www.citeab.com/antibodies/829993-sc-166060-tau-antibody-d-8?des=f48cd4599b3dd57c
anti-TDP43-AF790 (sc-376311). Validated by knockdown for mouse reactivity in WB and IF.
https://www.citeab.com/antibodies/829813-sc-376311-tardbp-antibody-e-10?des=233fb641653cddcb
anti-eIF4G-AF594 (sc-133155). Validated for IF in mouse samples.
https://www.citeab.com/antibodies/791053-sc-133155-eif4g-antibody-a-10?des=b5be06933a9ad85f
anti-TIA-1/TIAR(D-9) (sc-48371). Validated for IF in mouse samples.

https://www.citeab.com/antibodies/830803-sc-48371-tia-1-tiar-antibody-d-9?des=250dbefc32e8ee92

Eukaryotic cell lines

Policy information about cell lines and Sex and Gender in Research

Cell line source(s)	Cse−/− mouse embryonic fibroblasts (MEF) were from Professor Bindu Paul (Johns Hopkins University) and were generated from Cse+/+ and Cse−/− mice and immortalized using SV40T antigen. (Sbodio et al., 2016)<br>STHdhQ7/Q7and STHdhQ111/Q111 cell lines are a striatal derived cell line from a knock-in transgenic mouse containing homozygous Huntingtin (HTT) loci with a humanized Exon 1 containing 7 or 111 polyglutamine repeats respectively. Both cell lines are available at Coriell institute for medical research (Cat #: CH00097; CH00095).<br>Human primary fibroblasts (AG1449, AG16359) were obtained from the Coriell Institute for Medical Research.
Authentication	Not authenticated.
Mycoplasma contamination	Mycoplasma infection was regularly assessed using visual detection assay (DAPI), and mycosplasma test (strips).
Commonly misidentified lines (See ICLAC register)	No commonly misidentified cell lines were used in this study.

Animals and other research organisms

Policy information about studies involving animals; ARRIVE guidelines recommended for reporting animal research, and Sex and Gender in Research

Laboratory animals	C57Bl/6J mice were purchased from the Jackson laboratory (Strain #: 000664). The CSE knockout has been previously described (Yang et al., 2008). All the studies were done on male mice at 10 weeks, 10 months, or 18 months old.
Wild animals	No wild animals were used.
Reporting on sex	Only males were used in this study.
Field-collected samples	No filed-collected samples were used in this study.
Ethics oversight	Animals were bred/housed in ventilated cages, under specific pathogen-free, temperature controlled (22 °C) and 12 h light/dark cycle conditions in full compliance with the guidelines of the Federation of Laboratory Animal Science Association recommendations in the Laboratory Animal Unit of Biomedical Research Foundation of the Academy of Athens (BRFAA) and allowed unrestricted access to chow diet and water. As per Greek regulations, no individual license is required for experiments where animals are not treated. All experiment procedures reported here were approved by the Veterinary Authority of the Prefecture of Athens, in accordance with National Registration (Presidential Decree 56/2013), harmonized with European Directive 2010/63EU.

Note that full information on the approval of the study protocol must also be provided in the manuscript.

Plants

Seed stocks	*Report on the source of all seed stocks or other plant material used. If applicable, state the seed stock centre and catalogue number. If plant specimens were collected from the field, describe the collection location, date and sampling procedures.*
Novel plant genotypes	*Describe the methods by which all novel plant genotypes were produced. This includes those generated by transgenic approaches, gene editing, chemical/radiation-based mutagenesis and hybridization. For transgenic lines, describe the transformation method, the number of independent lines analyzed and the generation upon which experiments were performed. For gene-edited lines, describe the editor used, the endogenous sequence targeted for editing, the targeting guide RNA sequence (if applicable) and how the editor was applied.*
Authentication	*Describe any authentication procedures for each seed stock used or novel genotype generated. Describe any experiments used to assess the effect of a mutation and, where applicable, how potential secondary effects (e.g. second site T-DNA insertions, mosiacism, off-target gene editing) were examined.*
