# Supplementary material for: Protein thiol alterations drive pathologic liquid–liquid phase separation in the aging brain
Source: Nat Struct Mol Biol. 2026 Jul 30;33(8):1252–65. doi: 10.1038/s41594-026-01857-w (PMC13468144; doi:10.1038/s41594-026-01857-w)
Supplement: Supplementary file 8 [file 41594_2026_1857_MOESM3_ESM.pdf]

nature portfolio

Peer Review File

Protein thiol alterations drive pathologic liquid-liquid phase separation in the aging brain

Corresponding Author: Professor Milos Filipovic

Version 0:

Decision Letter:

13th Jun 2025

Dear Professor Filipovic,

I am writing on behalf of my colleague Dr Kat Ciazynska, who is out of the office.

Thank you again for submitting your manuscript "Protein thiol alterations drive pathologic liquid-liquid phase separation in the aging brain". We now have comments (below) from the 3 reviewers who evaluated your paper. In light of those reports, we remain interested in your study and would like to see your response to the comments of the referees in the form of a revised manuscript.

You will see that the reviewers found the work interesting but indicated that further work was needed to deepen and strengthen the mechanistic analyses as well as the relevance to aging. We have discussed the referees' points in detail editorially and find them valid and significant. Addressing all the reviewers' technical and experimental remarks is in our view necessary to convince readers of your conclusions.

We should stress that the referees' concerns about the conclusions, mechanistic studies, and relevance to aging would need to be addressed thoroughly experimentally and in full, and reconsideration of the study for this journal and re-engagement of the referees will depend on the strength of these revisions as assessed editorially at resubmission.

We are committed to providing a fair and constructive peer-review process, and our standard revision process is about 6 months. Please do not hesitate to contact us to discuss the reviews or if you anticipate any issues or delays.

If you cannot send your revision within 6 months, please let us know. We will be happy to consider your revision as long as nothing similar has been accepted for publication at NSMB or published elsewhere. Should your manuscript be substantially delayed without notifying us in advance and your article is eventually published, the received date would be that of the revised, not the original, version.

Lastly, while we are interested in your manuscript and would like to see a revision, we appreciate that the revisions requested are extensive. If you find that the level of revision required to address the reviews and resubmit to our journal is beyond what you wish to carry out, please let us know and we would help you withdraw the paper from consideration at our journal and we could consult with our sister journals to determine whether transfer of the manuscript might be more suitable to your plans.

As you already know, we put great emphasis on ensuring that the methods and statistics reported in our papers are correct and accurate. As such, if there are any changes that should be reported, please submit an updated version of the Reporting Summary along with your revision.

Reporting Summary:
https://www.nature.com/documents/nr-reporting-summary.pdf

Please note that the form is a dynamic 'smart pdf' and must therefore be downloaded and completed in Adobe Reader.

When submitting the revised version of your manuscript, please pay close attention to our href="https://www.nature.com/nature-portfolio/editorial-policies/image-integrity">Digital Image Integrity Guidelines. and to the following points below:

-- that unprocessed scans are clearly labelled and match the gels and western blots presented in figures. Please note that all key data shown in the main figures as cropped gels or blots should be presented in uncropped form, with molecular weight markers. While these data can be displayed in a relatively informal style, they must refer back to the relevant figures. These data should be submitted as source data with the last revision, prior to acceptance.
-- that control panels for gels and western blots are appropriately described as loading on sample processing controls
-- all images in the paper are checked for duplication of panels and for splicing of gel lanes.
-- For any revision that includes light microscopy data, we ask our authors to please include a completed light microscopy reporting table [https://www.nature.com/documents/Light_microscopy_reporting_table.xlsx] to ensure the methods are described thoroughly. The table will be available to reviewers and ultimately published should the manuscript be accepted at the journal.

EXTENDED DATA FIGURES

When re-submitting your manuscript, please ensure that any supplementary figures and tables that are crucial to the manuscript's conclusions are converted into Extended Data figures and tables to increase visibility of these data. Extended Data figures and tables are online-only (present in the online PDF and full-text HTML versions of the paper), peer-reviewed display items that provide essential background to the article but are not included in the main article due to space constraints. A maximum of ten Extended Data display items (figures and tables) is permitted.

Finally, please ensure that you retain unprocessed data and metadata files after publication, ideally archiving data in perpetuity, as these may be requested during the peer review and production process or after publication if any issues arise.

If there are additional or modified structures presented in the final revision, please submit the corresponding PDB validation reports.

SOURCE DATA: we urge authors to provide, in tabular form, the data underlying the graphical representations used in figures. This is to further increase transparency in data reporting, as detailed in this editorial (http://www.nature.com/nsmb/journal/v22/n10/full/nsmb.3110.html). Spreadsheets can be submitted in excel format. Only one (1) file per figure is permitted; thus, for multi-paneled figures, the source data for each panel should be clearly labeled in the Excel file; alternately the data can be provided as multiple, clearly labeled sheets in an Excel file. When submitting files, the title field should indicate which figure the source data pertains to. We encourage our authors to provide source data at the revision stage, so that they are part of the peer-review process.

While we encourage the use of color in preparing figures, please note that this will incur a charge to partially defray the cost of printing. Information about color charges can be found at http://www.nature.com/nsmb/authors/submit/index.html#costs

We require deposition of coordinates (and, in the case of crystal structures, structure factors) into the Protein Data Bank with the designation of immediate release upon publication (HPUB). Electron microscopy-derived density maps and coordinate data must be deposited in EMDB and released upon publication. Deposition and immediate release of NMR chemical shift assignments are highly encouraged. Deposition of deep sequencing and microarray data is mandatory, and the datasets must be released prior to or upon publication. To avoid delays in publication, dataset accession numbers must be supplied with the final accepted manuscript and appropriate release dates must be indicated at the galley proof stage. Please find the complete NRG policies on data availability at http://www.nature.com/authors/policies/availability.html.

Nature Structural & Molecular Biology is committed to improving transparency in authorship. As part of our efforts in this direction, we are now requesting that all authors identified as 'corresponding author' on published papers create and link their Open Researcher and Contributor Identifier (ORCID) with their account on the Manuscript Tracking System (MTS), prior to acceptance. This applies to primary research papers only. ORCID helps the scientific community achieve unambiguous attribution of all scholarly contributions. You can create and link your ORCID from the home page of the MTS by clicking on 'Modify my Springer Nature account'. For more information please visit please visit www.springernature.com/orcid.

Please use the link below to submit your revised manuscript and related files:

Link Redacted

Note: This URL links to your confidential home page and associated information about manuscripts you may have submitted, or that you are reviewing for us. If you wish to forward this email to co-authors, please delete the link to your homepage.

We look forward to seeing the revised manuscript and thank you for the opportunity to review your work.

Sincerely,

Melina Casadio, PhD
Locum Chief Editor, Nature Structural & Molecular Biology
ORCID ID: https://orcid.org/0000-0003-2389-2243

Referee expertise:

Referee #1: redox biology, proteomics

Referee #2: protein aggregation, neurodegeneration

Referee #3: condensate biology

Reviewers' Comments:

Reviewer #1 (Remarks to the Author):

This proof-of-concept study seeks to uncover a redox regulatory mechanism linking age-dependent cysteine post-translational modifications (PTMs)—specifically increased sulfenylation (PSOH)/sulfonylation (PSO_3H) and decreased persulfidation (PSSH)—to pathological liquid-liquid phase separation (LLPS) in the brain. Overall, this study is a technical tour de force by using many state-of-the-art proteomics methods in aging mice and CSE-knockout models. Notably, it provides the first evidence that specific cysteine PTMs directly regulate LLPS of neuronal proteins. The authors demonstrate that PSSH loss promotes aberrant LLPS, stress granule persistence, protein aggregation, and synaptic impairment, while H_2S donors rescue these defects—highlighting therapeutic potential. However, several limitations temper enthusiasm for the current manuscript.

Major Concerns:
1. While extensive redox proteomics datasets are generated, the bioinformatic analysis does not fully explore the combinatorial complexity of cysteine PTMs. Mapping concurrent PSOH and PSSH changes on the same proteins during aging and in CSE-KO models would clarify their crosstalk and collective impact on the "thiol redoxome." This is essential to establish how global thiol remodeling drives age-related LLPS dysregulation.
2. In vitro LLPS experiments rely on crowding agents (PEG) and purified proteins. These conditions may not accurately replicate in vivo triggers, composition, or functional consequences of neuronal condensates. Key concerns include: 1) The extremely low stoichiometry of endogenous PSO_3H raises questions about its sufficiency to drive pathogenic LLPS in aging brains. Direct in vivo evidence linking specific cysteine hyperoxidation to condensate formation/aggregation is needed. 2) The efficacy of exogenous H_2S or H_2S donors to significantly increase site-specific PSSH occupancy in vivo requires further validation to support therapeutic relevance.
3. The protocols for generating specific cysteine PTMs (PSO_3H, PSSH) on purified proteins lack thorough validation: 1) Quantitative conversion of thiols to PSO_3H using H_2O_2 is unlikely. Residual disulfides or sulfinic acid (PSO_2H) could confound results. 2) Pre-formed PSO_2H/PSO_3H are irreversible by reductants like DTT, potentially skewing comparisons with reduced/PSSH forms. 3) Clarifying the exact chemical composition and yield of the modified proteins used in LLPS assays is critical for interpreting the observed effects.
4. The study establishes that PSSH inhibits LLPS but offers limited insight into the biophysical mechanism (e.g., conformational changes, altered hydrophobicity, charge modulation) compared to the detailed analysis of sulfonylation's effects. Understanding how persulfidation dissolves/prevents condensates is fundamental.

Minor Concerns & Clarifications:
1. Clarify the rationale for focusing specifically on the frontotemporal cortex.
2. Address potential artifactual oxidation during sample preparation for the 645 sulfonylated peptides detected without enrichment. Comment on their reproducibility.
3. Figure 1j: The comparison of mouse PSSH data with human cell line data (Fu et al.) requires justification regarding cross-species relevance.
4. Figure 2i: The relevance of the survival curve to the core LLPS/redox mechanisms could be strengthened. Otherwise, this can be removed.
5. Figures 4f,g: Including H_2S donor (e.g., GYY4137) treatment in these MEF experiments (mirroring Fig. 4c) would directly test its role in resolving protein condensates.
6. Figures reporting undetectable PSO_3H in controls (e.g., Fig. 2g, 3c, Extended Data Fig. j,k,m,n) should acknowledge limitations of DDA proteomics' stochastic nature.
7. Extended Data Fig. 6c: Clarify the chemical identity denoted by "SO-IAM" and its origin. Is this a product from the side-reaction between PSOH and IAM? Or shall this be a sulfoxide product from the initial PSH-IAM conjugate.
8. Extended Data Fig. 10a: The marginal FT-IR change for PSO_3H-GAPDH seems inconsistent with strong LLPS effects; please comment.
9. Methods: Provide details on MS1-based quantification parameters; Specify software and key settings for Persulfidome data analysis; Report if any BTD-modified (+418.1311 Da) sites were identified.

Reviewer #2 (Remarks to the Author):

In this study, the authors used advanced chemoproteomic techniques to investigate cysteine PTMs in the mouse brain. They find age-related increases in thiol oxidation promote the formation of biomolecular condensates, leading to protein

aggregation and protein persulfidation inhibits biomolecular condensation. They also find mice deficient in cystathionine g-lyase (CSE), exhibit reduced lifespans and spontaneously develop neurofibrillary tangles with age. This is an interesting study with both technical and conceptual innovations. However, the major issue is that in multiple places the data didn't really support the conclusion. Following are my comments for the authors to consider:

1. In Fig 1l, there seems to be no consistent decrease of PSSH in large amount of proteins in the aged mice. This makes the overall correlation of decreased PSSH with aging questionable.
2. The neurofibrillary tangle shown in Fig 2j doesn't look like neurofibrillary tangle at all. This observation needs to be confirmed using many of commercially available antibodies against pathological tau.
3. The data shown in Figure 4e didn't demonstrate "more pronounced phase separation" for glycolytic enzymes. It only indicates that these enzymes are in different protein/molecule complex. Also, the changes shown in Figure 4b&e are very small, without statistical significance.
4. The data shown in Figure 4f&g need to be quantified。
5. The authors claimed "an approximately 3-fold increase in GAPDH within the aggregated fraction of Cse-/- lysates" based on the data shown in Figure 5l. However, based on the figure， there is a dramatic decrease of soluble GAPDH rather than increase of insoluble GAPDH in Cse-/- mice.

Reviewer #3 (Remarks to the Author):

The ambitious work by Vigane et al seeks to test a new hypothesis that thiol modifications change liquid-liquid phase separation in a pathological manner in the brain. The authors perform an extensive proteome wide identification of PSOH and PSSH in the frontotemporal mouse brain vs aging using some new tools to specifically identify the modified forms, finding changes with age. Using a CSE-/- mouse that should disrupt persulfidation, they show changes in the proteomic modifications and in phenotype. Modified proteins are predicted to have biophysical impacts. Enhanced formation of PSO3H is also probed for the CSE ko. Then the authors shift to examining two specific targets. For synapsin1, they show that its modification levels change with aging and in the CSE ko, the modification found should have some effect on the structure and it has an impact on the phase separation in recombinant protein assays as well as some data on neurotransmitter release. This led the authors to examine potential phase separating proteins in the proteome including G3BP, followed by GAPDH. For GAPDH, they examine the effect of the modifications on phase separation and biochemical activity as well as aggregation biochemically and in the mouse brain. Overall the work is intriguing and expansive but a number of major issues prevent it from being robust and convincing is several areas. The proteomic evaluations seems a large strength, but the candidate data overall is not clear and the biochemical experiments have issues in design and lack proper controls as well as evidence of mechanism. I see the proteomic experiments are exciting but do not think the biochemical experiment fit well.

Major

Do the authors have any explanation for why the proteins that were found only by other groups in Fig 1j and Supplemental Fig. 7a/b were not found in this study?

The mechanistic insight provided by the phase separation experiments needs to be improved extensively. The effect of the modifications on synapsin1 droplet area is very small. I understand that the authors have performed a replicate experiment with independently "prepared" samples. Those data should be presented. A wetting change also means that the droplet area is not the correct measure. As oxidation can cause aggregation and loss of soluble protein, even the figure 3g could be reporting on something other than changes due to cysteine adducts. The attempt to use hydrogen peroxide alone as a control for methionine oxidation is confusing as cysteine is oxidized by hydrogen peroxide also, so I do not follow the logic. The GFP could also be oxidized. The authors should find out where their biochemical protein is being oxidized. The authors really need to make variants with all surface and specific cysteines changed to alanine (or something else to prevent oxidation) and show what effect that has on the mass spec before and after treatment (to show what is really happening) and then on the phase separation (nothing should happen if the cysteines are no longer present) as it is possible the conditions themselves (slightly changes in pH etc) alter the phase separation. This would also help with mechanism. Further, the authors should mimic the oxidation perhaps by seeing if methionine is a mimic for cysteine SSH (or a reaction with cysteine with MTS to make SSCH3) and with an acidic residue to mimic SOH (is it expected to be deprotonated at this pH? the one reference I found says pKa is 6.1 so perhaps it is deprotonated). This would then need to be expanded to try to understand why the change in the residue causes the change in phase separation (if a similar change was made at another site, would the same change in phase separation and wetting occur?)

The "shift toward higher-density fractions" observed could be due to formation of cysteine disulfide forms or other cross linked forms, aggregates, etc. Even the in vitro experiments are a concern as the formation of cross linked forms definitely happens when hydrogen peroxide is added to proteins, so I am really not sure how to interpret this. Much more data could be collected on the Cse -/- samples by using chromatography separation, native gel electrophoresis / western, SDS-PAGE (without and with reducing agent) / western

Is the modification causing the aggregation or behavior or are proteins modified only as a consequence of destabilization (are they in the process of being disposed of)? In cell or mouse expression and analysis of modification of variants where the folds are disrupted should be examined.

The dissolution of SGs by H2S is interesting but I am not convinced that the mechanism suggested is the only means of

achieving this observation. A lot of metabolic changes could be occurring. Additionally, the use of oxidative stress to generate the stress granules and then applying other changes to oxidation is problematic. Inducing stress granules with heat, osomotic shock, etc would be important to show then the effect of H2S.

GAPDH phase separation could be an in vitro artifact if it requires so much PEG. Is the PEG entering the droplets / does it phase separate with dextran or ficoll, or a protein crowder? If it doesn't phase separate without a crowder, do we know if the phase separation is relevant?

The GAPDH, like the synapsin, has the same issues for the biochemical phase separation. Control experiments and a mechanism needs to be proposed and probed and demonstrated using mutations that prevent and mimic the effects of the modification. The modification sites and numbers need to be examined by mass spectrometry (total and site specific).

GAPDH has an active site cysteine – is that being modified? The assay probing the "in droplet" activity is very confusing as the remaining "bulk" form is missing whatever amount of protein is in the droplet, so the comparison is not at all "fair" (unless I misunderstand, there is not the same amount of protein). Even if the amount is the same (are the authors measuring the concentration and then calculating the rates with that?), there are complicated transport questions about droplets (what droplet size) and those are not addressed with this methodology. That the droplets have only 1.5% to 2% of volume (how is that measured?) does not mean that they have a small amount of the protein, they may have a large fraction of the total enzyme. Hence the H2O2 treated experiments are even harder to interpret. There is not in my opinion any evidence to show conformation change that increases catalytic activity.

The C245 modeling may be interesting but again mutations should be made to test this hypothesis. The assembly state (covalent and non-covalent) of the protein should be tested not by FTIR but by chromatographic separation, dynamic light scattering, mass photometry, gels (as described above). The authors present the differences upon RNA addition as a surprise but much more thorough characterization of the samples should be performed.

Minor
The section title "Biomolecular condensation of …. Enhanced in aged Cse-/- mice" should say the cortex or the region.

What is the reference for " LLPS of GAPDH has been shown to .."

Fig. 5a: Did the authors look at any other disorder prediction software or previous literature to confirm the disorder content of GAPDH seen with CD-Code?

Version 1:

Decision Letter:

Our ref: NSMB-A51045A

13th Mar 2026

Dear Dr. Filipovic,

Thank you for submitting your revised manuscript "Protein thiol alterations drive pathologic liquid-liquid phase separation in the aging brain" (NSMB-A51045A). It has now been seen by the original referees and their comments are below.

I am sincerely sorry for the delay in this decision.

You will see that reviewer #3 continues to bring up persistent concerns. Considering the comments of the other reviewers, and strengths of the papers even despite the lack of in-depth mechanism requested by reviewer #3, we consulted reviewers #1 and #2 to ask for their input regarding the comments of reviewer #3. This was specifically regarding technical points about pKa values reported, and the identity of the tangles. These comments are marked as [ADDITIONAL COMMENTS] in the reports below.

In some cases, we engage in consultations like this to increase in the quality of peer review at the journal, and to lead to more informed editorial decisions, and better guidance for authors. I am sorry this has led to some delay, however we found it necessary to be able to reach this decision.

Informed by the review process, I am writing that we have decided to in principle publish the revised manuscript in Nature Structural & Molecular Biology, pending minor textual revisions to satisfy the referees' final requests and to comply with our editorial and formatting guidelines.

We also appreciate that some of the data on the tangles is being reserved for the related publication, which you have shared with us and the reviewers during the process. To increase transparency in this aspect, we suggest that you consider opting into transparent peer review, and having the reviewer reports published alongside the manuscript online.

We are now performing detailed checks on your paper and will send you a checklist detailing our editorial and formatting requirements in about 2-3 weeks. Please do not upload the final materials and make any revisions until you receive this

additional information from us.

To facilitate our work at this stage, it is important that we have a copy of the main text as a word file. If you could please send along a word version of this file as soon as possible, we would greatly appreciate it; please make sure to copy the NSMB account (cc'ed above).

Thank you again for your interest in Nature Structural & Molecular Biology Please do not hesitate to contact me if you have any questions.

Sincerely,

Katarzyna Ciazynska, PhD
(she/her)
Senior Editor
Nature Structural & Molecular Biology
https://orcid.org/0000-0002-9899-2428

Reviewer #1 (Remarks to the Author):

The authors have addressed all my concerns. The manuscript at this version can be accepted as it is, at least in principle.

[ADDITIONAL COMMENTS]
I agree with the concerns raised by Reviewer #3. The predicted apparent pKa values of approximately -2 and -6 for these cysteines are exceptionally low. While negative pKa values are chemically possible for extremely strong acids, they are highly unusual for cysteine thiols in a biological context. My main concern is that these values are derived from computational predictions rather than experimental validation. Given how unusual these numbers are, it is difficult to assess whether they represent a genuine biochemical insight or if they might result from the specific parameters or limitations of the prediction algorithm (or possibly even a typographical error in the figure). Using such predictions as mechanistic evidence without experimental confirmation weakens that specific argument. However, I believe this result could be simply removed without invalidating this interesting and comprehensive study.

Reviewer #2 (Remarks to the Author):

All my concerns are addressed. I don't have further comments.

[ADDITIONAL COMMENTS]
I do share the same concern as reviewer #3. I think confirming the tau pathology is a very critical point in this paper. I wrote in my initial comments “The neurofibrillary tangle shown in Fig 2j doesn’t look like neurofibrillary tangle at all. This observation needs to be confirmed using many of commercially available antibodies against pathological tau.”

However, the author responded “The reviewer is right that staining with tau antibodies is the best way to show the neurofibrillary tangles. Gallaya’s staining (with all its limitations) has been in use in confirming postmortem AD from human samples long before the introduction of tau (Moloney et al, Visualization of neurofibrillary tangle maturity in Alzheimer’s disease: A clinicopathologic perspective for biomarker research, Azlheimer & Dementia, 2021). The reason we did not include tau is that in collaboration with Prof Paul we have another manuscript which is dealing with phenotyping of CSE ko mice (Chakraborty et al, 2025, accompanying study for reviewer only). We show that those mice not only have accumulation of tau fibrils but a full cognitive phenotype reminiscent of AD and/or dementia.”

In this context, I can accept this response and the data provided, but want to stress that some of the most important data of the study is saved for another publication.

Reviewer #3 (Remarks to the Author):

“In both G3BP2 and synapsin-1, CyspKa predicts exceptionally low apparent pKa values for their redox-active cysteines (≈ −2 for G3BP2 C73 and ≈ −6 for synapsin-1 C360) (Supplementary Fig. 5), indicating that these residues occupy highly thiolate-stabilized reactive hotspots.”
This seems like an unrealistic pKa (negative numbers) and so it is not clear if this is reliable. Furthermore, the authors repeat several times that “the reaction rate for the oxidation of cysteine is approximately two orders of magnitude faster than that for methionine” but this ignores that structure and context could dramatically affect reaction rate (if pKa shifts from pH ~7.8 to pH -6, 12 orders of magnitude due to hotspots, the reaction rate will also be highly dependent on details).

The biggest issue is that G3BP2 is a good idea but the example shows that the mechanism, which would be required for this

journal in my view, is not at all probed or explained by the data. The pKa being negative basically means this is a buried cysteine. So what does modification here do to the protein? Some biophysical and structural evaluation of the changes (circular dichroism, NMR spectroscopy, molecular simualtions) caused by distinct modifications could be done (the modifications could be made in the recombinant protein). With a single cysteine, lots of different modifications could be explored in addition to the modifications that are made by the various oxidation states. Also, the idea that mutations to the 20 natural amino acids cannot cannot capture all the dimensions of the cysteine modifications is true, but surely they will capture some of the changes. Either way, the data are not adequate to support the claims.

The neurofibrillary tangles are still not stained as requested by reviewer to confirm their identity.

Version 2:

Decision Letter:

2nd Jul 2026

Dear Dr. Filipovic,

We are now happy to accept your revised paper "Protein thiol alterations drive pathologic liquid-liquid phase separation in the aging brain" for publication as an Article in Nature Structural & Molecular Biology.

Acceptance is conditional on the manuscript's not being published elsewhere and on there being no announcement of this work to the newspapers, magazines, radio or television until the publication date in Nature Structural & Molecular Biology.

Over the next few weeks, your paper will be copyedited to ensure that it conforms to Nature Structural & Molecular Biology style. Once your paper is typeset, you will receive an email with a link to choose the appropriate publishing options for your paper and our Author Services team will be in touch regarding any additional information that may be required.

After the grant of rights is completed, you will receive a link to your electronic proof via email with a request to make any corrections within 48 hours. If, when you receive your proof, you cannot meet this deadline, please inform us at rjsproduction@springernature.com immediately.

You will not receive your proofs until the publishing agreement has been received through our system.

Due to the importance of these deadlines, we ask that you please let us know now whether you will be difficult to contact over the next month. If this is the case, we ask you provide us with the contact information (email, phone and fax) of someone who will be able to check the proofs on your behalf, and who will be available to address any last-minute problems.

To assist our authors in disseminating their research to the broader community, our SharedIt initiative provides all co-authors with the ability to generate a unique shareable link that will allow anyone (with or without a subscription) to read the published article. Recipients of the link with a subscription will also be able to download and print the PDF.

As soon as your article is published, you can generate your shareable link by entering the DOI of your article here: http://authors.springernature.com/share. Corresponding authors will also receive an automated email with the shareable link

Note the policy of the journal on data deposition: http://www.nature.com/authors/policies/availability.html.

Your paper will be published online soon after we receive proof corrections and will appear in print in the next available issue. You can find out your date of online publication by contacting the production team shortly after sending your proof corrections.

You may wish to make your media relations office aware of your accepted publication, in case they consider it appropriate to organize some internal or external publicity. Once your paper has been scheduled you will receive an email confirming the publication details. This is normally 3-4 working days in advance of publication. If you need additional notice of the date and time of publication, please let the production team know when you receive the proof of your article to ensure there is sufficient time to coordinate. Further information on our embargo policies can be found here: https://www.nature.com/authors/policies/embargo.html

You can now use a single sign-on for all your accounts, view the status of all your manuscript submissions and reviews, access usage statistics for your published articles and download a record of your refereeing activity for the Nature journals.

If you have not already done so, we strongly recommend that you upload the step-by-step protocols used in this manuscript to the Protocol Exchange. Protocol Exchange is an open online resource that allows researchers to share their detailed

experimental know-how. All uploaded protocols are made freely available, assigned DOIs for ease of citation and fully searchable through nature.com. Protocols can be linked to any publications in which they are used and will be linked to from your article. You can also establish a dedicated page to collect all your lab Protocols. By uploading your Protocols to Protocol Exchange, you are enabling researchers to more readily reproduce or adapt the methodology you use, as well as increasing the visibility of your protocols and papers. Upload your Protocols at www.nature.com/protocolexchange/. Further information can be found at www.nature.com/protocolexchange/about.

An online order form for reprints of your paper is available at https://www.nature.com/reprints/author-reprints.html. Please let your coauthors and your institutions' public affairs office know that they are also welcome to order reprints by this method.

Authors may need to take specific actions to achieve compliance with funder and institutional open access mandates. If your research is supported by a funder that requires immediate open access (e.g. according to Plan S principles or the NIH public access policy) then you should select the gold OA route, and we will direct you to the compliant route where possible. Because authors warrant under our subscription licensing terms that they haven’t committed to licensing any version of their article under a licence inconsistent with the terms of our agreement – including the applicable embargo period – publication under the subscription model isn’t suitable for authors whose funders require no embargo.

In approximately 10 business days you will receive an email with a link to choose the appropriate publishing options for your paper and our Author Services team will be in touch regarding any additional information that may be required.

You will not receive your proofs until the publishing agreement has been received through our system.

If you have any questions about our publishing options, costs, Open Access requirements, or our legal forms, please contact ASJournals@springernature.com

Sincerely,

Katarzyna Ciazynska, PhD
(she/her)
Senior Editor
Nature Structural & Molecular Biology
https://orcid.org/0000-0002-9899-2428

Click here if you would like to recommend Nature Structural & Molecular Biology to your librarian:
http://www.nature.com/subscriptions/recommend.html#forms

Open Access This Peer Review File is licensed under a Creative Commons Attribution 4.0 International License, which

permits use, sharing, adaptation, distribution and reproduction in any medium or format, as long as you give appropriate credit to the original author(s) and the source, provide a link to the Creative Commons license, and indicate if changes were made.
In cases where reviewers are anonymous, credit should be given to 'Anonymous Referee' and the source.
The images or other third party material in this Peer Review File are included in the article's Creative Commons license, unless indicated otherwise in a credit line to the material. If material is not included in the article's Creative Commons license and your intended use is not permitted by statutory regulation or exceeds the permitted use, you will need to obtain permission directly from the copyright holder.
To view a copy of this license, visit https://creativecommons.org/licenses/by/4.0/

Reviewers' Comments:

Reviewer #1 (Remarks to the Author):

This proof-of-concept study seeks to uncover a redox regulatory mechanism linking age-dependent cysteine post-translational modifications (PTMs)—specifically increased sulfenylation (PSOH)/sulfonylation (PSO_3H) and decreased persulfidation (PSSH)—to pathological liquid-liquid phase separation (LLPS) in the brain. Overall, this study is a technical tour de force by using many state-of-the-art proteomics methods in aging mice and CSE-knockout models. Notably, it provides the first evidence that specific cysteine PTMs directly regulate LLPS of neuronal proteins. The authors demonstrate that PSSH loss promotes aberrant LLPS, stress granule persistence, protein aggregation, and synaptic impairment, while H_2S donors rescue these defects—highlighting therapeutic potential. However, several limitations temper enthusiasm for the current manuscript.

We thank the reviewer for these encouraging comments.

Major Concerns:

1. While extensive redox proteomics datasets are generated, the bioinformatic analysis does not fully explore the combinatorial complexity of cysteine PTMs. Mapping concurrent PSOH and PSSH changes on the same proteins during aging and in CSE-KO models would clarify their crosstalk and collective impact on the "thiol redoxome." This is essential to establish how global thiol remodeling drives age-related LLPS dysregulation.

We are grateful to the reviewer for this insightful comment, which motivated us to advance the project beyond our initial plans and develop an online platform for interactive data exploration:
https://aging-redox-proteomics-viewer.streamlit.app/
The site is currently password-protected (username: reviewer.app; password: Ycql59H3s621).
This platform allows users to search proteins, examine their individual aging PSOH and PSSH profiles (including changes observed in CSE knockout animals), and select pathways of interest across datasets. We also integrated direct links to the UniProt database, enabling users to access detailed protein information, including structural data. In addition to creating this interactive resource, we have added double plots for all proteins whose PSOH and PSSH profiles change significantly with aging. They are now part of Extended Data Figure 3.

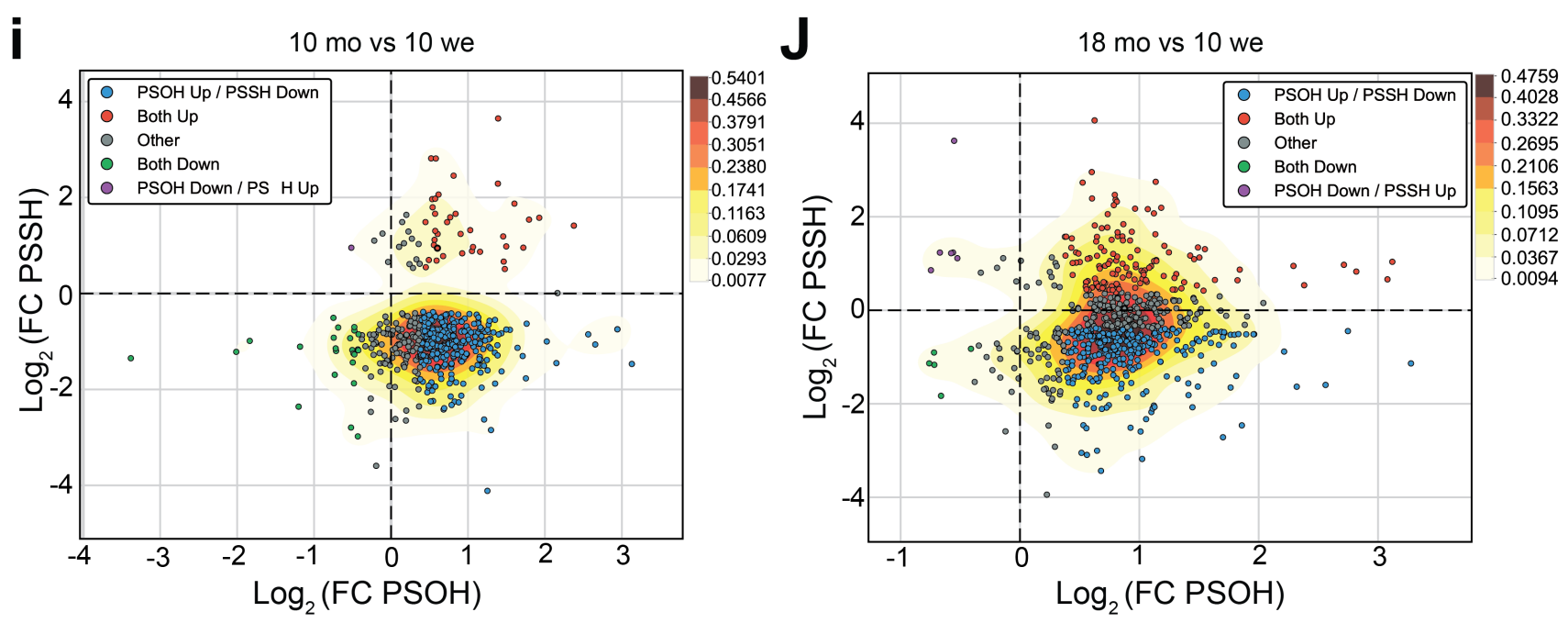

Extended Data Fig 3.

We have also incorporated the following sentences into the revised manuscript:
"An ~ 37 % overlap between proteins found to change their PSOH and PSSH with aging is observed (Extended Data Fig. 3h) with majority of those showing a decline of PSSH and an increase of PSOH (Extend Data Fig. 3i,j), in agreement with reduced transsulfuration pathway and increased ROS observed in aging. To make this complex data analysis easily accessible, we generated an interactive platform that allows comparison across different PTMs and ages

and integrates protein search with pathway visualization (https://aging-redox-proteomics-viewer.streamlit.app/).”

2. In vitro LLPS experiments rely on crowding agents (PEG) and purified proteins. These conditions may not accurately replicate in vivo triggers, composition, or functional consequences of neuronal condensates.

PEG is widely used as a crowding agent in LLPS assays, including the seminal study on synapsin phase separation (Milovanovic et al., Science 339, 2013, PMID: 29976799), so we employed PEG to maintain direct comparability with published data. By contrast, GAPDH's phase separation can be driven both by PEG and by RNA. To address the reviewer 3's concerns we now replaced GAPDH experiments with experiments on G3BP2, the principal stress-granule nucleator (see our response to Reviewer 3). G3BP2 phase separation is usually triggered by interaction with RNA, which is what we used (Yang et al., Cell 181, 2020, PMID: 32302571). These assays include a wider range of in-vivo experiments strengthening our in-vitro observations.

Key concerns include: 1) The extremely low stoichiometry of endogenous PSO_3H raises questions about its sufficiency to drive pathogenic LLPS in aging brains. Direct in vivo evidence linking specific cysteine hyperoxidation to condensate formation/aggregation is needed.

Quantifying the absolute stoichiometry of cysteine PTMs is inherently difficult and in strict chemical terms impossible (see ***Supplementary Text*** *in* ***Supplementary material*** *file), since TCEP reduction only captures a subset of modifications (e.g., PSO_xH species remain invisible, as do other modification such as PSCN, electrophile-modified cysteines etc). We agree that global occupancy may be low but not extremely low, as even a few percent oxidation at typical cellular protein concentrations (~100 µM for GAPDH and synapsin) corresponds to low-micromolar levels of modified protein, more than sufficient to impact phase behaviour. We do identify and quantify synapsin-1 PSO_3H “in vivo” in aged brain samples of $Cse^{-/-}$ mice and estimate that this is ~3% of total thiol content (****Fig. 3d, Extended Data Fig. 6c****). Indeed, our phase diagrams demonstrate that just 1 µM synapsin-SO_xH undergoes LLPS with only 1 % PEG, whereas tenfold more reduced synapsin is required under identical conditions (Fig. 3h). Thus, even low-stoichiometry modification can drive condensation, which we believe represents an important mechanistic insight from this study. Sulfonylation does not necessarily cause aggregation of the modified protein directly; rather, by promoting LLPS and reducing the reversibility of condensate dissolution, it creates a microenvironment that can act as a nucleation site for protein aggregation.*
We would also like to clarify that PSO_3H is the terminal oxidation product, but PSO_2H would likely exert similar effects because of their comparable steric bulk and charge. In previous work, using tools for selective PSO_2H labelling developed by Kate Carroll's group (Akter et al., Nat Chem Biol 14, 2018, PMID: 30177848), we demonstrated that PSO_2H levels increase in aged human samples (Zivanovic et al., Cell Metab, 2019, PMID: 31735592).

The manuscript is now modified to read as follows: “Synapsin 1 is present at approximately 100 µM in nerve terminals.[1] Given the phase-diagram shift we observed for PSO_3H, sulfenylation of only about 3% of the protein population would be sufficient to drive the onset of phase separation.” We also added a sentence in Supplementary text: “Having observed that many proteins undergoing age-induced thiol remodelling also undergo LLPS highlights the importance of the local concentration of modified species, rather than global assessments of site occupancy, which may remain low.”

*Furthermore, as we now show new data on G3BP2 protein, we also include detection of PSO_3H in G3BP2 isolated from the MEF cells during stress and recovery experiment (****Supplementary Figure 6****).*

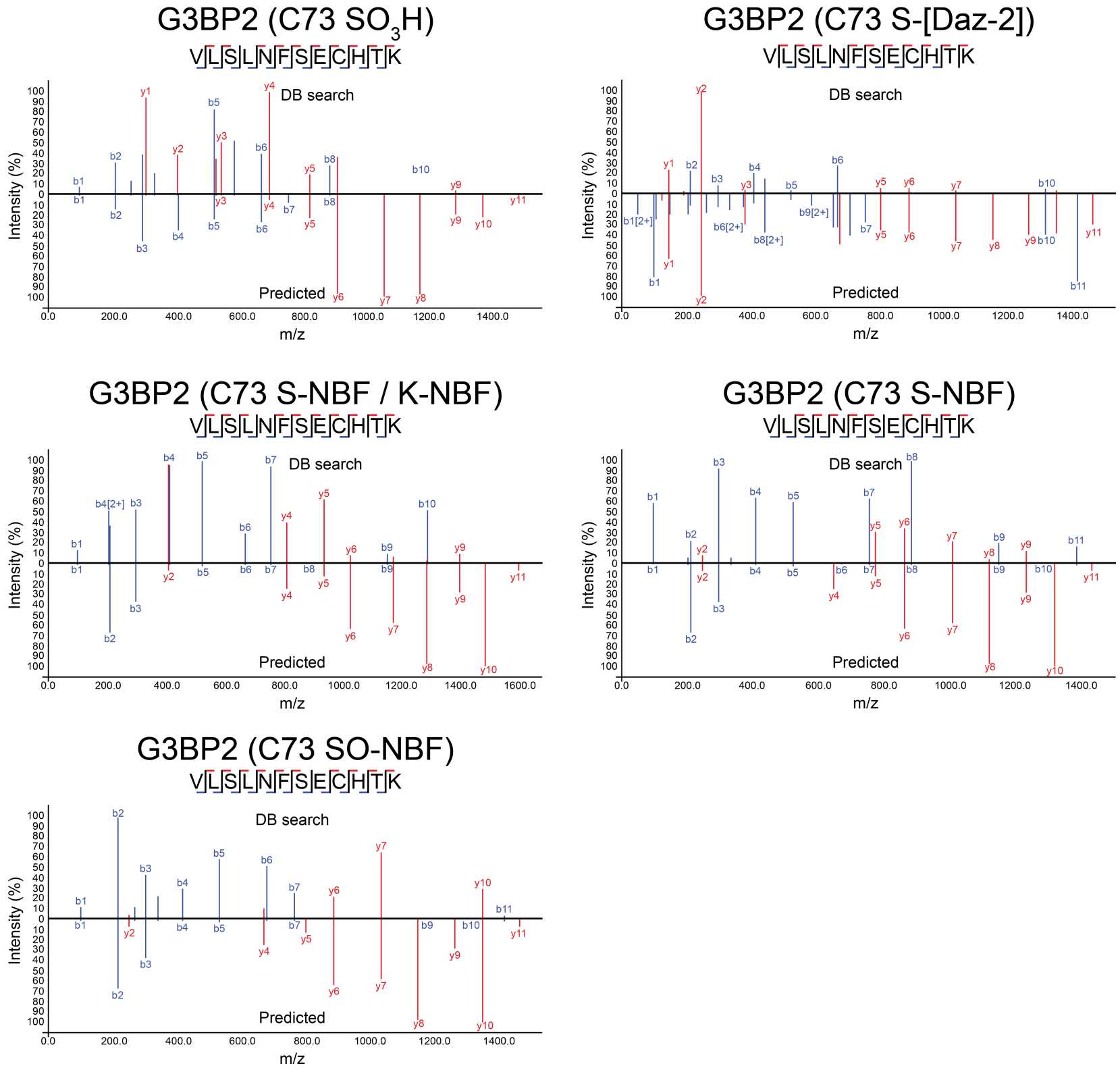

Supplementary Fig 6.

2) The efficacy of exogenous H_2S or H_2S donors to significantly increase site-specific PSSH occupancy in vivo requires further validation to support therapeutic relevance.

*We thank the reviewer for this comment. The ability of H_2S and donors of H_2S such as GYY4137 have been already shown to efficiently increase intracellular protein persulfidation (Zivanovic et al, Cell Metab, 2019, PMID: 31735592). Recently we also showed that ergothioneine can be used as an H_2S source, as it serves as an alternative substrate for CSE resulting in global PSSH increase (Petrovic et al. Cell Metab, 2025, PMID: 39842434). As we now included data on G3BP2, a single cysteine-containing protein, we first designed experiment to assess % of protein present as PSSH and PSOH in vivo, in brain samples (**Extended Data Fig 7h**).*

h

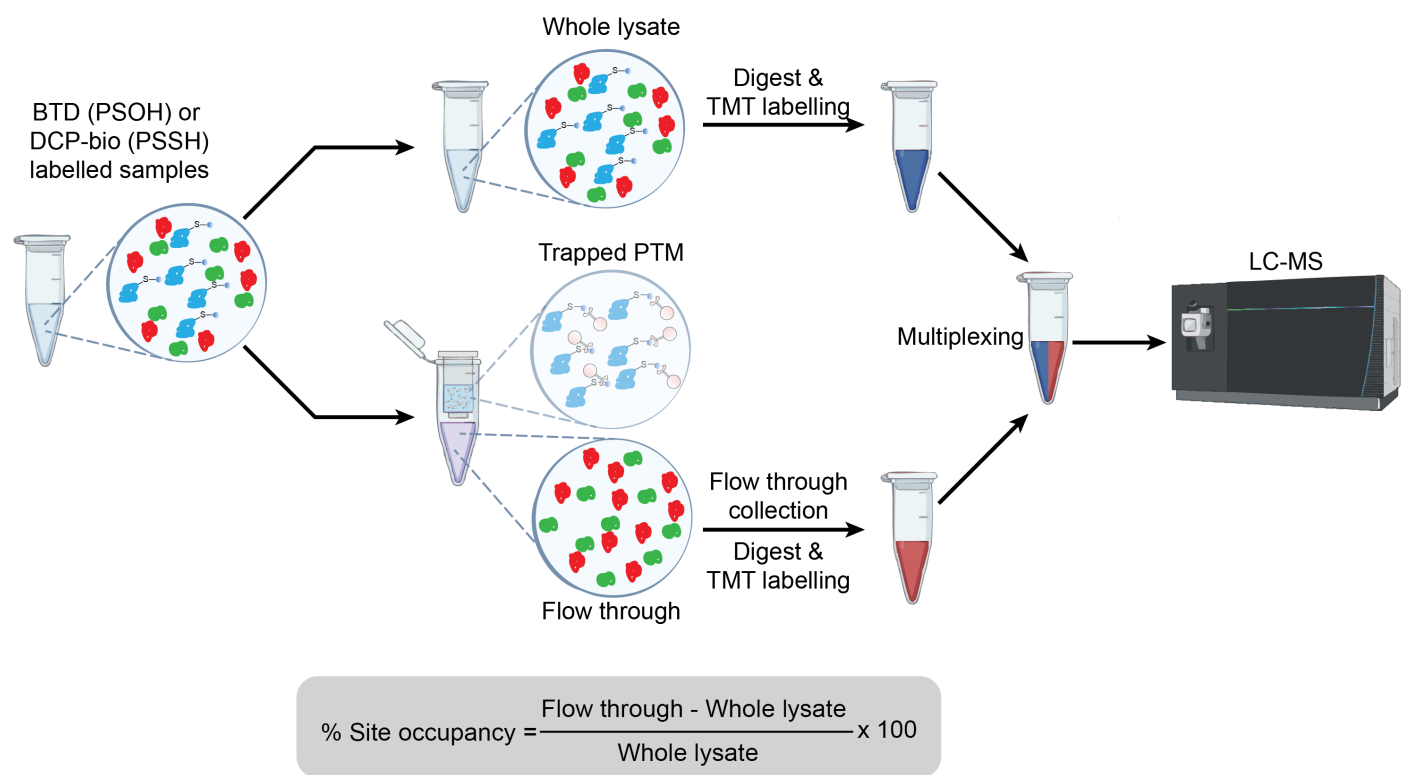

Extended Data Fig 7.

Instead of looking for persulfidated or sulfenylated peptide, we looked at fraction that was not labelled and pulled by enrichment. This difference gave us the % of protein labelled as PSOH or PSSH, the results of which are shown in Fig 4c. We also monitored the dynamic changes

*of PSSH during stress and recovery in MEFs and estimated site G3BP2 PSSH occupancy in those cells (**Figure 5b, Extended Data Fig. 9b,c**).*

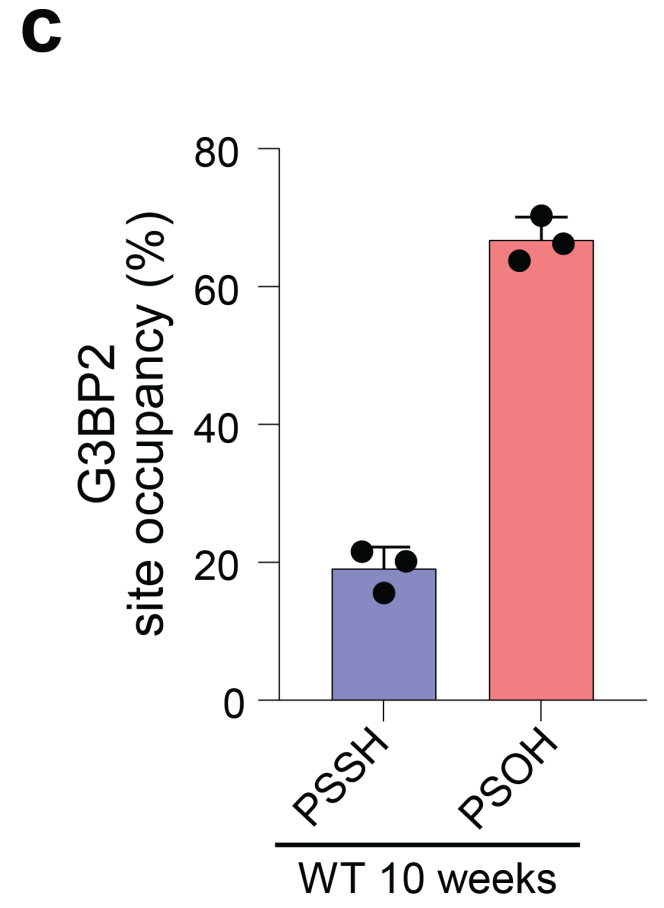

Figure 4.

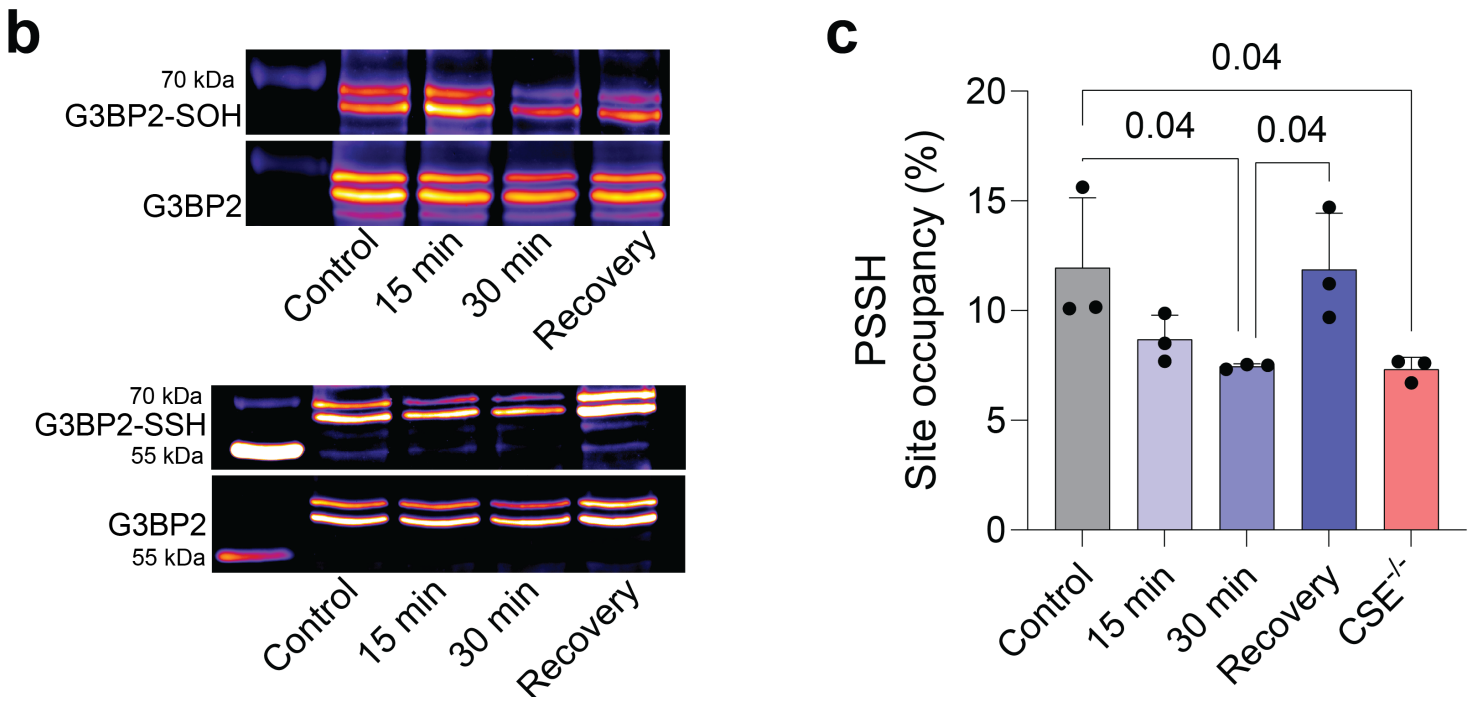

Extended Data Fig. 9

Furthermore, we used H_2S treatment to show in vivo dissolution of SG, that is absent in C73S mutant (Figure 5d, Supplementary Video 7-8).

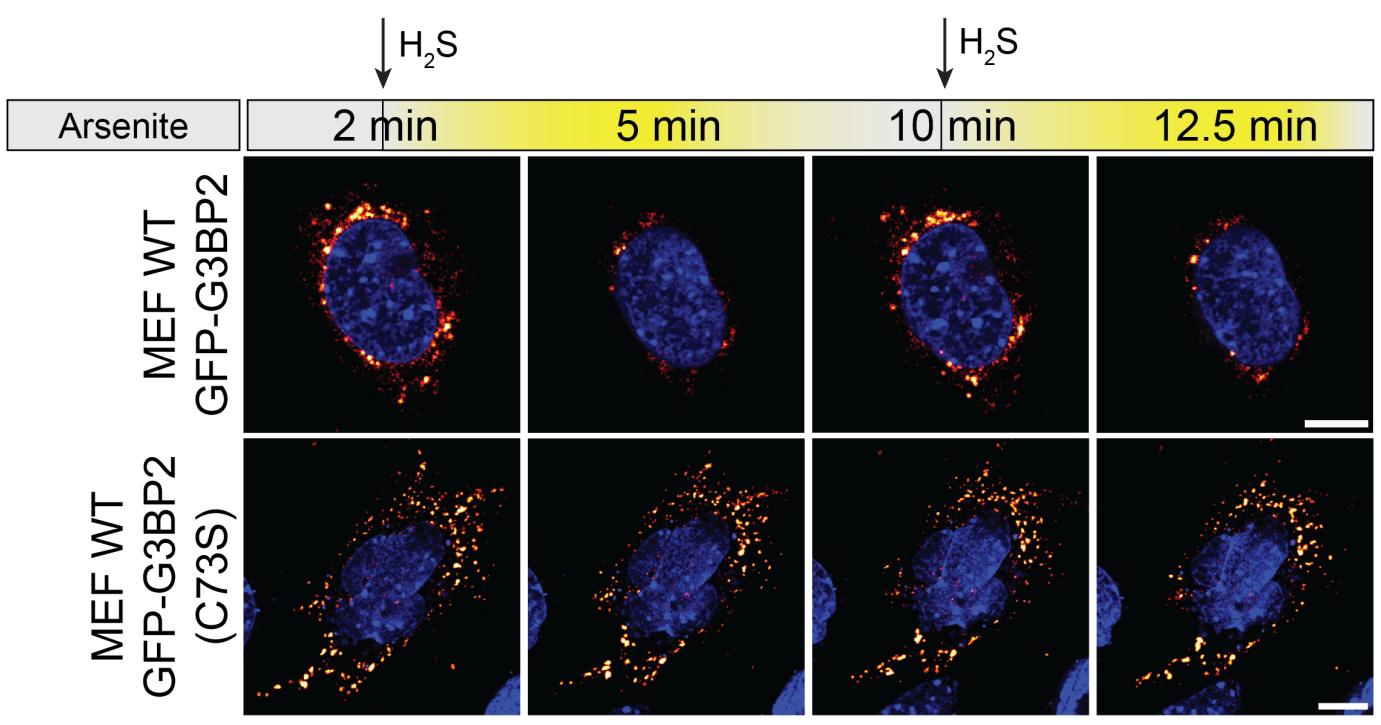

Figure 5.

*SG dissolution is absent in $Cse^{-/-}$ MEF cells (**Fig. 5e–g**), in Huntington's disease cells (**Extended Data Fig. 10a,b**), in which we have previously demonstrated near-complete loss of CSE and PSSH (Zivanovic et al., Cell Metab, 2019), and, most importantly, in human primary fibroblasts from both young and old donors (**Fig. 5h–k**). We further show that ergothioneine produces a similar effect, but only when CSE is present, as CSE is required to convert it into H_2S (Petrovic et al., Cell Metab, 2025). Conversely, pharmacological inhibition of CSE during the recovery phase produces the opposite phenotype, an inability to resolve SGs (**Extended Data Fig. 9e**).*

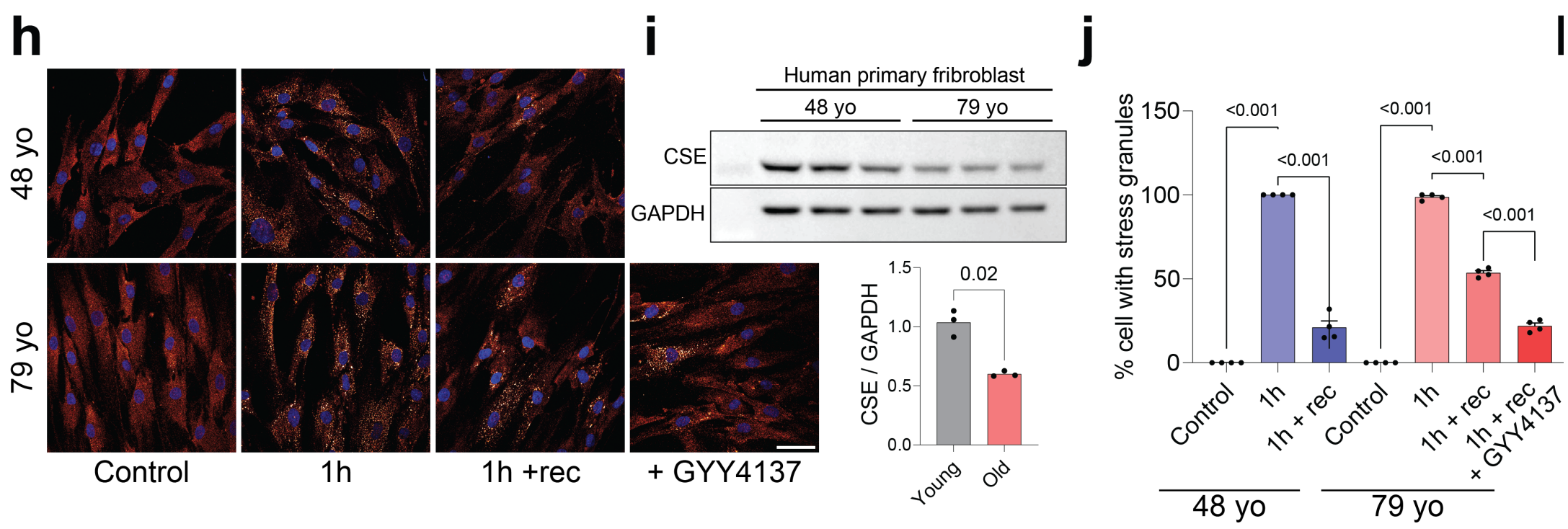

Figure 5.

3. The protocols for generating specific cysteine PTMs (PSO_3H, PSSH) on purified proteins lack thorough validation: 1) Quantitative conversion of thiols to PSO_3H using H_2O_2 is unlikely. Residual disulfides or sulfinic acid (PSO_2H) could confound results. 2) Pre-formed PSO_2H/PSO_3H are irreversible by reductants like DTT, potentially skewing comparisons with reduced/PSSH forms. 3) Clarifying the exact chemical composition and yield of the modified proteins used in LLPS assays is critical for interpreting the observed effects.

*We thank the reviewer for this comment. Indeed, in our schemes (**Extended data Fig. 6d and Extended data Fig. 8a**) we acknowledged that the used approaches could not yield 100% of the desired modification. As a redox biology expert, the reviewer will surely appreciate that it is generally impossible to achieve something like that. The protocol we used for cysteine hyperoxidation requires DTT to re-reduce any PSSP that could be formed and to enforce their full oxidation by H_2O_2. Thus, the use of DTT as a control. DTT-reduced protein is also a control for the PSSH, as reduced thiols do not react with H_2S. We clarify this in the manuscript: We used H_2O_2 treatment of fully reduced synapsin 1 as a method to generate PSOH (and/or PSSP) mimicking the intracellular thiol oxidation.[2,3] As shown in numerous previous studies, co-treatment with H_2O_2 and H_2S was used to transiently generate PSSH,[4–7] also mimicking intracellular mechanism for PSSH generation. PSO_3H was generated by adding H_2O_2 to protein containing small amounts of DTT. The role of DTT was to prevent disulfide formation and drive cysteine hyperoxidation. It is worth noting that this strategy also leads to the formation of PSO_2H, so we refer to it as PSO_xH (x = 2,3) (Extended Data Fig. 6d). Addition of H_2S to fully reduced protein was used as a negative control for PSSH, whereas treatment of iodoacetamide (IAM)-blocked protein thiols with H_2O_2 served as a control for methionine oxidation.*

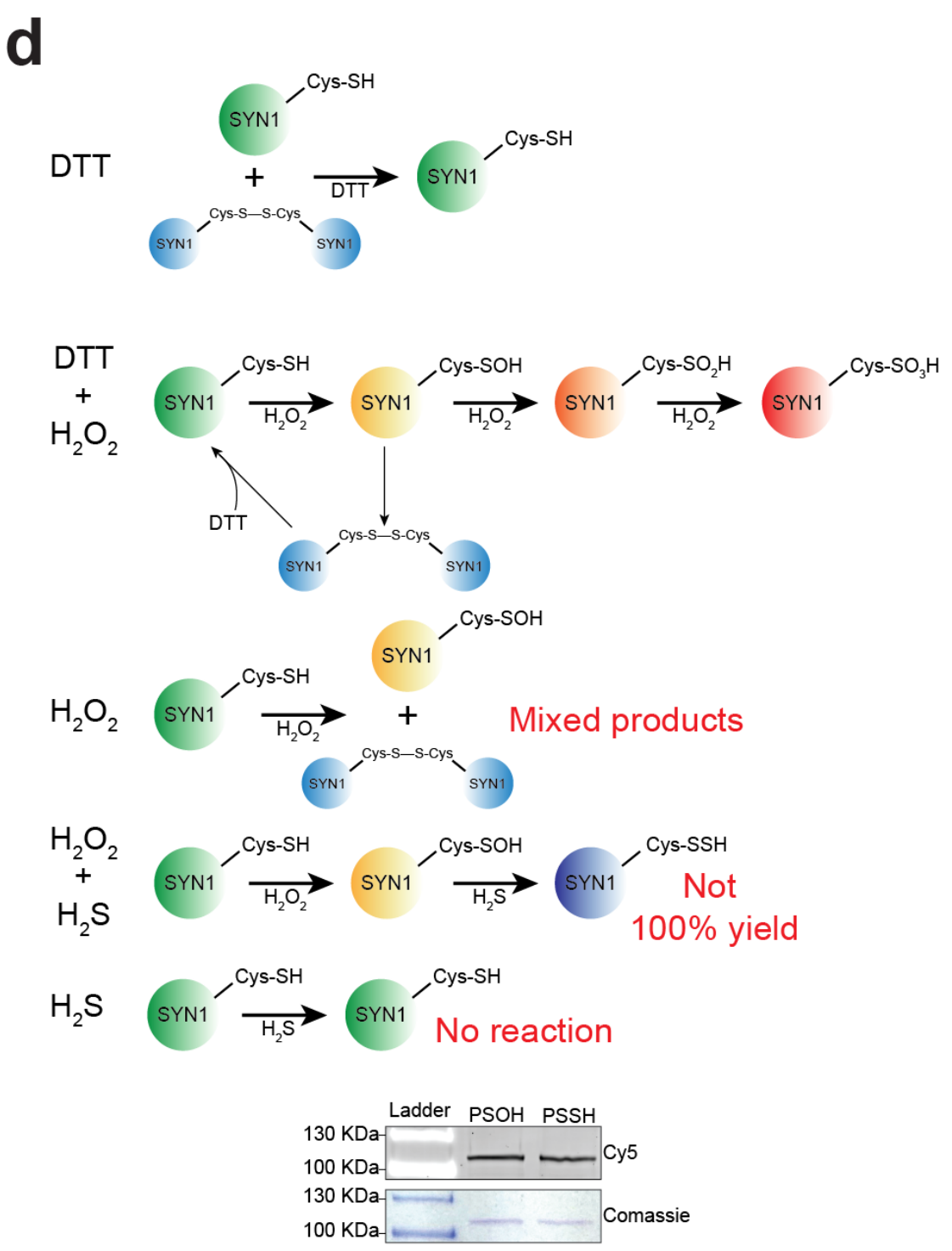

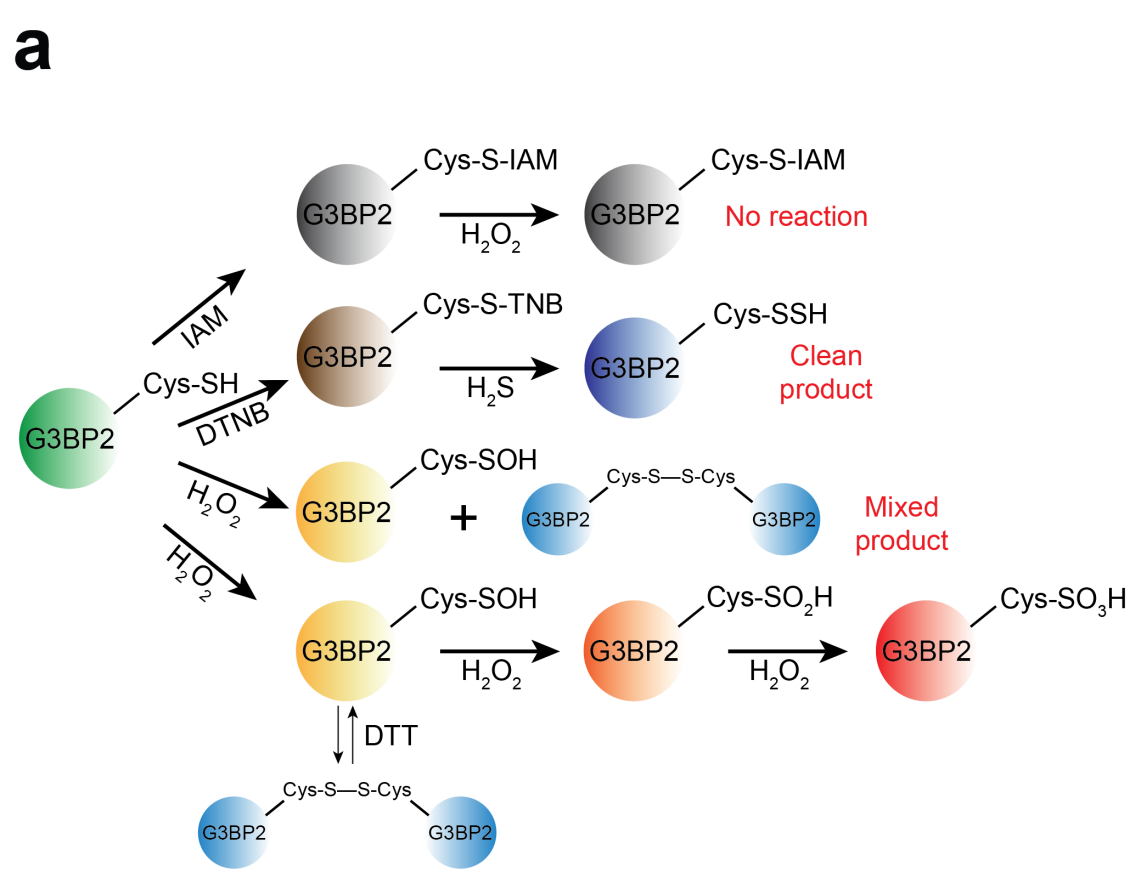

Extended data Fig 6. ***Extended data Fig 8.***

*For example, in our **Synapsin-1 experiments (Figure 3g,e,f, Extended data Fig 6f,g)**:*

1. *Treatment with **H_2O_2 alone** (which can produce PSOH and disulfides, and potentially methionine oxidation (Paulsen & Carroll, Chem Rev, 2013)—although methionine*

oxidation by H_2O_2 is very slow and kinetically unfavourable under the low molar ratios and short incubation times we used) did not change droplet size or FRAP recovery.

2. *In contrast, treatment with **H_2O_2 + DTT**, which drives cysteines towards cysteine hyperoxidation, produced clear effects on droplet size and FRAP recovery. This experiment is therefore controlled by experiment (1). We now refer to it as PSOxH, as the reviewer is absolutely right, it is impossible not to have PSO_2H there.*
3. *Similarly, addition of **H_2S** had no effect on DTT-reduced Synapsin-1. This is expected, as H_2S cannot react with reduced thiols. Only H_2S applied after cysteine oxidation (i.e., H_2O_2 + H_2S) resulted in persulfide (PSSH) formation (Filipovic et al, Chem Rev, 2018) and change in droplet size and FRAP recovery. As the reviewer is undoubtedly aware, PSSH will keep reacting with itself, forming disulfides and trisulfides, but considering that we show that disulfides have no effect (experiment (1) with H_2O_2 alone) the potential impurities in this experiment could not explain the observed effect of PSSH.*

*We now included additional control experiments where cysteine residues of synapsin 1 and G3BP2 were blocked with IAM and then treated with DTT, H_2O_2 or both, to show that they don't have effect on LLPS properties, excluding possibility that methionine oxidation plays a role (**Supplementary Figure 5** (for synapsin) and **Extended Data 8c,d** (for G3BP2)).*

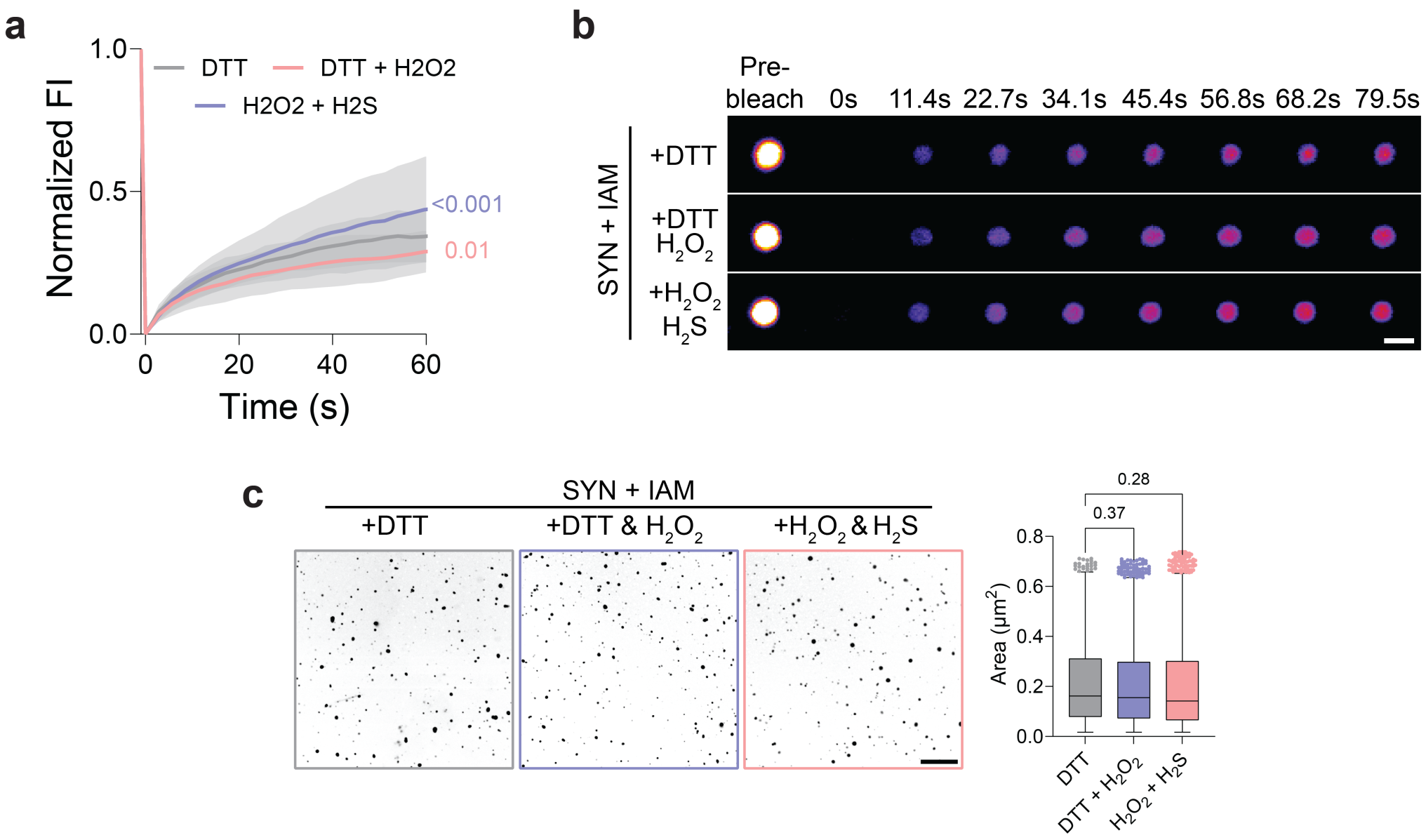

Supplementary Fig 5.

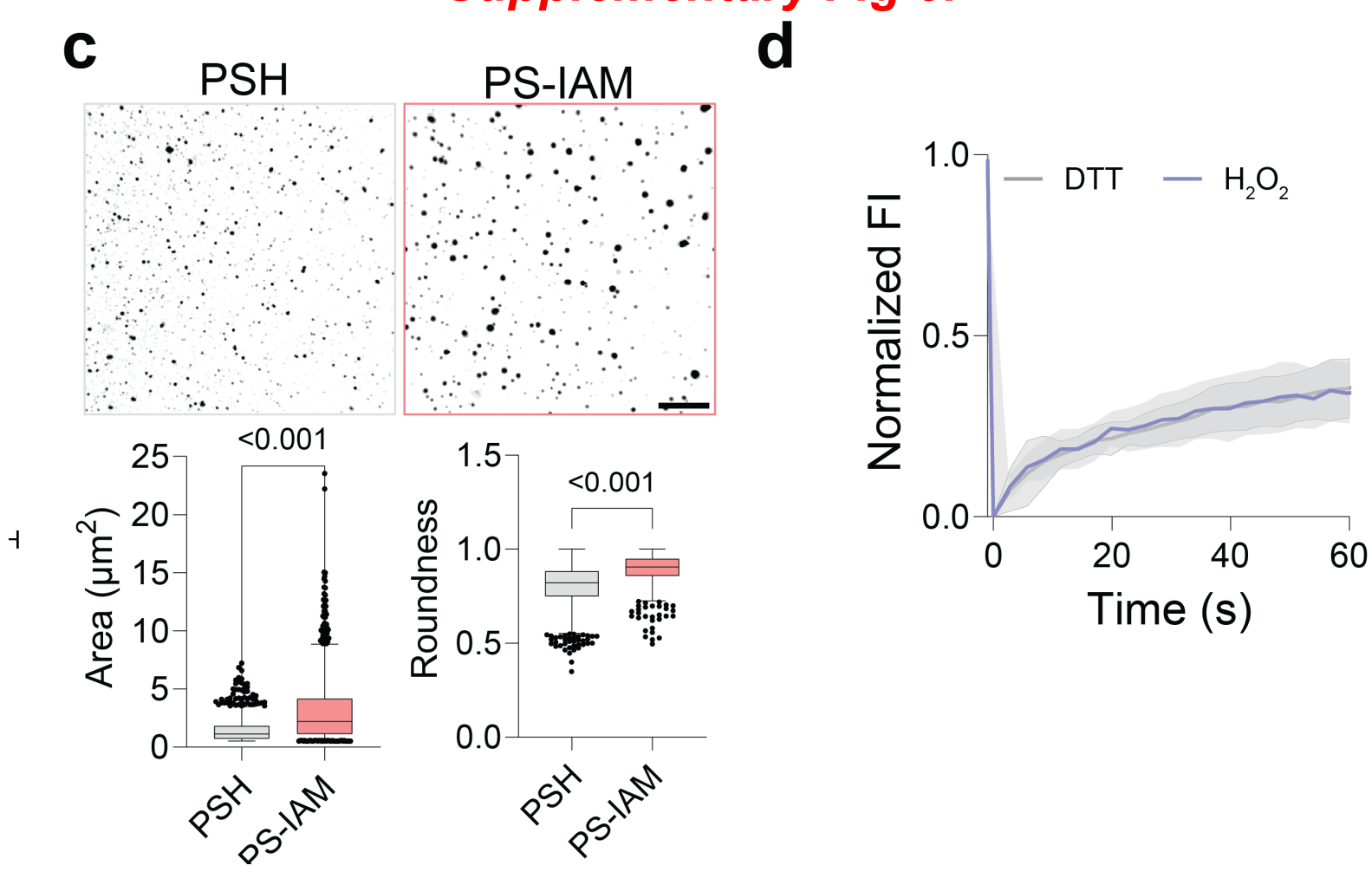

Extended Data Fig 8.

In summary,** we have provided chemical controls to the best of our experimental capabilities for each species generated. For PSSH, addition of H_2S to the DTT-reduced protein (in the case of synapsin) had no effect, in contrast to the conditions specifically used to generate PSSH. Likewise, treatment with H_2O_2 alone produced no changes, indicating that the PSOH/PSSP mixture does not alter synapsin behaviour. In contrast, conditions designed to induce hyperoxidation (PSO_xH), namely the combination of H_2O_2 and DTT, produced a significant and highly reproducible effect. These changes were consistent across independent protein preparations and even when experiments were performed months apart, suggesting that although the exact distribution of PSO_2H and PSO_3H within the PSO_xH mixture may vary, the functional outcome is remarkably stable. Blocking cysteine residues with IAM prior to applying the same treatments had no effect on LLPS behaviour, ruling out the possibility that the observed changes arise from methionine oxidation. Thiol quantification further confirmed that H_2O_2/DTT treatment results in complete loss of free thiols. Additionally, the protocol used to generate PSSH in G3BP2 (**Extended Data Fig 8a**) yields ~95% PSSH, as determined by TNB release following the addition of H_2S, consistent with our previous studies employing this approach. **These details have now been incorporated into the Materials and Methods section describing the relevant protocols.

We also included the following statement in the manuscript: Our experimental design deliberately aimed to mimic the continual formation and interconversion of these oxidative and persulfidated states, analogous to the dynamic conditions in cells where fluxes of H_2O_2 and/or H_2S continuously reshape the redox landscape (*Supplementary Text*).

4. The study establishes that PSSH inhibits LLPS but offers limited insight into the biophysical mechanism (e.g., conformational changes, altered hydrophobicity, charge modulation) compared to the detailed analysis of sulfonylation's effects. Understanding how persulfidation dissolves/prevents condensates is fundamental.

*The reviewer is absolutely correct to raise this important question, and it is something we have considered extensively. However, modelling LLPS is not a trivial undertaking and, to date, has only been achieved in a limited number of cases (Schäfer & Stelzl, Current Opinion in Structural Biology, 2025). The physicochemical principles governing LLPS remain incompletely understood and are not yet readily quantifiable or predictable. Even structural optimization of PSSH and PSOH modifications in well-folded proteins is a considerable challenge and rarely attempted. To our knowledge, no PSSH modelling has been reported to date, and only a few examples exist for PSOH. Nonetheless, we estimated the pKa values of the cysteines modified in synapsin-1 and G3BP2 using CysPka and found them to be unusually acidic and therefore fully deprotonated (and reactive) at physiological pH. This finding rules out the possibility that the observed LLPS differences arise from changes in cysteine protonation state, as PSOH, PSSH, and PSO_xH are all expected to be deprotonated and generally more acidic than the corresponding thiol. We further performed AlphaFold-based modelling of C/S and C/E mutants, intended to mimic PSOH and PSO_3H, respectively. These analyses revealed opposite effects on the local hydrophobic environment surrounding the modified cysteine: hydrophobicity increased in synapsin-1 but decreased in G3BP2 (**Supplementary Fig. 4**). This is consistent with the fact that LLPS in these two proteins is driven by distinct mechanisms. G3BP2 undergoes LLPS primarily through interactions between its positively charged IDRs and RNA, whereas synapsin-1 phase separates through multivalent interactions mediated by its intrinsically disordered regions, with additional contributions from weak hydrophobic contacts.*

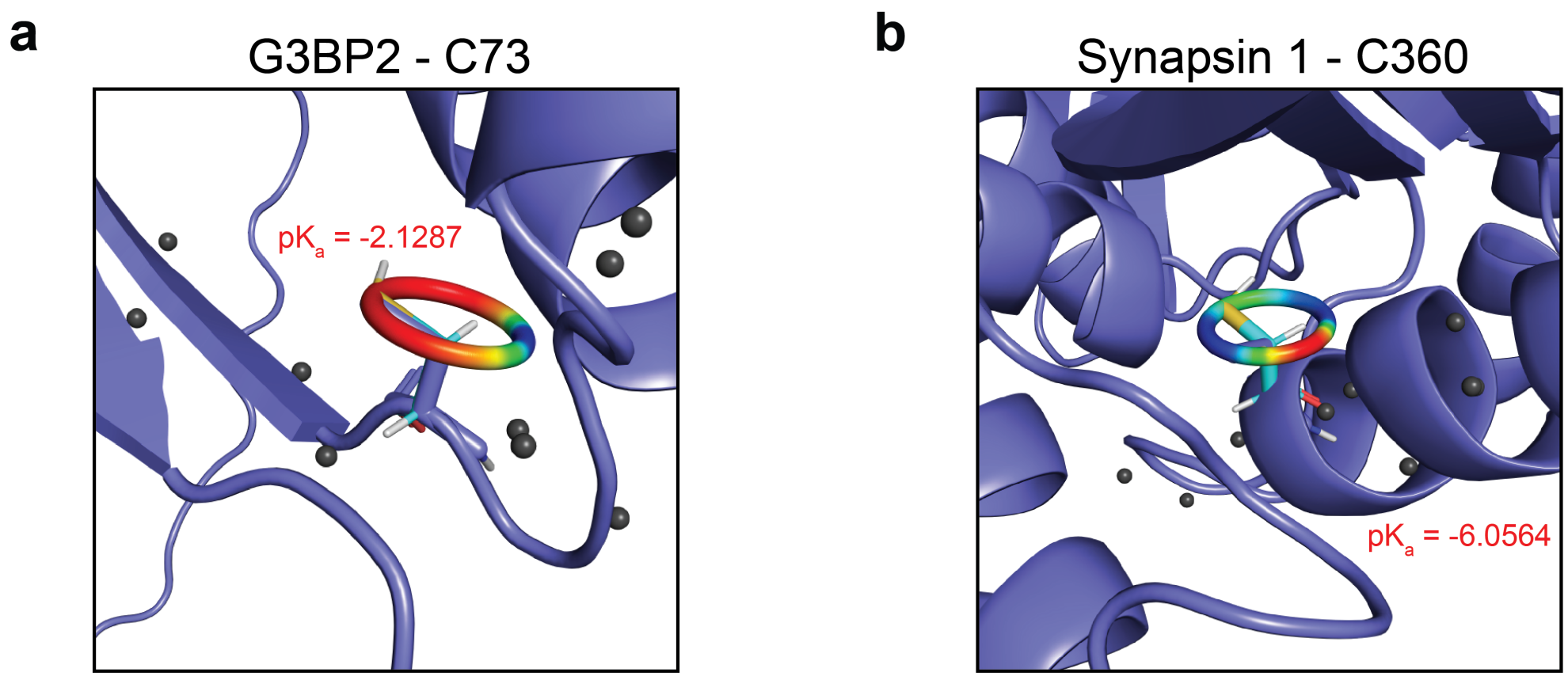

G3BP2	WT	C/S	C/E
Total sasa	342.229	496.19	459.206
Hydrophobic sasa	50.806	36.747	16.164
Hydrophobicity	0.148456	0.074058	0.035200

Synapsin-1	WT	C/S	C/E
Total sasa	192.914	200.261	227.544
Hydrophobic sasa	19.009	35.761	48.46
Hydrophobicity	0.098536	0.178572	0.212970

Supplementary Fig 4.

We also performed molecular dynamic simulations of G3BP2 WT and C/S mutant as monomers and dimers. Obtained data fit nicely to the observed biological effects, but these data are not included in the manuscript, and they will be included in our future work. Nonetheless, for the reviewer only, we summarize here our observations. Comparative MD simulations of WT and C73S G3BP2 monomers, show that the C73S mutation does not disrupt the overall fold but consistently shifts the protein toward a more flexible and partially expanded conformational ensemble. While RMSD, DSSP profiles, and hydrogen-bond counts indicate preserved global stability, the mutant exhibits higher RMSF values, particularly in the 150–220 region and the C-terminus, along with increased solvent exposure and a higher radius of gyration. Axis-resolved RoG and internal distance measurements further reveal that C73S adopts a more elongated structural state with greater dynamic fluctuations. Together, these changes suggest that the mutation subtly destabilizes local packing and enhances conformational mobility without altering the protein's core architecture.

Modelling of the interactions of dimeric form of the protein, required for the initiation of LLPS, we could observe that the combined simulations and binding free energy calculations suggest that the C73S substitution subtly but consistently remodels G3BP2's structural and interaction landscape in ways that could promote stress granule (SG) assembly. MM/GBSA analysis indicates a more favorable ΔTOTAL for the mutant, with compensatory shifts between electrostatics and solvation that are consistent with a more hydrated, polarity-adjusted microenvironment near residue 73. This is accompanied by local RMSF decreases at specific loop and interface regions, implying a reduction in conformational flexibility, and by a small but reproducible compaction in radius of gyration and tighter inter-chain distances. In the context of phase separation, such changes could lower the energetic barrier for multimerization by stabilizing intermolecular contacts and reducing the entropic cost of network formation. A more rigid interaction network would also be expected to slow internal rearrangements within condensates, consistent with the reduced FRAP recovery and increased droplet solidity observed experimentally. Thus, the C73S mutation may "prime" G3BP2 for condensation even under basal conditions, explaining the occasional spontaneous SG formation, while simultaneously producing condensates that are mechanically more solid-like and resistant to turnover once assembled.

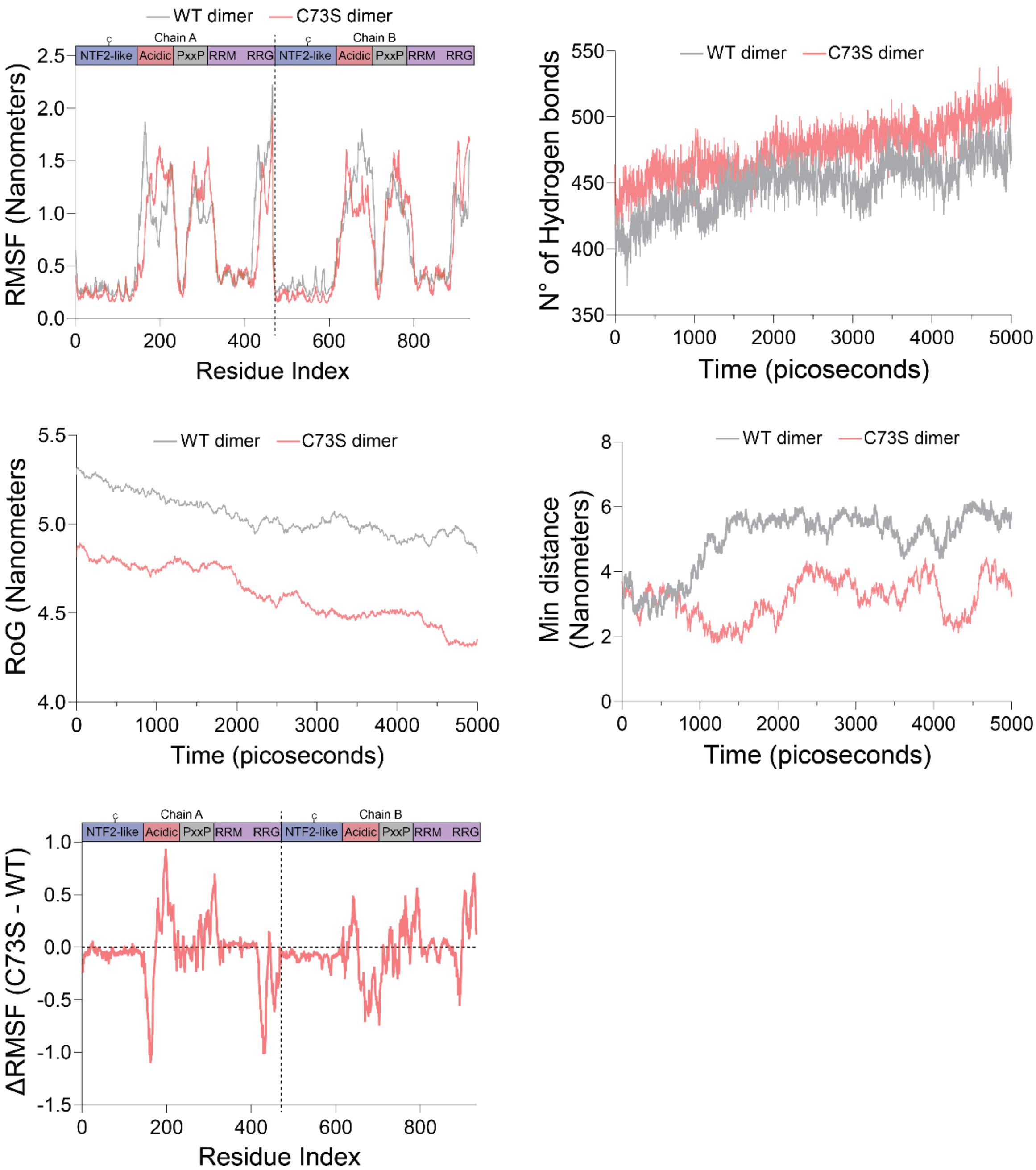

Figure 1 for the reviewers only. MD simulation results for the dimeric G3BP2 (gray) and C73S mutant (red). Dimeric structures are first obtained in AlphaFold and then used for the GROMACS simulation using AMBER99SB-ILDN force field.

Taking all this into consideration, as well as the fact that no mutation could substitute for PSSH, we included the following discussion in the manuscript: "In both G3BP2 and synapsin-1, CyspKa predicts exceptionally low apparent pKa values for their redox-active cysteines (≈ −2 for G3BP2 C73 and ≈ −6 for synapsin-1 C360) (Supplementary Fig. 5), indicating that these residues occupy highly thiolate-stabilized reactive hotspots. Notably, mutation of these cysteines to serine or glutamate produced opposite shifts in the hydrophobic character of their local environments: hydrophobicity decreased twofold around G3BP2 C73 but increased approximately twofold around synapsin-1 C360 (Supplementary Fig. 5). These divergent behaviours underscore that local packing effects are highly context-specific and that no single physicochemical parameter (e.g., hydrophobicity) uniformly explains the functional

outcomes. Likewise, because PSSH, PSOH, and PSO_xH modifications are all expected to be deprotonated at physiological pH, changes in net charge cannot account for the markedly different effects of each oxidation state.

Instead, we propose that persulfidation at such thiolate hotspots acts as a general anti-sticker modification. By extending the cysteine side chain with an additional sulfur atom and increasing local solvation, polarizability, and conformational flexibility,[8] persulfidation disrupts thiolate-dependent contacts and introduces dynamic S–S exchange. These features collectively reduce the effective multivalency of the interaction network. In G3BP2, this could manifest as weakened coupling between the NTF2-like domain, low-complexity regions, and RNA, whereas in synapsin-1 it could diminish the cohesive interactions required for crowding-enhanced condensation with synaptic vesicles. In both systems, the consequence is dissolution of the condensate.

By contrast, irreversible overoxidation to cysteine sulfonic acid introduces a rigid, strongly electronegative SO_xH group that acts as a local anchoring point, akin to phosphorylation,[9] thereby enhancing cohesion and promoting larger, more persistent droplets. Together, these observations indicate that the behaviour of cysteine-cantered redox PTMs is best understood through their differential effects on sticker strength rather than through global physicochemical variables such as net charge."

Minor Concerns & Clarifications:

1. Clarify the rationale for focusing specifically on the frontotemporal cortex.
We added a sentence which reads: We focused on the frontotemporal region because it is particularly vulnerable to age-associated neurodegeneration and exhibits early molecular changes relevant to cognitive decline.

2. Address potential artifactual oxidation during sample preparation for the 645 sulfonylated peptides detected without enrichment. Comment on their reproducibility.
As discussed in our manuscript, we used NBF-Cl as a blocking reagent in the lysis buffer. NBF is meant to block not only PSH and PSSH but PSOH, preventing the further oxidation of the latter. Manuscript text reads as: While established methods for selectively labeling and enriching PSO_3H are lacking, we analyzed proteome data obtained by lysing tissues with NBF-Cl, which blocks all thiols, including sulfenic acids (Fig. 1b), thereby preventing unwanted cysteine oxidation during sample preparation. We also specified in the Material and Methods section that we considered only peptide identified in 3 out of 4 biological samples as positive signal.

3. Figure 1j: The comparison of mouse PSSH data with human cell line data (Fu et al.) requires justification regarding cross-species relevance.
All mouse proteins were converted to their corresponding human protein identifiers before comparison with existing datasets. We believe that the ~45% overlap with targets reported across the experimental models used by Fu et al. represents a strong level of concordance, particularly given the expected differences between species.

4. Figure 2i: The relevance of the survival curve to the core LLPS/redox mechanisms could be strengthened. Otherwise, this can be removed.

*This figure is now **Fig. 6f** and this paragraph now reads: These findings suggest that aging, combined with reduced H_2S production, drives significant thiol remodeling, which may also have widespread implications for cellular survival in $Cse^{-/-}$ mice. These effects are unlikely to be restricted to the brain and may instead be widespread, as G3BP-dependent stress granule assembly and disassembly play a universal role in cellular stress protection. With aging, impaired resolution of biomolecular condensates can disrupt cellular homeostasis and*

contribute to a decline in healthspan, ultimately affecting overall lifespan. Indeed, Cse$^{-/-}$ mice exhibited higher mortality rate before reaching 18 months of age. Comparative lifespan analysis revealed that Cse$^{-/-}$ mice have a significantly shorter lifespan than their WT littermates (Fig. 6f).

5. Figures 4f,g: Including H_2S donor (e.g., GYY4137) treatment in these MEF experiments (mirroring Fig. 4c) would directly test its role in resolving protein condensates.
*This figure is removed, but new figures on G3BP2 now show potential of Na2S, GYY4137 and ergothioneine to dissolve SG in vivo (**Fig 5d, Supplementary Movie 7, Figure 5h, Extended Data Fig 10**).*

6. Figures reporting undetectable PSO_3H in controls (e.g., Fig. 2g, 3c, Extended Data Fig. j,k,m,n) should acknowledge limitations of DDA proteomics' stochastic nature.
It is done

7. Extended Data Fig. 6c: Clarify the chemical identity denoted by "SO-IAM" and its origin. Is this a product from the side-reaction between PSOH and IAM? Or shall this be a sulfoxide product from the initial PSH-IAM conjugate.

This is expected to be a product of PSOH an IAM as documented by Reisz et al, FEBS J, 2013. We easily detect 1000-2000 perfectly resolved PSO-IAM peptides during the proteomic analysis when lysis is performed with excess of IAM. We clarified this in Material and Methods section.

8. Extended Data Fig. 10a: The marginal FT-IR change for PSO_3H-GAPDH seems inconsistent with strong LLPS effects; please comment.

Although these experiments are removed, we provide the answer here: Spectral shifts may seem subtle, they occur in the highly hydrated amide-bond region, where even minor rearrangements of hydrogen-bond networks can have pronounced functional consequences. The amide I band—dominated by C=O stretching between 1600–1700 cm^{-1}—is exquisitely sensitive to secondary-structure changes, but its broad envelope reflects overlapping contributions that must be deconvoluted to resolve distinct motifs. In oxidized GAPDH after 8 h, the most striking alterations are increased absorbance in the β-sheet regions: 1616–1625 cm^{-1} (aggregation-prone inter- and intramolecular β-sheets) and 1626–1640 cm^{-1} (intramolecular β-sheets). These shifts, though modest in absolute magnitude, indicate a significant stabilization of β-rich conformers upon oxidation.

9. Methods: Provide details on MS1-based quantification parameters; Specify software and key settings for Persulfidome data analysis; Report if any BTD-modified (+418.1311 Da) sites were identified.

This is all now modified according to the reviewer's request.

Reviewer #2 (Remarks to the Author):

In this study, the authors used advanced chemoproteomic techniques to investigate cysteine PTMs in the mouse brain. They find age-related increases in thiol oxidation promote the formation of biomolecular condensates, leading to protein aggregation and protein persulfidation inhibits biomolecular condensation. They also find mice deficient in cystathionine g-lyase (CSE), exhibit reduced lifespans and spontaneously develop neurofibrillary tangles with age. This is an interesting study with both technical and conceptual innovations. However, the major issue is that in multiple places the data didn't really support the conclusion. Following are my comments for the authors to consider:

We thank the reviewer for the positive evaluation of our manuscript.

1. In Fig 1I, there seems to be no consistent decrease of PSSH in large amount of proteins in the aged mice. This makes the overall correlation of decreased PSSH with aging questionable.

The linear decrease is not expected as this is a dynamic modification that is subject to protein-controlled H_2S formation and protein-controlled depersulfidation. Also, H_2S production is regulated by 3 different proteins. We show that most of the detected proteins show decrease in at least one aging point (most of them show decrease mid-life, 10-month-old mice). This mid-life decline aligns with recent reports of non-linear dynamics of multi-omics profiles in human aging (Shen et al, Nat Aging, 2024).

These observations are also consistent with the cellular distribution of sulfide- producing enzymes: CSE is localized to neurons, cystathionine beta synthase (CBS) to glial cells, and 3-mercaptopyruvate sulfurtransferase (MPST) to both neurons and astrocytes (Zivanovic et al, Cell Metab, 2019). Thus, the observed changes in persulfidation reflect cumulative effects across cell types. However, it is the loss of CSE-regulated persulfidation that has profound implications for aging and neurodegeneration, as its depletion shifts the balance toward neurodegenerative events (Chakraborty et al, 2025, accompanying study for reviewer only).

To make data more accessible and user friendly, we have now created an online platform for interactive data exploration: ***https://aging-redox-proteomics-viewer.streamlit.app/***
The site is currently password-protected (username: reviewer.app; password: Ycql59H3s621).
This platform allows users to search proteins, examine their individual aging PSOH and PSSH profiles (including changes observed in CSE knockout animals), and select pathways of interest across datasets. We also integrated direct links to the UniProt database, enabling users to access detailed protein information, including structural data.

2. The neurofibrillary tangle shown in Fig 2j doesn't look like neurofibrillary tangle at all. This observation needs to be confirmed using many of commercially available antibodies against pathological tau.

The reviewer is right that staining with tau antibodies is the best way to show the neurofibrillary tangles. Gallaya's staining (with all its limitations) has been in use in confirming postmortem AD from human samples long before the introduction of tau (Moloney et al, Visualization of neurofibrillary tangle maturity in Alzheimer's disease: A clinicopathologic perspective for biomarker research, Azlheimer & Dementia, 2021). The reason we did not include tau is that in collaboration with Prof Paul we have another manuscript which is dealing with phenotyping of CSE ko mice (Chakraborty et al, 2025, accompanying study for reviewer only). We show that those mice not only have accumulation of tau fibrils but a full cognitive phenotype reminiscent of AD and/or dementia. In attempt to provide something to further strengthen our claims, and without compromising our collaborator's work, we did include in the revised manuscript the filter trap assay for detecting aggregated proteins and we show now increased

*aggregation of TDP-43 in Cse$^{-/-}$ MEF cells (**Supplementary Figure 8a**), but more importantly increased aggregation of amyloid beta and TIA-1 in Cse$^{-/-}$ brain lysates (**Figure 6b,c**).*

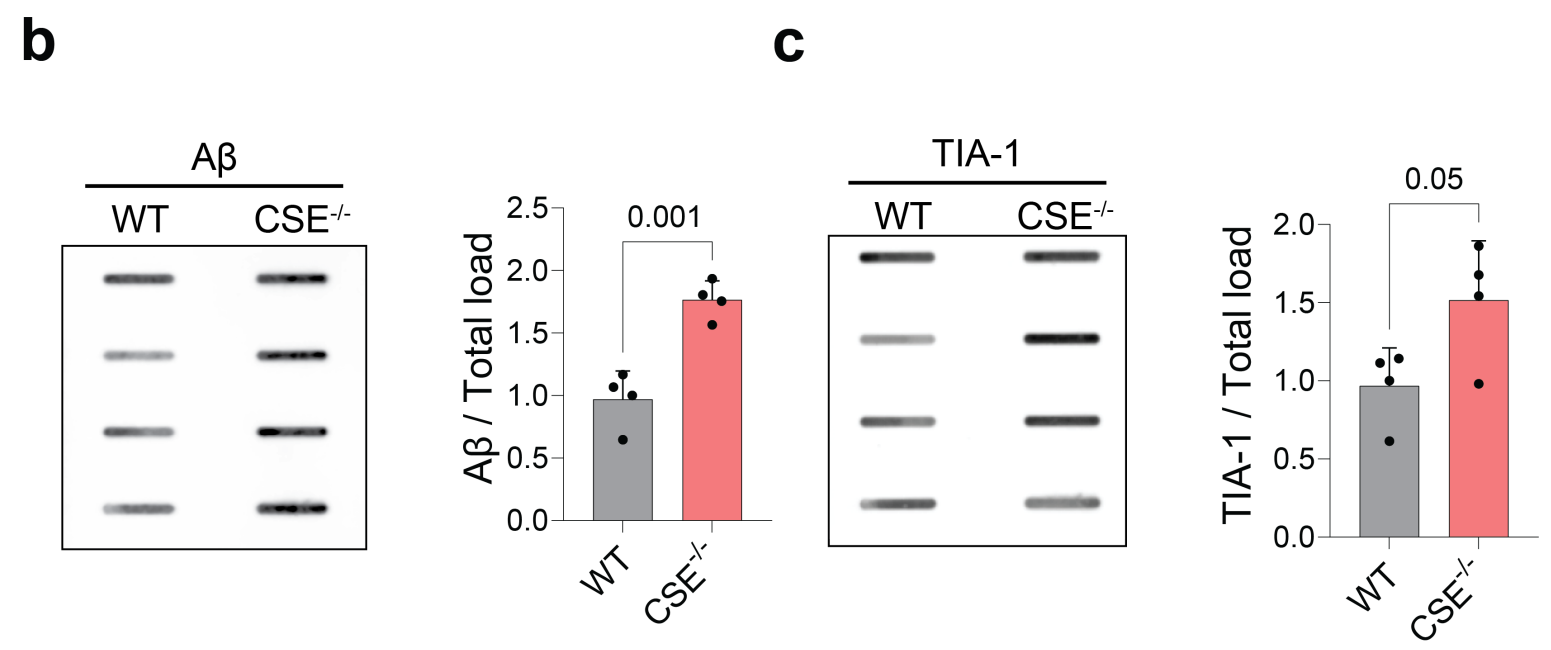

Figure 6.

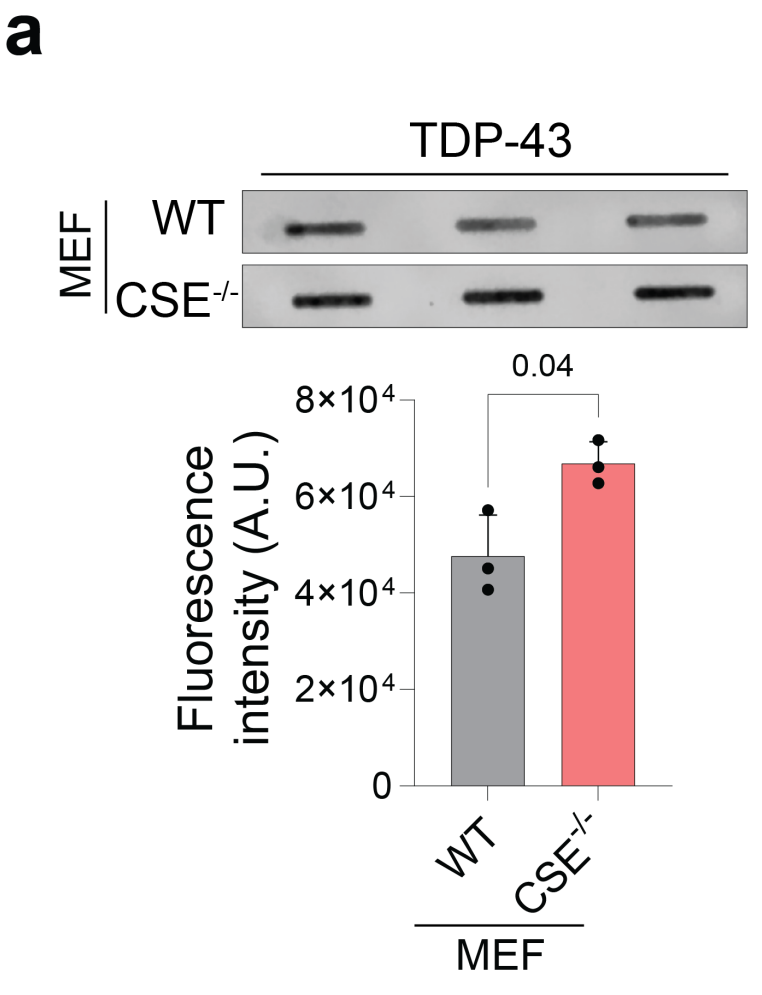

Supplementary Fig 8.

3. The data shown in Figure 4e didn't demonstrate "more pronounced phase separation" for glycolytic enzymes. It only indicates that these enzymes are in different protein/molecule complex. Also, the changes shown in Figure 4b&e are very small, without statistical significance.

As communicated before, we removed this figure. It seems that our attempt to use newly developed method for proteome-wide detection of proteins undergoing LLPS (Li et al. Nat Chem, 2024, PMID: 38499848) have created more concerns than provided answer. We followed, to the letter this method, which uses volumetric compression and sucrose gradient fractionation to profile endogenous biomolecular condensates. This method distinguishes between LLPS and higher-order macromolecular assemblies. Their paper validated this approach using FRAP, disorder prediction, and urea/RNase treatments.

We however apologize for the omission of significance markers in several panels. All reported differences were indeed statistically significant ($p < 0.05$). However, as per criteria established by Li et al, 2024, LLPS membership requires a ≥50 % enrichment in the condensed fraction relative to fraction 1; proteins that fail to reach this threshold are not classified as condensate-associated, even if the change is statistically significant. Thus, in cases where only one condition (e.g., CSE KO) exceeds the 50 % cutoff, we deliberately omitted stars on the non-enriched groups to avoid implying condensate partitioning where none occurs.

4. The data shown in Figure 4f&g need to be quantified.

*As we removed GAPDH data and replaced them with new data on G3BP2, we provide data quantification for all microscopy images (These are now new **Fig. 4f-i, Fig. 5 e-k, Extended data Figs 9e, 10, and Supplementary Fig 7**). As an example, we show here new **Fig 4f-i.***

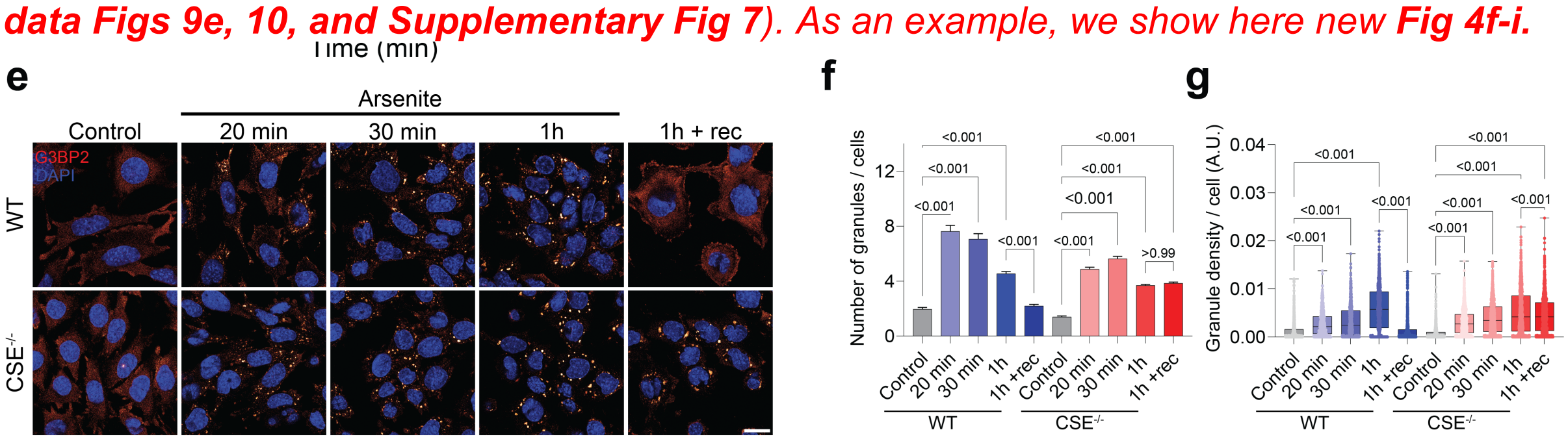

Figure 5.

5. The authors claimed "an approximately 3-fold increase in GAPDH within the aggregated fraction of Cse-/- lysates" based on the data shown in Figure 5I. However, based on the figure , there is a dramatic decrease of soluble GAPDH rather than increase of insoluble GAPDH in Cse-/- mice.

These data are now removed from the manuscript.

Reviewer #3 (Remarks to the Author):

The ambitious work by Vigane et al seeks to test a new hypothesis that thiol modifications change liquid-liquid phase separation in a pathological manner in the brain. The authors perform an extensive proteome wide identification of PSOH and PSSH in the frontotemporal mouse brain vs aging using some new tools to specifically identify the modified forms, finding changes with age. Using a CSE-/- mouse that should disrupt persulfidation, they show changes in the proteomic modifications and in phenotype. Modified proteins are predicted to have biophysical impacts. Enhanced formation of PSO3H is also probed for the CSE ko. Then the authors shift to examining two specific targets. For synapsin1, they show that its modification levels change with aging and in the CSE ko, the modification found should have some effect on the structure and it has an impact on the phase separation in recombinant protein assays as well as some data on neurotransmitter release. This led the authors to examine potential phase separating proteins in the proteome including G3BP, followed by GAPDH. For GAPDH, they examine the effect of the modifications on phase separation and biochemical activity as well as aggregation biochemically and in the mouse brain. Overall the work is intriguing and expansive but a number of major issues prevent it from being robust and convincing is several areas. The proteomic evaluations seems a large strength, but the candidate data overall is not clear and the biochemical experiments have issues in design and lack proper controls as well as evidence of mechanism. I see the proteomic experiments are exciting but do not think the biochemical experiment fit well.

We thank the reviewer for the encouraging words.

Major

Do the authors have any explanation for why the proteins that were found only by other groups in Fig 1j and Supplemental Fig. 7a/b were not found in this study?

The comparisons shown in Fig. 1 are not exclusively with brain tissue or neurons; some are performed using human cell lines, as they serve distinct purposes. Our study employs a chemoselective methodology for detecting protein persulfidation that we developed to selectively label PSSH (Zivanovic et al., Cell Metab., 2019; Petrovic et al., Cell Metab., 2025), as well as the established chemoselective method developed by the Carroll and Yang groups for detecting PSOH. While the latter is widely adopted, the former is still relatively new, and we therefore compared our PSSH data with the limited datasets available from other groups. Despite these methodological and biological differences, we observe strong overlap with both Fu et al. and Gupta et al. for PSOH (>60%), even though their analyses were performed in human cell lines. To our knowledge, no studies have quantified PSOH in brain tissue to date. When comparing our PSSH dataset with that of Bithi et al. (2021), which was generated in mouse brain, we observe an even higher overlap (~80%), with the notable distinction that our method identifies approximately twice as many persulfidated targets. Taken together, we consider these overlaps to be robust by proteomic standards, particularly given the differences in species, sample types, and analytical methodologies.

The mechanistic insight provided by the phase separation experiments needs to be improved extensively.

We hope that this revised version of the manuscript will satisfy the reviewer's concerns.

The effect of the modifications on synapsin1 droplet area is very small.

This must be some misunderstanding. Arithmetic mean of more than 360 analyzed droplets suggests that the difference is ~2 fold. This is also obvious from the wetting experiments. We

can include quantification of other parameters such as roundness etc. The differences in FRAP kinetics and recovery are also obvious.

I understand that the authors have performed a replicate experiment with independently “prepared” samples. Those data should be presented.

These data were already presented. We always plot all our data from all our replicates. As mentioned in the legend of Fig 3, we analysed between 360-840 droplets (depending on the experimental conditions). The excel datasheet showing all raw, source data for the entire manuscript is now included in resubmission.

A wetting change also means that the droplet area is not the correct measure.

We thank the reviewer for this comment. We interpret the concern to be that changes in wetting behavior can alter the droplet footprint area independently of changes in condensate volume. In our system, however, wetting differences in synapsin appear only at later time points, whereas droplet area was quantified at the very onset of LLPS, before detectable wetting changes occurred. Thus, the initial droplet-size measurements are not confounded by wetting effects and provide a valid readout of condensate formation. The increased wetting observed later in PSO_3H-modified synapsin is consistent with, and further supports, the enhanced condensate engagement of this species but does not influence the initial droplet measurements reported.

As oxidation can cause aggregation and loss of soluble protein, even the figure 3g could be reporting on something other than changes due to cysteine adducts.

Some of us have already shown previously that synapsin does not aggregate even when left for extended periods (up to two weeks at room temperature) under air oxidation. Its LLPS properties and FRAP recovery remain indistinguishable from control conditions, as previously demonstrated (Hoffmann et al., J. Mol. Biol., 2021, PMID: 33774037). Even if one assumes that some aggregation could occur, such a process would be expected to remove protein from solution rather than increase its apparent propensity to undergo LLPS, unless the reviewer suggests that the observed droplets are aggregates. However, our FRAP experiments clearly show ***faster and more complete fluorescence recovery*** *in hyperoxidized synapsin 1 compared with fully reduced synapsin, which is incompatible with an aggregation-based interpretation.*
Furthermore, synapsin treated only with H_2O_2 behaves identically to the fully reduced protein. *As an additional control, we blocked cysteines with iodoacetamide and then exposed the protein to H_2O_2, H_2O_2 + DTT, or H_2O_2+H_2S. Under these conditions, we observed no changes in droplet size (Supplementary Fig. 5). Together, these data strongly support that the effects we report arise from regulated redox modifications rather than nonspecific aggregation.*

Supplementary Figure 5.

The attempt to use hydrogen peroxide alone as a control for methionine oxidation is confusing as cysteine is oxidized by hydrogen peroxide also, so I do not follow the logic. The GFP could also be oxidized. The authors should find out where their biochemical protein is being oxidized.

We apologize if our previous explanation was unclear. While we attempted to illustrate the redox biochemistry through schematic depictions within the limited space of the format (Extended Data Fig. 6d and the corresponding figure legend), we now recognize that this may not have been sufficient. From a redox biochemistry perspective, we believe our experiments were well-controlled and performed to the highest standards of the field. However, it is crucial to view them as a whole rather than in isolation.

We have five experimental conditions:

1. ***Synapsin with DTT:** This ensures the cysteine residues remain fully reduced throughout the measurement.*
2. ***Synapsin with DTT and H_2S:** H_2S does not react with any amino acid under these conditions (Filipovic et al., Chem Rev, 2019). H2S can only react with oxidized cysteines (specifically, sulfenic acid, PSOH, and potentially disulfide bonds, PSSP).*
3. ***Synapsin treated with H_2O_2:** H_2O_2 initially reacts with cysteines to form sulfenic acid (PSOH), which can then lead to the formation of disulfide bonds (PSSP) (Paulsen & Carroll, Chem Rev, 2013). While H_2O_2 can also oxidize methionines, the reaction rate with cysteines (20-10^7 $M^{-1}s^{-1}$ to form sulfenic acid; 1-100 $M^{-1}s^{-1}$ to form sulfonate, PSO_xH; Paulsen and Carroll, Chem Rev, 2013) is significantly faster than with methionine (6-9 x 10^{-3} $M^{-1}s^{-1}$; Davis, Biochem J, 2016, Yao et al, Biochm, 1996, Sjoberg et al, J Photochem & Photobiol B; Biology, 2018). Given our controlled conditions (a 5-fold molar excess, which corresponds to only 1.67 H_2O_2 molecules per cysteine, and a short reaction time), we do not expect methionine to be the predominant target. Importantly, this treatment had no effect on synapsin's liquid-liquid phase separation (LLPS) or its FRAP recovery. Therefore, this experiment also serves as a control for methionine oxidation. **But we now performed experiments with IAM-blocked cysteines to ensure that methionine plays no role (Supplementary Fig 5).***

In these first three experiments, we observed no differences in droplet size or FRAP kinetics and recovery. This indicates that even if other modifications were occurring, as the reviewer suggested, they did not contribute to the observed effect. H_2O_2 treatment alone did not alter the size or LLPS properties of synapsin.

4. ***Synapsin with H_2O_2 and DTT:** The presence of DTT constantly reduces the newly formed PSSP, forcing the cysteines to react further with H_2O_2 to generate hyperoxidized forms (PSO_xH). Under these conditions, we observed a significant difference in droplet size and FRAP kinetics and recovery. The fact that these were enhanced, not reduced, argues against aggregation. Furthermore, we could generate a phase diagram showing clear shift towards lower synapsin concentrations and lower PEG concentrations.*
5. ***Synapsin with H_2O_2 and H_2S:** The addition of H_2S in combination with H_2O_2 traps the initial sulfenic acid (PSOH) as persulfide (PSSH). This also produced a noticeable effect, but one that was opposite to the H_2O_2 and DTT condition, highlighting that a completely different cysteine modification was formed. As previously stated, the chemistry of H_2S is well-characterized, and it does not react with other amino acids.*

Therefore, the suggestion that our observations are due to aggregation does not withstand chemical scrutiny. If aggregation were the cause, it would be expected to occur in the synapsin treated with H_2O_2 alone, and even more so in the synapsin treated with H_2O_2 and DTT. However, the former showed no change in FRAP recovery and kinetics, a gold standard for assessing the liquid state of droplets, while the latter increased both kinetics and recovery yield. This cannot be explained by aggregation.

*We have already provided evidence for the formation of these specific modifications using selective labelling methods (**Extended data Fig.6d**) and have identified them in the aging brains of wild-type and CSE knockout animals via mass spectrometry (**Fig. 3b, d**). We do detect the same C360 hyperoxidation in our isolated protein preparation; however, the absence of additional cysteine-oxidized peptides may simply reflect the inherent stochastic sampling limitations of DDA proteomics. In contrast, methionine oxidation is a ubiquitous modification observed in virtually all proteomic datasets. Its presence does not indicate that methionine oxidation occurred during the biochemical experiment itself, but rather that it arises during sample handling and processing for LC–MS analysis. For this reason, we relied heavily on "chemical controls," including experiments performed with IAM-blocked protein, to ensure that the observed effects originate specifically from cysteine-centered redox modifications.*

We have now modified the manuscript to provide clarity: We used H_2O_2 treatment of fully reduced synapsin 1 as a method to generate PSOH (and/or PSSP) mimicking the intracellular thiol oxidation.[45,46] As shown in numerous previous studies, co-treatment with H_2O_2 and H_2S was used to transiently generate PSSH,[47–50] also mimicking intracellular mechanism for PSSH generation. PSO_3H was generated by adding H_2O_2 to protein containing small amounts of DTT. The role of DTT was to prevent disulfide formation and drive cysteine hyperoxidation. It is worth noting that this strategy also leads to the formation of PSO_2H, so we refer to it as PSO_xH (x = 2,3) (Extended Data Fig. 6d). Addition of H_2S to fully reduced protein was used as a negative control for PSSH, whereas treatment of iodoacetamide (IAM)-blocked protein thiols with H_2O_2 served as a control for methionine oxidation. Our experimental design deliberately aimed to mimic the continual formation and interconversion of these oxidative and persulfidated states, analogous to the dynamic conditions in cells where fluxes of H_2O_2 and/or H_2S continuously reshape the redox landscape (Supplementary Text).

We also modified the Supplementary text to highlight the background on these modifications. Thiol oxidation to sulfenic acid (PSOH) represents only the initial step in a series of oxidative reactions that can occur within cells.[1–3] PSOH can react with other thiols to form disulfides (PSSP), either with protein thiols or with low–molecular weight thiols such as cysteine or glutathione. Alternatively, PSOH can react with H_2S to generate persulfides (PSSH)[4,5] or, in some cases, with cyanide to form thiocyanates (PSCN).[6] Under conditions of sustained oxidative stress, PSOH can undergo further oxidation to sulfinic (PSO_2H) and sulfonic (PSO_3H) acids.[3,7]

Persulfides are likewise highly reactive species.[4,8–12] The presence of both a nucleophilic and an electrophilic sulfur atom renders them intrinsically unstable, as they can react with themselves to form trisulfides (PSSSP). PSSH can also react with other thiols to form disulfides or with ROS, acting as stronger nucleophiles than their corresponding thiols and reacting with ROS at least an order of magnitude faster, to form S-sulfonates ($PSSO_3H$).[5]

The multiple and competing reaction pathways available to PSOH and PSSH necessitate the use of chemoselective methods to capture these modifications. At the same time, this chemical complexity means that any labelling and quantification strategy can only report their steady-state levels within cells, rather than their full dynamic range.

Finally, as previously reported, EGFP does not affect synapsin's ability to phase separate (Milovanovic et al, Science, 2018). EGFP-synapsin does not aggregate even when left to oxidize in the air at room temperature for two weeks; its LLPS and FRAP recovery remain the same as the contro (Hoffmann et al., J Mol Biol, 2021). EGFP is redox insensitive, due to the fact that cysteines are buried within the β-barrel structure and are not readily accessible. The mutational studies of EGFP to roGPF were required to achieve redox sensitivity to H_2O_2 (Hanson et al, JBC, 2004, PMID: 14722062), a feature used in development of genetically encoded H_2O_2 and redox sensors.

The authors really need to make variants with all surface and specific cysteines changed to alanine (or something else to prevent oxidation) and show what effect that has on the mass spec before and after treatment (to show what is really happening) and then on the phase separation (nothing should happen if the cysteines are no longer present) as it is possible the conditions themselves (slightly changes in pH etc) alter the phase separation. This would also help with mechanism.

*We appreciate this suggestion but disagree that this is the only way to prove what reviewer is keen to see. As mentioned earlier, these are not stable modifications (with exception of PSO_3H). PSOH and PSSH are transient modifications and we have applied the methods to generate them which are widely used in the redox biology field, some introduced and pioneered by us decades ago. It is impossible to "isolate" 100% pure PSOH or PSSH protein modification. If we haven't misunderstood, the reviewer is suggesting a top-down MS approach. Here are the reasons why top down MS approach would't work here: A mass increase of +16 Da, for example, cannot on its own distinguish between a sulfenic acid on cysteine (Cys-SOH) and an oxidation on methionine (Met-O). Similarly, a +32 Da shift could be interpreted as cysteine persulfidation (Cys-SSH) or the addition of two oxygen atoms. To unequivocally show that these modifications occur on cysteine residues, one must employ a targeted chemoproteomic approach. This involves selectively trapping the modified cysteines with a chemical probe (thus exposing the protein to chemical transformation), as we have done in Extended data Figure 6d**. Our new Supplementary Fig. 5 where experiments were performed with IAM-blocked protein achieve similar effect as a mutational study, that reviewer suggested, but also excludes the possibility that pH changes, buffer composition or methionine oxidation are involved in the process, as the IAM-proteins were exposed to the same treatment as the reduced protein.***

In the current analysis, we have applied an Occam's razor approach, relying on established kinetic and chemical principles to distinguish the most probable outcome from the possible.

- ***Chemical Kinetics:** It is well-established that the reaction rate for the oxidation of cysteine is approximately **two orders of magnitude faster** than that for methionine. The probability of oxidizing any other amino acid residue is statistically even lower, making cysteine the most likely target for the observed +16 Da modification. IAM-blocked samples do not show observed differences, excluding the possibility of methionine involvement and providing control for the pH and chemical composition changes.*
- ***Negative Controls:** Our conclusions are strongly supported by the absence of any effect in our key chemical controls. The application of H_2O_2 alone or H_2S alone failed to produce these modifications, which completely excludes the possibility that they arise from nonspecific or alternative chemical reactions. This demonstrates the modifications are a direct and specific result of our experimental conditions.*

Further, the authors should mimic the oxidation perhaps by seeing if methionine is a mimic for cysteine SSH (or a reaction with cysteine with MTS to make SSCH3) and with an acidic residue to mimic SOH (is it expected to be deprotonated at this pH? the one reference I found says pKa is 6.1 so perhaps it is deprotonated).

We appreciate the reviewer's suggestion to use mutations to mimic the modified cysteine. However, in the field of redox biochemistry, creating a mutation that faithfully reproduces the chemical behaviour of these specific modifications is considered unfeasible. The primary reason is that the charge state of the modified cysteine is critical. Modifications like Cys-SOH and Cys-SSH are expected to be deprotonated (PSO^- and PSS^-) based on their general pKa, although their actual state within the protein is highly influenced by interactions with nearby amino acid residues and impossible to predict without modelling. So, all 3 modifications would

result in same net charge change but quite profound conformational differences. The proposed mutations do not capture this essential chemical feature. A cysteine-to-methionine mutation introduces fundamentally different steric and electronic properties, while an MMTS block creates a stable R-S-S-CH_3 bond that cannot mimic the dynamic protonation state of a Cys-SSH group. Because these approaches lack the necessary chemical logic to serve as meaningful mimics, we feel that pursuing them would not yield interpretable data.

However, we do address part of this in Supplementary Fig 5. We estimate the pKa of cysteine in synapsin and G3BP2 and hydrophobicity change around affected cysteine in WT, C-to-S and C-to-E mutants modelled by AlphaFold. More details are provided in the answer bellow.

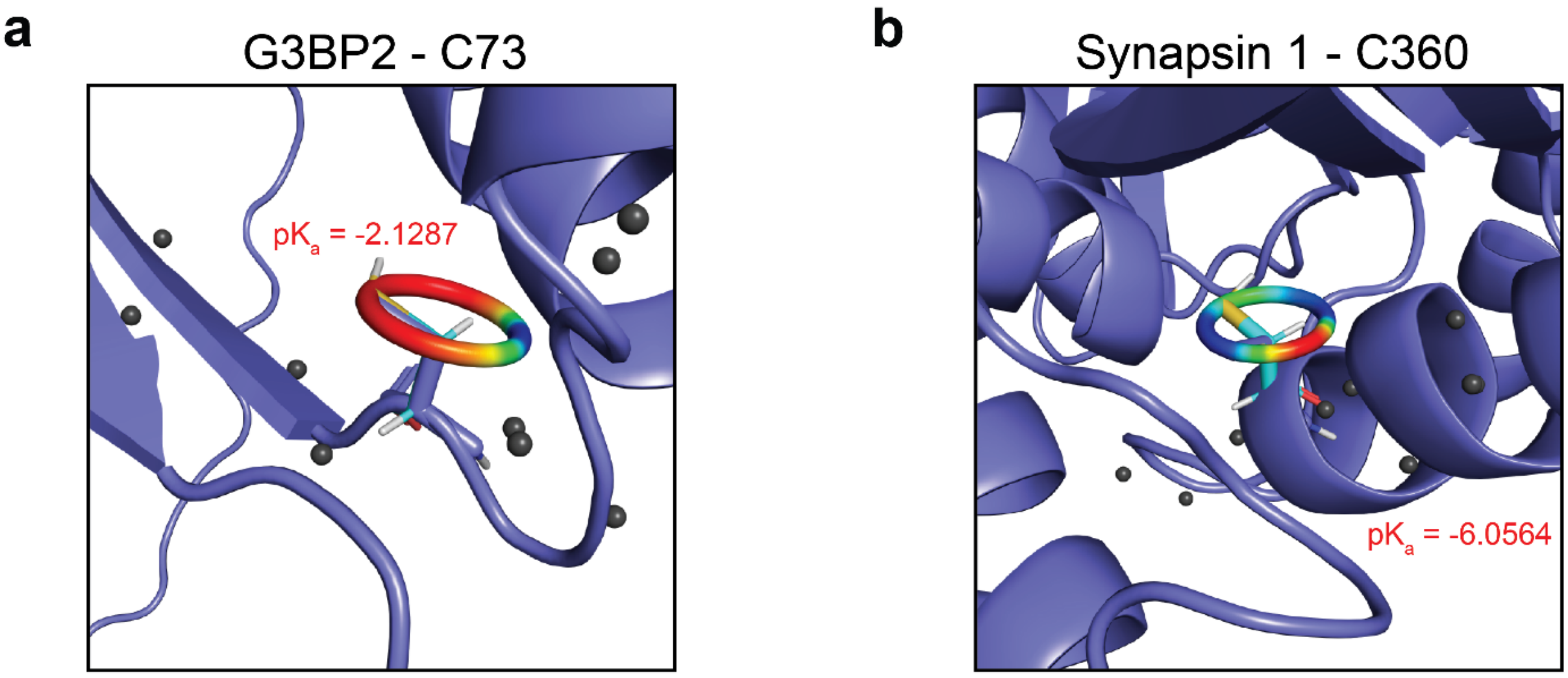

G3BP2	WT	C/S	C/E
Total sasa	342.229	496.19	459.206
Hydrophobic sasa	50.806	36.747	16.164
Hydrophobicity	0.148456	0.074058	0.035200

Synapsin-1	WT	C/S	C/E
Total sasa	192.914	200.261	227.544
Hydrophobic sasa	19.009	35.761	48.46
Hydrophobicity	0.098536	0.178572	0.212970

Supplementary Figure 5.

This would then need to be expanded to try to understand why the change in the residue causes the change in phase separation (if a similar change was made at another site, would the same change in phase separation and wetting occur?)

We thank the reviewer for this insightful comment. Elucidating the precise mechanism by which a specific residue modification alters phase separation is indeed a crucial long-term goal. Our current manuscript is intended as a foundational, proof-of-concept study (as Reviewer 1 also kindly noted) to demonstrate a novel link between these two fields. A full mechanistic dissection would be a substantial undertaking for a future project. The primary hurdle, which is a general problem in the field, is that we still struggle to accurately model cysteine PTMs. Given this lack of fundamental knowledge, randomly mutating other amino acid residues to either replicate or nullify the observed effect would unfortunately not be very informative. First, we must better understand the properties of the modified cysteine in question before we can meaningfully interpret the effects of changes in its surrounding environment.

***To best address the reviewer's concerns**, we introduced new results on the redox regulation of G3BP2 phase separation and replaced the GAPDH experiments. This decision was motivated by two considerations: (1) G3BP proteins are the primary regulators of stress granule (SG) assembly, and (2) G3BP2 contains only a single cysteine, allowing precise*

mechanistic interpretation of redox modifications. Nonetheless, we provide answers to the GAPDH-related questions below for the reviewer only, as those data now form part of a separate study.

*Using chemoselective proteomics, we quantified endogenous redox modifications in 10-month-old mouse brain and observed that ~65% of G3BP2 Cys73 exists as PSOH and ~20% as PSSH (**Fig. 4c**), highlighting the physiological relevance of these modifications. Arsenite-induced stress, a ROS-based stressor and thus highly relevant to aging, elevates global PSOH levels and specifically increases PSOH in G3BP2, while decreasing PSSH. These changes reverse during the recovery phase after stressor removal (**Fig. 5a,b; Extended Data Fig. 9a–c**).*

*We further show that PSOH enhances FRAP recovery, whereas PSO_3H markedly reduces it (**Fig. 4d,e**). Blocking Cys73 with IAM increases droplet size and roundness, indicating that the cysteine contributes to droplet formation (**Extended Data Fig. 8c**). Importantly, treating IAM-blocked protein with H_2O_2 does not alter FRAP recovery (**Extended Data Fig. 8d**), demonstrating that the observed effects require a reactive cysteine.*

*In cells, expression of the C73S mutant enhances SG formation kinetics, mirroring what we observe in $Cse^{-/-}$ cells (**Fig. 5f–i; Supplementary Movie 4**). These effects cannot be attributed to expression-level differences, as confirmed by GFP immunoblotting provided to the reviewer (**Figure 2 for the reviewer**).*

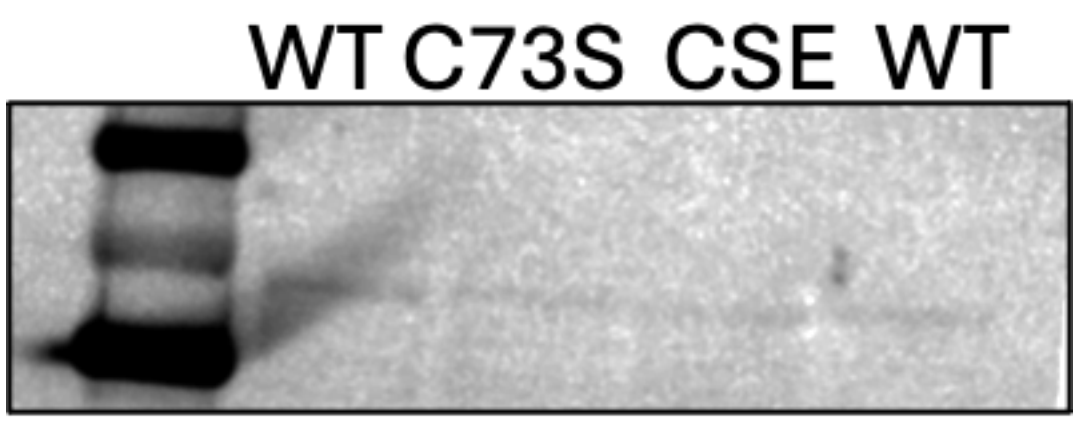

Figure 2 for the reviewer. WB of GFP in cell lysates of MEF cells expressing WT or C73S GFP-G3BP2, or in $Cse^{-/-}$ cell expressing GFP-G3BP2.

*We also demonstrate that PSSH promotes SG dissolution both in vitro and in vivo. To generate PSSH in vitro, we first converted G3BP2 into the G3BP2–TNB adduct using Ellman's reagent, then monitored H_2S-induced conversion to PSSH, which dissolves droplets (**Fig. 5b; Movie 6**). This established method (Cuevasanta et al., JBC 2015; Zivanovic et al., Cell Metab. 2019) yields ~95% PSSH, quantified by TNB release at 412 nm. Addition of H_2S to fully reduced G3BP2, which cannot form PSSH, had no effect on droplet behaviour (**Extended Data Fig. 9d**). In cells, H_2S treatment accelerates SG dissolution, but this effect is absent in cells expressing the C73S mutant, confirming that cysteine persulfidation drives the response (**Fig. 5d; Movies 7–8**). Pharmacological inhibition of H_2S formation blocks SG dissolution during the recovery phase (**Extended Data Fig. 9e**). Consistently, $Cse^{-/-}$ cells fail to resolve SGs even 1 hour after stress removal (**Fig. 5e–g**). To rule out differences in protein abundance, we provide proteomic quantification of key SG proteins, showing identical levels in WT and $Cse^{-/-}$ cells (**Figure 3 for the reviewer**).*

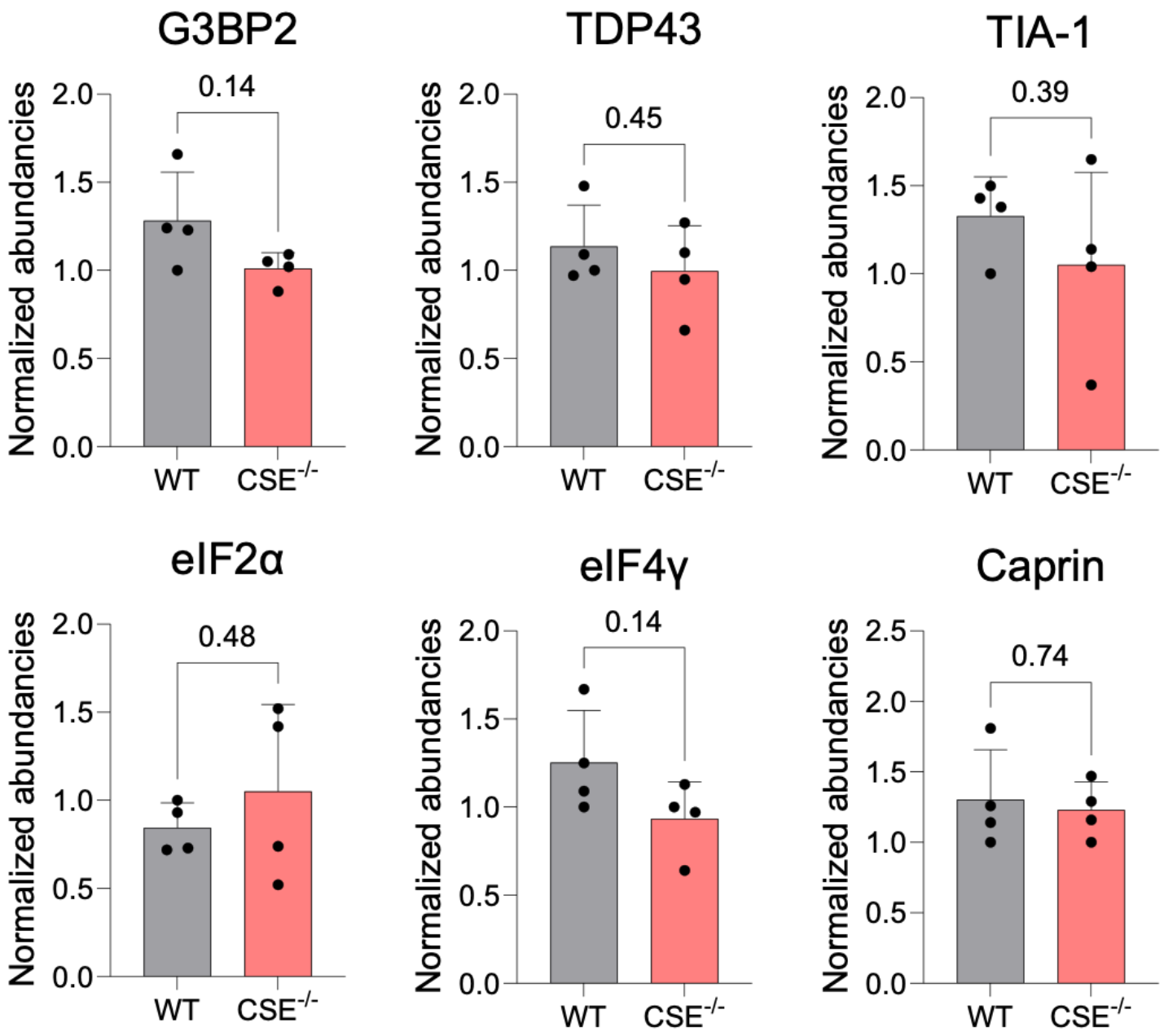

Figure 3 for the reviewer. Levels of key SG proteins in WT and Cse$^{-/-}$ MEFs determined by label-free proteomics.

*We also observe similar SG dissolution defects in striatal neurons from a Huntington's disease mouse model (**Extended Data Fig. 10a,b**), consistent with our prior findings that these cells have markedly reduced CSE and PSSH levels (Zivanovic et al., Cell Metab. 2019). Comparable defects are present in primary human fibroblasts from aged donors (**Fig. 5h–k**). Notably, SG dissolution can be rescued by Na_2S, by the slow-releasing donor GYY4137, and by the naturally occurring compound ergothioneine (**Extended Data Fig. 10c,d**). Ergothioneine requires CSE for conversion to H_2S and PSSH; thus, its effects in WT MEFs can only be attributed to CSE-dependent persulfidation (Petrovic et al., Cell Metab. 2025).*

*Upon oxidative stress, Cse$^{-/-}$ MEFs accumulate more TDP-43–positive granules, with greater colocalization with G3BP2 compared to WT cells (**Supplementary Fig. 7**). These cells also exhibit increased cytosolic localization and higher aggregation of TDP-43 (**Supplementary Fig. 7, 8a**).*

*Finally, in brains of aged Cse$^{-/-}$ mice, we observe elevated aggregation of amyloid-β and TIA-1 (**Fig. 6b,c**). These differences are not attributable to altered disulfide levels, as reducing/non-reducing gel analyses show no genotype-dependent differences (**Supplementary Fig. 8b**).*

Together, these data, supported by the C73S mutant, chemical thiol-blocking controls, consistent droplet behavior in vitro and in cellulo, and opposite responses to H_2S donors versus inhibitors, demonstrate that G3BP2 is a redox-regulated protein whose ability to resolve oxidative stress depends on Cys73 persulfidation.

*To gain mechanistic insight, we performed molecular dynamics simulations of monomeric and dimeric G3BP2, as detailed in our response to Reviewer 1. We summarize relevant observations in **Figure 1 for the reviewer**. The manuscript now discusses these findings as follows: In both G3BP2 and synapsin 1, CysPka predicts exceptionally low apparent pKa values for their redox-active cysteines (≈ −2 for G3BP2 C73 and ≈ −6 for synapsin 1 C360) (Supplementary Fig. 5), indicating that these residues occupy highly thiolate-stabilized reactive hotspots. Notably, mutation of these cysteines to serine or glutamate produced opposite shifts in the hydrophobic character of their local environments: hydrophobicity decreased twofold around G3BP2 C73 but increased approximately twofold around synapsin 1 C360 (Supplementary Fig. 5). These divergent behaviours underscore that local packing effects are highly context-specific and that no single physicochemical parameter*

(e.g., hydrophobicity) uniformly explains the functional outcomes. Likewise, because PSSH, PSOH, and PSO_xH modifications are all expected to be deprotonated at physiological pH, changes in net charge cannot account for the markedly different effects of each oxidation state.

Instead, we propose that persulfidation at such thiolate hotspots acts as a general anti-sticker modification. By extending the cysteine side chain with an additional sulfur atom and increasing local solvation, polarizability, and conformational flexibility,[67] persulfidation disrupts thiolate-dependent contacts and introduces dynamic S–S exchange. These features collectively reduce the effective multivalency of the interaction network. In G3BP2, this could manifest as weakened coupling between the NTF2-like domain, low-complexity regions, and RNA, whereas in synapsin 1 it could diminish the cohesive interactions required for crowding-enhanced condensation with synaptic vesicles. In both systems, the consequence is dissolution of the condensate.

By contrast, irreversible overoxidation to cysteine sulfonic acid introduces a rigid, strongly electronegative SO_xH group that acts as a local anchoring point, akin to phosphorylation,[89] thereby enhancing cohesion and promoting larger, more persistent droplets. Together, these observations indicate that the behaviour of cysteine redox PTMs is best understood through their differential effects on sticker strength rather than through global physicochemical variables such as net charge.

We hope that these new results clearly demonstrate the importance of thiol-based redox modifications in regulating LLPS. Below, we provide answers to the reviewer's questions regarding the previous datasets.

The "shift toward higher-density fractions" observed could be due to formation of cysteine disulfide forms or other cross linked forms, aggregates, etc. Even the in vitro experiments are a concern as the formation of cross linked forms definitely happens when hydrogen peroxide is added to proteins, so I am really not sure how to interpret this.

We followed, to the letter, the method from Li et al. (Nat Chem, 2024)—a state-of-the-art platform using volumetric compression and sucrose gradient fractionation to profile endogenous biomolecular condensates. This method distinguishes between LLPS and higher-order macromolecular assemblies. Their paper validated this approach using FRAP, disorder prediction, and urea/RNase treatments. Protein aggregates do not get retained in these sucrose gradients and they would end up as pellets on the bottom of the tube. Li et al, provide the full description and validation of this elaborate technique that was even cover by the journal's commentary (https://www.nature.com/articles/s41557-024-01503-2). We recognize that, in trying to use absolute state-of-the-art in the LLPS field and include as much supporting data as possible to impress the reviewers, we may have achieved the opposite effect. This is why we removed those data and focused on G3BP2 and stress granules as it provides a cleaner and straight-forward story.

Much more data could be collected on the Cse -/- samples by using chromatography separation, native gel electrophoresis / western, SDS-PAGE (without and with reducing agent) / western.

We appreciate the suggestion to include 1D separations. In our hands, standard SDS–PAGE (and parallel HPLC UV profiles) of WT versus CSE-KO lysates yield virtually indistinguishable band patterns, reflecting the well-documented limitations of 1D approaches to detect subtle redox-induced stability changes or low-abundance modified species, that could only be detected by chemoproteomic approaches. We now include those data as Supplementary Fig 8b. In fact, we could even argue that they support our hypothesis that the CSE ko results predominantly in cysteine hyperoxidation, but that wouldn't be right and would go against our every redox biochemistry fibber.

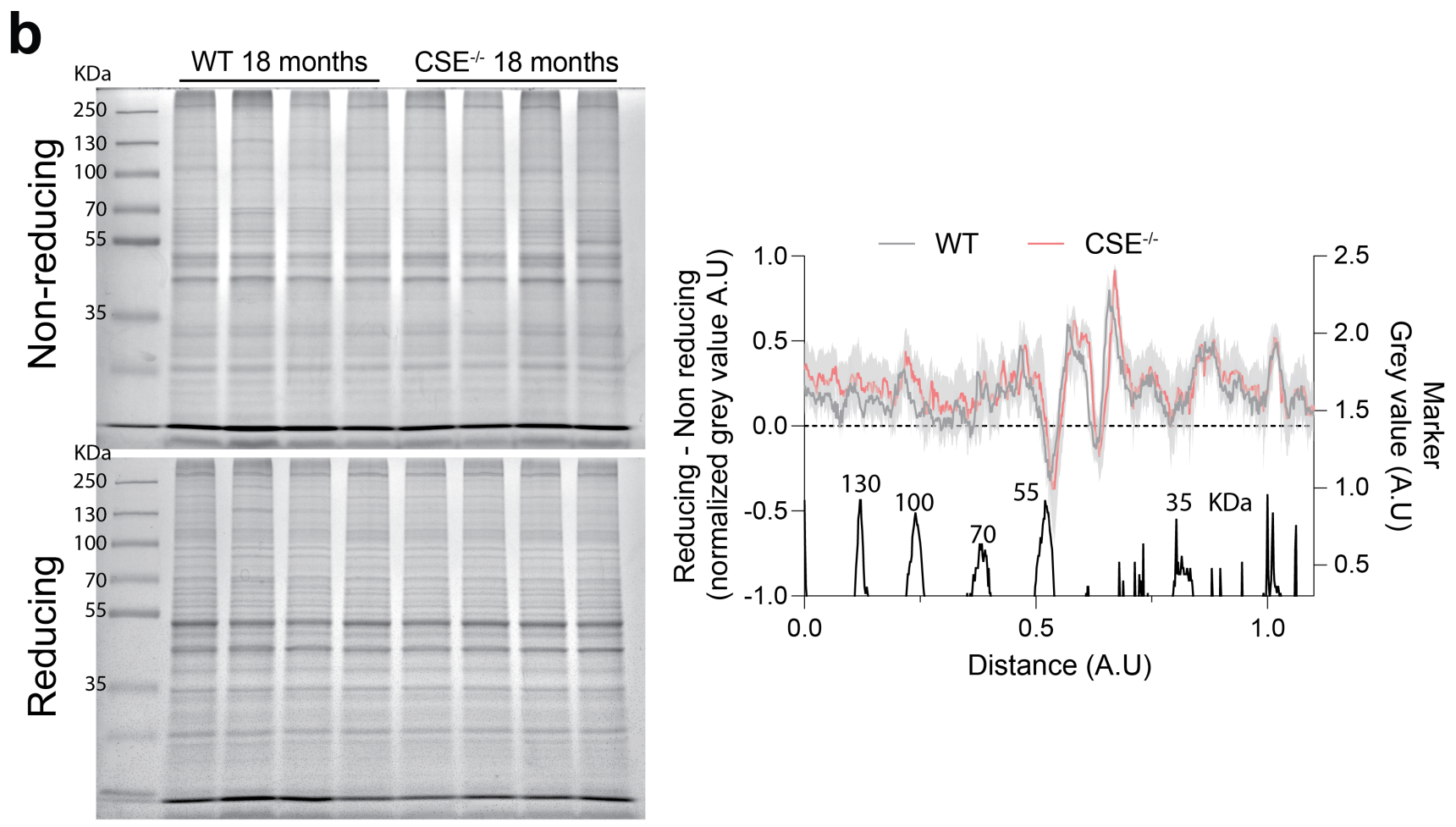

Supplementary Fig 8.

*By contrast, our thermal-proteome profiling (TPP) quantitatively captures alterations in protein thermostability across the proteome. As shown in new **Figure 6a**, dozens of proteins in CSE-KO lysates exhibit a pronounced leftward shift in melting behavior—many collapsing out of solution by 47 °C—whereas their WT counterparts remain soluble through 55 °C. These melting-curve shifts directly report on the thermodynamic consequences of CSE loss, offering mechanistic insight that simple band-intensity comparisons cannot provide.*

Is the modification causing the aggregation or behavior or are proteins modified only as a consequence of destabilization (are they in the process of being disposed of)? In cell or mouse expression and analysis of modification of variants where the folds are disrupted should be examined.

*We clearly show that sulfonylation of GAPDH alters its conformation using ATR-FT-IR and modeling predictions (former Figs. 5i,j and Extended Data Fig. 10). We also show that GAPDH undergoes aggregation only when LLPS is induced in those samples (former Fig. 5k and Extended Data Fig. 10d). We demonstrate that proteins in CSE KO cells exhibit global destabilization using thermal-proteome profiling (**new Fig. 6a**). Sulfonylation enforces LLPS of GAPDH (former Fig. 5g) and drives aggregation only when coupled to phase separation (Fig. 5k). Additionally, we show that once proteins are in the form of condensates, they are more prone to oxidation, presumably due to changes in cysteine reactivity affecting the rate constants with H_2O_2, forming a vicious cycle of protein hyperoxidation (former Extended Data Fig. 9g). We believe this level of information suffices as these data are now removed and constitute manuscript of its own.*

The dissolution of SGs by H2S is interesting but I am not convinced that the mechanism suggested is the only means of achieving this observation. A lot of metabolic changes could be occurring.

This question is addressed above. We provide evidence that the phenomenon occurs both in vitro and in vivo, and that it is absent in the C73S mutant in living cells. Short-term CSE inhibition—a gold-standard approach in the H_2S field—prevents SG dissolution, as does genetic loss of CSE or CSE downregulation associated with Huntington's disease or aging. Conversely, multiple H_2S donors consistently rescue SG dissolution across different cell types. Together, these data demonstrate that the effect is robust, reproducible, and strictly dependent on CSE-mediated cysteine persulfidation.

Additionally, the use of oxidative stress to generate the stress granules and then applying other changes to oxidation is problematic. Inducing stress granules with heat, osomotic shock, etc would be important to show then the effect of H2S.

While we share the reviewers enthusiasm to find answers to all possible questions, we don't understand the logic for the proposed experiments as they are clearly out of scope of this study. We are trying to link age-induced rise in cysteine oxidation and decline in persulfidation to the dysregulation of LLPS. For persulfides to be formed cysteines need to be oxidized either to PSOH or PSSP. To the best of our knowledge no one has studied the effect of heat or osmotic shock on cysteine PTMs. The suggested experiment do not fit into the narrative and distract from the main goal. They could be part of future studies.

GAPDH phase separation could be an in vitro artifact if it requires so much PEG. Is the PEG entering the droplets / does it phase separate with dextran or ficoll, or a protein crowder? If it doesn't phase separate without a crowder, do we know if the phase separation is relevant?

We believe that there is some misunderstanding here. What constitutes too much PEG? We show that GAPDH phase separates at 1 and 3 and 5 % PEG (former Extended data Fig. 8f). We also show that those droplet fuse and show FRAP recovery (former Extended data Fig. 8g,h), suggesting that they exhibit liquid behaviour. ***But most importantly, we performed all our other experiments with RNA not PEG.***

The GAPDH, like the synapsin, has the same issues for the biochemical phase separation. Control experiments and a mechanism needs to be proposed and probed and demonstrated using mutations that prevent and mimic the effects of the modification. The modification sites and numbers need to be examined by mass spectrometry (total and site specific).

Everything explained about the chemical controls for synapsin applies here as well. Mutational studies—particularly of structural or active-site cysteines in a well-structured enzyme—do not seem advisable, as there would be no way to judge whether the enzyme is properly folded and active. The formation of PSOH, PSSH, and PSO_3H has been demonstrated by a selective labelling method and by an antibody against PSO_3H (former Extended Data Fig. 8i,j), and has also been confirmed by MS in vivo (former Fig. 5c, Extended data Fig 8-e). The experiment in which an H_2O_2/DTT mix leads to an increase in droplet size, whereas an H_2O_2/H_2S mix leads to complete dissolution of the droplets, is a chemically perfect control, since only the latter can generate persulfides (former Fig. 5f).

GAPDH has an active site cysteine – is that being modified?

Yes, and we show that by detecting it in aging mice and CSE ko mice (former Extended data Fig 8-e). However, it is not the one that shows the age-dependent increase. We also showed that once "in phase" GAPDH gets inactivated by H_2O_2 faster (former Extended Data Fig 9); this inactivation can only be caused by the oxidation of active site cysteine.

The assay probing the "in droplet" activity is very confusing as the remaining "bulk" form is missing whatever amount of protein is in the droplet, so the comparison is not at all "fair" (unless I misunderstand, there is not the same amount of protein). Even if the amount is the same (are the authors measuring the concentration and then calculating the rates with that?), there are complicated transport questions about droplets (what droplet size) and those are not addressed with this methodology. That the droplets have only 1.5% to 2% of volume (how is that measured?) does not mean that they have a small amount of the protein, they may have a large fraction of the total enzyme. Hence the H2O2 treated experiments are even harder to interpret. There is not in my opinion any evidence to show conformation change that increases catalytic activity.

We apologize that we did not describe this methodology in sufficient detail, as it constituted only a limited part of the study and was presented in former Extended Data Fig. 9. As with Li et al. (Nat. Chem. 2024), we followed the methods developed by Peeples & Rosen (Nat. Chem. Biol. 2021), who were the first to quantify the effect of LLPS on enzyme activity (see also their News & Views: https://www.nature.com/articles/s41589-021-00799-2).

We used the differences in total and bulk-phase protein concentration measured by SDS–PAGE (former Extended Data Fig. 9b). They showed 10 % difference which was within the error of this approach. With the estimated volume (Φ) of 1.5% (following Peeples & Rosen (Nat Chem Biol, 2021)), that results in partitioning coefficient of ~7.5 ($P=[S_{dense}]/[S_{bulk}]=[0.1/0.015]/[0.9/0.985]=7.5$). The observed rate in the heterogeneous system is then a weighted sum of the two microenvironments:

$$v_{tot}=k_{bulk}[S]_{bulk}(1-\Phi) + k_{dense}[S]_{dense}\Phi$$

$$k_{dense} = \{v_{tot} - k_{bulk}[S]_{bulk}(1-\Phi)\}/[S]_{dense}\Phi = \{1.68 * 1 - 0.71[0.9]*0.985\}/[6.67]*0.015\} = 10.3$$

for simplicity: total enzyme conc = 1, total reaction volume = 1

However, even without these calculations, it is clear that centrifugation of droplets to isolate the bulk phase does not remove significant enzyme (former Extended Data Fig. 9b) to account for more than a twofold reduction in reaction rate (former Extended Data Fig. 9c). Indeed, the bulk-phase rate constant is comparable to that of the reaction without a crowding agent (at the same enzyme concentration).

The results of H_2O_2 inhibition are even more compelling: we chose an H_2O_2 concentration that does not inhibit the enzyme over the assay period, so adding H_2O_2 to GAPDH alone produces no significant change in rate. Yet when H_2O_2 is added to a GAPDH solution in which phase separation has been stimulated, clear enzyme inhibition is observed (former Extended Data Fig. 9d–f). This is clearly supported by the BTD-trapping experiment where PSOH formation was trapped to show that once LLPS of GAPDH is induced, even the normally negligible thiol oxidation on air becomes facilitated (former Extended Data Fig. 9g). These data are now removed from the manuscript.

The C245 modeling may be interesting but again mutations should be made to test this hypothesis. The assembly state (covalent and non-covalent) of the protein should be tested not by FTIR but by chromatographic separation, dynamic light scattering, mass photometry, gels (as described above).

*The assembly state is not tested by FTIR. We specifically calculate the abundance of beta sheet domains, and the protein's transition to molten globe state and exposure of amyloid-like site. As already mentioned, protein **does not aggregate** unless it is (i) hyperoxidized and (ii) phase separated (former Fig. 5k, Extended data Fig 10d).*

The authors present the differences upon RNA addition as a surprise but much more thorough characterization of the samples should be performed.

We did not fully understand this comment. What we found surprising is that GAPDH undergoes phase separation, as a well-structured protein without intrinsically disordered domains. Furthermore, GAPDH is found in several global proteome analyses of LLPS and SG. Finally, not only GAPDH, but PKM and possibly several other glycolytic enzymes undergo LLPS with RNA despite their well structured nature (former Figs 4b,d,e,f).

Point-by point answers to the reviewers

We thank all 3 reviewers for their comments
Reviewer #1 (Remarks to the Author):

The authors have addressed all my concerns. The manuscript at this version can be accepted as it is, at least in principle.

[ADDITIONAL COMMENTS]
I agree with the concerns raised by Reviewer #3. The predicted apparent pKa values of approximately -2 and -6 for these cysteines are exceptionally low. While negative pKa values are chemically possible for extremely strong acids, they are highly unusual for cysteine thiols in a biological context. My main concern is that these values are derived from computational predictions rather than experimental validation. Given how unusual these numbers are, it is difficult to assess whether they represent a genuine biochemical insight or if they might result from the specific parameters or limitations of the prediction algorithm (or possibly even a typographical error in the figure). Using such predictions as mechanistic evidence without experimental confirmation weakens that specific argument. However, I believe this result could be simply removed without invalidating this interesting and comprehensive study.

Rev1: We thank the reviewer for the support and agree that these computationally identified pKa values are distracting from our data and we therefore removed any mentioning of the data.

Reviewer #2 (Remarks to the Author):

All my concerns are addressed. I don't have further comments.

[ADDITIONAL COMMENTS]
I do share the same concern as reviewer #3. I think confirming the tau pathology is a very critical point in this paper. I wrote in my initial comments “The neurofibrillary tangle shown in Fig 2j doesn’t look like neurofibrillary tangle at all. This observation needs to be confirmed using many of commercially available antibodies against pathological tau.”

However, the author responded “The reviewer is right that staining with tau antibodies is the best way to show the neurofibrillary tangles. Gallaya’s staining (with all its limitations) has been in use in confirming postmortem AD from human samples long before the introduction of tau (Moloney et al, Visualization of neurofibrillary tangle maturity in Alzheimer’s disease: A clinicopathologic perspective for biomarker research, Azlheimer & Dementia, 2021). The reason we did not include tau is that in collaboration with Prof Paul we have another manuscript which is dealing with phenotyping of CSE ko mice (Chakraborty et al, 2025, accompanying study for reviewer only). We show that those mice not only have accumulation of tau fibrils but a full cognitive phenotype reminiscent of AD and/or dementia.”

In this context, I can accept this response and the data provided, but want to stress that some of the most important data of the study is saved for another publication.

Rev2: We thank the reviewer. Indeed, the study uploaded for the reviewers only is currently under revision. We agree that Gallyas staining alone does not provide the molecular specificity of modern Aβ and phospho-tau immunohistochemistry. However, silver impregnation methods, including the Gallyas technique, were historically regarded as a gold-standard neuropathological approach for identifying Alzheimer-type lesions in human postmortem brain tissue prior to the widespread characterization of tau pathology and the development of tau-specific immunostaining methods. We therefore rephrased neurofibrillary tangles and used the term “neuritic-like plaques” to describe argyrophilic plaque-associated neuritic structures without overinterpreting their molecular composition. As demonstrated in our accompanying manuscript that was submitted for the reviewers only, we have extensively characterized the tau staining and indeed confirmed higher tau aggregation in aging $Cse^{-/-}$ mice.

Reviewer #3 (Remarks to the Author):

"In both G3BP2 and synapsin-1, CyspKa predicts exceptionally low apparent pKa values for their redox-active cysteines (≈ −2 for G3BP2 C73 and ≈ −6 for synapsin-1 C360) (Supplementary Fig. 5), indicating that these residues occupy highly thiolate-stabilized reactive hotspots."
This seems like an unrealistic pKa (negative numbers) and so it is not clear if this is reliable. Furthermore, the authors repeat several times that "the reaction rate for the oxidation of cysteine is approximately two orders of magnitude faster than that for methionine" but this ignores that structure and context could dramatically affect reaction rate (if pKa shifts from pH ~7.8 to pH -6, 12 orders of magnitude due to hotspots, the reaction rate will also be highly dependent on details).

A negative predicted pK_a does not imply that the cysteine is buried; rather, it indicates strong stabilization of the thiolate state by the local electrostatic environment. This typically reflects a highly reactive cysteine. Modification at this site would neutralize or alter the thiolate, thereby perturbing local interactions and potentially affecting protein function. Nonetheless we removed that following suggestions of the rev 1.

The biggest issue is that G3BP2 is a good idea but the example shows that the mechanism, which would be required for this journal in my view, is not at all probed or explained by the data. The pKa being negative basically means this is a buried cysteine. So what does modification here do to the protein? Some biophysical and structural evaluation of the changes (circular dichroism, NMR spectroscopy, molecular simualtions) caused by distinct modifications could be done (the modifications could be made in the recombinant protein). With a single cysteine, lots of different modifications could be explored in addition to the modifications that are made by the various oxidation states. Also, the idea that mutations to the 20 natural amino acids cannot cannot capture all the dimensions of the cysteine modifications is true, but surely they will capture some of the changes. Either way, the data are not adequate to support the claims.

We have included the statement in our manuscript to acknowledge that no one amino-acid replacement can fully account for the physico-chemical properties of the cysteine PTMs studied by us: "Likewise, because PSSH, PSOH, and PSOxH modifications are all expected to be deprotonated at physiological pH, changes in net charge cannot account for the markedly different effects of each oxidation state." Although we provided, for the reviewer only, MD simulations for some of the studied cysteine PTMs we also acknowledged that "Future detailed computational studies will provide physicochemical explanations for these observations." Addressing all other cysteine PTMs was out of the scope of this study and will be subject of our future studies. Due to their intrinsic instability and reactivity (PSOH, PSSH), some of these modifications cannot be studied by techniques that require long recording times such as NMR etc.

The neurofibrillary tangles are still not stained as requested by reviewer to confirm their identity.

We agree that Gallyas staining alone does not provide the molecular specificity of modern Aβ and phospho-tau immunohistochemistry. However, silver impregnation methods, including the Gallyas technique, were historically regarded as a gold-standard neuropathological approach for identifying Alzheimer-type lesions in human postmortem brain tissue prior to the widespread characterization of tau pathology and the development of tau-specific immunostaining methods. We therefore rephrased neurofibrillary tangles and used the term "neuritic-like plaques" to describe argyrophilic plaque-associated neuritic structures without overinterpreting their molecular composition. As demonstrated in our accompanying manuscript that was submitted for the reviewers only, we have extensively characterized the tau staining and indeed confirmed higher tau aggregation in aging $Cse^{-/-}$ mice.
